# Intermolecular Enantioselective Nickel‐Catalyzed Arylation of Un‐Activated C(sp^3^)─H Bond

**DOI:** 10.1002/anie.202523241

**Published:** 2026-01-05

**Authors:** Erwan Brunard, Fengjie Huang, Àlex Díaz‐Jiménez, Sofiya Kostiukovska, Joanna Wencel‐Delord

**Affiliations:** ^1^ Institute of Organic Chemistry JMU Würzburg, Am Hubland 97074 Würzburg Germany

**Keywords:** Arylation, Asymmetric catalysis, C─H Activation, Quaternary stereocenter

## Abstract

We report the first example of an intermolecular, enantioselective Ni(II)‐catalyzed C(sp^3^)─H arylation, providing direct access to challenging all‐carbon quaternary stereogenic centers. Key to this success is the use of a specially designed BINOL‐derived ligand, which enables efficient desymmetrization of gem‐dimethyl groups through a rate‐ and enantio‐determining C─H activation step. This catalytic system demonstrates remarkable functional group tolerance, affording a wide variety of arylated products in high yields (up to 85%) and with excellent enantioselectivities (up to 95:5 e.r.). Mechanistic studies, combining kinetic isotope effect measurements, deuterium labeling, and DFT calculations, reveal that the Ni–BINOL complex promotes a unique concerted metalation–deprotonation pathway assisted by ligand coordination and π–π interactions. This work establishes nickel as a powerful and sustainable alternative to precious metals for asymmetric C─H activation, opening new perspectives for the construction of enantioenriched quaternary carbon centers of high relevance in drug discovery and materials science.

## Graphical abstract

Ni: a new solution for asymmetric C(sp^3^)─H activation. The stereoselective synthesis of all‐carbon quaternary stereogenic centres is achieved via enantioselective C(sp^3^)─H arylation using a Ni‐BINOL‐derived catalyst. Rational catalytic cycle design uncovers a new reaction pathway that can efficiently induce chiral information during the stereoselective and rate‐determining metalation event.

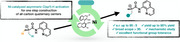

## Introduction

**Figure 1:**
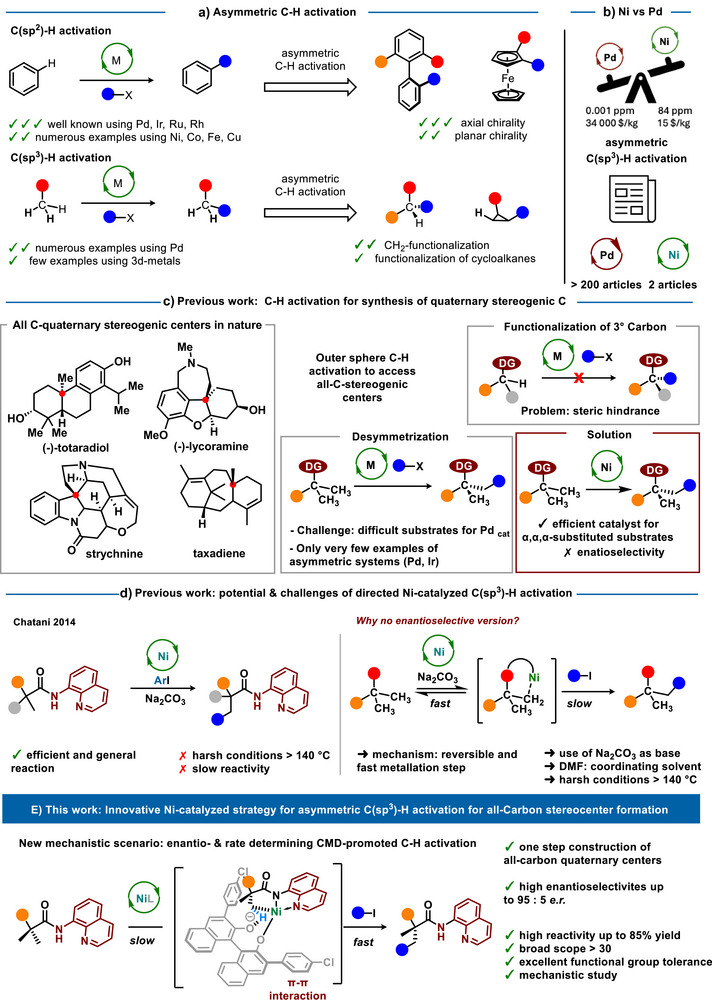
Development of a Ni(II)‐catalyzed enantioselective C(sp^3^)─H arylation enabling efficient access to all‐carbon quaternary stereocenters.

Over the past twenty years, the field of enantioselective catalysis has seen significant growth. While asymmetric synthesis has been investigated since the 1970s, the demand for optically pure compounds has increased considerably, driven not only by pharmaceutical chemistry^[^ ^1^, ^2^ ^]^ but also by agrochemistry^[^ ^3^ ^]^ and materials science.^[^ ^4^ ^]^ Both the development of sustainable enantioselective synthetic methodologies and the design of original chiral scaffolds are thus of prime importance. In this context, asymmetric C─H activation proved to be a real game‐changer.^[^ ^5^, ^6^, ^7^, ^8^ ^]^ As the C─H activation strategy is particularly appealing in the case of aromatic compounds,^[^ ^9^, ^10^, ^11^, ^12^, ^13^, ^14^, ^15^ ^]^ implementation of the C(sp^2^)─H activation mindset to access a diversity of enantiomerically pure atropoisomeric compounds,^[^ ^16^, ^17^, ^18^, ^19^, ^20^, ^21^, ^22^ ^]^ planar chiral molecules,^[^ ^23^, ^24^ ^]^ or helicoidal scaffolds^[^ ^25^ ^]^ allowed to revolutionize the synthesis of such compounds (Figure 1a). In sharp contrast, the generation of C‐stereogenic molecules via direct activation of C(sp^3^)─H bonds is less developed, primarily due to the low reactivity of aliphatic substrates toward metalation.^[^ ^26^, ^27^, ^28^ ^]^ Over the last years, Yu,^[^ ^29^, ^30^, ^31^, ^32^, ^33^ ^]^ Boudoin,^[^ ^34^ ^]^ Cramer,^[^ ^35^ ^]^ Phipps,^[^ ^36^ ^]^ Duan^[^ ^37^ ^]^ and Shi,^[^ ^38^ ^]^ showed that desymmetrization reactions of the CH_2_ motif can be astutely used to reach diversity of enantiomerically enriched compounds.^[^ ^1^ ^]^ Besides, enantiopure cyclopropanes^[^ ^39^, ^40^, ^41^, ^42^ ^]^ and cyclobutanes,^[^ ^31^, ^43^ ^]^ as well as indane‐derived compounds,^[^ ^44^ ^]^ can thus be afforded.

An especially elegant and synthetically valuable application of asymmetric C(sp^3^)─H activation involves the functionalization of gem‐dimethyl groups. Resulting all‐carbon quaternary stereogenic centers are, indeed, key motifs in drug design.^[^ ^45^ ^]^ However, their enantioselective synthesis remains a well‐recognized challenge.^[^ ^46^, ^47^, ^48^, ^49^ ^]^ In particular, literature examples of successful implementation of the C─H activation to access all‐carbon quaternary stereogenic centers remain extremely rare (Figure 1c). Pioneering work by Yu on Pd‐catalyzed transformations,^[^ ^50^ ^]^ followed by sporadic reports from Matsunaga^[^ ^51^, ^52^, ^53^, ^54^, ^55^ ^]^ and Dixon^[^ ^56^ ^]^ on Rh(III) and Co(III) catalyzed aminations and alkynylations, along with the recent Ir‐catalyzed borylation,^[^ ^57^ ^]^ clearly illustrates the challenging nature of such transformations. In contrast, Chatani^[^ ^58^ ^]^ demonstrated in 2014 that a Ni‐catalyst efficiently promotes the direct arylation of benzyldimethylacetic acid bearing an 8‐aminoquinoline directing group^[^ ^59^ ^]^ (Figure 1d). The high reactivity and sustainability of nickel, approximately 2000 times less expensive and 10⁴ times more abundant than palladium, make this reaction a highly appealing platform for developing sustainable^[^ ^60^ ^]^ asymmetric routes to all‐carbon quaternary stereocenters (Figure 1b). However, from a fundamental and mechanistic viewpoint, a key difficulty can be rapidly defined. Stereoselective desymmetrization of gem‐dimethyl groups is expected to occur via a rate‐ and enantio‐determining C─H activation step, as irreversible metalation should warrant high stereoinduction.^[^ ^53^, ^56^ ^]^ However, as demonstrated by Chatani, Ni‐based catalytic system proceeds via a reversible C─H activation step.^[^ ^61^ ^]^ This fundamental difference clearly renders Ni‐catalyzed C─H activation extremely difficult to adapt for enantioselective transformation. The design of an alternative catalytic system, capable of reversing the “energy barriers” of the metalation event versus oxidative addition and/or reductive elimination, is therefore necessary.

Other difficulties, including elevated reaction temperatures,^[^ ^62^, ^63^ ^]^ which undermine stereochemical control, or a wide range of Nickel oxidation states (from 0 to +IV),^[^ ^64^ ^]^ engaging in both single‐ and two‐electron redox processes, further exacerbate the difficulty.^[^ ^65^, ^66^ ^]^ Furthermore, unlike palladium with its well‐defined coordination patterns, Ni(II) complexes display variable coordination numbers (4, 5, or 6) and adopt diverse geometries, including square planar, tetrahedral, trigonal bipyramidal, square pyramidal, and octahedral.^[^ ^67^, ^68^, ^69^ ^]^ As a result, conceptualizing a chiral Ni‐complex capable of efficient chiral induction remains a formidable challenge. To date, the enantioselective intramolecular annulation of formamides enabled by a phosphine‐oxide‐ligated Ni/Al bimetallic catalyst represents the only reported example of asymmetric Ni‐catalyzed C(sp^3^)─H activation for C─C bond formation.^[^ ^70^ ^]^ However, during the preparation of this manuscript, Shi and co‐workers disclosed a complementary Ni(II)/BINOL‐catalyzed C(sp^3^)─H amination, further underscoring the emerging yet still highly limited landscape of asymmetric nickel‐based C─H functionalization.^[^ ^71^ ^]^

Herein, we present the first example of asymmetric intermolecular Ni‐catalyzed C(sp^3^)─H arylation (Figure 1e). Building on the pioneering work of Shi and co‐workers on Ni(II)/BINOL‐catalyzed C(sp^2^)─H activation,^[^ ^72^ ^]^ which established the fundamental reactivity and coordination behavior of BINOL‐based ligands in nickel catalysis, we sought to extend this reactivity manifold to the far more challenging C(sp^3^)─H activation. Thanks to the bicoordinating and hemilabile character of the BINOL ligand: (S)‐3,3′‐bis(4‐chlorophenyl)‐[1,1′‐binaphthalene]‐2,2′‐diol (**L6**), increased reactivity of Ni‐complex could be reached, and the expected altered mechanistic scenario operates, with the C─H activation being rate‐ and enantio‐determining step, thus enabling enantioselective metalation and further arylation. This unique 3d‐metal catalyzed asymmetric C─H activation shows impressive functional group tolerance and warrants an efficient approach toward all carbon quaternary chiral centers with high yields and unprecedented enantioselectivities. Indepth mechanistic studies, combining experimental investigations and DFT calculations, demonstrate the unique reactivity of our catalytic system, thus paving the way toward the long‐desired reactivity. (Figure 1e)

## Results and Discussion

While targeting the synthesis of an all‐Carbon quaternary stereogenic center by Ni‐catalyzed asymmetric C─H activation, benzyldimethylacetic acid substrate bearing an 8‐aminoquinoline (AQ) directing group was selected as the model substrate **1aa**. The AQ auxiliary is commercially available (approx. 1 €/g), can be easily installed on and removed from the carboxylic acids, and has excellent versatility in C─H functionalizations.^[^ ^73^ ^]^ We hypothesized that efficient stereoinduction should be possible if the metalation step is enantio‐determining and irreversible. Besides, as the substrate **1aa** undergoes C─H metalation quite rapidly using simple Ni‐precatalysts,^[^ ^58^ ^]^ design of a more reactive catalytic system is essential to outcompete the racemic background reaction with a non‐ligated Ni‐source. Based on this analysis, we surmised that 1) replacement of the commonly used in the non‐chiral reactions coordinating solvent DMF is crucial, 2) use of carbonate base seems also fundamentally incompatible with our targeted reaction, as its participation in the CMD‐type metalation event might be expected to enhance the racemic pathway. Accordingly, we have selected dioxane as the possible solvent together with alkoxide base, such as sodium *tert*‐butoxide, as demonstrated by Johnson for their unique reactivity toward these systems.^[^ ^69^ ^]^ The nickel source remained nickel triflate Ni(OTf)_2_, which demonstrated excellent reactivity under these conditions.

**Figure 2:**
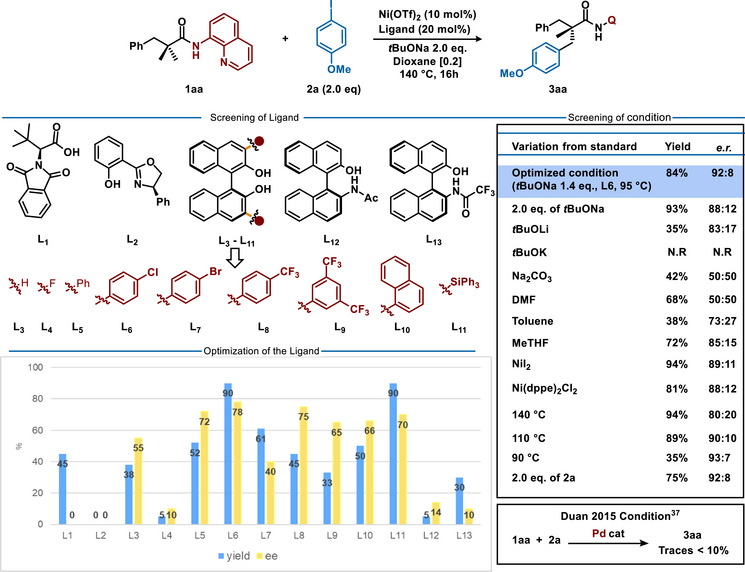
Optimization of the reaction conditions for Ni(II)‐catalyzed enantioselective C─H arylation.

While aiming for asymmetric base‐assisted metalation, the initial catalytic tests began by screening several families of ligands (Figure 2). Chiral N‐protected carboxylic acid **L1** promoted the desired reaction, albeit no chiral induction was observed. In contrast, Salox **L2,** shut down the desired reactivity. The first promising reaction outcome was observed while performing the reaction in the presence of a BINOL (**L3**), as the desired product was afforded in 77.5: 22.5 e.r. and 38% yield. Subsequent ligands’ optimization study revealed that substitution at positions 3 and 3′ of BINOL is essential.^[^ ^72^ ^]^ Fluoro‐substituted ligand **L4** delivered the desired product in trace amounts, whereas 3,3′‐aryl substituted ligands appeared as a clear hint toward more selective transformation. While adjusting the electronic and steric properties of the BINOL ligand, 4‐chlorophenyl substituted BINOL **L6** outcompeted other congeners, delivering the desired compounds in 89:11 e.r. Importantly, other binaphthyl‐based ligands, such as **L12** and **L13** exhibited very poor stereocontrol. Further reaction optimization revealed several important features. Consistent with our initial hypothesis, use of the carboxylate base or the coordinating DMF solvent totally inhibited the chiral induction. While *tert*‐butoxide base is essential, the choice of the counterion is not innocent, with the optimal results being achieved only in the presence of sodium *tert*‐butoxide. The amount of NaO*t*Bu is also a key parameter. An excess of base reduces the product's enantiomeric excess by favoring the racemic background reaction. In contrast, using 1.4 equivalents proves optimal, as it ensures selective coordination of the substrate to the metal while enabling the stoichiometric deprotonation of the BINOL ligand.^[^ ^69^ ^]^ While dioxane appears as the optimal solvent, use of toluene or Me‐THF proved to be deleterious to both catalytic activity and enantioselectivity. The source of the Ni‐precatalyst has a minor impact on the reaction outcome. Remarkably, use of the optimized BINOL **L6** allows for decreasing the reaction temperature as well, thus overcoming a major limitation of nickel‐catalyzed C─H activation, as previously described by Chatani (typically high temperatures >140 °C are required).^[^ ^62^ ^]^ To our delight, reaction temperature could be decreased to 95 °C, while reaching high conversion of the substrate within only a few hours, thus demonstrating higher reactivity of our catalytic system compared to the pioneering conditions. As expected, lowering the reaction temperature facilitated efficient chirality transfer and the desired product could be isolated in 84% yield and enantiomeric ratio of 92:8. Further recrystallization allows for affording the optically pure S‐**3aa** (99:1 *e.r.;* CCDC 2472361). To benchmark our methodology against previously reported palladium‐based conditions, we tested our new catalytic system in two scenarios: first, under our conditions with palladium as the catalyst, and second, under the conditions developed by Duan.^[^ ^37^ ^]^ In the first case, no product formation was detected. In the second, only traces of the desired product were obtained, displaying a modest 55:45 e.r. after isolation. These results highlight the distinct performance of nickel versus palladium to promote quaternary center via C─H activation.

### Scope

**Figure 3:**
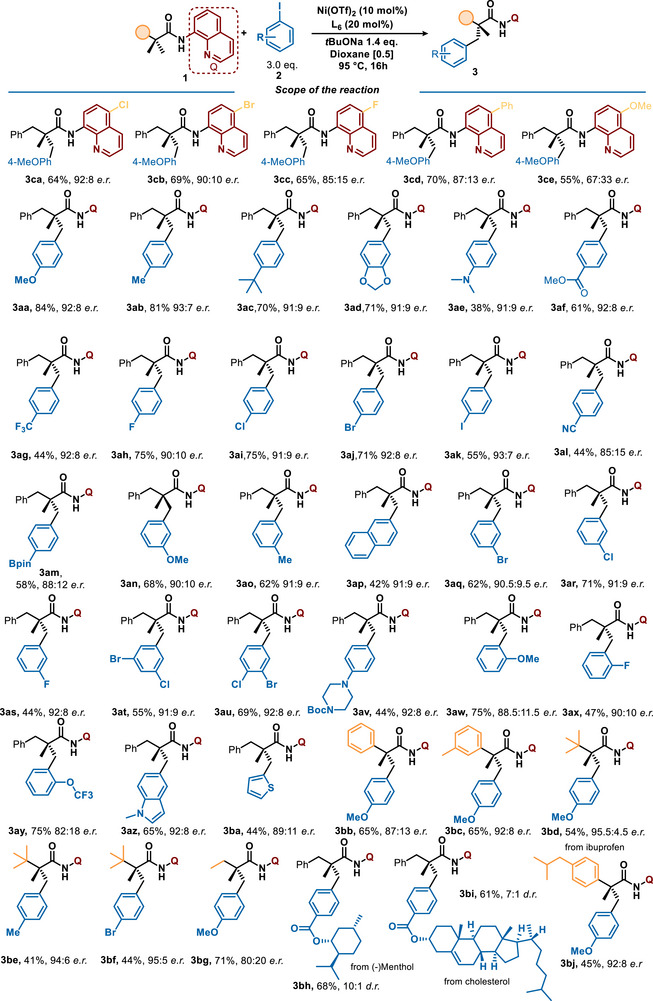
Scope of enantioselective nickel‐catalyzed direct arylation of aliphatic amides with aryl iodides.

With the optimal reaction conditions, the scope of this reaction was explored (Figure 3). Initially, modifications of the 8‐aminoquinoline directing group were studied. Rewardingly, similar results were obtained while using substrates bearing Cl‐ and Br‐ substituents at C4‐position (**3ca** and **3cb**), allowing for potential post‐functionalization. A slight decrease in the reaction efficiency is noticeable, using fluoro‐ and phenyl‐substituted aminoquinoline directing groups (**3cc** and **3cd**). We also evaluated the methoxy‐substituted substrate **1ce**, considering the utility of this group in downstream deprotection.^[^ ^74^ ^]^ However, only moderate enantioselectivity was obtained, presumably arising from new, undesired interactions with the ligand. When considering the iodoarene coupling partners, the reaction proved to be exceptionally general. Introduction of electron‐rich substituents in the *para*‐position, including aliphatic motifs (**3ab**, **3ac**), did not modify either the yield or the chiral induction significantly. Lower yield observed while using dimethylamine‐substituted coupling partner can be possibly attributed to the coordinating properties of this group (**3ae**). Presence of electron‐withdrawing motifs, including ester (**3af**), cyano‐ (**3al**), and CF_3_‐groups (**3ag**) is also generally well tolerated. Notably, the reaction occurred smoothly in the presence of halogen substituents, including bromo‐ and iodo‐atoms (**3ah**–**3ak**), thus demonstrating a remarkable chemoselectivity and its complementarity with respect to Pd‐catalyzed systems. Besides, Bpin‐aryl coupling partner could also be employed as a viable substrate (**3am**), albeit a slight decrease in the enantioselectivity was observed. *Meta‐*substituted aryl‐iodides could be converted into the desired products smoothly, furnishing the C‐stereogenic compounds in comparable and synthetically useful yields (**3an**–**3au**). Synthesis of the naphthyl‐substituted product was equally selective, albeit **3ap** was isolated in 42% yield. While using bromo‐chloro‐iodo‐benzene, selective arylation was observed, furnishing **3at** in 91:9 e.r. Thanks to the decreased reaction temperature, a Boc‐protected aryl‐iodide coupling partner was successfully used, while fully maintaining the protecting group, and **3av** was thus afforded in 44% yield. While considering the *ortho*‐substituted coupling partners, small substituents, such as methoxy‐, fluoro‐ and OCF_3_ are well tolerated (**3aw**–**3ay**). The potential of this reaction could be further extended toward heterocyclic aryl iodides, as supported by the synthesis of indole‐ and thiophene derivatives (**3az**–**3ba**).

While exploring the substrate's scaffold modification, a further increase in steroinduction was observed. The use of trialkyl‐substituted starting material turned out to be particularly appealing for its potential steric repulsion. Indeed, desymmetrization of the substrate bearing a *t*Bu in *alpha* of the quaternary center occurred smoothly, affording **3bd** in 95.5: 4.5 e.r. Other aryl iodides could be efficiently coupled with this substrate, furnishing **3be** and **3bf** in the same range of enantioselectivity. Substituting the benzyl group at the prochiral carbon with a phenyl ring yielded products **3bb** with 65% efficiency and e.r. of 87:13, demonstrating the ligand's ability to differentiate between substituent positions of the phenyl ring. Introducing a methyl group at the *meta* position further enhanced the enantioinduction, achieving an e.r. of 92:8 for product **3bc**. Similarly, introduction of an ethyl substituent only slightly reduces the system's efficiency, while still preserving a significant level of asymmetric induction, affording **3bg** in 71% yield with an encouraging 80:20 e.r.

Encouraged by the generality of our Ni‐catalyzed C(sp^3^)─H arylation, we embarked on demonstrating its utility in late‐stage diversification. As expected, aryl‐iodides derived from menthol and cholesterol could be used efficiently, supplying the complex products **3bh** and **3bi** in high yield and diastereoselectivities. Besides, direct arylation of the ibuprofen‐derived substrate **1bj** occurred smoothly; the ibuprofen's congener bearing all‐Carbon quaternary stereocenter was formed in 92:8 e.r. and 45% yield.

**Figure 4:**
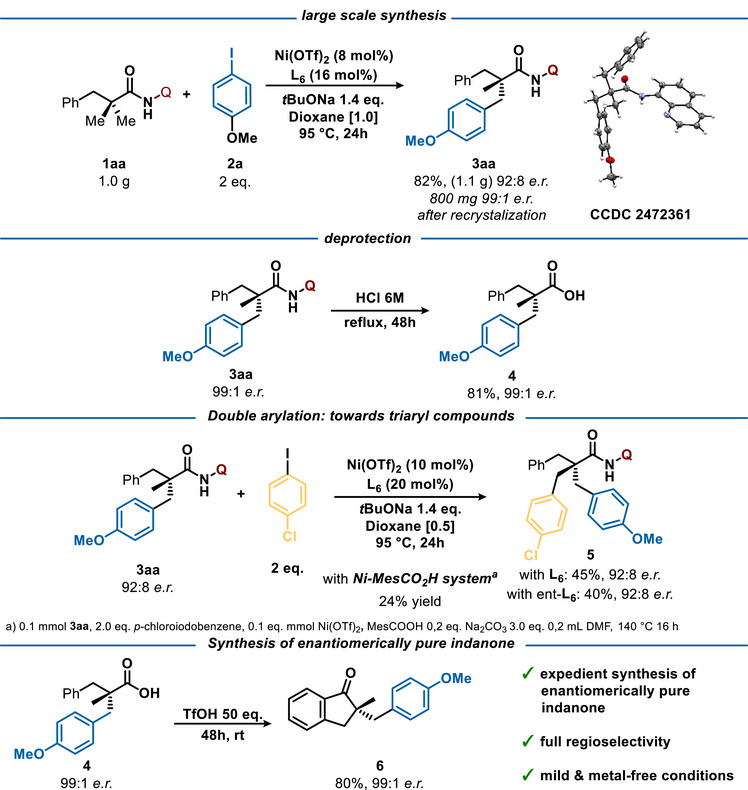
Gram‐scale amide synthesis and application in further functionalizations.

To further demonstrate the synthetic utility of this protocol, a large‐scale reaction was conducted using **1aa** as the standard substrate. Despite the reduced catalyst loading, the desired product was isolated without significant loss of either efficiency or stereoselectivity. After recrystallization, **3aa** was obtained in 60% yield (800 mg) and >99% ee. Remarkably, not only the unreacted substrate but also the ligand could be recovered from the reaction mixture, further emphasizing the sustainability and cost‐effectiveness of this methodology (Figure 4). Under acidic conditions, the 8‐aminoquinoline directing group can be removed, delivering the enantiopure carboxylic acid **4** in 81% yield.^[^ ^75^ ^]^

Expansion of the chemical space of chiral compounds accessible via this approach could be reached via a second direct arylation of **3aa**. Under our standard reaction conditions, functionalization of **3aa** proceeded efficiently, delivering the desired tribenzyl all‐carbon quaternary stereogenic center product **5** in a synthetically useful yield. Interestingly, **5** was isolated with the same optical purity as the starting material, regardless of the enantiomer of the ligand **L6** used, clearly showing that while Ni‐BINOL system can desymmetrize two Me‐groups, kinetic resolution of the chiral product **3aa** does not occur. Interestingly, while the second arylation was attempted using original Chatani´s conditions^[^ ^58^ ^]^ the desired product was isolated in significantly lower yield of 24%. Finally, encouraged by the ease of the directing group removal, a possible cyclization was explored. Remarkably, the enantiopure carboxylic acid **4** was smoothly converted into the desired chiral indanone **6** at room temperature in the absence of a metal catalyst. This regioselective cyclization occurs with full chirality conservation, thus providing an expedient route toward the chiral indanone, an important scaffold in medicinal chemistry and natural products.^[^ ^76^ ^]^

Encouraged by the unique reactivity and broad applicability of our Ni‐based catalytic system for asymmetric C(sp^3^)─H activation, we undertook mechanistic studies combining experimental and theoretical approaches. In our initial hypothesis, C─H activation step was expected to be rate‐determining. To validate this hypothesis, KIE studies were conducted.^[^ ^77^ ^]^ Both independent measurements of the initial reaction rates, as well as the competition experiments, point toward relatively high KIE values of 2.4 and 4.0, respectively, thus clearly supporting the rate determining nature of the metalation event. In addition, the scrambling experiment revealed no D–H exchange, further indicating the irreversibility of this step. Besides, the deuteration studies clearly showcase the mechanistic divergence between our catalytic system and the racemic one (Ni‐MesCO_2_H system).^[^ ^58^ ^]^

To understand the nature of the Ni‐BINOL complex, our attention subsequently focused on the non‐linear effect study. The linear correlation between the optical purity of the ligand and the enantioselectivity was experimentally confirmed. Interestingly, upon reacting the Ni precatalyst with **L6** in the presence of *t*BuONa, new metallic species (**C‐Ni‐1**) were observed. X‐Ray analysis of C‐Ni‐1 indicates the formation of a **Ni–(L6)_2_** complex. The most interesting is, however, the fact that the BINOL‐derived ligand seems to adopt a bicoordinating mode, with one O‐atom acting as X ligand and one O‐atom acting as L‐ligand, adopting pseudo planar geometry (structure pointed by two Na‐cations). This structure seems coherent with the importance of counteranion present in our reaction (optimization study), while supporting the hypothesis that the BINOL ligand adopts its coordinating properties to efficiently promote the CMD‐metallation, thus acting as a surrogate to largely explored carboxylic acid‐ligands in Pd‐catalyzed C─H activation. While C‐Ni‐1 can promote the arylation reaction, as the expected product was obtained in 64% and 90:10 e.r. (for details see Supporting Information, Section 7.5), a higher temperature of 120 °C is requested. Therefore, it can be expected that a productive precatalyst contains only one molecule of ligand **L6**, as supported by the absence of NLE.

### Density Functional Theory (DFT) Calculations

Based on the experimental results obtained (vide supra), several mechanistic questions arose concerning the unique behavior of our system. Specifically, attention was drawn to: 1) the non‐innocent role of the sodium counterion; 2) the involvement of the BINOL ligand throughout the course of the transformation; 3) the rate‐determining and irreversible nature of the C─H activation step; and 4) the origin of enantioselectivity. To address these points and gain deeper insight into the underlying reaction mechanism, density functional theory (DFT) calculations were performed using the Gaussian 16 software package at the B3LYP‐D3/6‐311+G**–LANL2DZ(Ni,I)–SMD(dioxane)//B3LYP‐D3/6‐31G*–LANL2DZ(Ni,I) level of theory at 373 K (Figure 6; see the Supporting Information for full computational details).

The isolation of complex **C‐Ni‐1** suggests a close interaction between the sodium cation and the BINOL moiety. Therefore, our proposed catalytic cycle begins with coordination intermediate INT1, featuring the sodium unit between the binolate fragment and the carbonyl group of the amide moiety. From INT1, the formation of INT2 proceeds in a mildly endergonic manner. In INT2, the nickel center is placed near a methyl group (2.34 Å), while one of the pendant BINOL oxygens lies in close proximity to a hydrogen atom of that methyl unit (2.31 Å). The subsequent C─H activation step occurs with assistance from the BINOL ligand, wherein hydrogen abstraction is facilitated by the pendant oxygen, concurrent with Ni─C bond formation. This step requires an activation barrier of 26.7 kcal mol^−1^, consistent with the temperature required experimentally (approx. 95 °C), and yields intermediate INT3. Notably, this mode of C─H activation differs from literature‐reported examples, which typically involve strong bases to deprotonate the C─H bond. These findings highlight the role of BINOL as a CMD ligand.

Previous DFT studies on non‐asymmetric nickel‐catalyzed C(sp^3^)─H arylation by the groups of Liu^[^ ^78^ ^]^ and Sunoj^[^ ^79^ ^]^ reported a ligand exchange between iodoanisole and the pendant ligand in the nickel center, followed by oxidative addition as the rate‐determining step of the reaction. Based on these precedents, ligand exchange followed by oxidative addition of the iodoaryl in our system requires an extremely high barrier of 34.2 kcal mol^−1^ (Figure S2), which is inconsistent with our experimental conditions.

**Figure 5:**
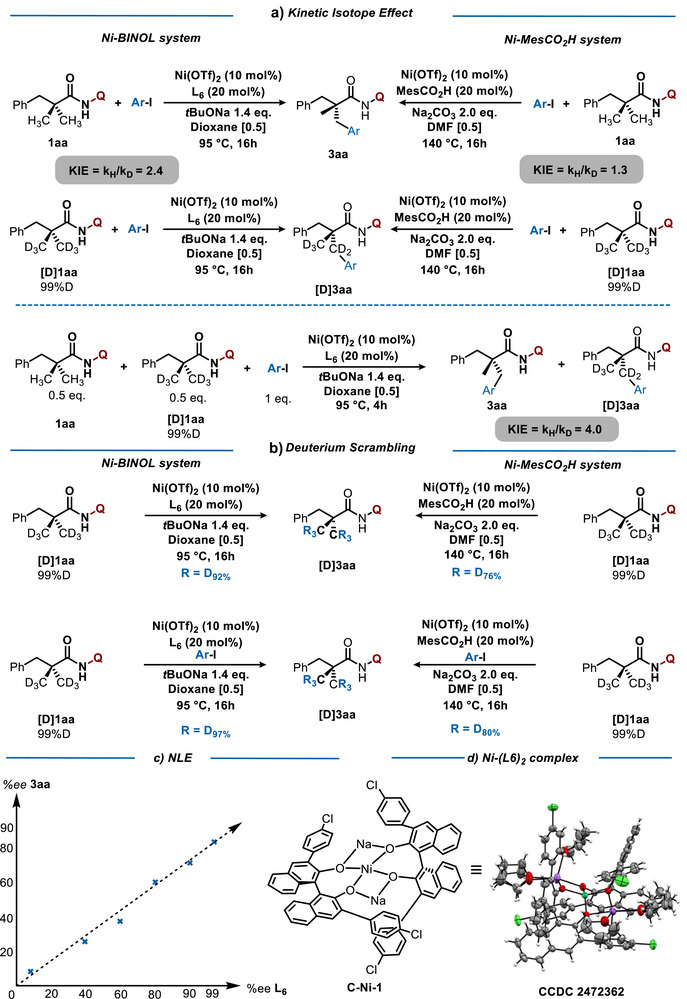
Deuterium‐Labeling Experiments and NLE.

Alternatively, we considered the possibility that the protonated BINOL ligand engages in a stabilizing π–π stacking interaction with the quinoline and/or anisole units, owing to its aromatic character. In this scenario, ligand exchange leads to the formation of INT4, followed by oxidative addition, with a significantly lower energy barrier of 23.4 kcal mol^−1^. These results highlight the dual role of the BINOL ligand: as a CMD ligand and as a stabilizing agent throughout the catalytic cycle. Moreover, the oxidative addition step has a lower barrier than the C─H activation (23.4 versus 26.7 kcal mol^−1^), supporting the experimental results that C─H activation is both irreversible and rate‐determining in our system (Figure 5a,b). Following oxidative addition, INT5 is formed, which undergoes facile reductive elimination with a barrier of only 17.1 kcal mol^−1^ to produce INT6. Subsequent ligand exchange with in situ generated sodium triflate leads to the release of sodium iodide and formation of INT7. From INT7, a new equivalent of the deprotonated aminoquinoline substrate enters the catalytic cycle, releasing the deprotonated product, giving rise to INT8 and completing the product turnover step. Finally, INT8 can regenerate INT1, thus completing the catalytic cycle. Overall, these computational results provide a rationale for the novel mechanism operating in our system, in which C─H activation is the rate‐determining step, at difference from previously reported examples, and underscore the excellent enantioselectivities observed experimentally.

**Figure 6:**
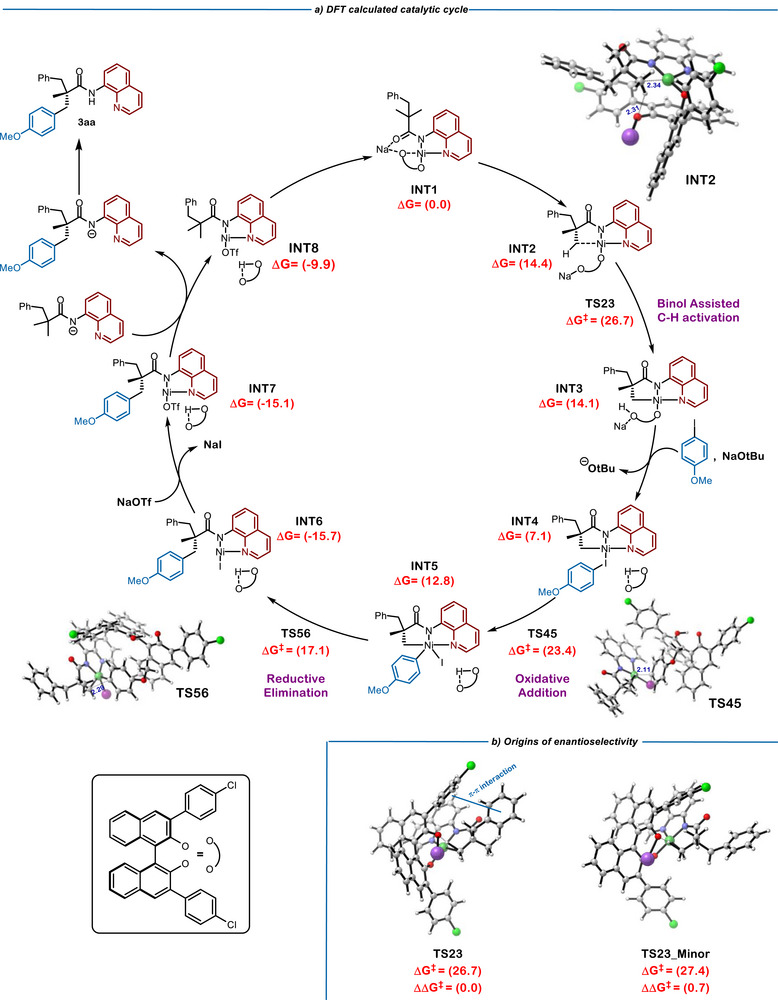
DFT calculations for the operating mechanism in the transformation and origins of enantioselectivity. Energies in kcal mol^−1^, bond lengths, labeled in blue, in Ångstroms.

To further investigate the origins of enantioselectivity in the transformation, we calculated the transition state leading to the minor enantiomer (Figure 6b). The computed energy barrier is of 27.4 kcal mol^−1^, which is 0.7 kcal mol^−1^ higher than that of the transition state leading to the experimentally observed major enantiomer. Analysis of Non‐Covalent Interactions (NCI) (Figure S1) reveals enhanced dispersion interactions, specifically a π–π stacking interaction between the benzyl group of the substrate and the *p*‐chlorophenyl moiety of the BINOL ligand for the major enantiomer. This interaction stabilizes the transition state thus explaining the observed enantioselectivity.

## Conclusion

In summary, we present an enantioselective C─H activation protocol enabled by a divalent nickel catalyst, offering an efficient approach to synthesizing all‐carbon quaternary chiral centers with high yields and excellent enantioselectivity. Central to this success is the utilization of the readily available BINOL‐derived ligand **L6**, which promotes enantioselective C─H nickelation under mild conditions. This study not only expands the scope of nickel‐catalyzed asymmetric C(sp^3^)─H activation but also lays the groundwork for future developments in 3d metal‐catalyzed asymmetric C─H functionalization strategies, fostering continued innovation and application in this field.

## Conflict of Interests

The authors declare no conflict of interest.

## Supporting information



Supporting Information

Supporting Information

## Data Availability

The data that support the findings of this study are available in the supplementary material of this article.
