# Supplementary material for: Intermolecular Enantioselective Nickel‐Catalyzed Arylation of Un‐Activated C(sp3)─H Bond
Source: Angew Chem Int Ed Engl. 2026 Jan 5;65(7):e23241. doi: 10.1002/anie.202523241 (PMC12887637; doi:10.1002/anie.202523241)
Supplement: Supplementary file 1 — Supporting Information [file ANIE-65-e23241-s001.pdf]

Intermolecular Enantioselective Nickel-Catalyzed Arylation of Unactivated C(sp^3)-H Bond

Erwan Brunard,[a] Fengjie Huang,[a] Àlex Díaz-Jiménez,[a] Sofiya Kostiukovska,[a] Joanna Wencel-Delord* [a]

a) Institute of Organic Chemistry, JMU Würzburg, Am Hubland, 97074, Würzburg, Germany

Contents

Intermolecular Enantioselective Nickel-Catalyzed Arylation of Unactivated C(sp^3)-H Bond 1

1. General Experimental Information 3
2. General Synthetic Procedures 3
   2.1 Ligand Synthesis 10
   2.2 Racemic Synthesis 13
3. Initial Optimization of Conditions 17
4. General Procedure for Arylation 20
5. Description of products and HPLC 21
6. Gram-scale Arylation and post-transformation 77
7. Experimental Mechanistic Studies and Control Experiments 82
   7.1 Synthesis of [D]3aa 82
   7.2 Intermolecular KIE experiment 83
   7.3 KIE for two parallel reactions 84
   7.4 Investigating the reversibility of the C–H activation step through H/D exchange 86
   7.5 Synthesis and Characterization of Ni(II) intermediates 91
8. X-Ray Crystallographic Data 92
9.DFT Calculations 94
10. NMR spectrum 128
11. References 181

1. General Experimental Information

All reagents were obtained from commercial suppliers unless otherwise stated.

Thin-layer chromatography was performed on silica gel 60 F254 on aluminium plates (Merck) and visualized under a UVP Mineralight UVLS-28 lamp (254 nm) and by staining with solutions of either ninhydrin, basic $KMnO_4$, phosphomolybdic acid, or acidic p-anisaldehyde in ethanol. Flash column chromatography was conducted on Merck silica gel 60 (40-63 µm) at medium pressure (300 mbar) or on puriFlash Spott II (biotage). Melting points were measured in capillary tubes on a Büchi B-540 apparatus and are uncorrected. Proton (^{1}H) and carbon (^{13}C) NMR spectra were recorded on Bruker spectrometers: Bruker Avance III HD 400 BBFO standard probe with z gradient and ATM, ^{15}N to ^{31}P + ^{19}F / ^{1}H, -150 to +150°C. Fluorine (^{19}F) NMR were recorded in the Bruker Avance II spectrometer. Carbon NMR (^{13}C) spectra were recorded at 101 MHz, using a broadband decoupled. NMR experiments were carried out in deuterated chloroform ($CDCl_3$), MeOH-d_4, or DMSO-d_6. Chemical shifts (d) are reported in parts per million (ppm). The following abbreviations are used for the proton spectra multiplicities: s: singulet, d: doublet, t: triplet, q: quadruplet, quint: quintuplet, sept: septet, app.: apparent, m: multiplet, br.: broad. Coupling constants (*J*) are reported In Hertz (Hz). Mass spectra were obtained using Brucker daltronics microOTOF focus electrospray ionization (ESI), or and Bruker daltonics ultraflextreme Matrix Assisted Laser Desorption Ionisation (MALDI) for the high resolution mass spectra (HRMS). The enantiomeric excesses were determined by HPLC analysis on a Daicel Chiralpak IA-5 column or a Daicel Chiralpak IC or a Daicel Chiralpak ID-5 column.

2. General Synthetic Procedures

All amides bearing an 8-aminoquinoline moiety were prepared by the reaction of the corresponding acid chlorides with 8-aminoquinoline.

General Procedure for the Preparation of the Starting Carboxylic Acid.

A solution of *n*-buthyllithium in hexane (1.6 M, 15 mL, 24 mmol) was added dropwise to a solution of diisopropylamine (2.4 g, 24 mmol) in 30 mL of THF at -78°C. The solution was stirred for 1h and a solution of methyl 2-phenylpropanoate (3.3 g, 20 mmol) in 10 ml of THF was added dropwise to the LDA solution. The solution was stirred for 1h at -78°C and 1-bromopentane (6.0 g, 40 mmol) was added dropwise to the solution. The solution was stirred for 1 h at -78°C and then 4h at r.t. The reaction mixture was quenched with water and the organic layer was separated. The aqueous layer was extracted with ether (2 x 15 mL). The combined organic layers were washed with brine (30 mL), dried over $MgSO_4$, filtered and evaporated in vacuo to give the desired methyl 2-methyl-2-phenylheptanoate (4.5 g, 96%). The ester was hydrolyzed in 30 mL of 4N NaOH aq. and MeOH at 100°C for 1day. After cooling the solution, the aqueous layer was extracted with DCM (2 x 15 mL). The aqueous layer was acidified with aqueous HCl and the aqueous layer was extracted with DCM (2 x 15 mL) and brine (30 mL), dried over $MgSO_4$, filtered and evaporated in vacuo to give the desired carboxylic acid (2.9 g, 70%).

General Procedure for the Preparation of Starting Amide.

To an oven-dried 100 mL three-necked flask, 2-methyl-2-phenylheptanoic acid (3.3 g, 15 mmol) was dissolved in thionyl chloride (60 mL) and refluxed overnight. Excess thionyl chloride was

removed by rotary evaporator to give a desired crude acid chloride and used for the subsequent steps without further purification. To another oven-dried 100 mL three-necked flask, 8-aminoquinoline (2.9 g, 20 mmol, 1.3 equiv.), Et_3N (4.1 mL, 30 mmol, 2 equiv.) and DCM (30 mL) were added. A solution of the acid chloride in DCM (10 mL) was added dropwise to the solution at 0°C, and the solution was then warmed to room temperature. After stirring overnight, the reaction system was quenched with sat. aq. $NaHCO_3$ (30 mL) and the organic layer was separated. The aqueous layer was extracted with DCM (2 x 15mL). The combined organic layers were washed with 1 M HCl aq. (30 mL) and brine (30 mL), dried over $MgSO_4$, filtered and evaporated in vacuo. The resulting crude amide was purified by column chromatography on silica gel (eluant: PE/EtOAc = 5/1) to afford the desired amide as a white solid (4.6 g, 90%).

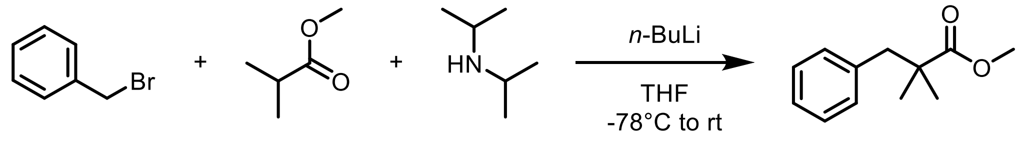

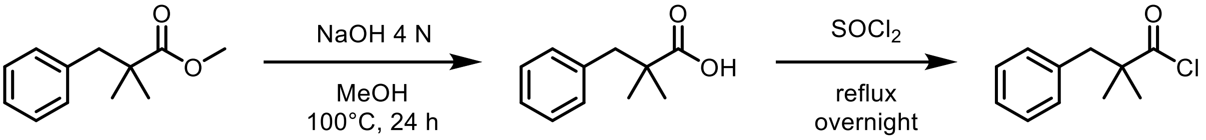

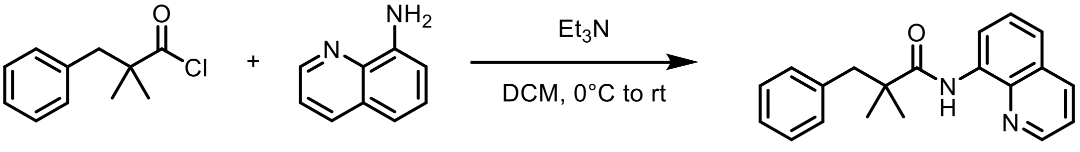

1aa

The crude product was dissolved in a minimal amount of ethanol at room temperature. Water was then added dropwise until the solution became turbid. The mixture was left undisturbed at 0°C overnight to allow for slow crystallization. The resulting crystals were collected by vacuum filtration, washed with cold pentane, and dried under reduced pressure to afford the pure compound **1aa** as a crystalline solid. Melting point = 77.5°C.

^{1}H NMR (400 MHz, $CDCl_3$) δ 10.17 (s, 1H), 8.84 (dd, *J* = 1.3, 7.6 Hz, 1H), 8.74 (dd, *J* = 1.6, 4.2 Hz, 1H), 8.13 (dd, *J* = 1.6, 8.3 Hz, 1H), 7.55 (t, *J* = 7.9 Hz, 1H), 7.49 (dd, *J* = 1.3 Hz, 8.3 Hz, 1H), 7.42 (dd, *J* = 4.2, 8.2 Hz, 1H), 7.09-7.23 (m, 5H), 3.05 (s, 2H), 1.41 (s, 6H).

^{13}C NMR (101 MHz, $CDCl_3$) δ 176.0, 148.1, 138.8, 137.9, 136.2, 134.5, 130.3 (2C), 128.0 (2C), 127.9, 127.4, 126.3, 121.5, 121.3, 116.2, 46.8, 44.9, 25.2 (2C).

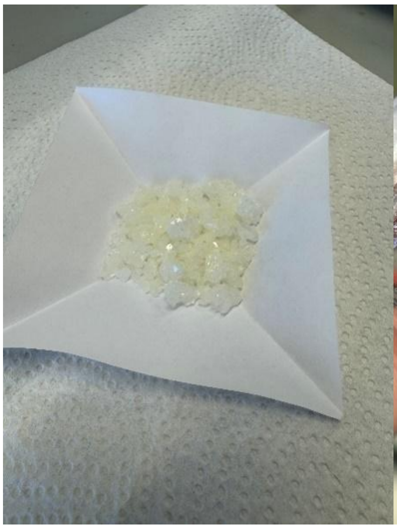
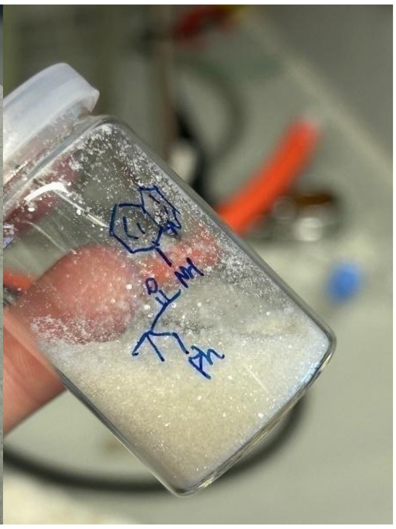

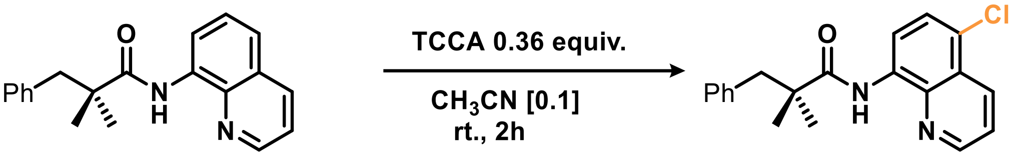

1aa 1ca

Compound **1ca** was afforded as white solid (237.0 mg, 71%yield) according to the reported procedure[1] using **1aa** (300.0 mg, 0.99 mmol) and trichlorocyanuric acid (cas 87-90-1, 83 mg, 0.36 mmol, 0.36 equiv.) as starting materials, purified by flash chromatography (PE/EA = 90:10).

^{1}H NMR (400 MHz, $CDCl_3$) δ 10.09 (s, 1H), 8.82 – 8.73 (m, 2H), 8.52 (dd, J = 8.5, 1.6 Hz, 1H), 7.60 (d, J = 8.4 Hz, 1H), 7.51 (dd, J = 8.5, 4.2 Hz, 1H), 7.22 – 7.11 (m, 5H), 3.03 (s, 2H), 1.42 (s, 6H).

^{13}C NMR (101 MHz, $CDCl_3$) δ 176.2, 148.8, 139.4, 137.9, 133.9, 133.4, 130.4 (2C), 128.1 (2C), 127.4, 126.5, 126.0, 124.2, 122.4, 116.3, 47.0, 45.1, 25.3.

These spectroscopic data correspond to reported data[2].

HRMS (ESI) [M + Na] calculated for $[C_{20}H_{19}ClN_2NaO_2]^+$ = 361.1078 found: 361.1073.

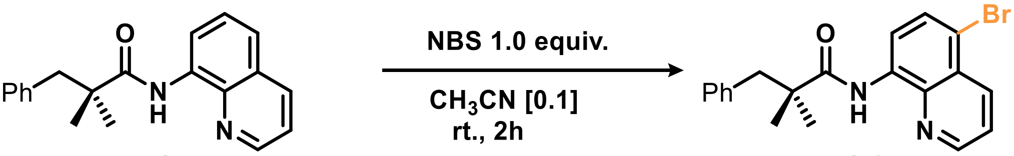

1aa 1cb

Compound **1cb** was afforded as white solid (290.0 mg, 77%yield) according to the reported procedure[1] using **1aa** (300.0 mg, 0.99 mmol) and NBS (cas 128-08-5, 180 mg, 0.99 mmol, 1.00 equiv.) as starting materials, purified by flash chromatography (PE/EA = 90:10).

---

[1] Motati, D. R., Uredi, D., Watkins, E. B. (2018) A general method for the metal-free, regioselective, remote C–H halogenation of 8-substituted quinolines. Chem. Sci., 9, 1782-1788. https://doi.org/10.1039/C7SC04107A.

[2] Aihara, Y., Chatani, N. (2016) Nickel-Catalyzed Reaction of C–H Bonds in Amides with I_2: ortho-Iodination via the Cleavage of C(sp^2)–H Bonds and Oxidative Cyclization to β-Lactams via the Cleavage of C(sp3)–H Bonds. ACS Catal. 6, 4323–4329. https://doi.org/10.1021/acscatal.6b00964.

^{1}H NMR (400 MHz, $CDCl_3$) δ 10.10 (s, 1H), 8.75 (dd, *J* = 4.3, 1.6 Hz, 1H), 8.73 (d, *J* = 8.4 Hz, 1H), 8.51 (dd, *J* = 8.6, 1.6 Hz, 1H), 7.81 (d, *J* = 8.4 Hz, 1H), 7.53 (dd, *J* = 8.5, 4.2 Hz, 1H), 7.21 – 7.11 (m, 5H), 3.03 (s, 2H), 1.41 (s, 6H).

^{13}C NMR (101 MHz, $CDCl_3$) δ 176.2, 148.8, 139.6, 137.9, 136.0, 134.5, 131.1, 130.4 (2C), 128.1 (2C), 127.3, 126.6, 122.7, 116.9, 114.2, 47.0, 45.1, 25.3 (2C).

HRMS (ESI) [M + Na] calculated for $[C_{20}H_{19}BrN_2NaO]^+$ = 405.0573 found: 405.0565.

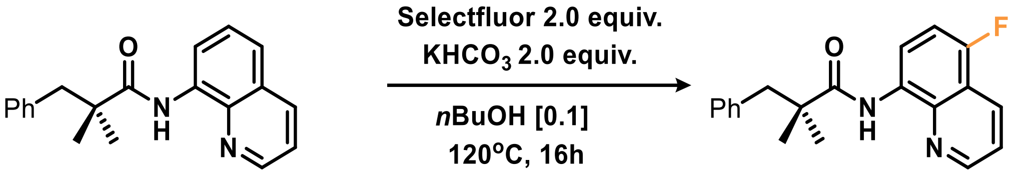

1aa 1cc

Compound **1cc** was afforded as white solid (190.6 mg, 60%yield) according to the reported procedure [3] using **1aa** (300.0 mg, 0.99 mmol) and 1-chloromethyl-4-fluoro-1,4-diazoniabicyclo[2.2.2]octane bis(tetrafluoroborate) (cas 140681-55-6, 700.0 mg, 1.97 mmol, 2.00 equiv.) as starting materials, purified by flash chromatography (PE/EA = 90:10).

^{1}H NMR (400 MHz, $CDCl_3$) δ 9.94 (s, 1H), 8.82 – 8.76 (m, 2H), 8.40 (dd, *J* = 8.4, 1.7 Hz, 1H), 7.47 (dd, *J* = 8.4, 4.3 Hz, 1H), 7.24 – 7.13 (m, 6H), 3.04 (s, 2H), 1.41 (s, 6H).

^{13}C NMR (101 MHz, $CDCl_3$) δ 176.0, 152.9 (d, *J* = 250.7 Hz), 149.1, 139.0 (d, *J* = 3.1 Hz), 138.0, 131.1 (d, *J* = 4.0 Hz), 130.4 (2C), 129.8 (d, *J* = 3.7 Hz), 128.1 (2C), 126.5, 121.7 (d, *J* = 2.7 Hz), 118.8 (d, *J* = 18.1 Hz), 115.8 (d, *J* = 7.6 Hz), 110.5 (d, *J* = 19.5 Hz), 47.0, 45.0, 25.3 (2C).

^{19}F NMR (376 MHz, $CDCl_3$) δ -129.5.

HRMS (ESI) [M + Na] calculated for $[C_{20}H_{19}FN_2NaO]^+$ = 345.1374 found: 345.1373.

[3] Chen, H., Li, P.H., Wang, M., Wang, L. (2018) Transition-Metal-Free Regioselective C–H Bond Fluorination of 8-Amidoquinolines with Selectfluor. Eur. J. Org. Chem. 18, 2091–2097. https://doi.org/10.1002/ejoc.201800389.

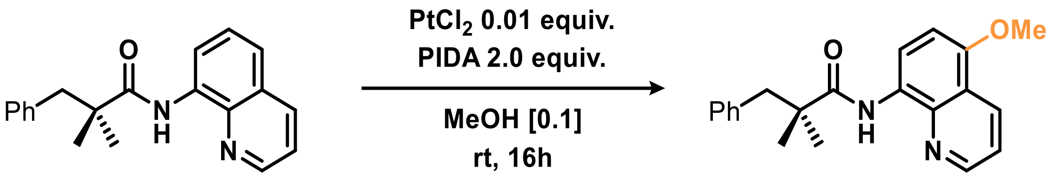

1aa 1ce

Compound **1ce** was afforded as white solid (125.2 mg, 38%yield) according to the reported procedure[4] using **1aa** (300.0 mg, 0.99 mmol) and Platinum(II) chloride as catalyst (2.62 mg, 0.009 mmol, 0.01 equiv.) and PIDA (634 mg, 1.97 mmol, 2.0 equiv.) as starting materials, purified by flash chromatography (PE/EA = 90:10).

^{1}H NMR (400 MHz, $CDCl_3$) δ 9.91 (s, 1H), 8.82 – 8.73 (m, 2H), 8.55 (dd, *J* = 8.4, 1.7 Hz, 1H), 7.41 (dd, *J* = 8.4, 4.2 Hz, 1H), 7.19 (d, *J* = 4.5 Hz, 4H), 7.19 – 7.09 (m, 1H), 6.85 (d, *J* = 8.6 Hz, 1H), 3.99 (s, 3H), 3.03 (s, 2H), 1.40 (s, 6H).

^{13}C NMR (101 MHz, $CDCl_3$) δ 175.7, 150.3, 148.8, 139.6, 138.2, 131.3, 130.4 (2C), 128.2, 128.1 (2C), 126.4, 120.8, 120.5, 116.5, 104.5, 55.9, 47.0, 44.9, 25.4 (2C).

HRMS (ESI) [M + Na] calculated for $[C_{21}H_{23}N_2O_2]^+$ = 335.1754 found: 335.1765.

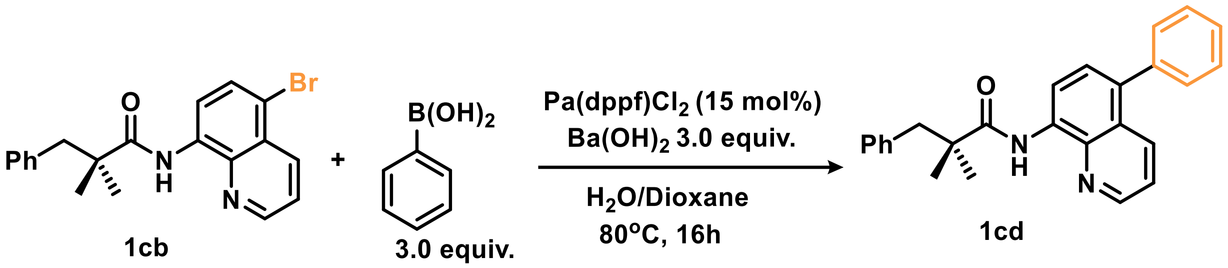

1cb 1cd

Compound **1cd** was afforded as white solid (140.0 mg, 75%yield) according to the modification following procedure[5] using **1cb** (190.0 mg, 0.50 mmol) with phenylboronic acid (CAS 98-80-6, 180 mg, 1.51 mmol) as starting materials, Pd(dppf)Cl_2 (55.0 mg, 0.08 mmol) as catalyst, $Ba(OH)_2$ (260.0 mg, 1.50 mmol) as base in 1,4-dioxane/H_2O (3:1), purified by flash chromatography (PE/EA = 90:10).

^{1}H NMR (400 MHz, $CDCl_3$) δ 10.28 (s, 1H), 8.90 (d, *J* = 8.0 Hz, 1H), 8.76 (dd, *J* = 4.2, 1.7 Hz, 1H), 8.28 (dd, *J* = 8.5, 1.6 Hz, 1H), 7.54 (d, *J* = 8.0 Hz, 1H), 7.51 – 7.42 (m, 5H), 7.39 (dd, *J* = 8.6, 4.2 Hz, 1H), 7.24 – 7.21 (m, 4H), 7.20 – 7.14 (m, 1H), 3.08 (s, 2H), 1.45 (s, 6H).

^{13}C NMR (101 MHz, $CDCl_3$) δ 176.2, 148.1, 139.4, 138.9, 138.1, 134.8, 134.2, 134.0, 130.4 (2C), 130.2 (2C), 128.6 (2C), 128.1 (2C), 128.1, 127.6, 126.5, 126.4, 121.6, 116.0, 47.0, 45.1, 25.4 (2C).

HRMS (ESI) [M + Na] calculated for $[C_{26}H_{24}N_2NaO]^+$ = 403.1781 found: 403.1782.

[4] G Wang, J Han, K Wang, H Li, G Duan, C Xia, F Li (2018) Methoxylation on the C5 and C6 positions of quinolines with methanol- *Catal. Commun*, *114*, 37-41. https://doi.org/10.1016/j.catcom.2018.05.021
[5] Li, Y., Zhu, L. Z., Cao, X., Au, C.-K., Qiu, R. H., Yin, S.-F. (2017) Metal–free C5-H Bromination of Quinolines for One-pot C−X (X=C, O, S) Bond Formations. Adv. Synth. Catal. 359, 2864 – 2873. https://doi.org/10.1002/adsc.201700391

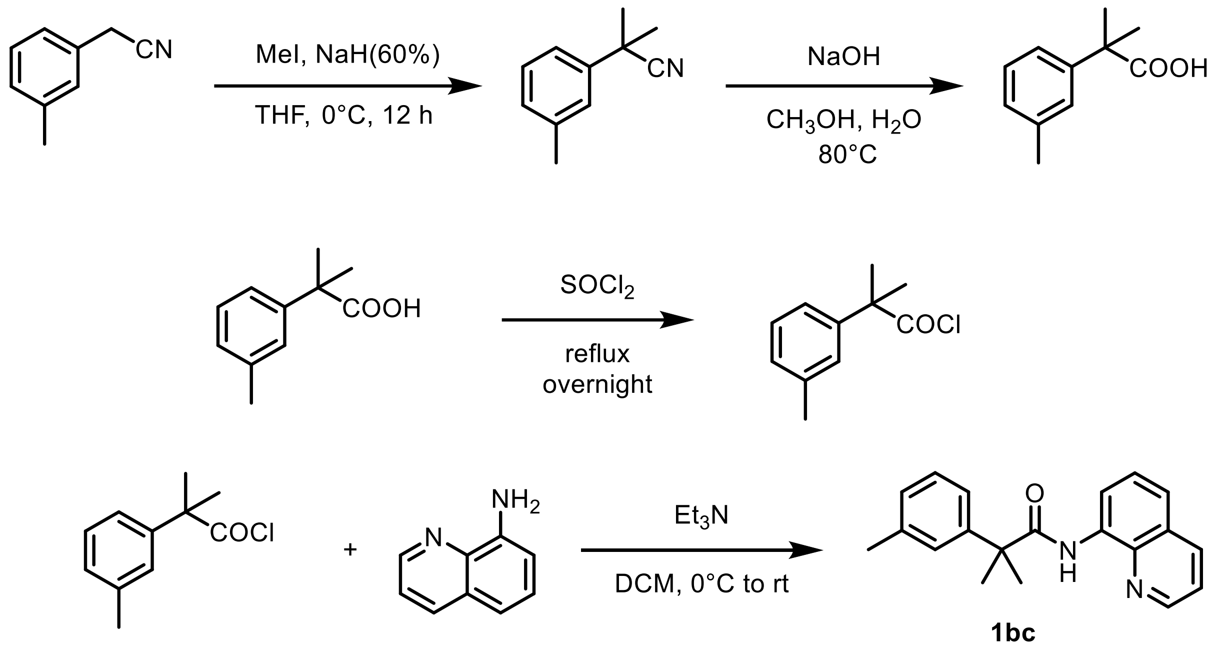

1bc

Compound **1bc** was afforded as white solid (1930.0 mg, 66% yield) according to literature[6] procedure using commercially available 2-(*m*-Tolyl)acetonitrile.

^{1}H NMR (400 MHz, $CDCl_3$) δ 9.90 (s, 1H), 8.77 (dd, *J* = 7.6, 1.4 Hz, 1H), 8.62 (dd, *J* = 4.2, 1.7 Hz, 1H), 8.08 (dd, *J* = 8.3, 1.7 Hz, 1H), 7.54 – 7.49 (m, 1H), 7.46 – 7.42 (m, 1H), 7.38 – 7.33 (m, 3H), 7.29 (dd, *J* = 8.5, 7.3 Hz, 1H), 7.14 – 7.10 (m, 1H), 2.38 (s, 3H), 1.78 (s, 6H).

^{13}C NMR (101 MHz, $CDCl_3$) δ 176.0, 148.2, 144.9, 138.8, 138.4, 136.2, 134.9, 128.8, 127.9, 127.9, 127.5, 127.3, 123.4, 121.5, 121.3, 116.1, 48.4, 27.1 (2C), 21.8.

HRMS (ESI) [M + Na] calculated for $[C_{20}H_{20}N_2NaO]^+$ = 327.1468 found: 327.1471.

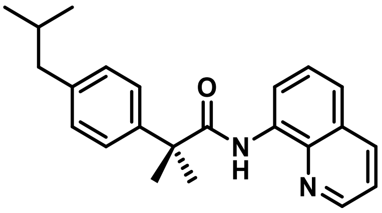

1bi

Compound **1bi** was afforded as white solid (1930.0 mg, 66% yield) according to General Procedure for the Preparation of Starting Amide, purified by flash chromatography (PE/EA = 90:10).

^{1}H NMR (400 MHz, $CDCl_3$) δ 9.82 (s, 1H), 8.77 (dd, *J* = 7.6, 1.4 Hz, 1H), 8.54 (dd, *J* = 4.3, 1.7 Hz, 1H), 8.07 (dd, *J* = 8.3, 1.7 Hz, 1H), 7.53 – 7.47 (m, 1H), 7.48 – 7.42 (m, 3H), 7.34 (dd, *J* = 8.3, 4.2 Hz, 1H), 7.22 – 7.15 (m, 2H), 2.49 (d, *J* = 7.2 Hz, 2H), 1.89 (dp, *J* = 13.7, 6.8 Hz, 1H), 1.77 (s, 6H), 0.92 (d, *J* = 6.6 Hz, 6H).

---

[6] Srinivas. D., Satyanarayana. G. (2018) Palladium-Catalyzed Distal *m*-C–H Functionalization of Arylacetic Acid Derivatives. Org. Lett. 23, 19, 7353–7358. https://doi.org/10.1021/acs.orglett.1c02460

^{13}C NMR (101 MHz, $CDCl_3$) δ 176.4, 148.2, 142.1, 140.6, 138.8, 136.2, 134.9, 129.6 (2C), 128.0, 127.5, 126.4 (2C), 121.5, 121.3, 116.1, 48.2, 45.1, 30.4, 27.2, 22.5.

HRMS (ESI) [M + Na] calculated for $[C_{23}H_{26}N_2NaO]^+$ = 369.1937 found: 369.1932.

Melting point = 100.5°C.

2.1 Ligand Synthesis

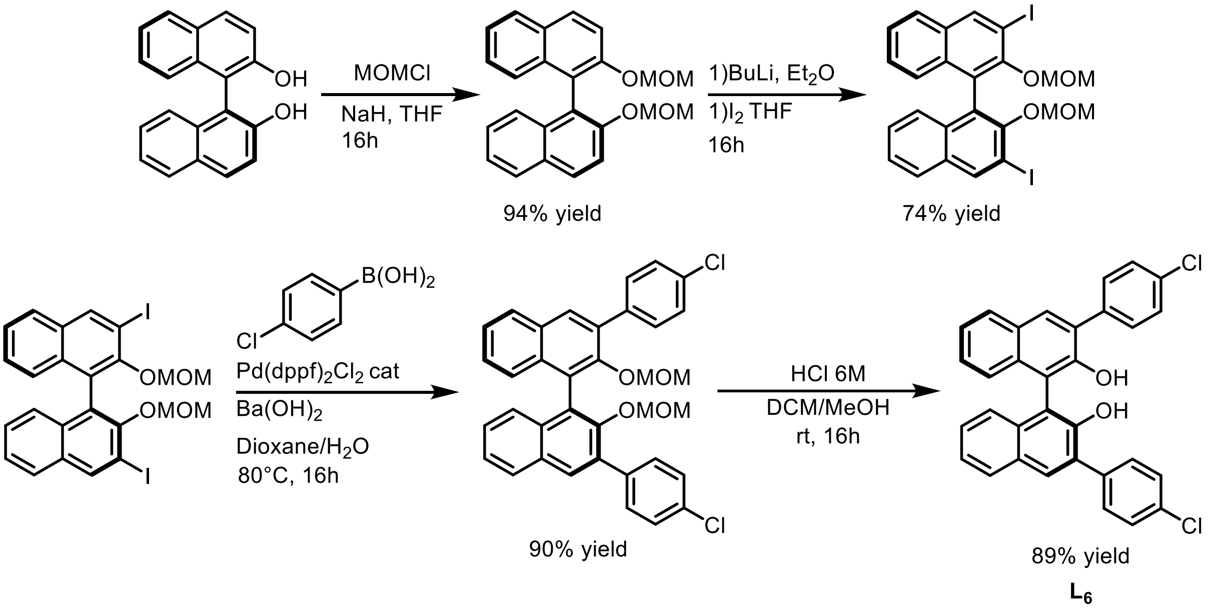

Ligand (*S*)-**L6** was synthesized following literature procedure using commercially available (*S*)-Binol (99% *ee*).[7] The deprotection step was carried out according to the reported method to minimize the risk of racemization.[8] MOM-protected BINOL (1.5 g, 2.5 mmol) was dissolved in 50 mL of a 1:1 mixture of DCM and MeOH in an oven-dried flask equipped with a magnetic stir bar. To this solution, 10 mL of 6 M HCl was added slowly dropwise. The reaction mixture was stirred at room temperature overnight, during which time TLC analysis confirmed complete consumption of the starting material and the formation of a more polar product. Saturated aqueous sodium bicarbonate (~40 mL) was then added to quench the reaction, and the mixture was extracted with DCM (3×). The combined organic layers were washed with brine, dried over Na_2SO_4, filtered, and concentrated. The crude product was purified by flash column chromatography (10% diethyl ether in pentane) to afford BINOL $\mathbf{L_6}$ as a white solid (1.1 g, 89% yield), whose NMR spectra matched the reported data.[6]

The product was dissolved in a minimal amount of dichloromethane at room temperature. Pentane was then added until the solution became turbid. The mixture was left undisturbed at 0°C overnight to allow for slow crystallization. The resulting crystals were collected by vacuum filtration, washed with cold pentane, and dried under reduced pressure to afford the pure compound as a crystalline solid in quantitative yield.

---

[7] Romanov-Michailidis, F., Guénée, L., and Alexakis, A. (2013). Enantioselective Organocatalytic Fluorination-Induced Wagner–Meerwein Rearrangement. Angew. Chem. Int. Ed. 52, 9266–9270. https://doi.org/10.1002/anie.201303527.

[8] Nistanaki, S.K., Williams, C.G., Wigman, B., Wong, J.J., Haas, B.C., Popov, S., Werth, J., Sigman, M.S., Houk, K.N., and Nelson, H.M. (2022). Catalytic asymmetric C–H insertion reactions of vinyl carbocations. Science 378, 1085–1091. https://doi.org/10.1126/science.ade5320.

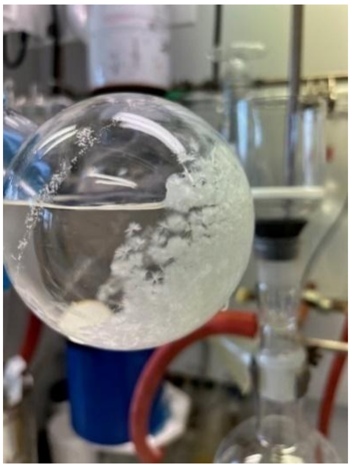

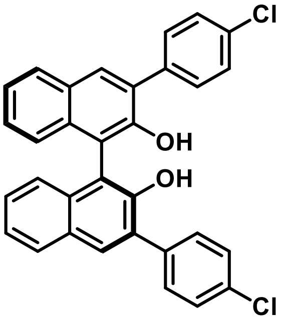

^{1}H NMR (400 MHz, $CDCl_3$) δ 8.02 (s, 2H), 7.93 (ddd, *J* = 8.6, 1.3, 0.5 Hz, 2H), 7.69 (d, *J* = 8.6 Hz, 4H), 7.46 (d, *J* = 8.6 Hz, 4H), 7.42 (ddd, *J* = 8.2, 6.8, 1.3 Hz, 2H), 7.34 (ddd, *J* = 8.3, 6.8, 1.4 Hz, 2H), 7.21 (ddt, *J* = 8.4, 1.4, 0.7 Hz, 2H), 5.30 (d, *J* = 0.7 Hz, 2H).

^{13}C NMR (101 MHz, $CDCl_3$) δ 150.1 (2C), 135.9 (2C), 133.8 (2C), 132.9 (2C), 131.6 (2C), 131.0 (4C), 129.5 (2C), 129.5 (2C), 128.6 (2C), 128.6 (4C), 127.7 (2C), 124.6 (2C), 124.2 (2C), 112.0 (2C).

HRMS (ESI) [M + Na] calculated for $[C_{32}H_{20}Cl_2NaO_2]^+$ = 529.0732 found: 529.0717.

$[\alpha]_D^{20}$= -35.0 (c = 0.58, $CHCl_3$).

Melting point = 177.7°C.

All the Binol's ligands used in the optimization were either purchased from BLD and Sigma or synthesized using the same synthetic pathway.

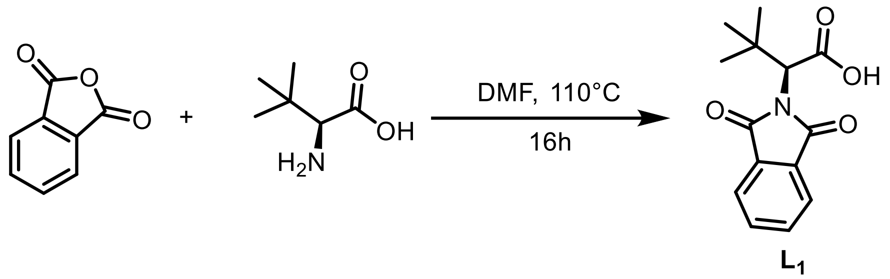

A flame-dried 250 mL round-bottom flask equipped with a football-shaped magnetic stirring bar and a reflux condenser was charged with the anhydride (17.0 mmol, 1 equiv) and tert-leucine (17.0 mmol, 1 equiv). Dry DMF (100 mL) was added under an argon atmosphere, and the resulting colorless solution was heated at 110°C for 16–24 hours under argon. After cooling to room temperature, the reaction mixture was diluted with EtOAc (400 mL) and washed seven times with brine (7 × 150 mL). The organic layer was dried over $MgSO_4$, filtered, and evaporated to dryness under reduced pressure. The resulting crude solid was purified by silica gel column chromatography, eluting with 2–10% MeOH in CH_2Cl_2, depending on the polarity of the product, to afford the pure carboxylic acid ligand in 57–92% yield. All analytical data (e.g., NMR, MS) were consistent with literature values for the expected compound.[9]

^{1}H NMR (400 MHz, $CDCl_3$) δ 7.95-7.85 (2H, m), 7.78-7.73 (2H, m), 4.74 (1H, s, CH), 1.19 (9H, s).

^{13}C NMR (101 MHz, $CDCl_3$) δ 173.6 (2C), 167.8 (2C), 134.1 (2C), 131.5 (2C), 123.5 (2C), 59.8, 35.7, 28.0 (3C).

L_2 was synthesized from following a previously reported procedure, and the characterization data are consistent with the literature.[10]

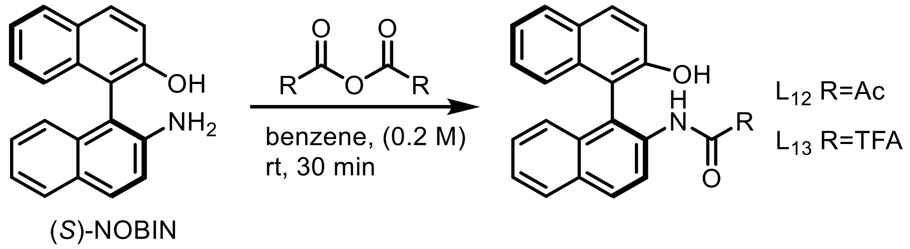

[9]Lindsay, V.N.G., and Charette, A.B. (2012). Design and Synthesis of Chiral Heteroleptic Rhodium(II) Carboxylate Catalysts: Experimental Investigation of Halogen Bond Rigidification Effects in Asymmetric Cyclopropanation. ACS Catal. *2*, 1221–1225. https://doi.org/10.1021/cs300214v.
[10] Yao, Q.-J., Chen, J.-H., Song, H., Huang, F.-R., and Shi, B.-F. (2022). Cobalt/Salox-Catalyzed Enantioselective C−H Functionalization of Arylphosphinamides. Angewandte Chemie International Edition *61*, e202202892. https://doi.org/10.1002/anie.202202892.

L_{12} and L_{13} were synthesized from NOBIN[11] following a previously reported procedure, and their characterization data are consistent with the literature.[12]

2.2 Racemic Synthesis

General Procedure for the Preparation of the Racemic products using Chatani's[13] condition

To an oven-dried 5 mL Schlenk flask, containing the substrate (0.3 mmol, 1.0 eq.), aryl iodide (0.9 mmol, 3.0 eq.), $Ni(OTf)_2$ (9.9 mg, 0.03 mmol, 0.1 eq.), MesCOOH (9.8 mg, 0.06 mmol), Na_2CO_3 (64 mg, 0.6 mmol) and DMF (0.6 mL) were added. The mixture was stirred for 24 h at 140°C followed by cooling. 30 mL of water was added to the reaction mixture and the aqueous layer was extracted with ethyl acetate (3 x 10 mL). The combined organic layers were washed with brine (20 mL), dried over $MgSO_4$, filtered and evaporated in vacuo. The residue was purified by column chromatography on silica gel (eluent: PE/EtOAc=10/1 or PE/DCM=30/70) to afford the following desired arylated product.

[11] Chang, X., Zhang, Q., and Guo, C. (2019). Switchable Smiles Rearrangement for Enantioselective O-Aryl Amination. Org. Lett. 21, 4915–4918. https://doi.org/10.1021/acs.orglett.9b01848.

[12] González, J.M., Vidal, X., Ortuño, M.A., Mascareñas, J.L., and Gulías, M. (2022). Chiral Ligands Based on Binaphthyl Scaffolds for Pd-Catalyzed Enantioselective C–H Activation/Cycloaddition Reactions. J. Am. Chem. Soc. 144, 21437–21442. https://doi.org/10.1021/jacs.2c09479.

[13] Aihara, Y., Chatani, N. (2014) Nickel-Catalyzed Direct Arylation of C(Sp^3)–H Bonds in Aliphatic Amides via Bidentate-Chelation Assistance. J. Am. Chem. Soc. 136, 898–901. https://doi.org/10.1021/ja411715v.

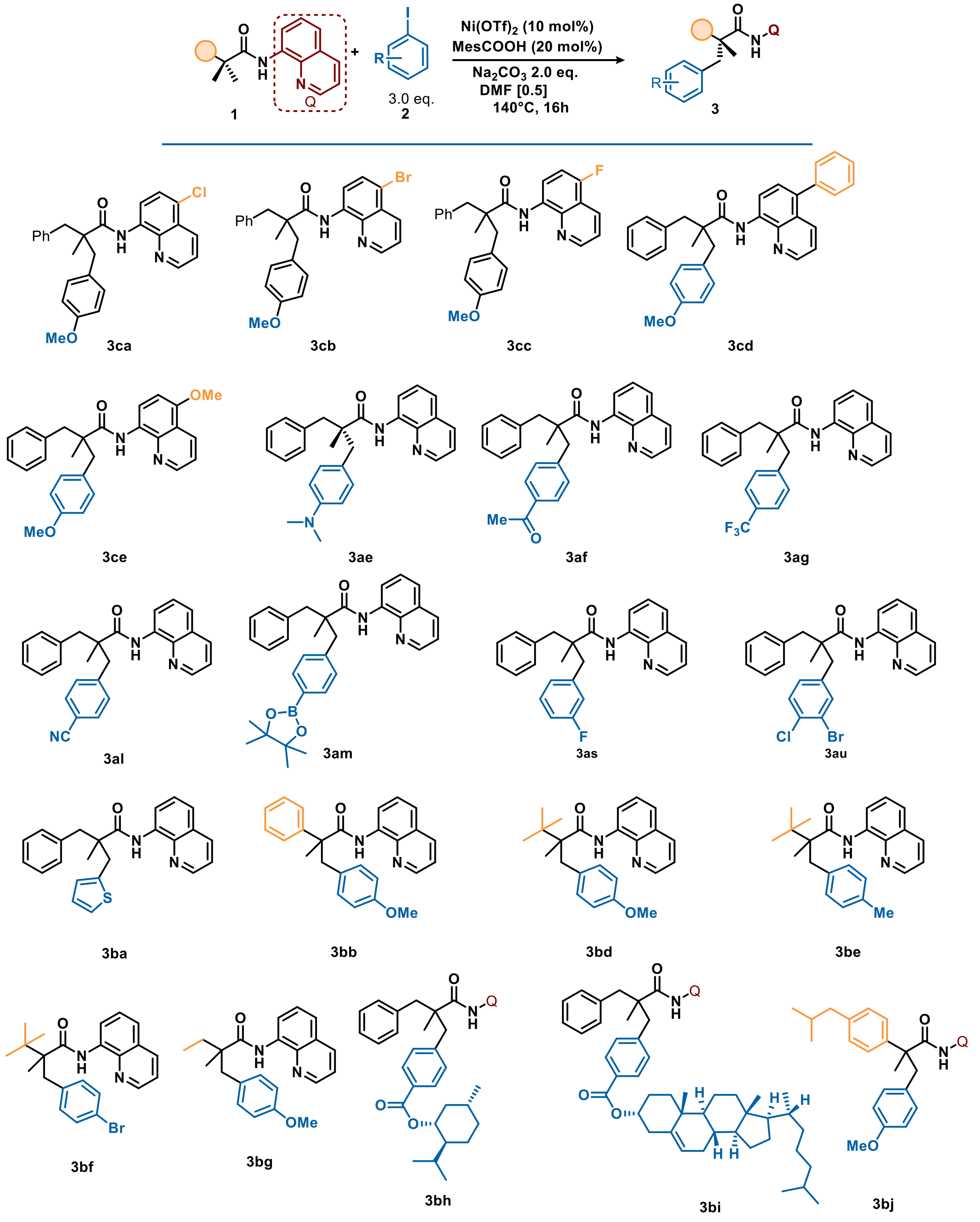

General Procedure for the Preparation of the Racemic products using You's[14] condition

An oven-dried Schlenk tube with a magnetic stir bar was charged with aliphatic amide 1 (0.2 mmol, 1 equiv), aryl iodide 2 (0.4 mmol or 0.6 mmol), $Ni(OTf)_2$ (7.2 mg, 0.02 mmol), PPh_3 (10.4

[14] Li, M.L., Dong, J.X., Huang, X. L., Li, K.Z., Wu, Q., Song, F.J., You, J.S. (2014) Nickel-catalyzed chelation-assisted direct arylation of unactivated C(sp^3)–H bonds with aryl halides. Chem. Commun., 50, 3944-3946. https://doi.org/10.1039/C4CC00716F

mg, 0.04 mmol), Na_2CO_3 (42 mg, 0.4 mmol), and PivOH (4.2 mg, 0.04 mmol). The tube was then taken to a Schlenk line and DMSO (50 μL, 0.7 mmol) and 1,4-dioxane (1 mL) were added under an argon atmosphere. The tube was sealed with a teflon-coated cap and the resulting mixture was stirred at 160°C for 36 h. After being cooled to ambient temperature, the solution was diluted with 20 mL of EtOAc, filtered through a celite pad, and washed with 10-20 mL of EtOAc. The combined organic layers were washed with brine (20 mL), dried over $MgSO_4$, filtered and evaporated in vacuo. The residue was purified by column chromatography on silica gel (eluent: PE/EtOAc=10/1 or PE/DCM=30/70) to afford the following desired arylated product.

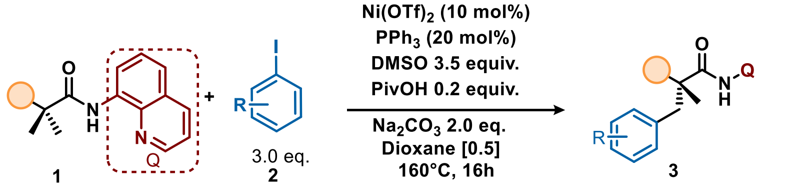

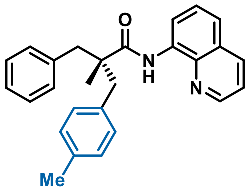
3ab

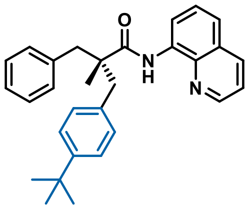
3ac

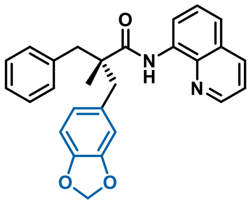
3ad

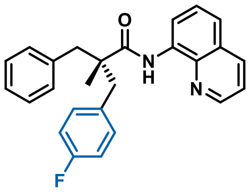
3ah

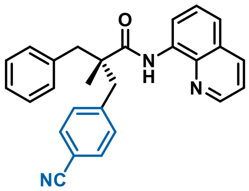
3al

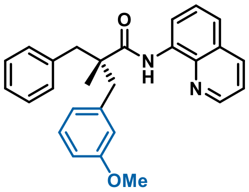
3an

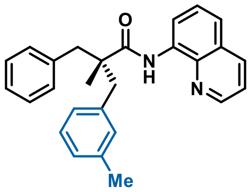
3ao

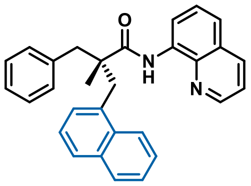
3ap

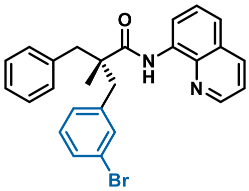
3aq

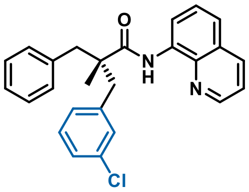
3ar

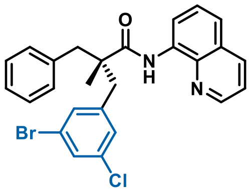
3at

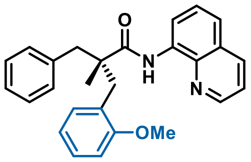
3aw

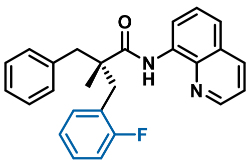
3ax

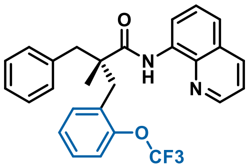
3ay

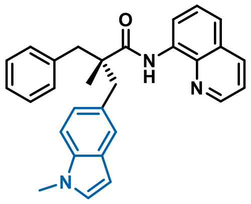
3az

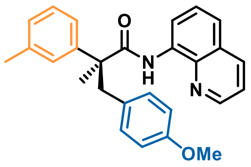
3bc

3. Initial Optimization of Conditions

Supplementary Table 1. Ligand screening

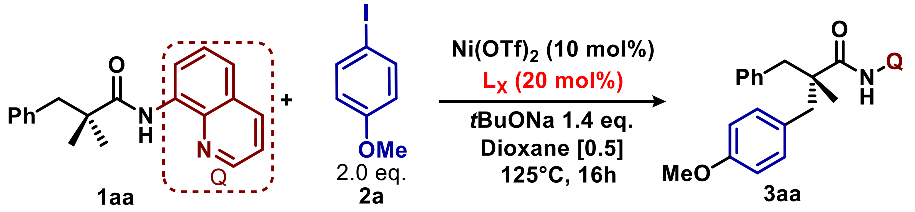

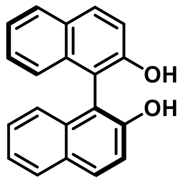

50% yield
57:43 *er*

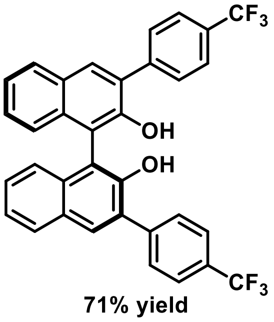

71% yield
80:20 *er*

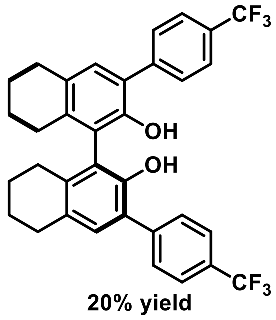

20% yield
76.5:23.5 *er*

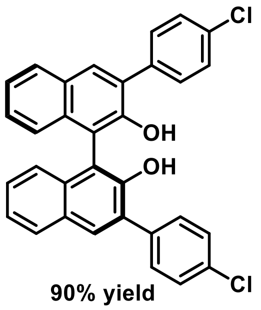

90% yield
75:25 *er*

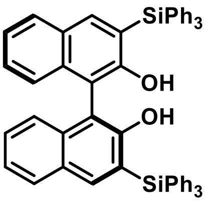

89% yield
72.5:27.5 *er*

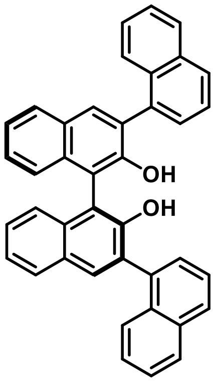

89% yield
75:25 *er*

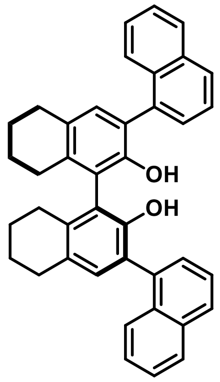

95% yield
77.5:22.5 *er*

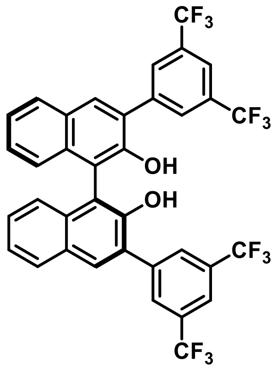

98%yield
69:31 *er*

Supplementary Table 2. solvent screening

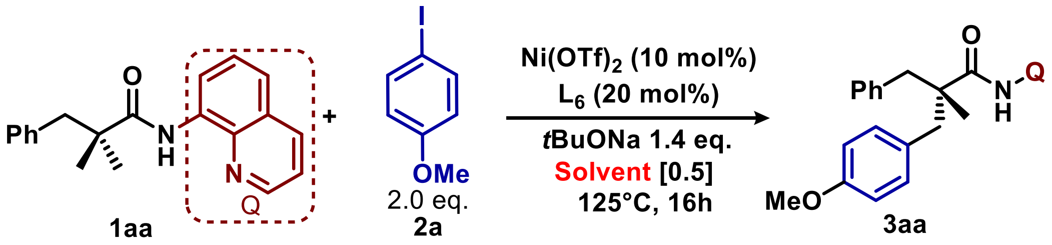

Solvent	Yield (%)	*er*
Dioxane	80	90:10
Toluene	30	69:31
Cumene	54	78:22
o-xylene	28	57:43
m-xylene	33	58:42
p-xylene	13	54:46
Chlorobenzen	21	54:46
MeTHF	65	85:15
THF	70	86:14
CPME	67	84:16
tert-amyl alcohol	73	80:20
*tert*butanol	51	86:14
Acetone	0	N.R
DCE	18	78:22
DMSO	0	N.R
MeCN	0	N.R

Supplementary Table 3. Equivalents of Base

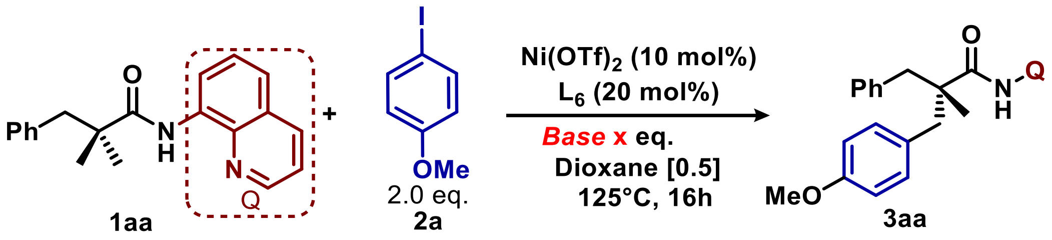

Base	equiv.	Yield (%)	*er*
*t*BuONa	1.5	79	91:9
*t*BuOLi	1.5	23	82.5:17.5
*t*BuOK	1.5	0	N.R
*t*BuONa	2.0	96	82:18
*t*BuONa	1.4	82	92:8

Supplementary Table 4. Ni screening

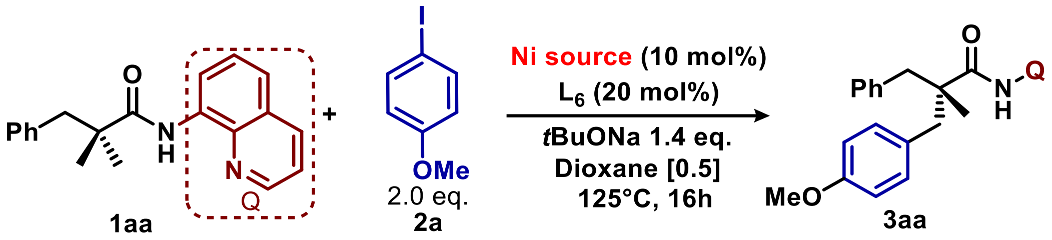

Ni source	Yield (%)	*er*
$Ni(OTf)_2$	82	92:8
$Ni(Cl)_2$	70	90:10
$Ni(I)_2$	90	88:12
$NiCl_2$(dppp)	67	89.5:10.5
$NiCl_2$(dppe)	67	89:11
No catalyst	0	-

Supplementary Table 5 No ligand screening

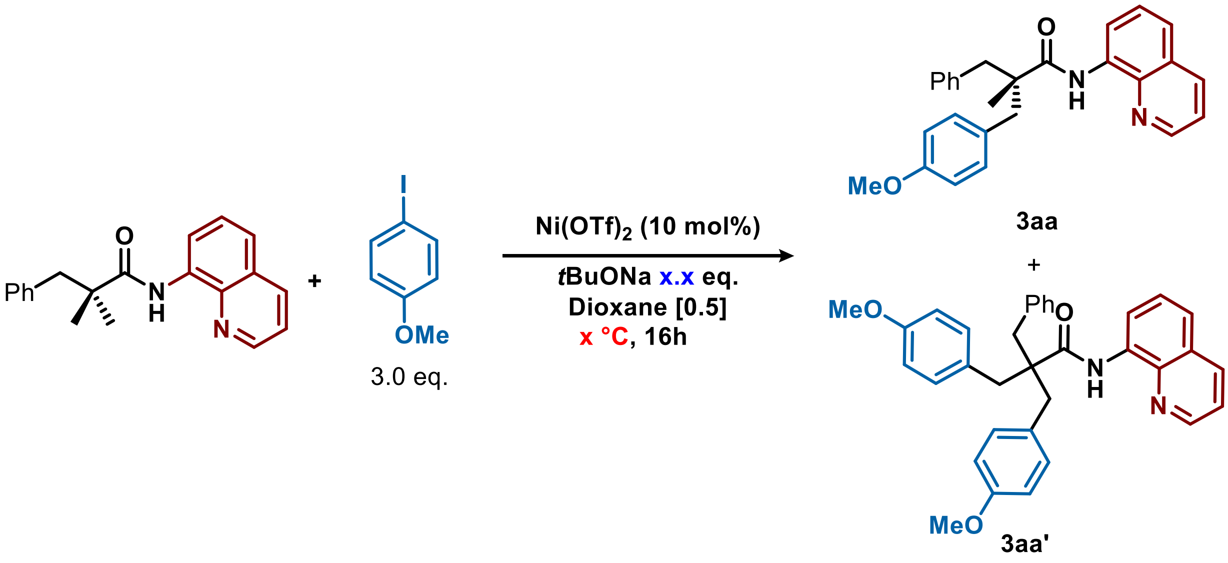

eq. base	temp	3aa %	3aa' %
0.5	120	5	-
1.5	120	30	5
2.0	120	40	18
2.5	120	49	22
2.0	110	32	14
2.0	100	15	5
2.0	90	-	-

4. General Procedure for Arylation

To an oven-dried 5 mL Schlenk flask, containing the substrate (0.3 mmol, 1.0 eq.), aryl iodide (0.9 mmol, 3.0 eq.), $Ni(OTf)_2$ (9.9 mg, 0.03 mmol, 0.1 eq.), $\mathbf{L_6}$ (33 mg, 0.06 mmol, 0.2 eq.), *t*BuONa (40.5 mg, 0.42 mmol, 1.4 eq.) and dry freshly distilled 1,4-dioxane (0.6 mL) were added under Ar atmosphere inside a glovebox. The mixture was stirred for 16 h at 95°C outside of the glovebox followed by cooling. 10 mL of water was added to the reaction mixture and the aqueous layer was extracted with ethyl acetate (3 x 10 mL). The combined organic phase was washed with brine (50 mL), dried over $MgSO_4$, filtered and evaporated in vacuo. The residue was purified by column chromatography on silica gel (eluent: PE/EtOAc=10/1 or PE/DCM=30/70) to afford the desired arylated product.

5. Description of products and HPLC

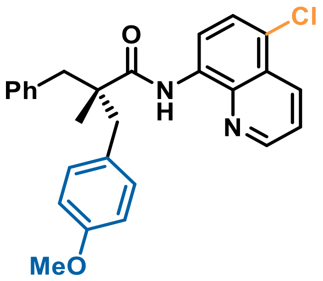

3ca

The product **3ca** was synthesized according to the general procedure using **1ca** (101.6 mg) as the substrate. The purification was performed by column chromatography (PE/EA = 90:10) yielding the title compound as a colorless oil (84.4 mg, 64% yield). The enantiomeric excess was determined by HPLC analysis on a Daicel Chiralpak ID-5 column: 90:10 Hexane/IPA, flow rate 0.5mL/min, λ= 250.0 nm: T_RMajor = 43.96 min, T_RMinor = 42.02 min. er = 91.5: 8.5.

^{1}H NMR (400 MHz, $CDCl_3$) δ 9.81 (s, 1H), 8.81 (d, *J* = 8.4 Hz, 1H), 8.67 – 8.63 (m, 1H), 8.54 – 8.48 (m, 1H), 7.61 (d, *J* = 8.4 Hz, 1H), 7.52 – 7.43 (m, 1H), 7.21 – 7.11 (m, 5H), 7.11 – 7.07 (m, 2H), 6.73 – 6.66 (m, 2H), 3.66 (s, 3H), 3.46 (d, *J* = 13.2 Hz, 1H), 3.41 (d, *J* = 13.4 Hz, 1H), 2.76 (d, *J* = 13.3 Hz, 1H), 2.72 (d, J = 13.5 Hz, 1H), 1.29 (s, 3H).

^{13}C NMR (101 MHz, $CDCl_3$) δ 174.8, 158.3, 148.6, 139.3, 137.8, 133.6, 133.3, 131.4 (2C), 130.4 (2C), 129.7, 128.1 (2C), 127.3, 126.5, 125.9, 124.3, 122.3, 116.4, 113.5 (2C), 55.2, 50.2, 46.6, 46.0, 19.7.

HRMS (ESI) [M + Na] calculated for $[C_{27}H_{25}ClN_2NaO_2]^+$ = 467.1497 found: 467.1489.

$[\alpha]_D^{20}$= -5.9 (c = 0.75, $CHCl_3$).

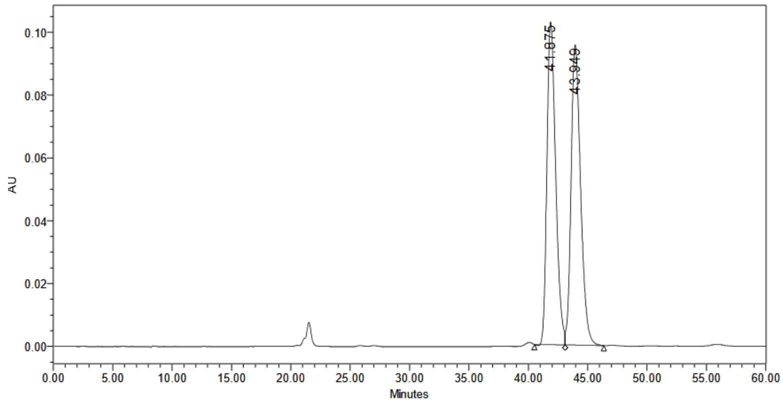

	RT	Area	% Area	Height
1	41.875	5269336	49.74	102790
2	43.949	5324153	50.26	95554

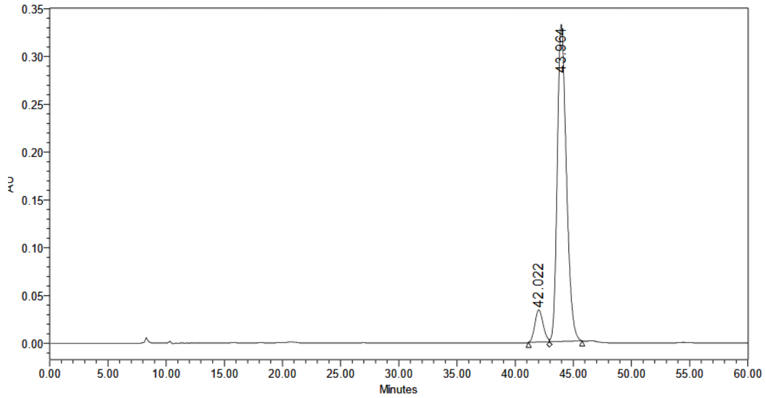

	RT	Area	% Area	Height
1	42.022	1662349	8.41	33581
2	43.964	18092567	91.59	331679

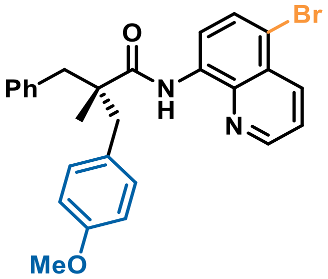

3cb

The product **3cb** was synthesized according to the general procedure using **1cb** (114.9 mg) as the substrate. The purification was performed by column chromatography (PE/DCM = 90:10) yielding the title compound as a colorless oil (101.3 mg, 69% yield). The enantiomeric excess was determined by HPLC analysis on a Daicel Chiralpak ID-5 column: 90:10 Hexane/IPA, flow rate 0.5mL/min, λ= 250.0 nm: T_RMajor = 49.51 min, T_RMinor = 47.07 min. er = 90.0: 10.0.

^{1}H NMR (400 MHz, $CDCl_3$) δ 9.83 (s, 1H), 8.76 (d, *J* = 8.4 Hz, 1H), 8.62 (dd, *J* = 4.2, 1.6 Hz, 1H), 8.46 (dd, *J* = 8.5, 1.6 Hz, 1H), 7.81 (d, *J* = 8.4 Hz, 1H), 7.47 (dd, *J* = 8.5, 4.2 Hz, 1H), 7.21 – 7.11 (m, 5H), 7.11 – 7.08 (m, 2H), 6.69 (d, *J* = 8.7 Hz, 2H), 3.66 (s, 3H), 3.46 (d, *J* = 13.2 Hz, 1H), 3.40 (d, *J* = 13.4 Hz, 1H), 2.76 (d, *J* = 13.2 Hz, 1H), 2.72 (d, *J* = 13.3 Hz, 1H), 1.29 (s, 3H).

^{13}C NMR (101 MHz, $CDCl_3$) δ 174.9, 158.3, 148.7, 139.5, 137.7, 135.8, 134.2, 131.4 (2C), 131.0, 130.4 (2C), 129.6, 128.2 (2C), 127.2, 126.5, 122.6, 116.9, 114.2, 113.5 (2C), 55.2, 50.3, 46.6, 46.0, 19.7.

HRMS (ESI) [M + Na] calculated for $[C_{27}H_{26}BrN_2NaO_2]^+$ = 511.0992 found: 511.0991.

$[\alpha]_D^{20}$= -3.4 (c = 0.75, $CHCl_3$).

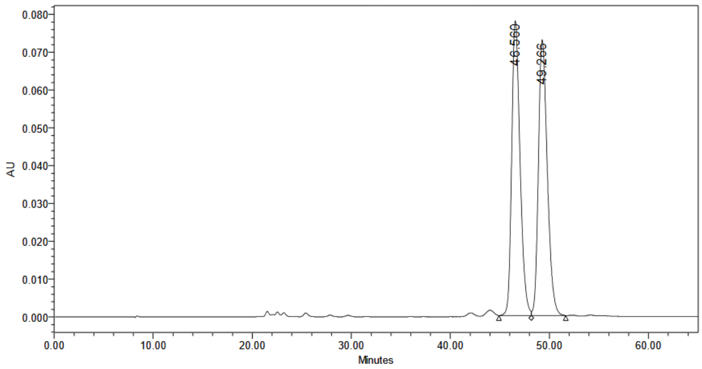

	RT	Area	% Area	Height
1	46.560	4538918	49.98	78001
2	49.266	4543055	50.02	72950

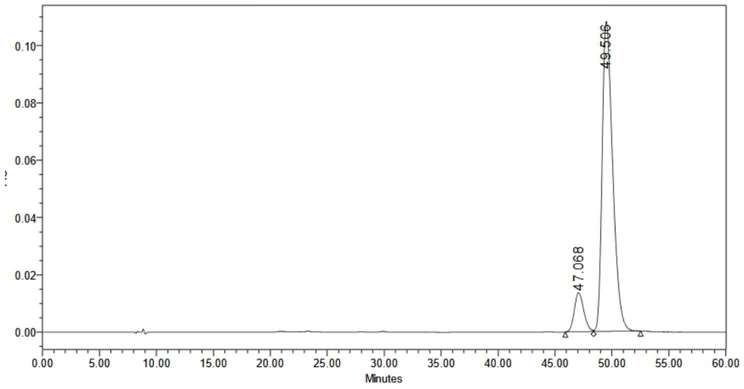

	RT	Area	% Area	Height
1	47.068	786521	10.14	13650
2	49.506	6970039	89.86	108248

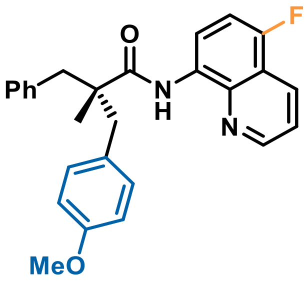

3cc

The product **3cc** was synthesized according to the general procedure using **1cc** (96,7 mg) as the substrate. The purification was performed by column chromatography (PE/DCM = 90:10) yielding the title compound as a colorless oil (85.6 mg, 65% yield). The enantiomeric excess was determined by HPLC analysis on a Daicel Chiralpak IC-5 column: 90:10 Hexane/IPA, flow rate 0.5mL/min, λ= 250.0 nm: T_RMajor = 24.66 min, T_RMinor = 26.78 min. er = 85.0:15.0.

^{1}H NMR (400 MHz, $CDCl_3$) δ 9.66 (s, 1H), 8.81 (dd, *J* = 8.6, 5.5 Hz, 1H), 8.66 (dd, *J* = 4.3, 1.7 Hz, 1H), 8.37 (dd, *J* = 8.4, 1.7 Hz, 1H), 7.43 (dd, *J* = 8.4, 4.2 Hz, 1H), 7.25 – 7.12 (m, 6H), 7.11 – 7.07 (m, 2H), 6.71 – 6.66 (m, 2H), 3.67 (s, 3H), 3.46 (d, *J* = 13.2 Hz, 1H), 3.40 (d, *J* = 13.3 Hz, 1H), 2.75 (d, *J* = 13.2 Hz, 1H), 2.71 (d, *J* = 13.4 Hz, 1H), 1.28 (s, 3H).

^{13}C NMR (101 MHz, $CDCl_3$) δ 174.6, 158.3, 153.0 (d, *J* = 250.8 Hz), 149.0, 139.0 (d, *J* = 3.1 Hz), 137.8, 131.4 (2C), 130.9 (d, *J* = 4.0 Hz), 130.5 (2C), 129.7, 129.7 (d, *J* = 3.7 Hz), 128.1 (2C), 126.5, 121.6 (d, *J* = 2.5 Hz), 118.7 (d, *J* = 18.1 Hz), 115.9 (d, *J* = 7.7 Hz), 113.5 (2C), 110.5 (d, *J* = 19.5 Hz), 55.2, 50.1, 46.6, 45.9, 19.7.

^{19}F NMR (376 MHz, $CDCl_3$) δ -129.5.

HRMS (MALDI) [M + H] calculated for $[C_{27}H_{26}FN_2O_2]^+$ = 429.1973 found: 429.2046.

$[\alpha]_D^{20}$= -5.5 (c = 0.45, $CHCl_3$).

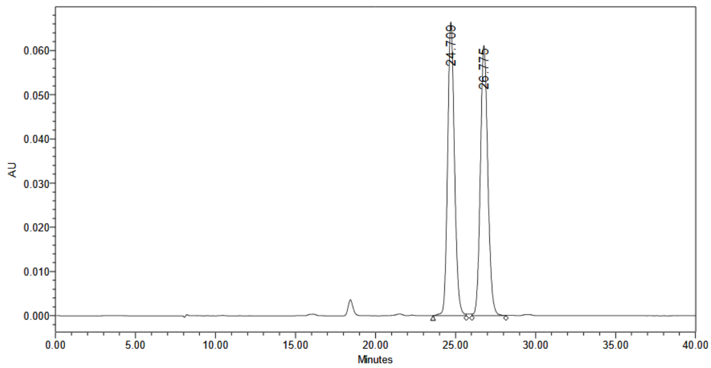

	RT	Area	% Area	Height
1	24.709	1967233	49.98	66438
2	26.775	1969021	50.02	61083

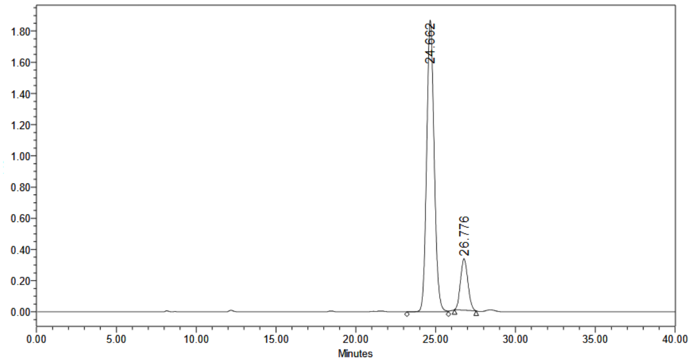

	RT	Area	% Area	Height
1	24.662	61271219	85.26	1871559
2	26.776	10595756	14.74	330935

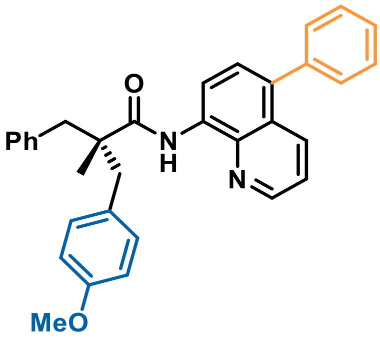

3cd

The product **3cd** was synthesized according to the general procedure using **1cd** (114. mg) as the substrate. The purification was performed by column chromatography (PE/DCM = 90:10) yielding the title compound as a colorless oil (102.2 mg, 70% yield). The enantiomeric excess was determined by HPLC analysis on a Daicel Chiralpak IC-5 column: 90:10 Hexane/IPA, flow rate 0.5mL/min, λ= 250.0 nm: T_RMajor = 34.97 min, T_RMinor = 38.73 min. er = 87.0: 13.0.

^{1}H NMR (400 MHz, $CDCl_3$) δ 10.01 (s, 1H), 8.92 (d, *J* = 8.0 Hz, 1H), 8.64 (dd, *J* = 4.2, 1.7 Hz, 1H), 8.24 (dd, *J* = 8.5, 1.6 Hz, 1H), 7.56 – 7.40 (m, 6H), 7.33 (dd, *J* = 8.5, 4.2 Hz, 1H), 7.25 – 7.16 (m, 4H), 7.16 – 7.11 (m, 3H), 6.75 – 6.70 (m, 2H), 3.69 (s, 3H), 3.50 (d, *J* = 13.2 Hz, 1H), 3.44 (d, *J* = 13.4 Hz, 1H), 2.79 (d, *J* = 13.3 Hz, 1H), 2.75 (d, *J* = 13.5 Hz, 1H), 1.33 (s, 3H).

^{13}C NMR (101 MHz, $CDCl_3$) δ 174.8, 158.3, 148.0, 139.4, 138.8, 137.9, 134.6, 134.2, 133.8, 131.4 (2C), 130.5 (2C), 130.2 (2C), 129.8, 128.6 (2C), 128.2 (2C), 128.0, 127.6, 126.5, 126.3, 121.5, 116.0, 113.6 (2C), 55.2, 50.2, 46.7, 46.0, 19.8.

HRMS (MALDI) [M + H] calculated for $[C_{33}H_{31}N_2O_2]^+$ = 487.2380 found: 487.2385.

$[\alpha]_D^{20}$= -0.7 (c = 0.60, $CHCl_3$).

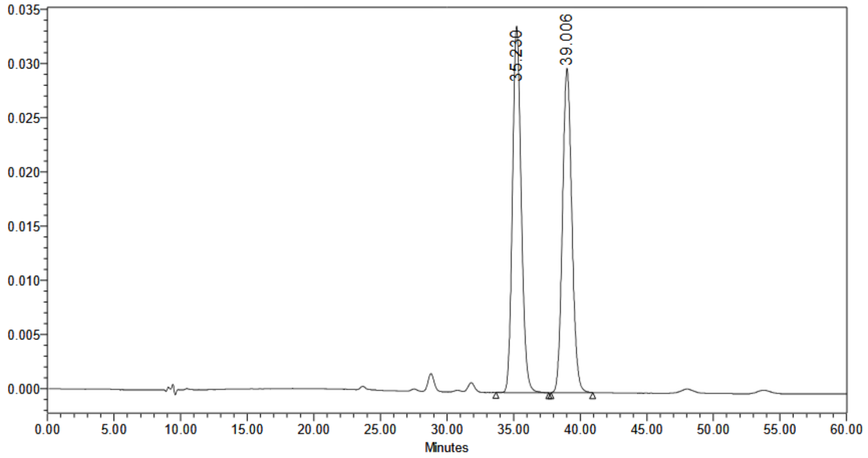

	RT	Area	% Area	Height
1	35.230	1537464	51.12	33830
2	39.006	1469848	48.88	29940

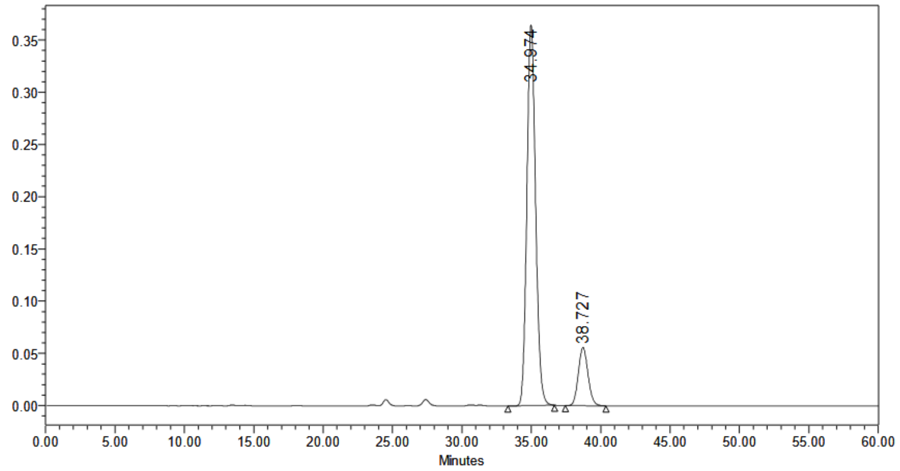

	RT	Area	% Area	Height
1	34.974	15960406	87.22	364627
2	38.725	2338036	12.78	52603

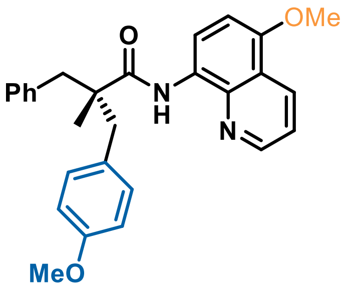

3ce

The product **3ce** was synthesized according to the general procedure using **1ce** (100.3mg) as the substrate. The purification was performed by column chromatography (PE/DCM = 90:10) yielding the title compound as a colorless oil (76.5 mg, 58% yield). The enantiomeric excess was determined by HPLC analysis on a Daicel Chiralpak IC-5 column: 90:10 Hexane/IPA, flow rate 0.5mL/min, λ= 321.0 nm: T_RMajor = 41.10 min, T_RMinor = 47.51 min. er = 67.0: 33.0.

^{1}H NMR (400 MHz, $CDCl_3$) δ 9.64 (s, 1H), 8.78 (d, *J* = 8.5 Hz, 1H), 8.63 (dd, *J* = 4.3, 1.7 Hz, 1H), 8.52 (dd, *J* = 8.4, 1.7 Hz, 1H), 7.36 (dd, *J* = 8.4, 4.3 Hz, 1H), 7.23 – 7.06 (m, 7H), 6.86 (d, *J* = 8.6 Hz, 1H), 6.73 – 6.64 (m, 2H), 4.00 (s, 3H), 3.67 (s, 3H), 3.46 (d, *J* = 13.2 Hz, 1H), 3.40 (d, *J* = 13.4 Hz, 1H), 2.75 (d, *J* = 13.2 Hz, 1H), 2.71 (d, *J* = 13.4 Hz, 1H), 1.26 (d, *J* = 3.9 Hz, 3H).

^{13}C NMR (101 MHz, $CDCl_3$) δ 174.3, 158.3, 150.3, 148.7, 139.5, 138.0, 131.4, 131.1, 130.5, 129.9, 128.1, 127.9, 126.4, 120.7, 120.4, 116.5, 113.5, 104.4, 55.9, 55.2, 50.0, 46.6, 45.9, 19.7.

HRMS (ESI) [M + Na] calculated for $[C_{28}H_{29}N_2O_3]^+$ = 441.2173 found: 441.2153.

$[\alpha]_D^{20}$= -5.2 (c = 0.74, $CHCl_3$).

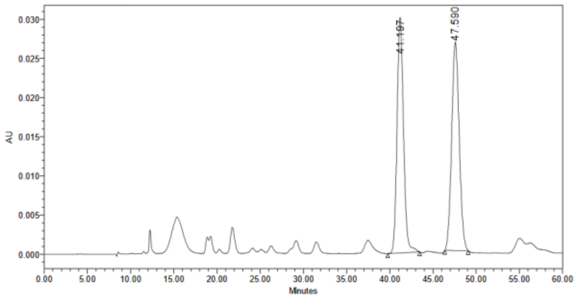

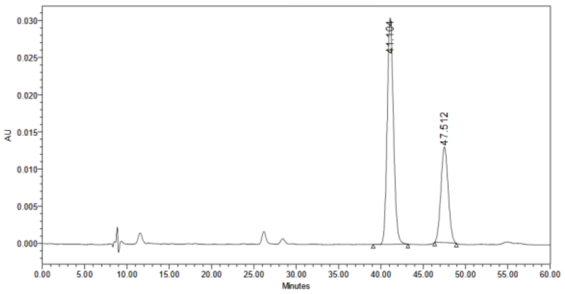

	RT	Area	% Area	Height	Peak Width (sec)
1	41.197	1571052	49.27	30068	67.72
2	47.590	1617731	50.73	26596	67.72

	RT	Area	% Area	Height
1	41.104	1579775	67.10	30470
2	47.512	774430	32.90	12893

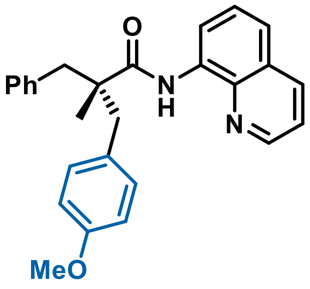

3aa

The product **3aa** was synthesized according to the general procedure using **1aa** (91.3mg) as the substrate. The purification was performed by column chromatography (PE/DCM = 90:10) yielding the title compound as a colorless oil (103.1 mg, 84% yield). The enantiomeric excess was determined by HPLC analysis on a Daicel Chiralpak IC-5 column: 90:10 Hexane/IPA, flow rate 0.5mL/min, λ= 250.0 nm: T_RMajor = 28.35 min, T_RMinor = 31.29 min. er = 92.0: 8.0.

^{1}H NMR (400 MHz, $CDCl_3$) δ 9.91 (s, 1H), 8.86 (dd, *J* = 7.6, 1.4 Hz, 1H), 8.62 (dd, *J* = 4.2, 1.7 Hz, 1H), 8.10 (s, 1H), 7.61 – 7.51 (m, 1H), 7.48 (dd, *J* = 8.3, 1.4 Hz, 1H), 7.37 (dd, *J* = 8.3, 4.2 Hz, 1H), 7.23 – 7.06 (m, 7H), 6.69 (d, *J* = 8.7 Hz, 2H), 3.67 (s, 3H), 3.47 (d, *J* = 13.2 Hz, 1H), 3.41 (d, *J* = 13.4 Hz, 1H), 2.77 (d, *J* = 13.2 Hz, 1H), 2.73 (d, *J* = 13.4 Hz, 1H), 1.30 (s, 3H).

^{13}C NMR (101 MHz, $CDCl_3$) δ 174.8, 158.3, 148.2, 138.8, 137.9, 136.2, 134.4, 131.4 (2C), 130.5 (2C), 129.8, 128.1 (2C), 127.9, 127.5, 126.5, 121.6, 121.5, 116.4, 113.5 (2C), 55.2, 50.2, 46.6, 45.9, 19.8.

HRMS (ESI) [M + Na] calculated for $[C_{27}H_{26}N_2NaO_2]^+$ = 433.1886 found: 433.1848.

Melting point = 66.7°C Melting point = 66.7°C (starting).

$[\alpha]_D^{20}$= -13.5 (c = 0.58, $CHCl_3$).

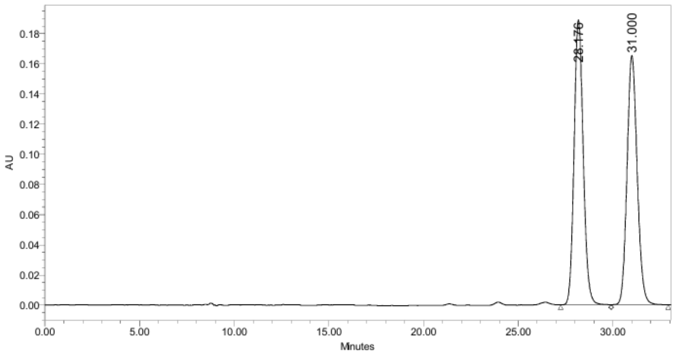

	RT	Area	% Area	Height
1	28.176	6361646	50.84	189007
2	31.000	6152507	49.16	165351

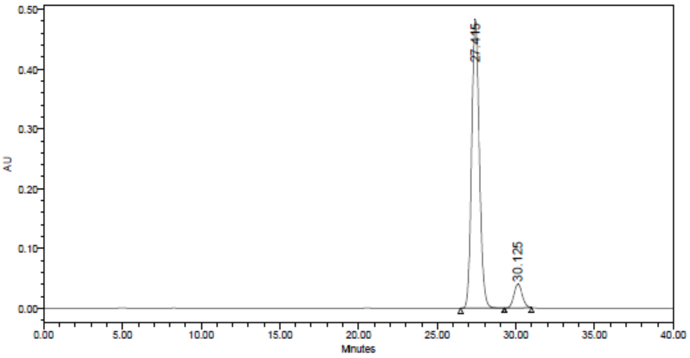

	RT	Area	% Area	Height
1	27.415	16403024	91.82	482423
2	30.125	1461175	8.18	39883

After recrystallization

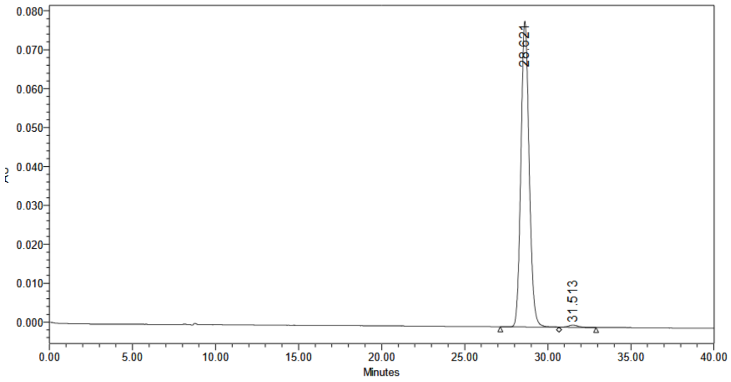

	RT	Area	% Area	Height
1	28.621	2712669	99.15	78693
2	31.513	23376	0.85	576

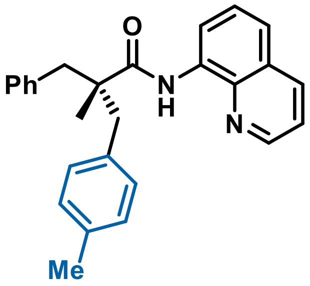

3ab

The product **3ab** was synthesized according to the general procedure using **1aa** (91.3 mg) as the substrate. The purification was performed by column chromatography (PE/DCM = 90:10) yielding the title compound as a colorless oil (95.9 mg, 81% yield). The enantiomeric excess was determined by HPLC analysis on a Daicel Chiralpak IC-5 column: 95:05 Hexane/IPA, flow rate 0.5mL/min, λ= 250.0 nm: T_RMajor = 23.56 min, T_RMinor = 26.73 min. er = 93.0:7.0.

^{1}H NMR (400 MHz, $CDCl_3$) δ 9.93 (s, 1H), 8.87 (dd, *J* = 7.6, 1.4 Hz, 1H), 8.62 (dd, *J* = 4.2, 1.7 Hz, 1H), 8.10 (dd, *J* = 8.3, 1.7 Hz, 1H), 7.55 (d, *J* = 7.8 Hz, 1H), 7.49 (d, *J* = 1.4 Hz, 1H), 7.37 (dd, *J* = 8.3, 4.2 Hz, 1H), 7.22 – 7.06 (m, 7H), 6.97 (dd, *J* = 8.2, 0.8 Hz, 2H), 3.48 (d, *J* = 13.2

Hz, 1H), 3.44 (d, *J* = 13.1 Hz, 1H), 2.79 (d, *J* = 5.4 Hz, 1H), 2.75 (d, *J* = 5.4 Hz, 1H), 2.21 (s, 3H), 1.30 (s, 3H).

^{13}C NMR (101 MHz, $CDCl_3$) δ 174.8, 148.2, 138.8, 137.9, 136.2, 136.0, 134.6, 134.4, 130.5 (2C), 130.4 (2C), 128.8 (2C), 128.1 (2C), 127.9, 127.5, 126.5, 121.5, 121.5, 116.4, 50.1, 46.6, 46.3, 21.1, 19.8.

HRMS (ESI) [M + Na] calculated for $[C_{27}H_{26}N_2NaO]^+$ = 417.1943 found: 417.1911.

$[\alpha]_D^{20}$= +1.3 (c = 0.36, $CHCl_3$).

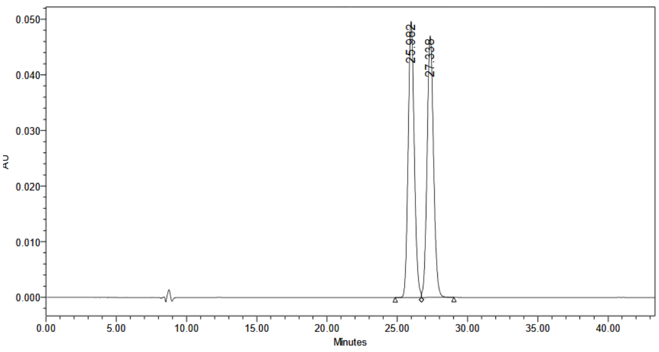

	RT	Area	% Area	Height
1	25.982	1494250	49.89	49674
2	27.338	1500853	50.11	47078

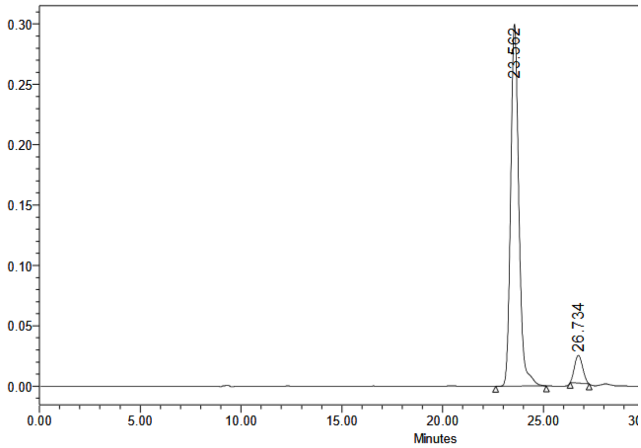

	RT	Area	% Area	Height
1	23.562	8534179	93.07	300034
2	26.734	635268	6.93	23011

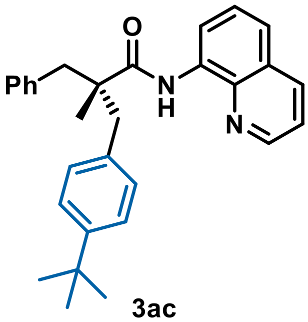

3ac

The product **3ac** was synthesized according to the general procedure using **1aa** (91.3 mg) as the substrate. The purification was performed by column chromatography (PE/DCM = 20:80) yielding the title compound as a colorless oil (91.7 mg, 70% yield). The enantiomeric excess was determined by HPLC analysis on a Daicel Chiralpak IC-5 column: 90:10 Hexane/IPA, flow rate 0.5mL/min, λ= 250.1 nm: T_RMajor = 20.69 min, T_RMinor = 18.23 min. er = 91.0: 9.0.

^{1}H NMR (400 MHz, $CDCl_3$) δ 9.89 (s, 1H), 8.87 (dd, *J* = 7.6, 1.4 Hz, 1H), 8.61 (dd, *J* = 4.2, 1.7 Hz, 1H), 8.10 (dd, *J* = 8.3, 1.7 Hz, 1H), 7.60 – 7.53 (m, 1H), 7.48 (dd, *J* = 8.3, 1.4 Hz, 1H), 7.36 (dd, *J* = 8.3, 4.2 Hz, 1H), 7.23 – 7.08 (m, 9H), 3.49 (d, *J* = 13.3 Hz, 1H), 3.41 (d, *J* = 13.2 Hz, 1H), 2.79 (d, *J* = 2.5 Hz, 1H), 2.76 (d, *J* = 2.5 Hz, 1H), 1.31 (s, 3H), 1.17 (s, 9H).

^{13}C NMR (101 MHz, $CDCl_3$) δ 174.8, 149.2, 148.1, 138.8, 137.9, 136.2, 134.6, 134.4, 130.5 (2C), 130.1 (2C), 128.1 (2C), 127.9, 127.5, 126.5, 125.0 (2C), 121.5, 121.5, 116.4, 50.1, 46.6, 46.3, 34.4, 31.4 (3C), 19.9.

HRMS (ESI) [M + Na] calculated for $[C_{30}H_{32}N_2NaO]^+$ = 459.2406 found: 459.2385.

$[\alpha]_D^{20}$= -3.7 (c = 0.40, $CHCl_3$).

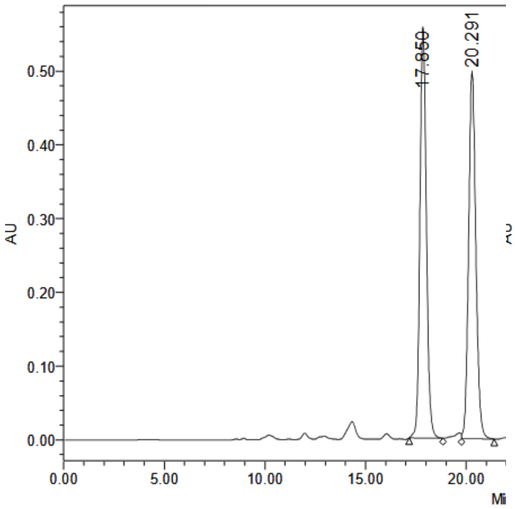

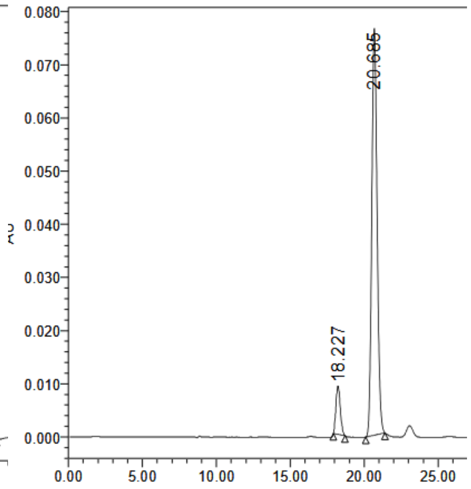

	RT	Area	% Area	Height
1	17.850	12376685	49.78	558103
2	20.291	12484598	50.22	498585

	RT	Area	% Area	Height
1	18.227	188010	8.99	9124
2	20.685	1903455	91.01	76540

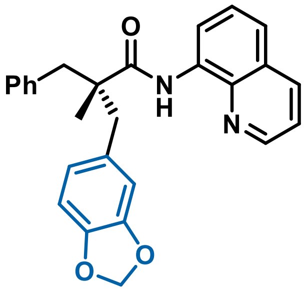

3ad

The product **3ad** was synthesized according to the general procedure using **1aa** (91.3 mg) as the substrate. The purification was performed by column chromatography (PE/DCM = 20:80) yielding the title compound as a colorless oil (89.2 mg, 71% yield). The enantiomeric excess was determined by HPLC analysis on a Daicel Chiralpak IC-5 column: 90:10 Hexane/IPA, flow rate 0.5mL/min, λ= 250.0 nm: T_RMajor = 29.73 min, T_RMinor = 33.92 min. er = 91.0: 9.0.

^{1}H NMR (400 MHz, $CDCl_3$) δ 9.90 (s, 1H), 9.83 (s, 1H), 8.77 (dd, *J* = 7.6, 1.4 Hz, 1H), 8.57 (dd, *J* = 4.2, 1.7 Hz, 1H), 8.04 (dd, *J* = 8.3, 1.7 Hz, 1H), 7.48 (t, *J* = 7.9 Hz, 1H), 7.41 (dd, *J* = 8.3, 1.4 Hz, 1H), 7.31 (dd, *J* = 8.3, 4.2 Hz, 1H), 7.22 – 7.07 (m, 5H), 6.71 – 6.63 (m, 2H), 6.60 (d, *J* = 7.9 Hz, 1H), 5.81 (d, *J* = 1.5 Hz, 1H), 5.75 (d, *J* = 1.4 Hz, 1H), 3.45 (d, *J* = 13.2 Hz, 1H), 3.40 (d, *J* = 13.4 Hz, 1H), 2.76 (d, *J* = 13.2 Hz, 1H), 2.68 (d, *J* = 13.4 Hz, 1H), 1.31 (s, 3H).

^{13}C NMR (101 MHz, $CDCl_3$) δ 174.5, 148.1, 147.2, 146.1, 138.7, 137.6, 136.1, 134.2, 131.3, 130.4 (2C), 128.0 (2C), 127.8, 127.4, 126.4, 123.4, 121.4 (2C), 116.4, 110.7, 107.9, 100.7, 50.2, 46.7, 46.5, 19.8.

HRMS (ESI) [M + Na] calculated for $[C_{27}H_{24}N_2NaO_3]^+$ = 447.1679 found: 447.1665.

$[\alpha]_D^{20}$= -4.7 (c = 0.50, $CHCl_3$).

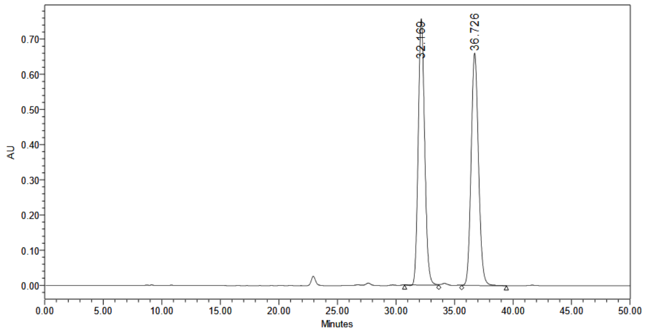

	RT	Area	% Area	Height
1	32.169	28941111	49.91	757929
2	36.726	29044940	50.09	661517

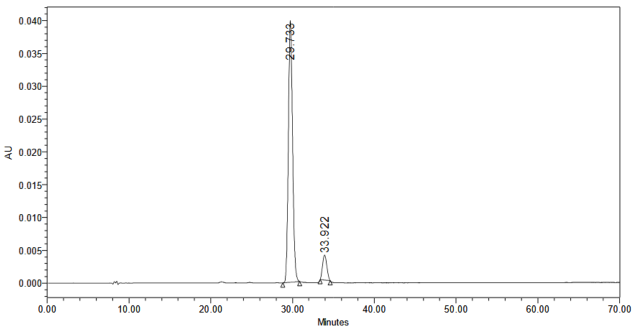

	RT	Area	% Area	Height
1	29.733	1422085	91.22	39889
2	33.922	136918	8.78	3797

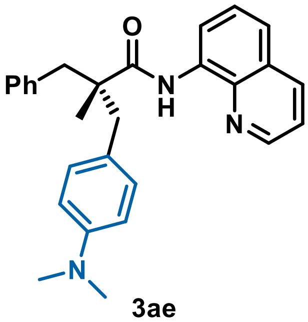

3ae

The product **3ae** was synthesized according to the general procedure using **1aa** (91.3 mg) as the substrate. The purification was performed by column chromatography (PE/EA = 90:10) yielding the title compound as a colorless oil (61 mg, 48% yield). The enantiomeric excess was determined by HPLC analysis on a Daicel Chiralpak ID-5 column: 90:10 Hexane/IPA, flow rate 0.5mL/min, λ= 250.0 nm: T_RMajor = 46.81 min, T_RMinor = 44.76 min. er = 91.0: 9.0.

^{1}H NMR (400 MHz, $CDCl_3$) δ 9.93 (s, 1H), 8.87 (dd, J = 7.6, 1.4 Hz, 1H), 8.63 (dd, J = 4.2, 1.7 Hz, 1H), 8.10 (dd, J = 8.3, 1.7 Hz, 1H), 7.58 – 7.52 (m, 1H), 7.48 (d, J = 7.0 Hz, 1H), 7.37 (dd, J = 8.2, 4.2 Hz, 1H), 7.21 – 7.05 (m, 7H), 6.58 (d, J = 8.1 Hz, 2H), 3.46 (d, J = 13.2 Hz, 1H), 3.36 (d, J = 13.4 Hz, 1H), 2.82 (s, 6H), 2.74 (dd, J = 13.3, 9.7 Hz, 2H), 1.30 (s, 3H).

^{13}C NMR (101 MHz, $CDCl_3$) δ 175.1, 148.2, 138.9, 138.1, 136.2, 134.5, 131.2, 130.5 (2C), 128.1 (2C), 127.9, 127.5, 126.4, 121.5, 121.4, 116.4, 112.7, 50.3, 46.5, 46.0, 19.8, 1.2 (2C).

HRMS (ESI) [M + Na] calculated for $[C_{28}H_{29}N_3NaO]^+$ = 446.2202 found: 446.2175.
$[\alpha]_D^{20}$= + 3.7 (c = 0.50, $CHCl_3$).

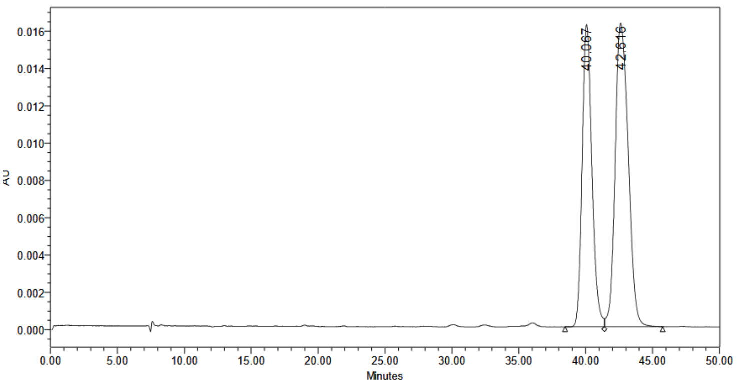

	RT	Area	% Area	Height
1	40.067	821770	42.66	16206
2	42.616	1104492	57.34	16271

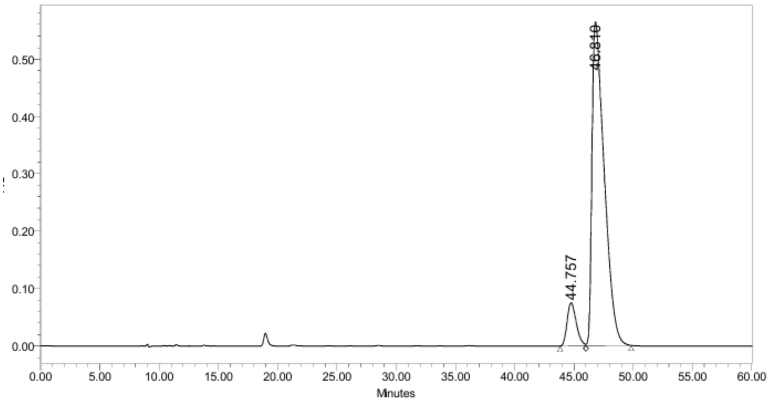

	RT	Area	% Area	Height
1	44.757	4025325	9.05	75212
2	46.810	40459675	90.95	564631

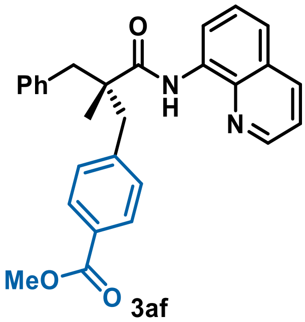

3af

The product **3af** was synthesized according to the general procedure using **1aa** (91.3 mg) as the substrate. The purification was performed by column chromatography (PE/DCM = 40:60) yielding the title compound as a colorless oil (80.3 mg, 61% yield). The enantiomeric excess was determined by HPLC analysis on a Daicel Chiralpak IC-5 column: 90:10 Hexane/IPA, flow rate 0.5mL/min, λ= 250.0 nm: T_RMajor = 47.53 min, T_RMinor = 63.46 min. er = 92.5: 7.5.

^{1}H NMR (400 MHz, $CDCl_3$) δ 9.89 (s, 1H), 8.85 (dd, *J* = 7.6, 1.4 Hz, 1H), 8.59 (dd, *J* = 4.2, 1.7 Hz, 1H), 8.10 (dd, *J* = 8.2, 1.7 Hz, 1H), 7.93 – 7.79 (m, 2H), 7.56 (dd, *J* = 8.3, 7.6 Hz, 1H), 7.49

(dd, J = 8.3, 1.4 Hz, 1H), 7.36 (dd, J = 8.2, 4.2 Hz, 1H), 7.28 (d, J = 1.8 Hz, 2H), 7.23 – 7.09 (m, 5H), 3.82 (s, 3H), 3.56 (d, J = 13.0 Hz, 1H), 3.48 (d, J = 13.2 Hz, 1H), 2.81 (d, J = 11.9 Hz, 1H), 2.77 (s, 1H), 1.31 (s, 3H).

^{13}C NMR (101 MHz, $CDCl_3$) δ 174.3, 167.2, 148.2, 143.4, 138.8, 137.4, 136.3, 134.1, 130.5 (2C), 130.5 (2C), 129.4 (2C), 128.4, 128.2 (2C), 127.9, 127.5, 126.7, 121.7, 121.6, 116.5, 52.1, 50.1, 46.9, 46.5, 19.7.

HRMS (ESI) [M + Na] calculated for $[C_{28}H_{26}N_2NaO_3]^+$ = 461.1835 found: 461.1829.
$[\alpha]_D^{20}$= + 2.7 (c = 0.10, $CHCl_3$).

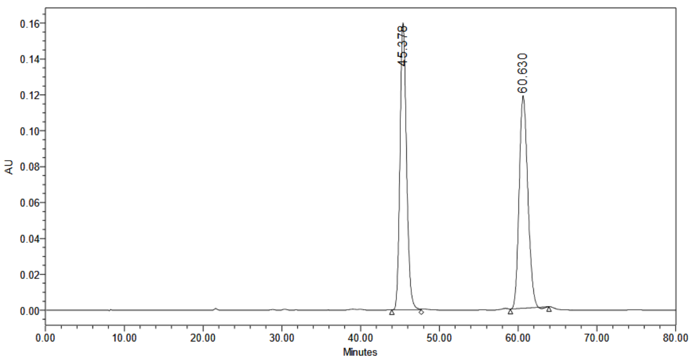

	RT	Area	% Area	Height
1	45.378	9158303	50.35	160159
2	60.630	9032458	49.65	118509

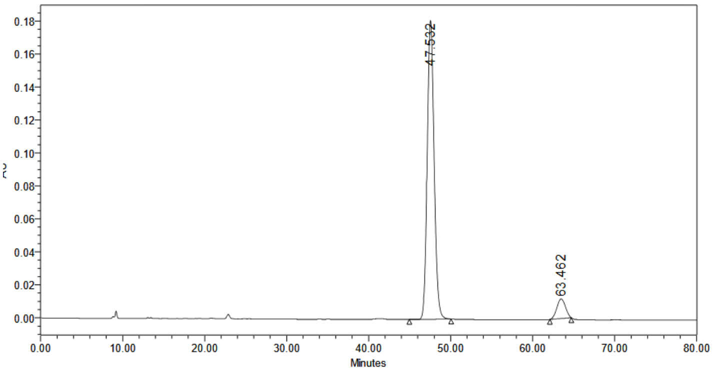

	RT	Area	% Area	Height
1	47.532	10592059	92.45	181346
2	63.462	865130	7.55	11908

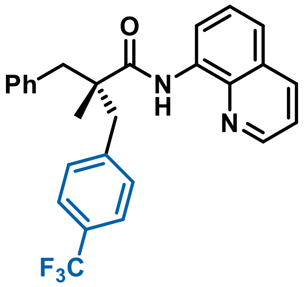

3ag

The product **3ag** was synthesized according to the general procedure using **1aa** (91.3 mg) as the substrate. The purification was performed by column chromatography (PE/DCM = 40:60) yielding the title compound as a colorless oil (82.3 mg, 44% yield). The enantiomeric excess was determined by HPLC analysis on a Daicel Chiralpak IC-5 column: 95:5 Hexane/IPA, flow rate 0.5mL/min, λ= 250.0 nm: T_RMajor = 16.58 min, T_RMinor = 18.38 min. er = 92.0:8.0.

^{1}H NMR (400 MHz, $CDCl_3$) δ 9.87 (s, 1H), 8.83 (dd, *J* = 7.5, 1.5 Hz, 1H), 8.60 (dd, *J* = 4.2, 1.7 Hz, 1H), 8.11 (dd, *J* = 8.3, 1.7 Hz, 1H), 7.59 – 7.53 (m, 1H), 7.50 (dd, *J* = 8.3, 1.5 Hz, 1H), 7.41 – 7.35 (m, 3H), 7.32 – 7.28 (m, 2H), 7.23 – 7.10 (m, 5H), 3.56 (d, *J* = 13.1 Hz, 1H), 3.48 (d, *J* = 13.2 Hz, 1H), 2.80 (dd, *J* = 18.9, 13.2 Hz, 2H), 1.31 (s, 3H).

^{13}C NMR (101 MHz, $CDCl_3$) δ 174.2, 148.3, 142.0 (d, *J* = 1.5 Hz), 138.8, 137.4, 136.3, 134.1, 130.7 (2C), 130.5 (2C), 128.8 (q, *J* = 32.4 Hz), 128.2 (2C), 127.9, 127.4, 126.7, 125.0 (q, *J* = 3.8 Hz, 2C), 124.3 (q, *J* = 271.8 Hz), 121.8, 121.6, 116.5, 50.1, 46.9, 46.3, 19.8.

^{19}F NMR (376 MHz, $CDCl_3$) δ -62.4.

HRMS (ESI) [M + Na] calculated for $[C_{27}H_{23}F_3N_2NaO]^+$ = 471,1660 found: 471.1646.

$[\alpha]_D^{20}$= -1.7 (c = 0.30, $CHCl_3$).

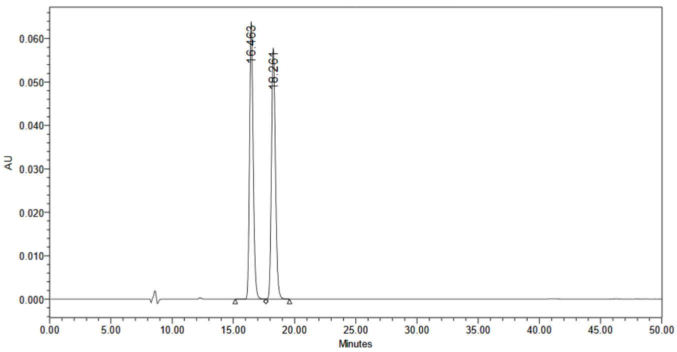

	RT	Area	% Area	Height
1	16.463	1315336	50.07	63955
2	18.261	1311413	49.93	57828

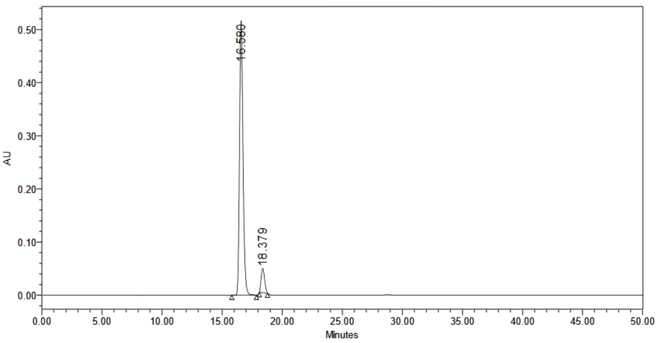

	RT	Area	% Area	Height
1	16.580	10467136	92.04	517069
2	18.379	904773	7.96	45757

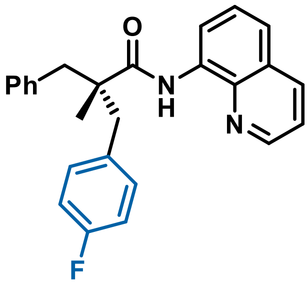

3ah

The product **3ah** was synthesized according to the general procedure using **1aa** (91.3 mg) as the substrate. The purification was performed by column chromatography (PE/DCM = 30:70) yielding the title compound as a colorless oil (89.6 mg, 75% yield). The enantiomeric excess was determined by HPLC analysis on a Daicel Chiralpak IC-5 column: 95:5 Hexane/IPA, flow rate 0.5mL/min, λ= 250.0 nm: T_RMajor = 21.43 min, T_RMinor = 23.15 min. er = 90.0: 10.0.

^{1}H NMR (400 MHz, $CDCl_3$) δ 9.89 (s, 1H), 8.85 (dd, *J* = 7.5, 1.4 Hz, 1H), 8.62 (dd, *J* = 4.2, 1.7 Hz, 1H), 8.11 (dd, *J* = 8.3, 1.7 Hz, 1H), 7.59 – 7.53 (m, 1H), 7.52 – 7.47 (m, 1H), 7.37 (dd, *J* = 8.2, 4.2 Hz, 1H), 7.23 – 7.16 (m, 4H), 7.16 – 7.11 (m, 3H), 6.88 – 6.79 (m, 2H), 3.49 (d, *J* = 2.6 Hz, 1H), 3.45 (d, *J* = 2.7 Hz, 1H), 2.78 (d, *J* = 13.2 Hz, 1H), 2.72 (d, *J* = 13.4 Hz, 1H), 1.30 (s, 3H).

^{13}C NMR (101 MHz, $CDCl_3$) δ 174.5, 161.8 (d, *J* = 244.3 Hz), 148.2, 138.8, 137.6, 136.3, 134.2, 133.4 (d, *J* = 3.3 Hz), 131.8 (d, *J* = 7.8 Hz, 2C), 130.5 (2C), 128.2 (2C), 127.9, 127.5, 126.6, 121.7, 121.6, 116.4, 114.9 (d, *J* = 21.0 Hz, 2C), 50.1, 46.8, 45.8, 19.7.

^{19}F NMR (376 MHz, $CDCl_3$) δ -116.9.

HRMS (MALDI) [M + H] calculated for $[C_{26}H_{24}FN_2O]^+$ = 399.1867 found: 399.1858.

$[\alpha]_D^{20}$= +1.0 (c = 0.93, $CHCl_3$).

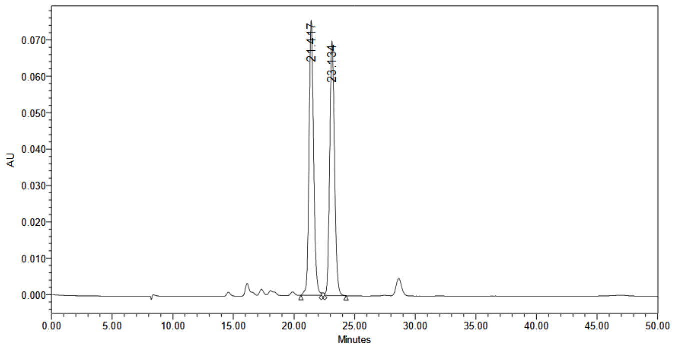

	RT	Area	% Area	Height
1	21.417	1968867	50.45	75629
2	23.134	1933368	49.55	69941

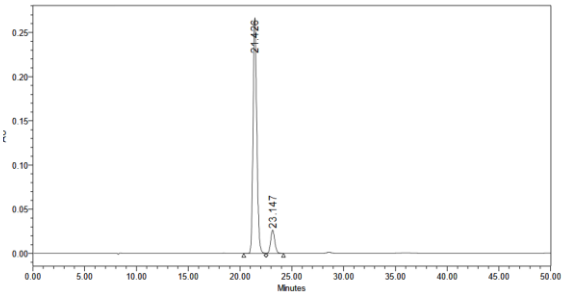

	RT	Area	% Area	Height
1	21.426	6798606	90.24	266874
2	23.147	735459	9.76	26513

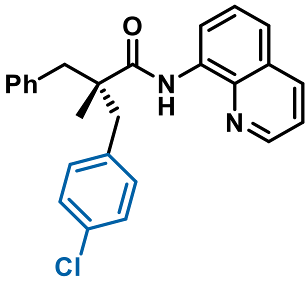

3ai

The product **3ai** was synthesized according to the general procedure using **1aa** (91.3 mg) as the substrate. The purification was performed by column chromatography (PE/DCM = 30:70) yielding the title compound as a colorless oil (93.4 mg, 75% yield). The enantiomeric excess was determined by HPLC analysis on a Daicel Chiralpak IA-5 column: 90:10 Hexane/IPA, flow rate 0.5mL/min, λ= 250.0 nm: T_RMajor = 17.31 min, T_RMinor = 18.54 min. er = 91.0:9.0.

^{1}H NMR (400 MHz, $CDCl_3$) δ 9.89 (s, 1H), 8.84 (dd, *J* = 7.5, 1.4 Hz, 1H), 8.62 (dd, *J* = 4.2, 1.7 Hz, 1H), 8.11 (dd, *J* = 8.3, 1.7 Hz, 1H), 7.56 (dd, *J* = 8.3, 7.5 Hz, 1H), 7.50 (dd, *J* = 8.3, 1.4 Hz, 1H), 7.38 (dd, *J* = 8.3, 4.2 Hz, 1H), 7.23 – 7.08 (m, 9H), 3.48 (d, *J* = 2.7 Hz, 1H), 3.45 (d, *J* = 2.7 Hz, 1H), 2.79 (d, *J* = 13.3 Hz, 1H), 2.70 (d, *J* = 13.2 Hz, 1H), 1.30 (s, 3H).

^{13}C NMR (101 MHz, $CDCl_3$) δ 174.4, 148.3, 138.8, 137.5, 136.3, 136.2, 134.2, 132.4, 131.8 (2C), 130.5 (2C), 128.3 (2C), 128.2 (2C), 128.0, 127.4, 126.6, 121.7, 121.6, 116.5, 50.1, 46.8, 45.9, 19.7.

HRMS (ESI) [M + Na] calculated for $[C_{26}H_{23}ClN_2NaO]^+$ = 437.1391 found: 437.1396.

$[\alpha]_D^{20}$= -5.7 (c = 0.40, $CHCl_3$).

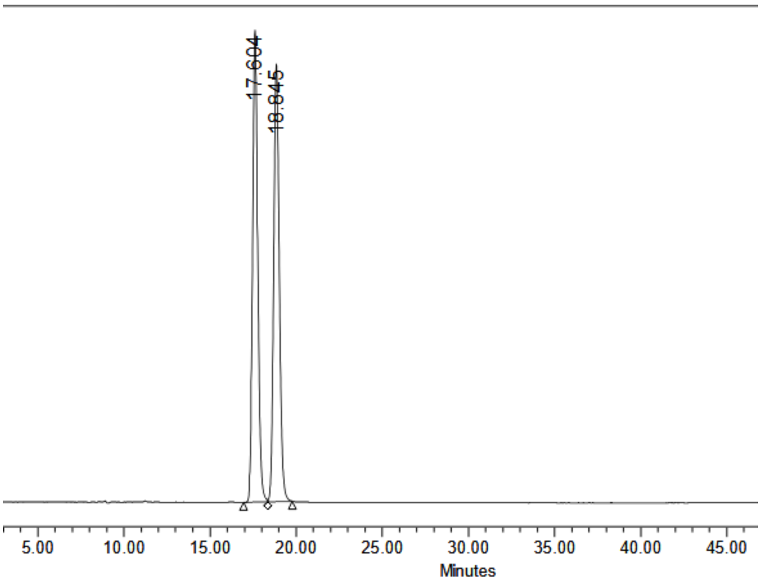

	RT	Area	% Area	Height
1	17.604	3203820	50.05	152247
2	18.845	3197118	49.95	141020

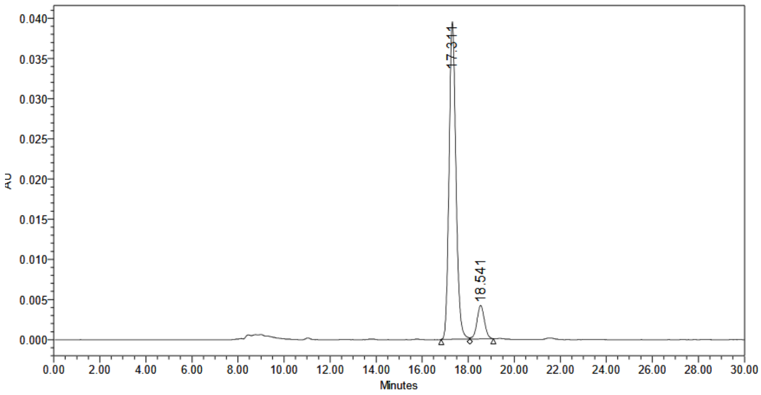

	RT	Area	% Area	Height
1	17.311	865141	91.19	42043
2	18.543	83569	8.81	4120

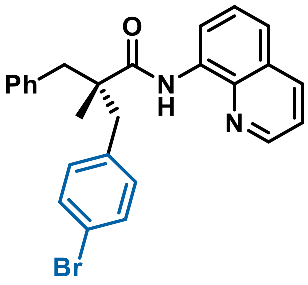

3aj

The product **3aj** was synthesized according to the general procedure using **1aa** (91.3 mg) as the substrate. The purification was performed by column chromatography (PE/DCM = 25:75) yielding the title compound as a colorless oil (93.9 mg, 71% yield). The enantiomeric excess was determined by HPLC analysis on a Daicel Chiralpak IA-5 column: 90:10 Hexane/IPA, flow rate 0.5mL/min, λ= 250.0 nm: T_RMajor = 17.54 min, T_RMinor = 19.08 min. er = 90.0:10.0.

^{1}H NMR (400 MHz, $CDCl_3$) δ 9.95 – 9.79 (m, 1H), 8.83 (dd, *J* = 7.5, 1.5 Hz, 1H), 8.63 (dd, *J* = 4.2, 1.7 Hz, 1H), 8.11 (dd, *J* = 8.3, 1.7 Hz, 1H), 7.55 (dd, *J* = 8.3, 7.5 Hz, 1H), 7.50 (dd, *J* = 8.3, 1.5 Hz, 1H), 7.38 (dd, *J* = 8.3, 4.2 Hz, 1H), 7.26 – 7.09 (m, 7H), 7.09 – 7.03 (m, 2H), 3.45 (dd, *J* = 13.2, 1.6 Hz, 2H), 2.80 (s, 1H), 2.68 (d, *J* = 13.2 Hz, 1H), 1.29 (s, 3H).

^{13}C NMR (101 MHz, $CDCl_3$) δ 174.4, 148.3, 138.8, 137.5, 136.8, 136.3, 134.2, 132.2 (2C), 131.2 (2C), 130.5 (2C), 128.2 (2C), 128.0, 127.4, 126.6, 121.7, 121.7, 120.6, 116.5, 50.0, 46.8, 46.0, 19.7.

HRMS (ESI) [M + Na] calculated for $[C_{26}H_{23}BrN_2NaO]^+$ = 481.0886 found: 481.0870.

$[\alpha]_D^{20}$= -10.1 (c = 0.60, $CHCl_3$).

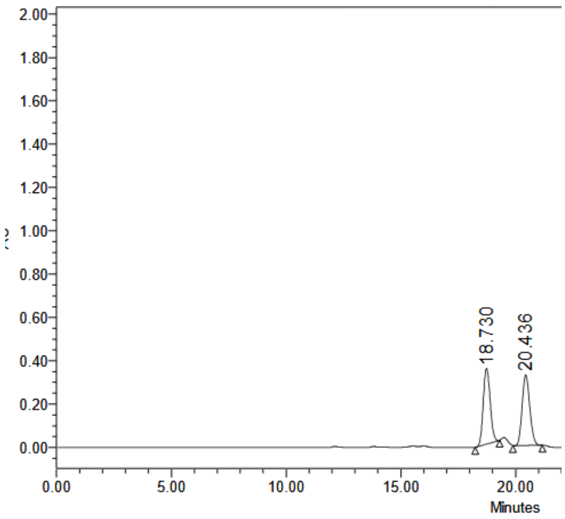

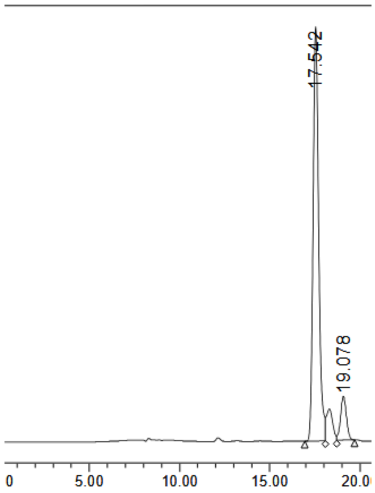

	RT	Area	% Area	Height
1	18.730	7279425	49.15	349632
2	20.436	7531928	50.85	325844

	RT	Area	% Area	Height
1	17.542	1099062	90.02	51267
2	19.078	121859	9.98	5396

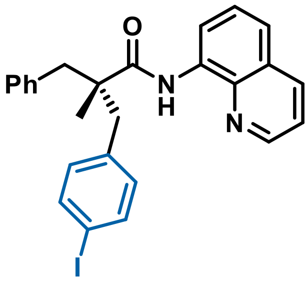

3ak

The product **3ak** was synthesized according to the general procedure using **1aa** (91.3 mg) as the substrate. The purification was performed by column chromatography (PE/DCM = 10:90) yielding the title compound as a colorless oil (107.9 mg, 55% yield). The enantiomeric excess was determined by HPLC analysis on a Daicel Chiralpak IC-5 column: 90:10 Hexane/IPA, flow rate 0.5mL/min, λ= 250.0 nm: T_RMajor = 18.25 min, T_RMinor = 20.07 min. er = 93.0:7.0.

^{1}H NMR (400 MHz, $CDCl_3$) δ 9.88 (s, 1H), 8.83 (dd, *J* = 7.5, 1.5 Hz, 1H), 8.63 (dd, *J* = 4.2, 1.7 Hz, 1H), 8.11 (dd, *J* = 8.3, 1.7 Hz, 1H), 7.55 (dd, *J* = 8.3, 7.5 Hz, 1H), 7.50 (dd, *J* = 8.3, 1.5 Hz, 1H), 7.48 – 7.43 (m, 2H), 7.39 (dd, *J* = 8.3, 4.2 Hz, 1H), 7.24 – 7.09 (m, 5H), 6.96 – 6.90 (m, 2H), 3.46 (d, *J* = 6.1 Hz, 1H), 3.43 (d, *J* = 6.1 Hz, 1H), 2.78 (d, *J* = 13.2 Hz, 1H), 2.67 (d, *J* = 13.2 Hz, 1H), 1.29 (s, 3H).

^{13}C NMR (101 MHz, $CDCl_3$) δ 174.3, 148.3, 138.8, 137.5, 137.5, 137.2 (2C), 136.3, 134.2, 132.5 (2C), 130.5 (2C), 128.2 (2C), 128.0, 127.4, 126.6, 121.7, 121.7, 116.5, 92.1, 50.0, 46.8, 46.1, 19.7.

HRMS (ESI) [M + Na] calculated for $[C_{26}H_{23}N_2NaIO_2]^+$ = 529.0753 found: 529.0736.

$[\alpha]_D^{20}$= -2.7 (c = 0.50, $CHCl_3$).

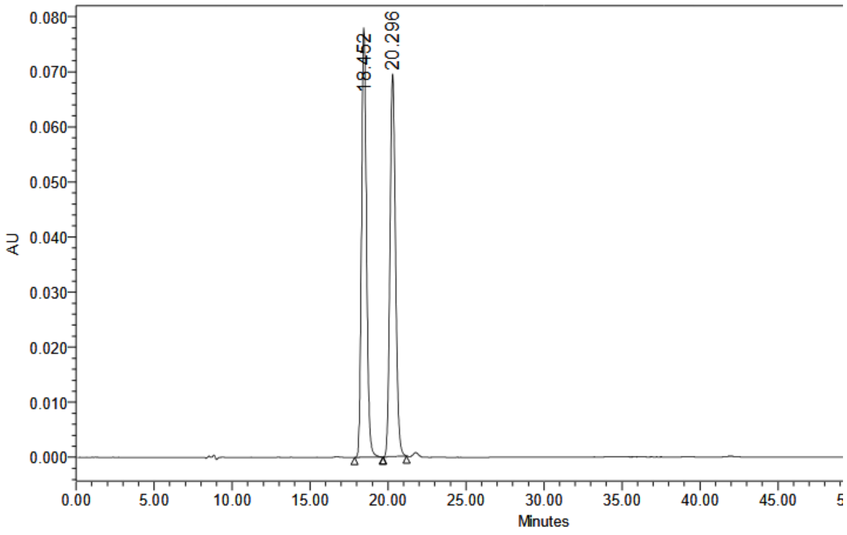

	RT	Area	% Area	Height
1	18.452	1734674	50.28	78014
2	20.296	1715206	49.72	69467

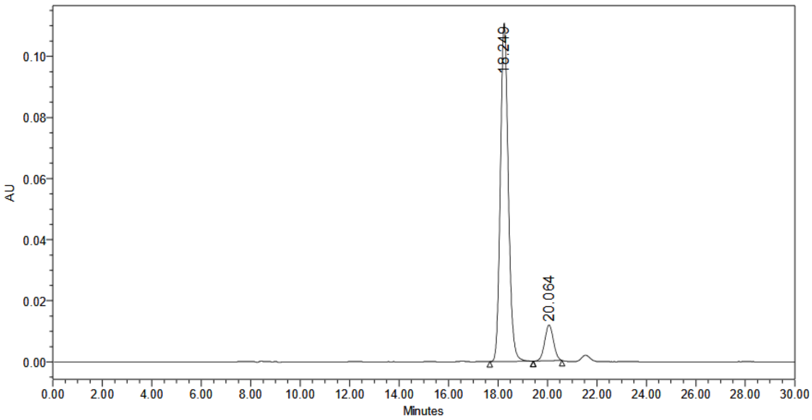

	RT	Area	% Area	Height
1	18.249	2474042	92.65	110842
2	20.070	196188	7.35	9676

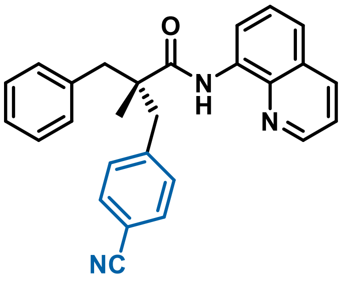

3al

The product **3al** was synthesized according to the general procedure using **1aa** (91.3 mg) as the substrate. The purification was performed by column chromatography (PE/DCM = 90:10) yielding the title compound as a colorless oil (53.4 mg, 44% yield). The enantiomeric excess was determined by HPLC analysis on a Daicel Chiralpak IA-5 column: 90:10 Hexane/IPA, flow rate 0.5mL/min, λ= 250.0 nm: T_RMajor = 81.51 min, T_RMinor = 86.96 min. er = 85.0:15.0.

^{1}H NMR (400 MHz, $CDCl_3$) δ 9.85 (s, 1H), 8.82 (dd, *J* = 7.5, 1.5 Hz, 1H), 8.61 (dd, *J* = 4.2, 1.7 Hz, 1H), 8.12 (dd, *J* = 8.3, 1.7 Hz, 1H), 7.58 – 7.54 (m, 1H), 7.53 – 7.49 (m, 1H), 7.43 – 7.37 (m, 3H), 7.31 – 7.27 (m, 2H), 7.23 – 7.11 (m, 5H), 3.57 (d, *J* = 13.0 Hz, 1H), 3.47 (d, *J* = 13.2 Hz, 1H), 2.82 (d, *J* = 13.2 Hz, 1H), 2.73 (d, *J* = 12.9 Hz, 1H), 1.31 (s, 3H).

^{13}C NMR (101 MHz, $CDCl_3$) δ 173.9, 148.3, 143.6, 138.7, 137.2, 136.3, 134.0, 131.9 (2C), 131.1 (2C), 130.5 (2C), 128.3 (2C), 128.0, 127.4, 126.8, 121.9, 121.7, 119.1, 116.5, 110.4, 50.2, 47.0, 46.5, 19.7.

HRMS (MALDI) [M + H] calculated for $[C_{27}H_{24}N_3O]^+$ = 406.1914 found: 406.1899.

$[\alpha]_D^{20}$= -10.8 (c = 0.33, $CHCl_3$).

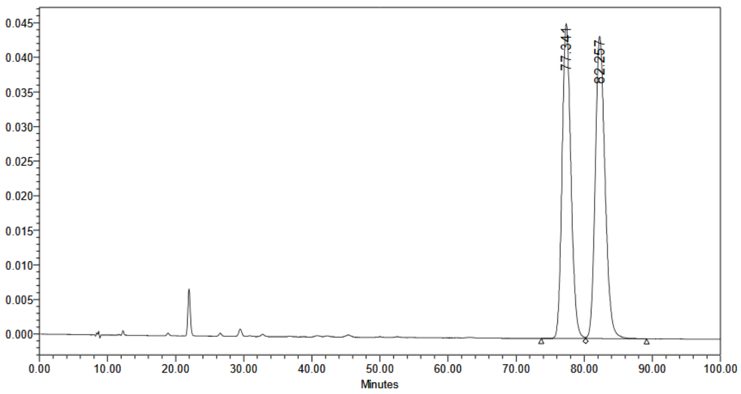

	RT	Area	% Area	Height
1	77.341	4099282	48.41	45557
2	82.257	4369234	51.59	43706

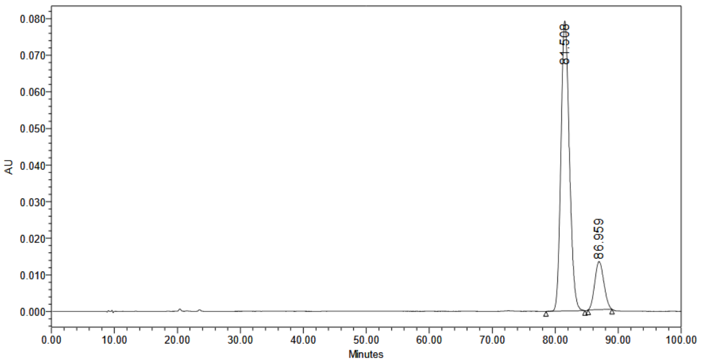

	RT	Area	% Area	Height
1	81.508	7375507	84.90	79277
2	86.959	1311867	15.10	13134

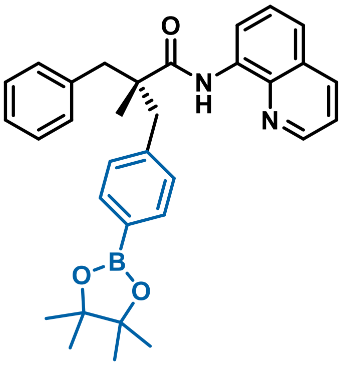

3am

The product **3am** was synthesized according to the general procedure using **1aa** (91.3 mg) as the substrate. The purification was performed by column chromatography (PE/DCM = 40:60) yielding the title compound as a colorless oil (188.2 mg, 58% yield). The enantiomeric excess was determined by HPLC analysis on a Daicel Chiralpak IC-5 column: 95:5 Hexane/IPA, flow rate 0.5mL/min, λ= 250.0 nm: T_RMajor = 23.92 min, T_RMinor = 26.01 min. er = 89.0:11.0.

^{1}H NMR (400 MHz, $CDCl_3$) δ 9.91 (s, 1H), 8.86 (dd, *J* = 7.6, 1.4 Hz, 1H), 8.61 (dd, *J* = 4.2, 1.7 Hz, 1H), 8.10 (dd, *J* = 8.3, 1.7 Hz, 1H), 7.63 – 7.59 (m, 2H), 7.55 (dd, *J* = 8.3, 7.6 Hz, 1H), 7.48 (dd, *J* = 8.3, 1.5 Hz, 1H), 7.36 (dd, *J* = 8.2, 4.2 Hz, 1H), 7.24 – 7.07 (m, 7H), 3.50 (d, *J* = 13.2 Hz, 1H), 3.46 (d, *J* = 13.3 Hz, 1H), 2.79 (d, *J* = 13.1 Hz, 2H), 1.28 (d, *J* = 1.9 Hz, 15H).

^{13}C NMR (101 MHz, $CDCl_3$) δ 174.7, 148.2, 141.2, 138.9, 137.7, 136.2, 134.6 (2C), 134.3, 131.7, 130.5 (2C), 130.0 (2C), 128.1 (2C), 128.0, 127.5, 126.5, 121.6, 121.5, 116.5, 83.8 (2C), 50.1, 46.8, 46.8, 25.0 (2C), 24.9 (2C), 19.7.

HRMS (ESI) [M + Na] calculated for $[C_{32}H_{26}N_2NaBO_3]^+$ = 529.2638 found: 529.2634.

$[\alpha]_D^{20}$= -5.7 (c = 0.30, $CHCl_3$).

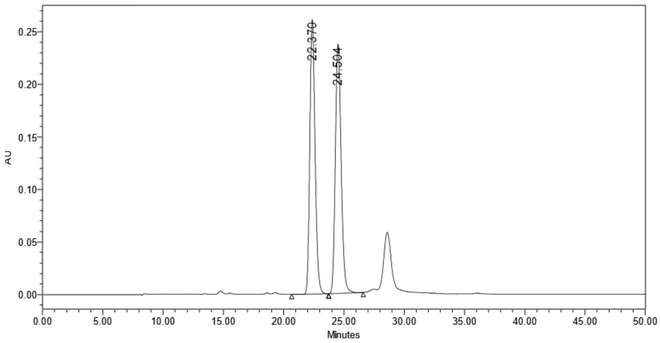

	RT	Area	% Area	Height
1	22.370	7918223	49.83	261454
2	24.504	7970990	50.17	237144

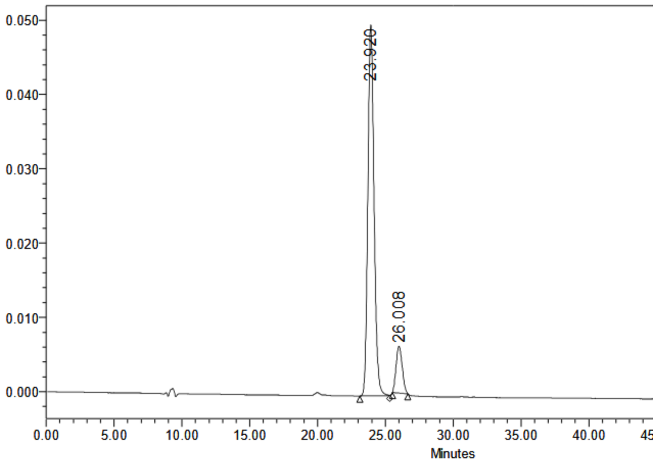

	RT	Area	% Area	Height
1	23.920	1583982	88.85	49956
2	26.008	198871	11.15	6301

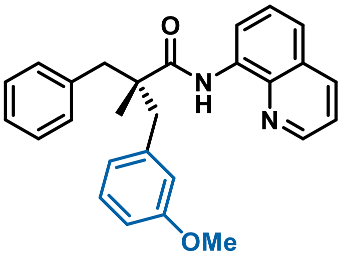

3an

The product **3an** was synthesized according to the general procedure using **1aa** (91.3 mg) as the substrate. The purification was performed by column chromatography (PE/DCM = 40:60) yielding the title compound as a colorless oil (83.8 mg, 68% yield). The enantiomeric excess was determined by HPLC analysis on a Daicel Chiralpak ID-5 column: 90:10 Hexane/IPA, flow rate 0.5mL/min, λ= 250.0 nm: T_RMajor = 44.09 min, T_RMinor = 39.88 min. er = 90.0:10.0.

^{1}H NMR (400 MHz, $CDCl_3$) δ 9.92 (s, 1H), 8.88 (dd, *J* = 7.6, 1.4 Hz, 1H), 8.62 (dd, *J* = 4.2, 1.7 Hz, 1H), 8.10 (dd, *J* = 8.3, 1.7 Hz, 1H), 7.58 – 7.52 (m, 1H), 7.51 – 7.46 (m, 1H), 7.37 (dd, *J* = 8.2, 4.2 Hz, 1H), 7.24 – 7.14 (m, 4H), 7.14 – 7.09 (m, 1H), 7.08 – 7.04 (m, 1H), 6.81 – 6.77 (m, 1H), 6.76 – 6.73 (m, 1H), 6.66 – 6.61 (m, 1H), 3.55 (s, 3H), 3.50 (d, *J* = 5.6 Hz, 1H), 3.47 (d, *J* = 5.5 Hz, 1H), 2.79 (d, *J* = 13.3 Hz, 1H), 2.74 (d, *J* = 13.1 Hz, 1H), 1.32 (s, 3H).

^{13}C NMR (101 MHz, $CDCl_3$) δ 174.8, 159.4, 148.2, 139.3, 138.8, 137.8, 136.2, 134.4, 130.5 (2C), 129.0, 128.2 (2C), 127.9, 127.4, 126.5, 123.0, 121.6 (2C), 116.4, 115.4, 112.6, 55.0, 50.1, 46.9, 46.8, 19.8.

HRMS (MALDI) [M + H] calculated for $[C_{27}H_{27}N_2O_2]^+$ = 411.2067 found: 411.2083.

$[\alpha]_D^{20}$= -4.2 (c = 0.48, $CHCl_3$).

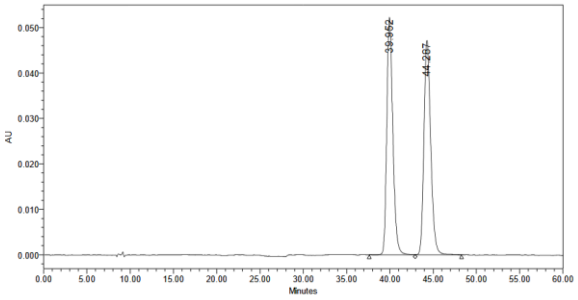

	RT	Area	% Area	Height
1	39.952	2548332	50.00	52276
2	44.287	2548800	50.00	47163

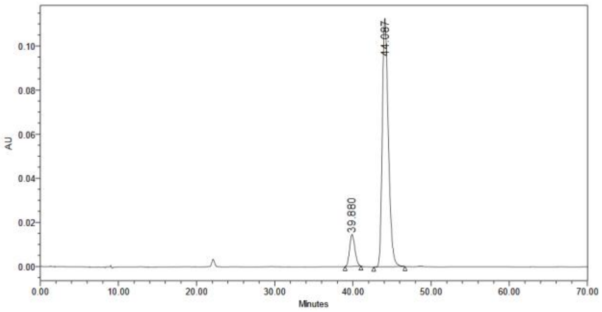

	RT	Area	% Area	Height
1	39.880	680383	10.02	14511
2	44.087	6109516	89.98	112859

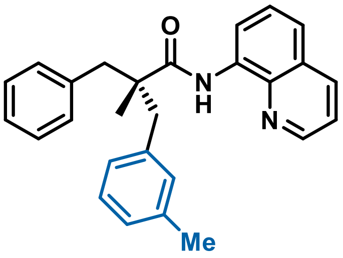

3ao

The product **3ao** was synthesized according to the general procedure using **1aa** (91.3 mg) as the substrate. The purification was performed by column chromatography (PE/DCM = 40:60) yielding the title compound as a colorless oil (73.4 mg, 62% yield). The enantiomeric excess was determined by HPLC analysis on a Daicel Chiralpak IC-5 column: 90:10 Hexane/IPA, flow rate 0.5mL/min, λ= 250.1 nm: T_RMajor = 42.95 min, T_RMinor = 36.27 min. er = 91.0:9.0.

^{1}H NMR (400 MHz, $CDCl_3$) δ 9.90 (s, 1H), 8.87 (dd, *J* = 7.6, 1.4 Hz, 1H), 8.61 (dd, *J* = 4.2, 1.7 Hz, 1H), 8.10 (dd, *J* = 8.3, 1.7 Hz, 1H), 7.60 – 7.53 (m, 1H), 7.48 (dd, *J* = 8.3, 1.4 Hz, 1H), 7.37 (dd, *J* = 8.3, 4.2 Hz, 1H), 7.24 – 7.10 (m, 6H), 7.07 – 6.98 (m, 2H), 6.93 – 6.88 (m, 1H), 3.50 (d, *J* = 13.2 Hz, 1H), 3.42 (d, *J* = 13.1 Hz, 1H), 2.78 (d, *J* = 8.7 Hz, 1H), 2.75 (d, *J* = 8.7 Hz, 1H), 2.11 (d, *J* = 0.7 Hz, 3H), 1.31 (s, 3H).

^{13}C NMR (101 MHz, $CDCl_3$) δ 174.8, 148.2, 138.8, 137.9, 137.6, 137.6, 136.2, 134.4, 131.3, 130.5 (2C), 128.1 (2C), 128.0, 127.9, 127.5, 127.5, 127.2, 126.5, 121.5, 121.5, 116.4, 50.1, 46.8, 46.6, 21.3, 19.8.

HRMS (ESI) [M + Na] calculated for $[C_{27}H_{26}N_2NaO]^+$ = 417.1937 found: 417.1932.

$[\alpha]_D^{20}$= -12.7 (c = 0.36, $CHCl_3$).

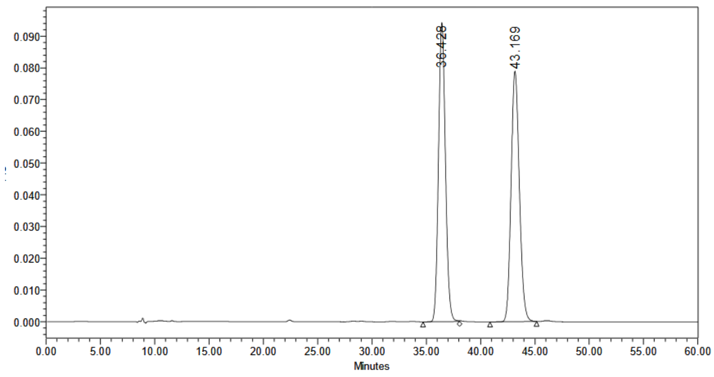

	RT	Area	% Area	Height
1	36.428	4169898	49.98	94356
2	43.169	4173942	50.02	79034

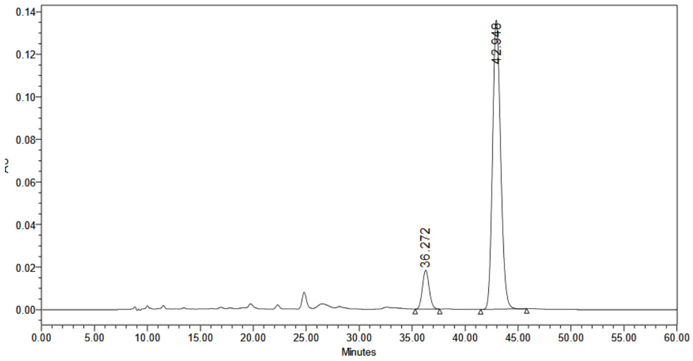

	RT	Area	% Area	Height
1	36.272	710812	8.86	17326
2	42.948	7307751	91.14	136941

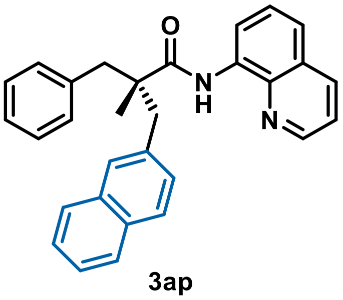

3ap

The product **3ap** was synthesized according to the general procedure using **1aa** (91.3 mg) as the substrate. The purification was performed by column chromatography (PE/DCM = 40:60) yielding the title compound as a colorless oil (54.3 mg, 42% yield). The enantiomeric excess was determined by HPLC analysis on a Daicel Chiralpak IC-5 column: 90:10 Hexane/IPA, flow rate 0.5mL/min, λ= 250.1 nm: T_RMajor = 23.07 min, T_RMinor = 25.50 min. er = 91.0:9.0.

^{1}H NMR (400 MHz, $CDCl_3$) δ 9.89 (s, 1H), 8.88 (dd, *J* = 7.6, 1.3 Hz, 1H), 8.47 (dd, *J* = 4.2, 1.7 Hz, 1H), 8.07 (dd, *J* = 8.3, 1.7 Hz, 1H), 7.71 – 7.52 (m, 5H), 7.48 (dd, *J* = 8.3, 1.3 Hz, 1H), 7.37 – 7.32 (m, 3H), 7.30 (dd, *J* = 8.3, 4.2 Hz, 1H), 7.24 – 7.09 (m, 5H), 3.64 (d, *J* = 13.2 Hz, 1H), 3.54 (d, *J* = 13.2 Hz, 1H), 2.94 (d, *J* = 13.2 Hz, 1H), 2.85 (d, *J* = 13.3 Hz, 1H), 1.35 (s, 3H).

^{13}C NMR (101 MHz, $CDCl_3$) δ 174.8, 148.1, 138.8, 137.8, 136.1, 135.5, 134.3, 133.4, 132.3, 130.6 (2C), 129.1, 128.9, 128.2 (2C), 127.9, 127.7, 127.6, 127.6, 127.4, 126.6, 125.8, 125.4, 121.6, 121.5, 116.5, 50.2, 46.8, 46.8, 19.9.

HRMS (ESI) [M + Na] calculated for $[C_{30}H_{26}N_2NaO]^+$ = 453.1937 found: 453.1925.

$[\alpha]_D^{20}$= -2.7 (c = 0.50, $CHCl_3$).

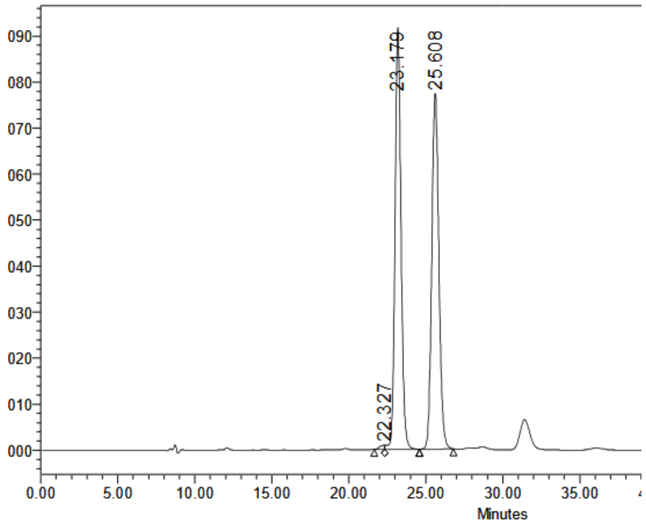

	RT	Area	% Area	Height
1	22.327	20586	0.41	908
2	23.179	2496880	49.99	91756
3	25.608	2476890	49.59	77307

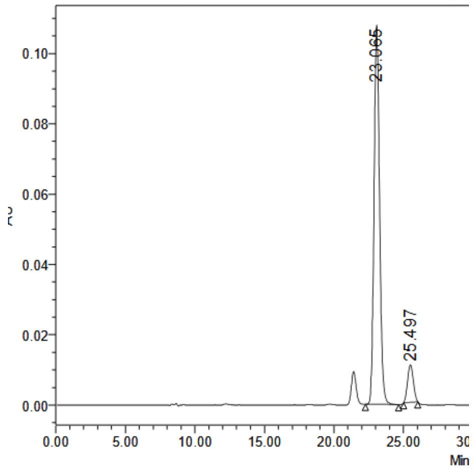

	RT	Area	% Area	Height
1	23.065	2982871	90.69	107990
2	25.497	306367	9.31	10671

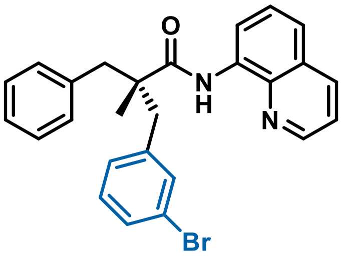

3aq

The product **3aq** was synthesized according to the general procedure using **1aa** (91.3 mg) as the substrate. The purification was performed by column chromatography (PE/DCM = 40:60) yielding the title compound as a colorless oil (85.5 mg, 62% yield). The enantiomeric excess was determined by HPLC analysis on a Daicel Chiralpak IA-5 column: 90:10 Hexane/IPA, flow rate 0.5mL/min, λ= 250.0 nm: T_RMajor = 37.49 min, T_RMinor = 41.40 min. er = 90.5:9.5.

^{1}H NMR (400 MHz, $CDCl_3$) δ 9.89 (s, 1H), 8.85 (dd, *J* = 7.6, 1.4 Hz, 1H), 8.62 (dd, *J* = 4.2, 1.7 Hz, 1H), 8.10 (dd, *J* = 8.3, 1.7 Hz, 1H), 7.59 – 7.54 (m, 1H), 7.49 (dd, *J* = 8.3, 1.4 Hz, 1H), 7.37 (dd, *J* = 8.3, 4.2 Hz, 1H), 7.35 – 7.33 (m, 1H), 7.23 – 7.16 (m, 5H), 7.15 – 7.10 (m, 2H), 7.03 – 6.97 (m, 1H), 3.48 (d, *J* = 5.1 Hz, 1H), 3.44 (d, *J* = 5.1 Hz, 1H), 2.79 (d, *J* = 13.2 Hz, 1H), 2.70 (d, *J* = 13.2 Hz, 1H), 1.32 (s, 3H).

^{13}C NMR (101 MHz, $CDCl_3$) δ 174.3, 148.2, 140.2, 138.8, 137.5, 136.2, 134.1, 133.4, 130.5 (2C), 129.7, 129.6, 129.0, 128.2 (2C), 127.9, 127.5, 126.6, 122.2, 121.7, 121.6, 116.5, 50.0, 46.7, 46.2, 19.7.

HRMS (MALDI) [M + H] calculated for $[C_{26}H_{24}BrN_2O]^+$ = 459.1067 found: 459.1088.

$[\alpha]_D^{20}$= +13.3 (c = 0.74, $CHCl_3$).

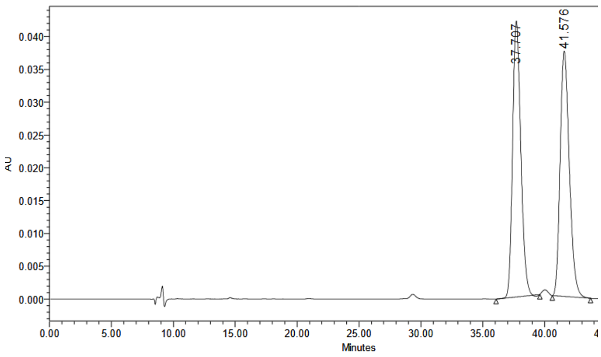

	RT	Area	% Area	Height
1	37.707	1943677	50.08	42075
2	41.576	1937221	49.92	37383

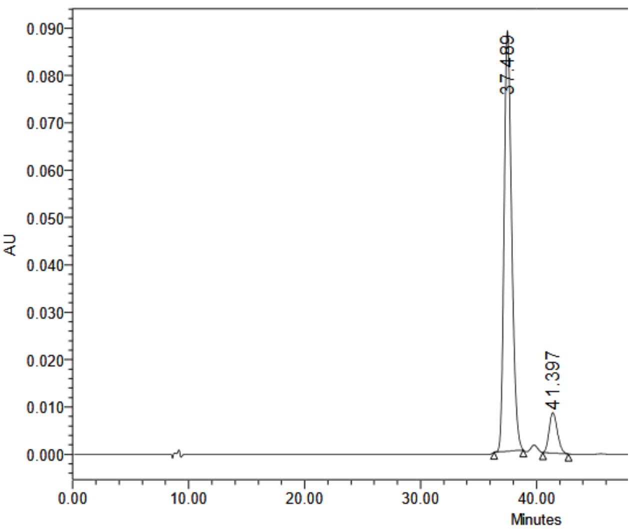

	RT	Area	% Area	Height
1	37.489	4018351	90.47	88858
2	41.397	423336	9.53	8507

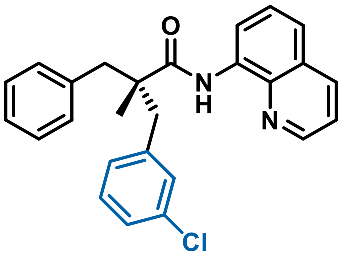

3ar

The product **3ar** was synthesized according to the general procedure using **1aa** (91.3 mg) as the substrate. The purification was performed by column chromatography (PE/DCM = 40:60) yielding the title compound as a colorless oil (88.4 mg, 71% yield). The enantiomeric excess was determined by HPLC analysis on a Daicel Chiralpak ID-5 column: 95:05 Hexane/IPA, flow rate 0.5mL/min, λ= 250.1 nm: T_RMajor = 34.51 min, T_RMinor = 37.22 min. er = 91.0:9.0.

^{1}H NMR (400 MHz, $CDCl_3$) δ 9.89 (s, 1H), 8.85 (dd, *J* = 7.6, 1.4 Hz, 1H), 8.62 (dd, *J* = 4.2, 1.7 Hz, 1H), 8.10 (dd, *J* = 8.3, 1.7 Hz, 1H), 7.59 – 7.53 (m, 1H), 7.51 – 7.47 (m, 1H), 7.37 (dd, *J* = 8.3, 4.2 Hz, 1H), 7.23 – 7.12 (m, 6H), 7.11 – 7.04 (m, 3H), 3.47 (d, *J* = 13.2 Hz, 2H), 2.79 (d, *J* = 13.2 Hz, 1H), 2.71 (d, *J* = 13.2 Hz, 1H), 1.32 (s, 3H).

^{13}C NMR (101 MHz, $CDCl_3$) δ 174.3, 148.2, 139.9, 138.8, 137.5, 136.2, 134.2, 133.8, 130.5, 130.5 (2C), 129.4, 128.6, 128.2 (2C), 127.9, 127.5, 126.7, 126.6, 121.7, 121.6, 116.5, 50.0, 46.7, 46.2, 19.8.

HRMS (MALDI) [M + H] calculated for $[C_{26}H_{24}ClN_2O]^+$ = 415.1572 found: 415.1576.

$[\alpha]_D^{20}$= +10.0 (c = 0.98, $CHCl_3$).

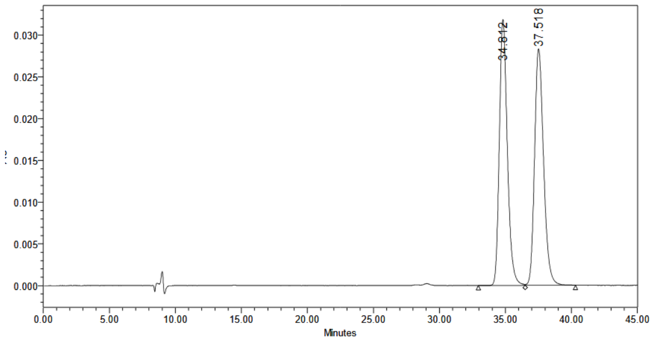

	RT	Area	% Area	Height
1	34.812	1339780	49.93	31873
2	37.518	1343645	50.07	28339

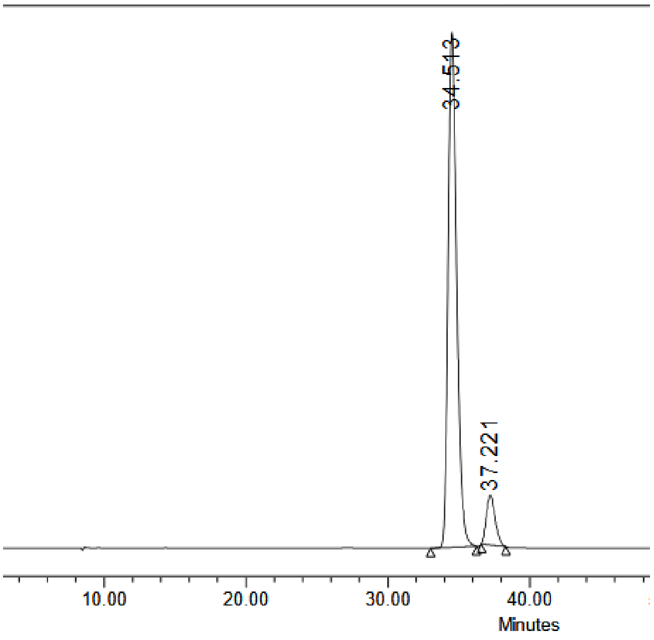

	RT	Area	% Area	Height
1	34.513	6763256	90.97	161028
2	37.221	671216	9.03	15578

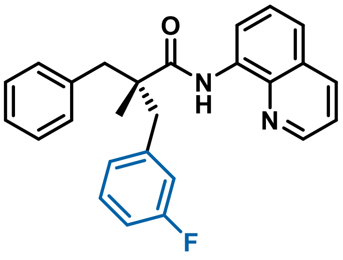

3as

The product **3as** was synthesized according to the general procedure using **1aa** (91.3 mg) as the substrate. The purification was performed by column chromatography (PE/DCM = 40:60) yielding the title compound as a colorless oil (52.6 mg, 44% yield). The enantiomeric excess was determined by HPLC analysis on a Daicel Chiralpak IC-5 column: 95:05 Hexane/IPA, flow rate 0.5mL/min, λ= 250.0 nm: T_RMajor = 17.34 min, T_RMinor = 18.62 min. er = 92.0:8.0.

^{1}H NMR (400 MHz, $CDCl_3$) δ 9.90 (s, 1H), 8.84 (dd, *J* = 7.6, 1.4 Hz, 1H), 8.63 (dd, *J* = 4.3, 1.7 Hz, 1H), 8.12 (dd, *J* = 8.3, 1.7 Hz, 1H), 7.59 – 7.53 (m, 1H), 7.50 (dd, *J* = 8.3, 1.4 Hz, 1H), 7.39 (dd, *J* = 8.3, 4.2 Hz, 1H), 7.22 – 7.06 (m, 6H), 6.99 – 6.90 (m, 2H), 6.79 (tdd, *J* = 8.5, 2.6, 1.0 Hz, 1H), 3.48 (dd, *J* = 15.0, 13.2 Hz, 2H), 2.76 (dd, *J* = 24.0, 13.2 Hz, 2H), 1.32 (s, 3H).

^{13}C NMR (101 MHz, $CDCl_3$) δ 174.4, 162.6 (d, *J* = 245.0 Hz), 148.2, 140.4 (d, *J* = 7.2 Hz), 138.8, 137.5, 136.3, 134.2, 130.5 (2C), 129.5 (d, *J* = 8.4 Hz), 128.2 (2C), 128.0, 127.5, 126.6, 126.2 (d, *J* = 2.8 Hz), 121.7, 121.6, 117.3 (d, *J* = 21.0 Hz), 116.6, 113.4 (d, *J* = 21.0 Hz), 50.1, 46.9, 46.3 (d, *J* = 1.7 Hz), 19.8.

^{19}F NMR (376 MHz, $CDCl_3$) δ -114.0 (ddd, J = 10.1, 8.7, 6.1 Hz).

HRMS (ESI) [M + Na] calculated for $[C_{26}H_{23}FN_2NaO]^+$ = 421.1692 found: 421.1687.

$[\alpha]_D^{20}$= -4.7 (c = 0.36, $CHCl_3$).

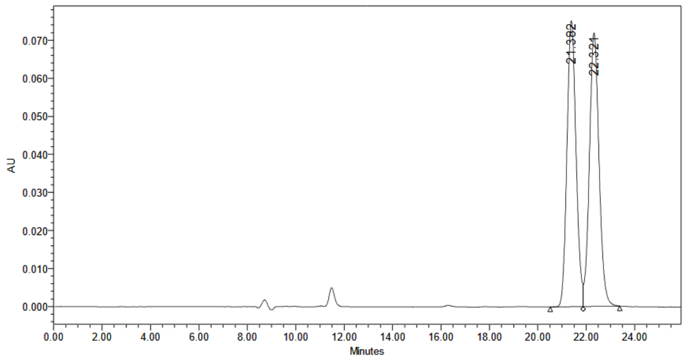

	RT	Area	% Area	Height
1	21.382	1954563	49.56	75201
2	22.321	1989466	50.44	71912

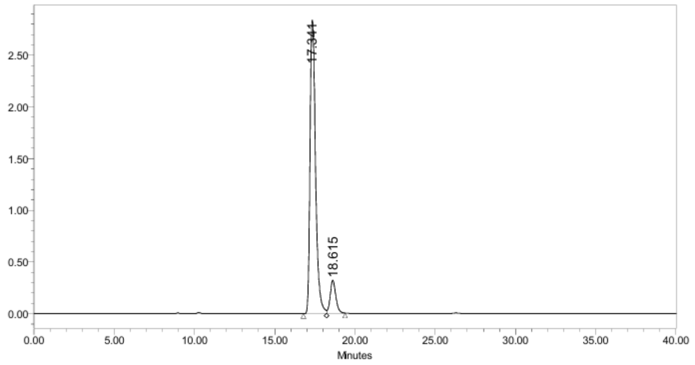

	RT	Area	% Area	Height
1	17.341	67492571	91.96	2838432
2	18.616	5897246	8.04	284998

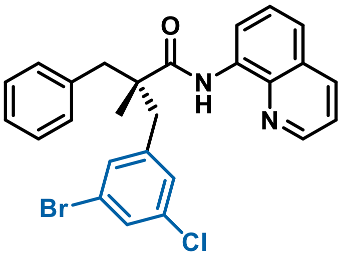

3at

The product **3at** was synthesized according to the general procedure using **1aa** (91.3 mg) as the substrate. The purification was performed by column chromatography (PE/DCM = 40:60) yielding the title compound as a colorless oil (81.5 mg, 55% yield). The enantiomeric excess

was determined by HPLC analysis on a Daicel Chiralpak ID-5 column: 90:10 Hexane/IPA, flow rate 0.5mL/min, λ= 250.0 nm: T_RMajor = 31.78 min, T_RMinor = 36.38 min. er = 91.0:9.0.

^{1}H NMR (400 MHz, $CDCl_3$) δ 9.87 (s, 1H), 8.82 (dd, *J* = 7.5, 1.4 Hz, 1H), 8.63 (dd, *J* = 4.2, 1.7 Hz, 1H), 8.11 (dd, *J* = 8.3, 1.6 Hz, 1H), 7.59 – 7.54 (m, 1H), 7.53 – 7.48 (m, 1H), 7.39 (dd, *J* = 8.2, 4.2 Hz, 1H), 7.24 – 7.16 (m, 6H), 7.16 – 7.11 (m, 2H), 3.45 (d, *J* = 11.6 Hz, 1H), 3.42 (d, *J* = 11.3 Hz, 1H), 2.79 (d, *J* = 13.2 Hz, 1H), 2.64 (d, *J* = 13.2 Hz, 1H), 1.33 (s, 3H).

^{13}C NMR (101 MHz, $CDCl_3$) δ 173.9, 148.2, 141.5, 138.8, 137.2, 136.3, 134.7, 133.9, 131.7, 130.5 (2C), 129.5, 129.3, 128.3 (2C), 128.0, 127.5, 126.7, 122.3, 121.9, 121.6, 116.7, 50.0, 46.6, 45.9, 19.8.

HRMS (MALDI) [M + H] calculated for $[C_{26}H_{23}BrClN_2O]^+$ = 493.0677 found: 493.0681.

$[\alpha]_D^{20}$= +7.6 (c = 0.85, $CHCl_3$).

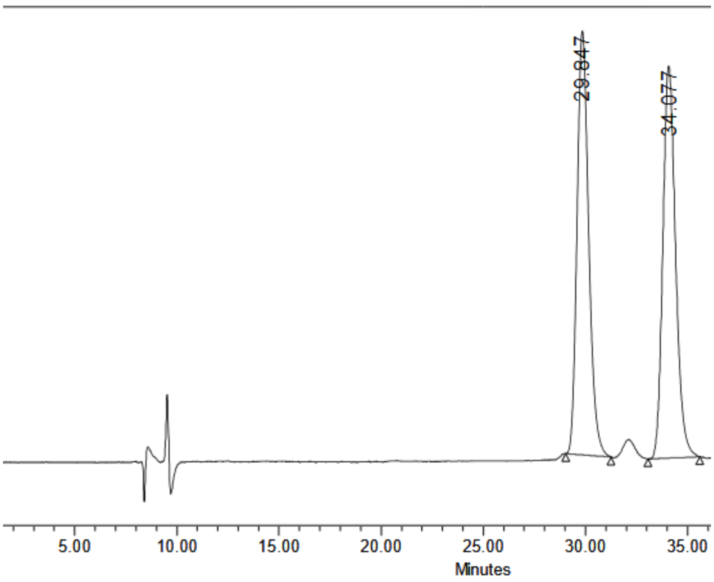

	RT	Area	% Area	Height
1	29.847	346424	49.55	8815
2	34.077	352683	50.45	8156

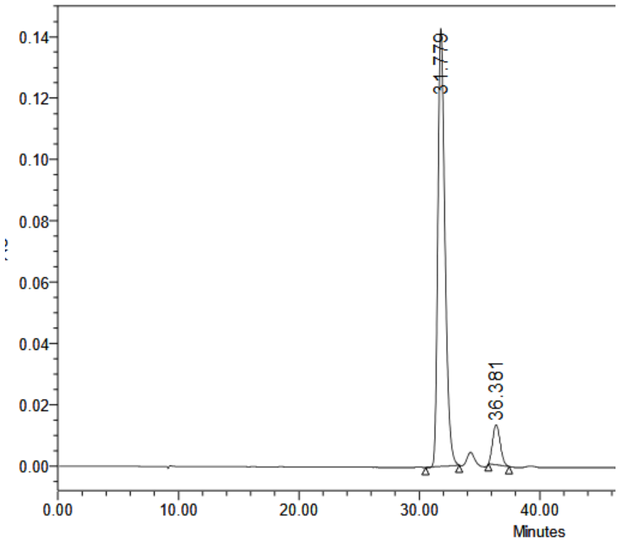

	RT	Area	% Area	Height
1	31.779	5886029	91.20	142937
2	36.381	567781	8.80	13036

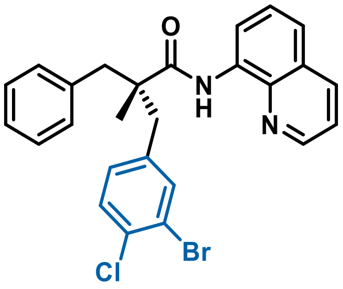

3au

The product **3au** was synthesized according to the general procedure using **1aa** (91.3 mg) as the substrate. The purification was performed by column chromatography (PE/DCM = 40:60) yielding the title compound as a colorless oil (102.8 mg, 69% yield). The enantiomeric excess was determined by HPLC analysis on a Daicel Chiralpak IC-5 column: 90:10 Hexane/IPA, flow rate 0.5mL/min, λ= 250.0 nm: T_RMajor = 17.14 min, T_RMinor = 18.51 min. er = 92.0:8.0.

^{1}H NMR (400 MHz, $CDCl_3$) δ 9.86 (s, 1H), 8.81 (dd, *J* = 7.5, 1.5 Hz, 1H), 8.63 (dd, *J* = 4.2, 1.7 Hz, 1H), 8.11 (dd, *J* = 8.3, 1.7 Hz, 1H), 7.56 (dd, *J* = 8.3, 7.5 Hz, 1H), 7.50 (dd, *J* = 8.3, 1.5 Hz, 1H), 7.39 (dd, *J* = 8.3, 4.2 Hz, 1H), 7.33 (d, *J* = 8.3 Hz, 1H), 7.28 (d, *J* = 2.1 Hz, 1H), 7.24 – 7.10 (m, 5H), 6.95 (dd, *J* = 8.3, 2.1 Hz, 1H), 3.46 (d, *J* = 7.1 Hz, 1H), 3.42 (d, *J* = 7.1 Hz, 1H), 2.80 (d, *J* = 13.3 Hz, 1H), 2.64 (d, *J* = 13.3 Hz, 1H), 1.32 (s, 3H).

^{13}C NMR (101 MHz, $CDCl_3$) δ 174.0, 148.3, 138.9, 138.8, 137.3, 136.3, 134.0, 134.0, 133.3, 132.2, 130.5 (2C), 130.0, 128.2 (2C), 128.0, 127.4, 126.7, 121.9, 121.7, 120.4, 116.6, 50.0, 46.8, 45.6, 19.7.

HRMS (ESI) [M + Na] calculated for $[C_{26}H_{22}BrClN_2NaO]^+$ = 515.0496 found: 515.0496.

$[\alpha]_D^{20}$= +1.9 (c = 0.68, $CHCl_3$).

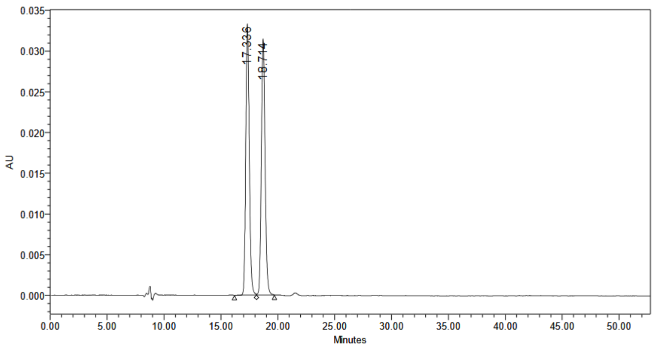

	RT	Area	% Area	Height
1	17.336	720614	50.11	33345
2	18.714	717534	49.89	31472

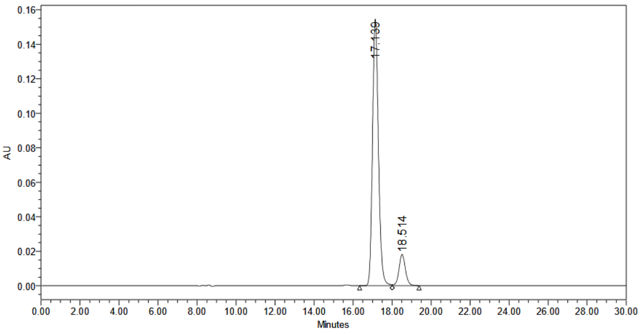

	RT	Area	% Area	Height
1	17.139	3127281	92.35	153050
2	18.517	259114	7.65	14486

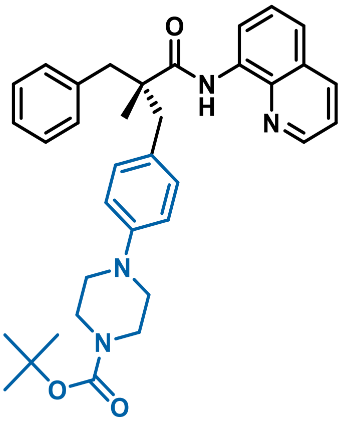

3av

The product **3av** was synthesized according to the general procedure using **1aa** (91.3 mg) as the substrate. The purification was performed by column chromatography (PE/DCM = 40:60) yielding the title compound as a colorless oil (74.6 mg, 44% yield). The enantiomeric excess was determined by HPLC analysis on a Daicel Chiralpak ID-5 column: 80:20 Hexane/IPA, flow rate 0.5mL/min, λ= 250.0 nm: T_RMajor = 70.80 min, T_RMinor = 57.00 min. er = 91.0:9.0.

^{1}H NMR (400 MHz, $CDCl_3$) δ 9.90 (s, 1H), 8.86 (dd, *J* = 7.6, 1.4 Hz, 1H), 8.62 (dd, *J* = 4.2, 1.7 Hz, 1H), 8.10 (dd, *J* = 8.3, 1.7 Hz, 1H), 7.55 (t, *J* = 7.9 Hz, 1H), 7.48 (dd, *J* = 8.3, 1.4 Hz, 1H), 7.37 (dd, *J* = 8.3, 4.2 Hz, 1H), 7.23 – 7.06 (m, 7H), 6.76 – 6.69 (m, 2H), 3.53 – 3.47 (m, 4H), 3.44 (d, *J* = 13.2 Hz, 1H), 3.39 (d, *J* = 13.4 Hz, 1H), 3.03 – 2.93 (m, 4H), 2.76 (d, *J* = 13.2 Hz, 1H), 2.71 (d, *J* = 13.4 Hz, 1H), 1.46 (s, 9H), 1.29 (s, 3H).

^{13}C NMR (101 MHz, $CDCl_3$) δ 174.9, 154.8, 149.8, 148.2, 138.8, 137.9, 136.2, 134.4, 131.2 (2C), 130.5 (2C), 129.5, 128.1 (2C), 127.9, 127.5, 126.5, 121.5, 121.5, 116.4 (3C), 80.0, 50.2, 49.5 (2C), 46.6, 46.0, 43.6 (2C), 28.5 (3C), 19.8.

HRMS (ESI) [M + Na] calculated for $[C_{35}H_{40}N_4NaO_3]^+$ = 587.2992 found: 587.2994.

$[\alpha]_D^{20}$= +3.5 (c = 0.68, $CHCl_3$).

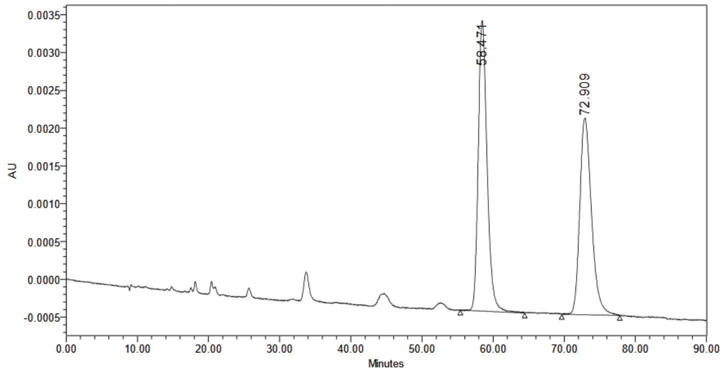

	RT	Area	% Area	Height
1	58.471	332777	52.65	3845
2	72.909	299297	47.35	2603

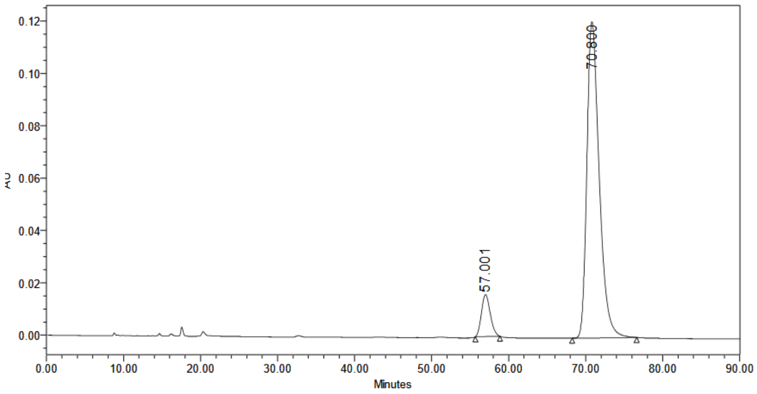

	RT	Area	% Area	Height
1	57.001	1278281	8.67	16020
2	70.800	13462495	91.33	120919

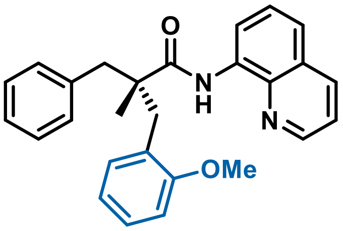

3aw

The product **3aw** was synthesized according to the general procedure using **1aa** (91.3 mg) as the substrate. The purification was performed by column chromatography (PE/DCM = 40:60) yielding the title compound as a colorless oil (92.4 mg, 75% yield). The enantiomeric excess was determined by HPLC analysis on a Daicel Chiralpak ID-5 column: 90:10 Hexane/IPA, flow rate 0.5mL/min, λ= 250.0 nm: T_RMajor = 38.02 min, T_RMinor = 35.79 min. er = 90.5:9.5.

^{1}H NMR (400 MHz, $CDCl_3$) δ 9.94 (s, 1H), 8.89 (dd, *J* = 7.6, 1.3 Hz, 1H), 8.62 (dd, *J* = 4.2, 1.7 Hz, 1H), 8.10 (dd, *J* = 8.3, 1.6 Hz, 1H), 7.58 – 7.52 (m, 1H), 7.49 – 7.45 (m, 1H), 7.36 (dd, *J* = 8.3, 4.2 Hz, 1H), 7.24 – 7.12 (m, 5H), 7.11 – 7.06 (m, 2H), 6.79 – 6.72 (m, 2H), 3.66 (s, 3H), 3.57 (d, *J* = 13.3 Hz, 1H), 3.30 (d, *J* = 13.3 Hz, 1H), 3.15 (d, *J* = 13.2 Hz, 1H), 2.75 (d, *J* = 13.2 Hz, 1H), 1.29 (s, 3H).

^{13}C NMR (101 MHz, $CDCl_3$) δ 175.1, 158.1, 148.1, 138.8, 138.3, 136.2, 134.6, 132.1, 130.5 (2C), 128.0 (2C), 127.9, 127.8, 127.5, 126.3, 126.3 121.5, 121.3, 120.3, 116.3, 110.3, 55.2, 50.3, 46.3, 39.9, 19.6.

HRMS (MALDI) [M + H] calculated for $[C_{27}H_{27}N_2O_2]^+$ = 411.2067 found: 411.2070.

$[\alpha]_D^{20}$= +1.5 (c = 0.68, $CHCl_3$).

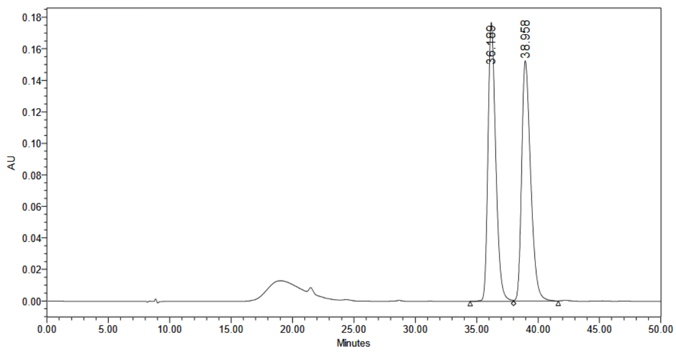

	RT	Area	% Area	Height
1	36.189	7798193	50.02	177028
2	38.958	7792305	49.98	152504

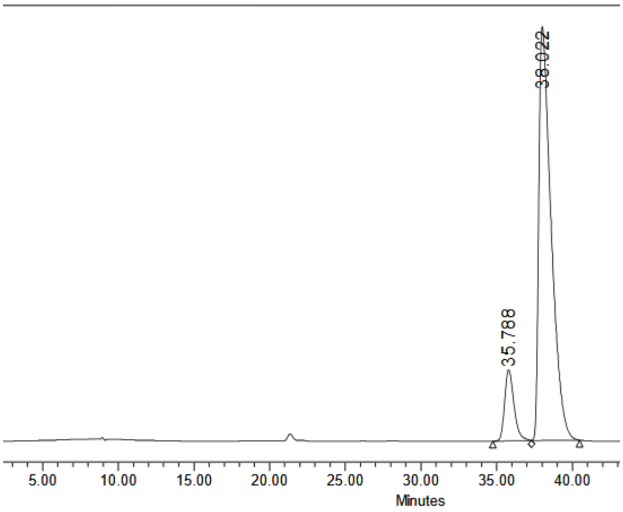

	RT	Area	% Area	Height
1	35.785	3554249	9.54	94361
2	38.022	33702898	90.46	594534

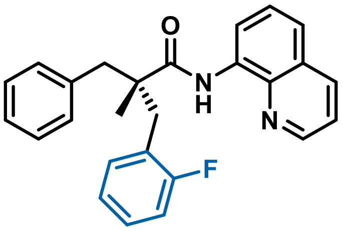

3ax

The product **3ax** was synthesized according to the general procedure using **1aa** (91.3 mg) as the substrate. The purification was performed by column chromatography (PE/DCM = 40:60) yielding the title compound as a colorless oil (56.2 mg, 47% yield). The enantiomeric excess was determined by HPLC analysis on a Daicel Chiralpak IC-5 column: 90:10 Hexane/IPA, flow rate 0.5mL/min, λ= 250.1 nm: T_RMajor = 17.86 min, T_RMinor = 19.41 min. er = 90.0:10.0.

^{1}H NMR (400 MHz, $CDCl_3$) δ 9.93 (s, 1H), 8.87 (dd, *J* = 7.7, 1.3 Hz, 1H), 8.61 (dd, *J* = 4.2, 1.7 Hz, 1H), 8.10 (dd, *J* = 8.3, 1.7 Hz, 1H), 7.59 – 7.53 (m, 1H), 7.48 (dd, *J* = 8.3, 1.4 Hz, 1H), 7.36 (dd, *J* = 8.2, 4.2 Hz, 1H), 7.28 – 7.23 (m, 1H), 7.22 – 7.05 (m, 6H), 7.02 – 6.86 (m, 2H), 3.54 (d, *J* = 13.2 Hz, 1H), 3.34 (d, *J* = 13.4 Hz, 1H), 3.09 (dd, *J* = 13.4, 1.1 Hz, 1H), 2.77 (d, *J* = 13.2 Hz, 1H), 1.34 (s, 3H).

^{13}C NMR (101 MHz, $CDCl_3$) δ 174.5, 161.6 (d, *J* = 244.9 Hz), 148.2, 138.8, 137.7, 136.2, 134.3, 132.6 (d, *J* = 4.4 Hz), 130.4 (2C), 128.3 (d, *J* = 8.0 Hz), 128.1 (2C), 127.9, 127.5, 126.5, 124.9 (d, *J* = 15.7 Hz), 124.0 (d, *J* = 3.6 Hz), 121.6, 121.6, 116.4, 115.2 (d, *J* = 23.1 Hz), 50.4, 46.5, 38.9 (d, *J* = 1.5 Hz), 19.3.

HRMS (ESI) [M + Na] calculated for $[C_{26}H_{23}FN_2NaO]^+$ = 421.1686 found: 421.1668.
$[\alpha]_D^{20}$= -11.7 (c = 0.40, $CHCl_3$).

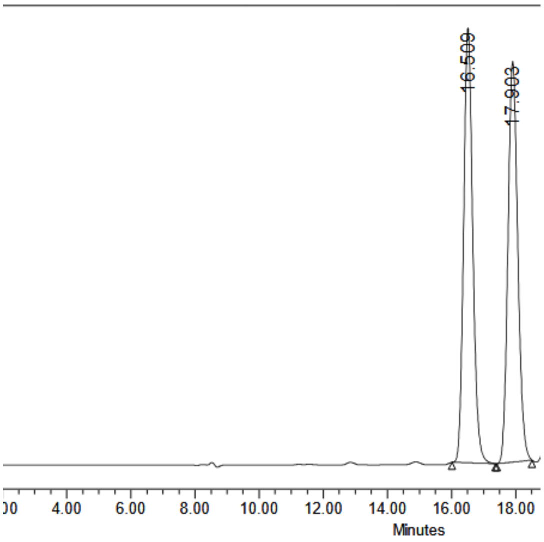

	RT	Area	% Area	Height
1	16.509	2753019	50.19	139551
2	17.903	2731993	49.81	128659

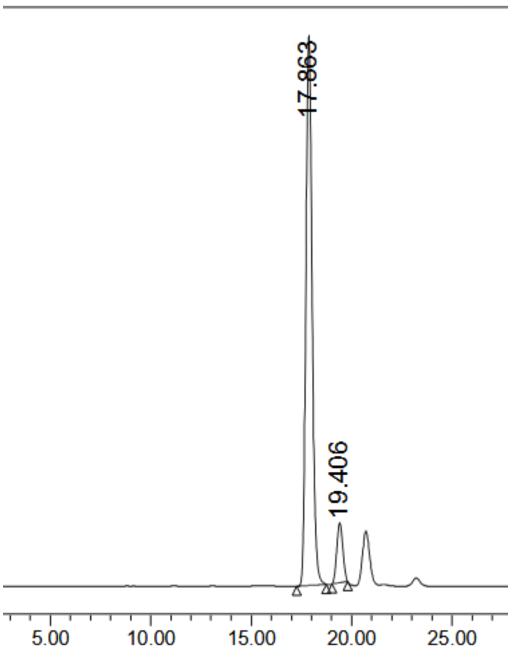

	RT	Area	% Area	Height
1	17.863	5723775	90.34	267085
2	19.406	611935	9.66	29057

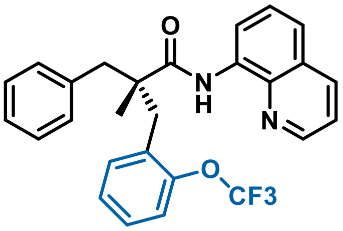

3ay

The product **3ay** was synthesized according to the general procedure using **1aa** (91.3 mg) as the substrate. The purification was performed by column chromatography (PE/DCM = 40:60) yielding the title compound as a colorless oil (105.5 mg, 75% yield). The enantiomeric excess was determined by HPLC analysis on a Daicel Chiralpak IC-5 column: 98:02 Hexane/IPA, flow rate 0.5mL/min, λ= 250.0 nm: T_RMajor = 17.06 min, T_RMinor = 18.63 min. er = 82.5:17.5.

^{1}H NMR (400 MHz, $CDCl_3$) δ 9.94 (s, 1H), 8.87 (dd, *J* = 7.6, 1.4 Hz, 1H), 8.61 (dd, *J* = 4.2, 1.7 Hz, 1H), 8.10 (dd, *J* = 8.3, 1.7 Hz, 1H), 7.59 – 7.53 (m, 1H), 7.51 – 7.47 (m, 1H), 7.36 (dd, *J* = 8.2, 4.2 Hz, 1H), 7.33 (dd, *J* = 7.7, 1.7 Hz, 1H), 7.22 – 7.12 (m, 6H), 7.12 – 7.07 (m, 1H), 7.01 (td, *J* = 7.4, 1.4 Hz, 1H), 3.53 (d, *J* = 13.1 Hz, 1H), 3.40 (d, *J* = 13.5 Hz, 1H), 3.12 (d, *J* = 13.6 Hz, 1H), 2.77 (d, *J* = 13.2 Hz, 1H), 1.30 (s, 3H).

^{13}C NMR (101 MHz, $CDCl_3$) δ 174.4, 148.3 (q, *J* = 1.5 Hz), 148.2, 138.8, 137.5, 136.2, 134.3, 132.6, 130.5 (3C), 128.1 (2C), 128.0, 127.9, 127.5, 126.6, 126.5, 122.0, 121.7, 120.7 (q, *J* = 257.4 Hz), 119.9 (q, *J* = 1.7 Hz), 116.4, 50.5, 46.9, 39.4, 19.2.

^{19}F NMR (376 MHz, $CDCl_3$) δ -56.7.

HRMS (MALDI) [M + H] calculated for $[C_{27}H_{24}F_3N_2O_2]^+$ = 465.1784 found: 465.1769.

$[\alpha]_D^{20}$= +1.9 (c = 0.67, $CHCl_3$).

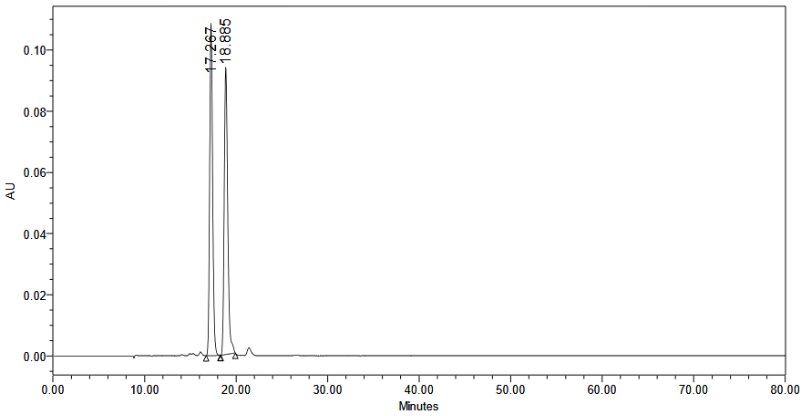

	RT	Area	% Area	Height
1	17.267	2476451	49.75	108658
2	18.885	2501778	50.25	93990

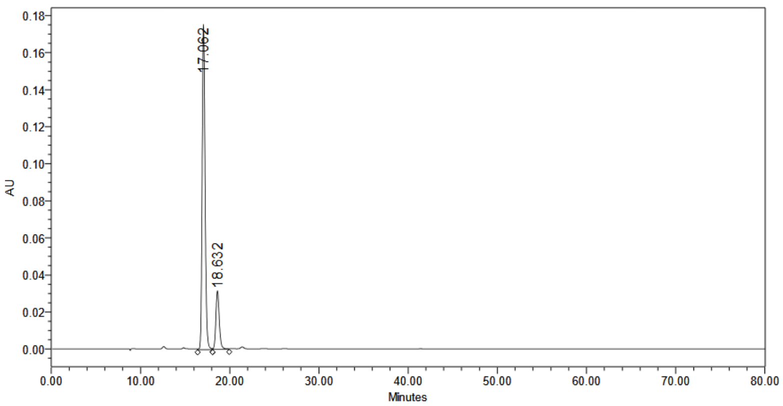

	RT	Area	% Area	Height
1	17.062	4020812	82.58	175629
2	18.632	848440	17.42	31688

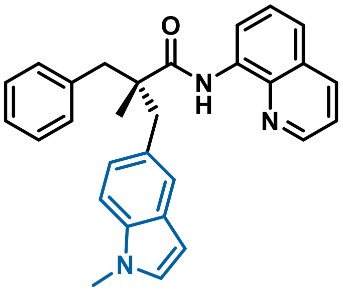

3az

The product **3az** was synthesized according to the general procedure using **1aa** (91.3 mg) as the substrate. The purification was performed by column chromatography (PE/DCM = 40:60) yielding the title compound as a white solid (84.5 mg, 65% yield). The enantiomeric excess was determined by HPLC analysis on a Daicel Chiralpak ID-5 column: 90:10 Hexane/IPA, flow rate 0.5mL/min, λ= 250.0 nm: T_RMajor = 85.16 min, T_RMinor = 67.26 min. er = 92.0:8.0.

^{1}H NMR (400 MHz, $CDCl_3$) δ 9.83 (s, 1H), 8.85 – 8.78 (m, 1H), 8.46 (dd, *J* = 4.2, 1.7 Hz, 1H), 8.04 – 7.96 (m, 1H), 7.53 – 7.44 (m, 1H), 7.42 – 7.35 (m, 2H), 7.25 (dd, *J* = 8.3, 4.2 Hz, 1H), 7.15 – 7.11 (m, 2H), 7.10 – 7.01 (m, 3H), 7.01 – 6.99 (m, 2H), 6.86 (d, *J* = 3.1 Hz, 1H), 6.26 (d, *J* = 3.1 Hz, 1H), 3.59 (s, 3H), 3.48 (d, *J* = 13.4 Hz, 1H), 3.45 (d, *J* = 13.2 Hz, 1H), 2.85 (d, *J* = 13.4 Hz, 1H), 2.73 (d, *J* = 13.2 Hz, 1H), 1.25 (s, 3H).

^{13}C NMR (101 MHz, $CDCl_3$) δ 175.3, 148.0, 138.7, 138.1, 136.2, 135.8, 134.5, 130.5 (2C), 128.8, 128.4, 128.4, 128.1 (2C), 127.9, 127.5, 126.4, 124.4, 122.6, 121.4, 121.4, 116.6, 108.8, 100.7, 50.4, 46.9, 46.5, 32.9, 19.9.

HRMS (MALDI) [M + H] calculated for $[C_{29}H_{28}N_3O]^+$ = 434.2227 found: 434.2249.

$[\alpha]_D^{20}$= +34.0 (c = 0.67, $CHCl_3$).

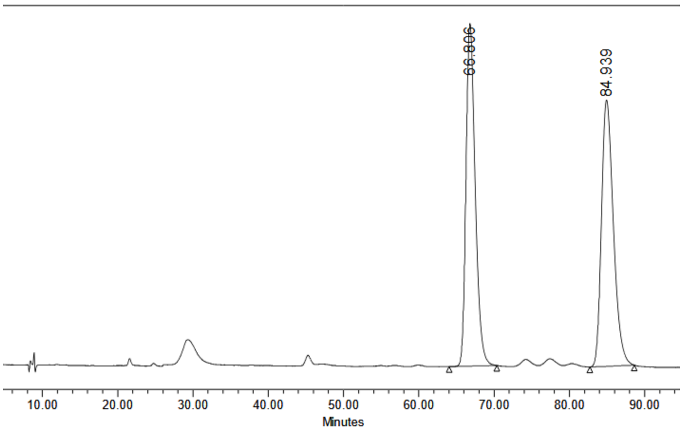

	RT	Area	% Area	Height
1	66.806	1707345	50.16	20008
2	84.939	1696395	49.84	15549

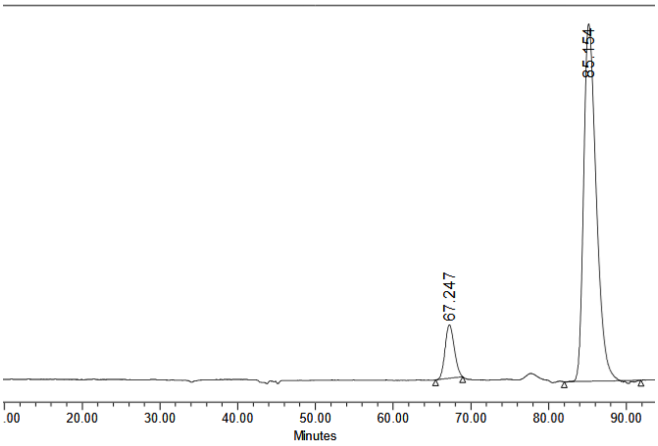

	RT	Area	% Area	Height
1	67.258	277791	7.92	3758
2	85.155	3228132	92.08	28623

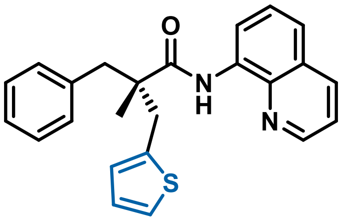

3ba

The product **3ba** was synthesized according to the general procedure using **1aa** (91.3 mg) as the substrate. The purification was performed by column chromatography (PE/DCM = 40:60) yielding the title compound as a white solid (51.0 mg, 44% yield). The enantiomeric excess was determined by HPLC analysis on a Daicel Chiralpak IA-5 column: 90:10 Hexane/IPA, flow rate 0.5mL/min, λ= 250.0 nm: T_RMajor = 37.87 min, T_RMinor = 35.11 min. er = 88.0:12.0.

^{1}H NMR (400 MHz, $CDCl_3$) δ 10.00 (s, 1H), 8.88 (dd, *J* = 7.6, 1.4 Hz, 1H), 8.66 (dd, *J* = 4.2, 1.7 Hz, 1H), 8.12 (dd, *J* = 8.3, 1.7 Hz, 1H), 7.60 – 7.54 (m, 1H), 7.50 (dd, *J* = 8.3, 1.4 Hz, 1H), 7.39 (dd, *J* = 8.3, 4.2 Hz, 1H), 7.23 – 7.08 (m, 5H), 7.07 – 7.03 (m, 1H), 6.87 – 6.81 (m, 2H), 3.67 (d, *J* = 14.4 Hz, 1H), 3.39 (d, *J* = 13.2 Hz, 1H), 2.99 (d, *J* = 14.5 Hz, 1H), 2.86 (d, *J* = 13.2 Hz, 1H), 1.38 (s, 3H).
^{13}C NMR (101 MHz, $CDCl_3$) δ 174.5, 148.2, 139.7, 138.8, 137.4, 136.3, 134.4, 130.5 (2C), 128.2 (2C), 128.0, 127.6, 127.3, 126.7, 126.6, 124.4, 121.6, 121.6, 116.6., 50.1, 46.6, 40.3, 20.2.
HRMS (ESI) [M + Na] calculated for $[C_{24}H_{22}N_2NaOS]^+$ = 409.1345 found: 409.1335.
$[\alpha]_D^{20}$= -8.7 (c = 0.26, $CHCl_3$).

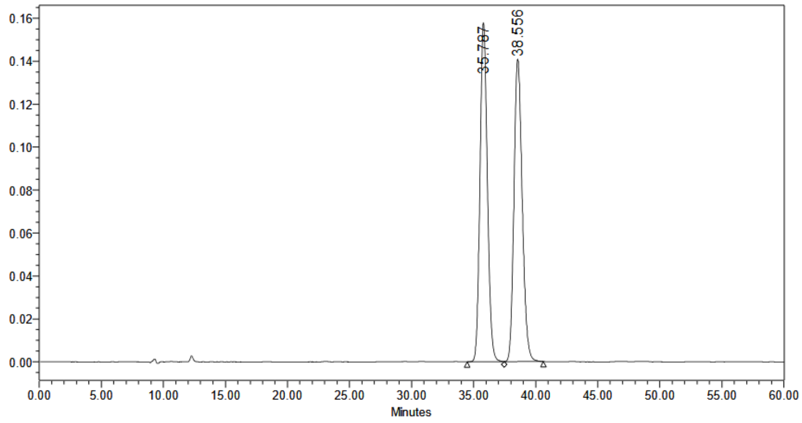

	RT	Area	% Area	Height
1	35.787	6532833	50.09	157965
2	38.556	6509514	49.91	140816

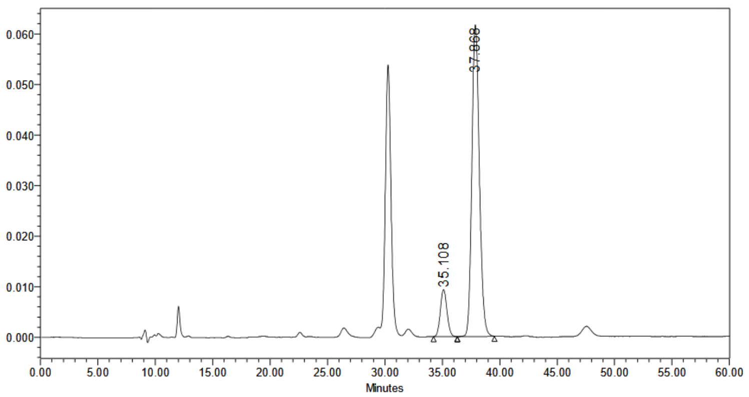

	RT	Area	% Area	Height
1	35.108	374077	11.79	9327
2	37.868	2797817	88.21	61690

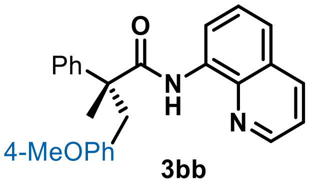

3bb

The product **3bb** was synthesized according to the general procedure using **1bb** (87.1 mg) as the substrate. The purification was performed by column chromatography (PE/DCM = 40:60) yielding the title compound as a white solid (75 mg, 63% yield). The enantiomeric excess was determined by HPLC analysis on a Daicel Chiralpak ID-5 column: 80:20 Hexane/IPA, flow rate 0.5mL/min, λ= 250.0 nm: T_RMajor = 39.81 min, T_RMinor = 31.01 min. er = 87.0:13.0.

^{1}H NMR (400 MHz, $CDCl_3$) δ 9.90 (s, 1H), 8.80 (dd, *J* = 7.6, 1.4 Hz, 1H), 8.58 (dd, *J* = 4.2, 1.7 Hz, 1H), 8.09 (dd, *J* = 8.3, 1.7 Hz, 1H), 7.56 – 7.50 (m, 1H), 7.48 – 7.42 (m, 3H), 7.40 – 7.28 (m, 4H), 6.83 – 6.77 (m, 2H), 6.69 – 6.64 (m, 2H), 3.73 (s, 3H), 3.57 (d, *J* = 13.6 Hz, 1H), 3.35 (d, *J* = 13.5 Hz, 1H), 1.68 (s, 3H).

^{13}C NMR (101 MHz, $CDCl_3$) δ 175.3, 158.2, 148.3, 143.3, 138.8, 136.2, 134.8, 131.7 (2C), 129.7, 128.7 (2C), 128.0, 127.5 (2C), 127.4, 127.2, 121.6, 121.4, 116.2, 113.2 (2C), 55.2, 53.1, 44.5, 23.0.

HRMS (MALDI) [M + H] calculated for $[C_{26}H_{25}N_2O_2]^+$ = 397.1911 found: 397.1907.

$[\alpha]_D^{20}$= + 3.7 (c = 0.55, $CHCl_3$).

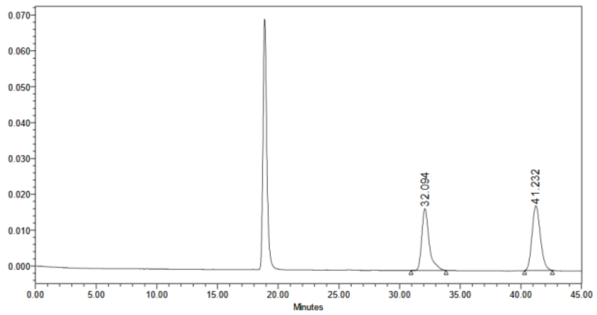

	RT	Area	% Area	Height
1	32.094	726152	45.79	17229
2	41.232	859682	54.21	18023

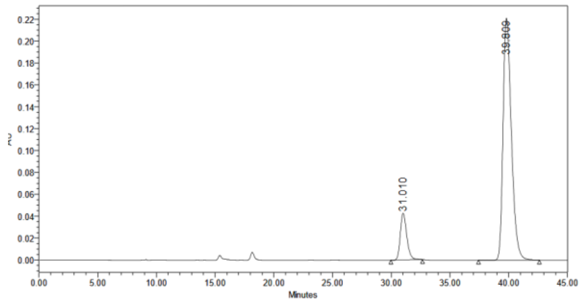

	RT	Area	% Area	Height
1	31.010	1626428	13.05	42672
2	39.809	10840820	86.95	221104

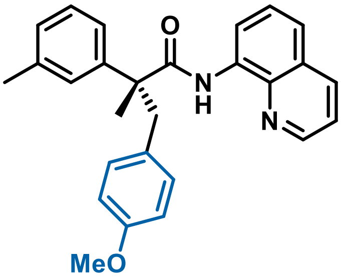

3bc

The product **3bc** was synthesized according to the general procedure using **1bc** (91.3 mg) as the substrate. The purification was performed by column chromatography (PE/DCM = 40:60) yielding the title compound as a white solid (69 mg, 56% yield). The enantiomeric excess was determined by HPLC analysis on a Daicel Chiralpak IC-5 column: 90:10 Hexane/IPA, flow rate 0.5mL/min, λ= 250.0 nm: T_RMajor = 42.95 min, T_RMinor = 36.27 min. er = 92.0:8.0.

^{1}H NMR (400 MHz, $CDCl_3$) δ 9.94 (s, 1H), 8.82 (dd, *J* = 7.7, 1.3 Hz, 1H), 8.61 (dd, *J* = 4.2, 1.7 Hz, 1H), 8.09 (dd, *J* = 8.3, 1.7 Hz, 1H), 7.57 – 7.51 (m, 1H), 7.48 – 7.44 (m, 1H), 7.35 (dd, *J* = 8.3, 4.2 Hz, 1H), 7.30 – 7.26 (m, 3H), 7.15 – 7.10 (m, 1H), 6.88 – 6.82 (m, 2H), 6.71 – 6.66 (m, 2H), 3.73 (s, 3H), 3.59 (d, *J* = 13.5 Hz, 1H), 3.34 (d, *J* = 13.5 Hz, 1H), 2.36 (s, 3H), 1.68 (s, 3H).

^{13}C NMR (101 MHz, $CDCl_3$) δ 175.3, 158.2, 148.2, 143.3, 138.8, 138.2,136.2, 134.8, 131.7 (2C), 129.8, 128.5, 128.1, 128.0, 128.0, 127.4, 124.3, 121.5, 121.3, 116.2, 113.2 (2C), 55.2, 52.9, 44.5, 23.0, 21.8.

HRMS (MALDI) [M + H] calculated for $[C_{28}H_{29}N_2O_2]^+$ = 411.2067 found: 411.2078.

$[\alpha]_D^{20}$= +10.2 (c = 1.55, $CHCl_3$).

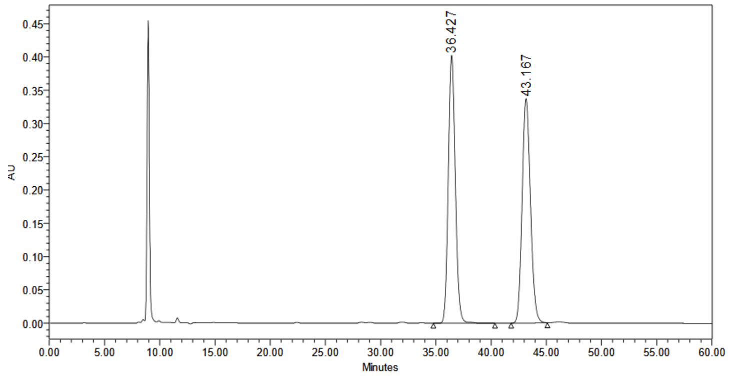

	RT	Area	% Area	Height
1	36.427	17894567	50.12	402880
2	43.167	17809943	49.88	337312

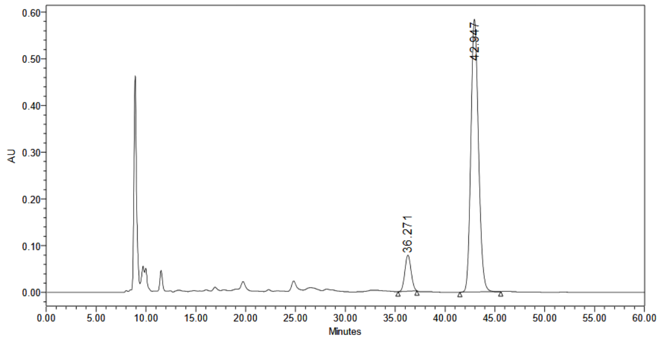

	RT	Area	% Area	Height
1	36.273	4733834	8.36	119622
2	42.947	51900905	91.64	974899

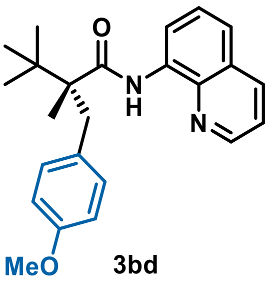

3bd

The product **3bd** was synthesized according to the general procedure using 2,2,3,3-tetramethyl-N-(quinolin-8-yl)butanamide (81,1 mg) as the substrate (*R*)-L_6 as ligand. The purification was performed by column chromatography (PE/EA = 90:10) yielding the title compound as a white solid (58.7 mg, 52% yield). The enantiomeric excess was determined by

HPLC analysis on a Daicel Chiralpak IC-5 column: 97:3 Hexane/IPA, flow rate 0.5mL/min, λ= 250.0 nm: T_RMajor = 26.16 min, T_RMinor = 29.96 min. er = 95.5:4.5.

^{1}H NMR (400 MHz, $CDCl_3$) δ 10.13 (s, 1H), 8.89 (dd, *J* = 7.6, 1.4 Hz, 1H), 8.73 (dd, *J* = 4.2, 1.7 Hz, 1H), 8.12 (dd, *J* = 8.3, 1.7 Hz, 1H), 7.55 (t, *J* = 7.9 Hz, 1H), 7.48 (dd, *J* = 8.3, 1.4 Hz, 1H), 7.40 (dd, *J* = 8.3, 4.2 Hz, 1H), 7.13 (d, *J* = 8.7 Hz, 2H), 6.70 (d, *J* = 8.7 Hz, 2H), 3.74 (d, *J* = 13.2 Hz, 1H), 3.69 (s, 3H), 2.46 (d, *J* = 13.2 Hz, 1H), 1.26 (s, 3H), 1.14 (s, 9H).

^{13}C NMR (101 MHz, $CDCl_3$) δ 174.7, 158.0, 148.3, 139.0, 136.3, 134.6, 131.5, 131.4 (2C), 128.1, 127.6, 121.6, 121.3, 116.2, 113.5 (2C), 55.2, 53.9, 38.8, 36.9, 26.6 (3C), 18.1.

HRMS (ESI) [M + H] calculated for $[C_{24}H_{29}N_2O_2]^+$ = 377.2223 found: 377.2210.

$[\alpha]_D^{20}$= -15.7 (c = 0.55, $CHCl_3$).

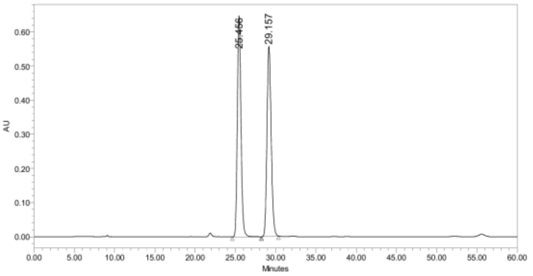

	RT	Area	% Area	Height
1	25.456	20333983	50.25	648021
2	29.157	20135352	49.75	557504

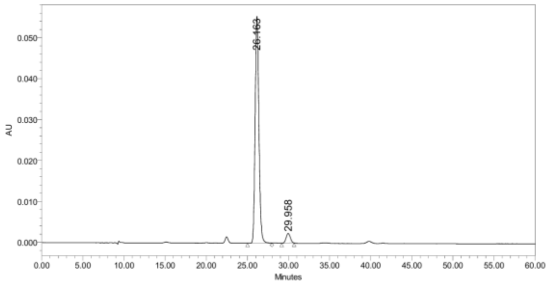

	RT	Area	% Area	Height
1	26.163	1763176	95.47	55407
2	29.958	83596	4.53	2369

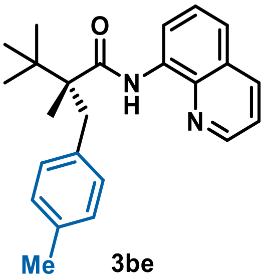

3be

The product **3be** was synthesized according to the general procedure 2,2,3,3-tetramethyl-N-(quinolin-8-yl)butanamide (81,1 mg) as the substrate (*R*)-L_6 as ligand. The purification was performed by column chromatography (PE/DCM = 40:60) yielding the title compound as a white solid (44.3 mg, 41% yield). The enantiomeric excess was determined by HPLC analysis

on a Daicel Chiralpak IC-5 column: 97:3 Hexane/IPA, flow rate 0.5mL/min, λ= 250.0 nm: T_RMajor = 18.22 min, T_RMinor = 22.45 min. er = 94.0:6.0.

^{1}H NMR (400 MHz, $CDCl_3$) δ 10.13 (s, 1H), 8.90 (dd, *J* = 7.6, 1.4 Hz, 1H), 8.72 (dd, *J* = 4.2, 1.7 Hz, 1H), 8.12 (dd, *J* = 8.3, 1.7 Hz, 1H), 7.58 – 7.52 (m, 1H), 7.48 (dd, *J* = 8.3, 1.4 Hz, 1H), 7.40 (dd, *J* = 8.2, 4.2 Hz, 1H), 7.15 – 7.07 (m, 2H), 6.99 – 6.94 (m, 2H), 3.76 (d, *J* = 13.1 Hz, 1H), 2.48 (d, *J* = 13.1 Hz, 1H), 2.22 (s, 3H), 1.25 (s, 3H), 1.14 (s, 9H).

^{13}C NMR (101 MHz, $CDCl_3$) δ 174.7, 148.3, 139.0, 136.4, 136.3, 135.5, 134.7, 130.3 (2C), 128.9 (2C), 128.1, 127.6, 121.6, 121.2, 116.2, 53.8, 39.3, 36.9, 29.9, 26.6 (3C), 21.1, 18.1.

HRMS (ESI) [M + H] calculated for $[C_{24}H_{29}N_2O]^+$ = 361.2274 found: 361.2272.

$[\alpha]_D^{20}$= -8.7 (c = 0.51, $CHCl_3$).

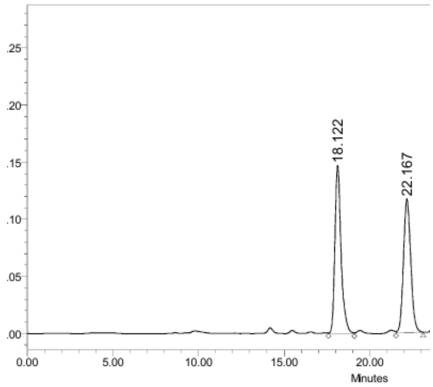

	RT	Area	% Area	Height
1	18.122	3682701	51.50	147056
2	22.167	3468532	48.50	117228

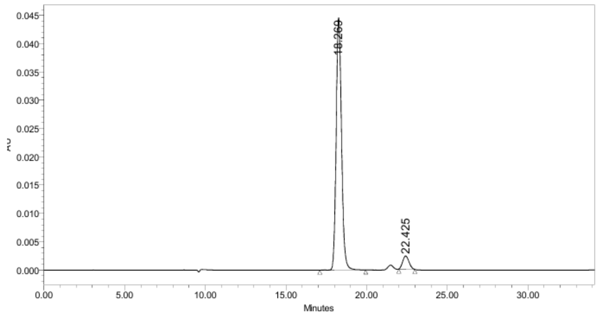

	RT	Area	% Area	Height
1	18.269	1026716	94.19	44538
2	22.425	63353	5.81	2349

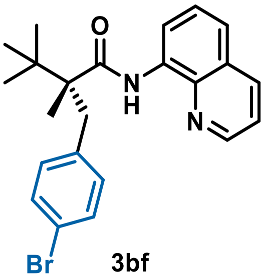

3bf

The product **3bf** was synthesized according to the general procedure using 2,2,3,3-tetramethyl-N-(quinolin-8-yl)butanamide (81,1 mg) as the substrate and (*R*)-L_6 as ligand. The purification was performed by column chromatography (PE/DCM = 40:60) yielding the title compound as a white solid (57.4 mg, 45% yield). The enantiomeric excess was determined by HPLC analysis on a Daicel Chiralpak IA-5 column: 95:05 Hexane/IPA, flow rate 0.5mL/min, λ= 250.0 nm: T_RMajor =14.36 min, T_RMinor = 15.44 min. er = 95.0:5.0.

^{1}H NMR (400 MHz, $CDCl_3$) δ 10.12 (s, 1H), 8.87 (dd, *J* = 7.5, 1.5 Hz, 1H), 8.73 (dd, *J* = 4.3, 1.7 Hz, 1H), 8.13 (dd, *J* = 8.3, 1.7 Hz, 1H), 7.55 (dd, *J* = 8.3, 7.4 Hz, 1H), 7.49 (dd, *J* = 8.3, 1.5 Hz, 1H), 7.41 (dd, *J* = 8.3, 4.2 Hz, 1H), 7.28 (d, *J* = 8.5 Hz, 2H), 7.09 (d, *J* = 8.5 Hz, 2H), 3.76 (d, *J* = 13.0 Hz, 1H), 2.46 (d, *J* = 13.0 Hz, 1H), 1.25 (s, 3H), 1.14 (s, 9H).

^{13}C NMR (101 MHz, $CDCl_3$) δ 174.3, 148.4, 139.0, 138.6, 136.4, 134.5, 132.2 (2C), 131.2 (2C), 128.1, 127.6, 121.7, 121.5, 120.1, 116.2, 53.8, 39.1, 37.0, 26.6 (3C), 18.1.

HRMS (ESI) [M + H] calculated for $[C_{23}H_{26}BrN_2O]^+$ = 425.1223 found: 425.1219.

$[\alpha]_D^{20}$= -6.7 (c = 0.41, $CHCl_3$).

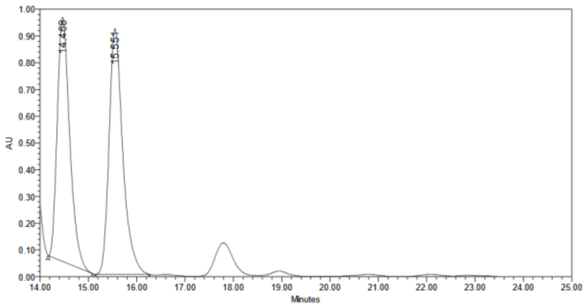

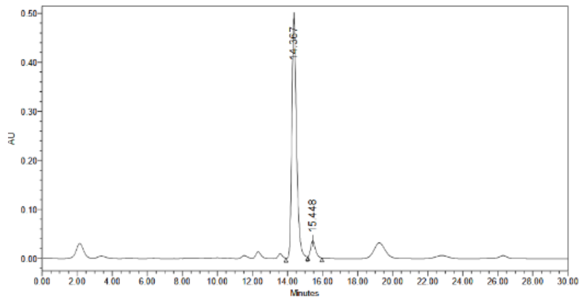

	RT	Area	% Area	Height
1	14.468	16406502	47.45	912804
2	15.551	18168857	52.55	918957

	RT	Area	% Area	Height	Peak Width (sec)
1	14.367	9009163	95.49	487736	34.27
2	15.444	425170	4.51	28543	34.27

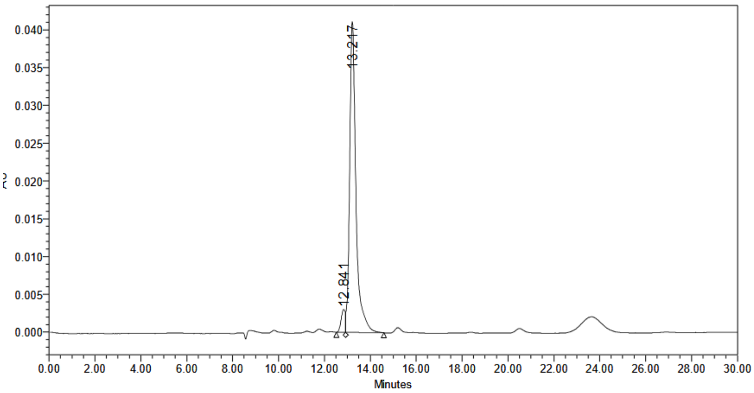

	RT	Area	% Area	Height
1	12.841	37665	4.59	3024
2	13.217	782896	95.41	41138

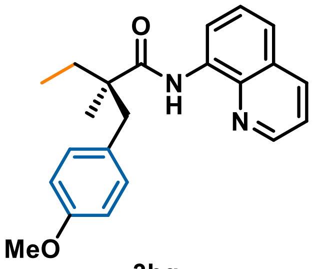

3bg

The product **3bg** was synthesized according to the general procedure using 1bg (72.7 mg) as the substrate. The purification was performed by column chromatography (PE/DCM = 40:60) yielding the title compound as a white solid (74.2 mg, 71% yield). The enantiomeric excess was determined by HPLC analysis on a Daicel Chiralpak ID-5 column: 80:20 Hexane/IPA, flow rate 0.5mL/min, λ= 250.0 nm: T_RMajor = 22.78 min, T_RMinor = 21.73 min. er = 80.0:20.0.

^{1}H NMR (400 MHz, $CDCl_3$) δ 10.10 (s, 1H), 8.84 (dd, *J* = 7.5, 1.5 Hz, 1H), 8.74 (dd, *J* = 4.2, 1.7 Hz, 1H), 8.14 (dd, *J* = 8.3, 1.7 Hz, 1H), 7.62 – 7.34 (m, 3H), 7.15 – 7.05 (m, 2H), 6.75 – 6.62 (m, 2H), 3.69 (s, 3H), 3.18 (d, *J* = 13.5 Hz, 1H), 2.76 (d, *J* = 13.5 Hz, 1H), 2.03 (dq, *J* = 14.7, 7.4 Hz, 1H), 1.77 – 1.52 (m, 2H), 1.33 (s, 3H), 0.98 (t, *J* = 7.5 Hz, 3H).

^{13}C NMR (101 MHz, $CDCl_3$) δ 175.5, 158.2, 148.3, 138.9, 136.3, 134.6, 131.3 (2C), 130.0, 128.0, 127.6, 121.6, 121.4, 116.4, 113.5 (2C), 55.2, 49.2, 45.4, 32.8, 20.3, 9.3.

HRMS (ESI) [M + Na] calculated for $[C_{22}H_{42}N_2NaO_2]^+$ = 371.1735 found: 371.1712.

$[\alpha]_D^{20}$= -3.7 (c = 0.51, $CHCl_3$).

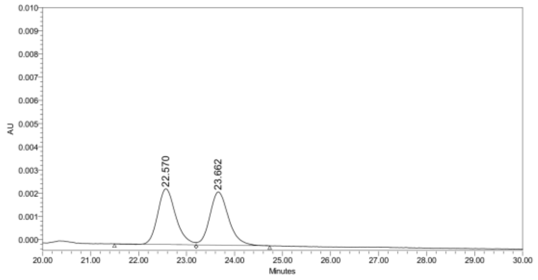

	RT	Area	% Area	Height
1	22.570	62850	50.17	2399
2	23.662	62431	49.83	2295

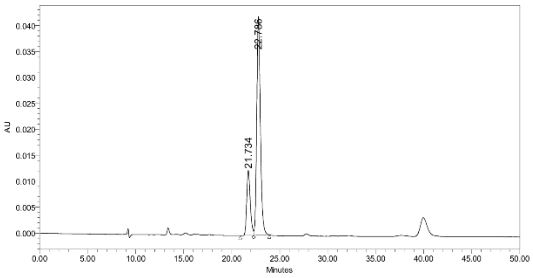

	RT	Area	% Area	Height	Peak Width (sec)
1	21.734	266630	19.91	11693	48.95
2	22.786	1072763	80.09	41578	48.95

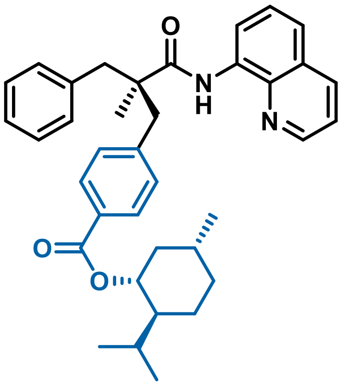

3bh

The product **3bh** was synthesized according to the general procedure using 1aa (91.3 mg) as the substrate. The purification was performed by column chromatography (PE/DCM = 40:60) yielding the title compound as a white solid (114.6 mg, 68% yield). The enantiomeric excess was determined by HPLC analysis on a Daicel Chiralpak IA-5 column: 90:10 Hexane/IPA, flow rate 0.5mL/min, λ= 250.0 nm: T_RMajor = 22.56 min, T_RMinor = 29.32 min. d.r = 10.0:1.0.

^{1}H NMR (400 MHz, $CDCl_3$) δ 9.82 (s, 1H), 8.78 (dd, *J* = 7.5, 1.5 Hz, 1H), 8.53 (dd, *J* = 4.2, 1.7 Hz, 1H), 8.04 (dd, *J* = 8.3, 1.7 Hz, 1H), 7.75 (d, *J* = 8.3 Hz, 2H), 7.54 – 7.47 (m, 1H), 7.43 (dd, *J* = 8.3, 1.5 Hz, 1H), 7.29 (dd, *J* = 8.3, 4.2 Hz, 1H), 7.21 – 7.17 (m, 2H), 7.15 – 7.02 (m, 5H), 4.77 (td, *J* = 10.8, 4.4 Hz, 1H), 3.48 (d, *J* = 13.0 Hz, 1H), 3.40 (d, *J* = 13.2 Hz, 1H), 2.75 (d, *J* = 8.6 Hz, 1H), 2.71 (d, *J* = 8.5 Hz, 1H), 1.97 (d, *J* = 12.1 Hz, 2H), 1.83 (pd, *J* = 6.9, 2.6 Hz, 1H), 1.66 – 1.58 (m, 2H), 1.46 – 1.36 (m, 2H), 1.18 (s, 3H), 1.04 – 0.90 (m, 2H), 0.82 (d, *J* = 3.8 Hz, 3H), 0.80 (d, *J* = 4.3 Hz, 3H), 0.67 (d, *J* = 6.9 Hz, 3H).

^{13}C NMR (101 MHz, $CDCl_3$) δ 174.3, 166.2, 148.2, 143.2, 138.8, 137.5, 136.3, 134.2, 130.5 (2C), 130.4 (2C), 129.4 (2C), 129.2, 128.2 (2C), 127.9, 127.5, 126.7, 121.7, 121.6, 116.5, 74.7, 50.1, 47.3, 47.0, 46.5, 41.1, 34.4, 31.5, 29.9, 26.5, 23.7, 22.2, 20.9, 16.6.

HRMS (ESI) [M + Na] calculated for $[C_{37}H_{42}N_2NaO_3]^+$ = 585.3087 found: 585.3078.

$[\alpha]_D^{20}$= -41.7 (c = 0.61, $CHCl_3$).

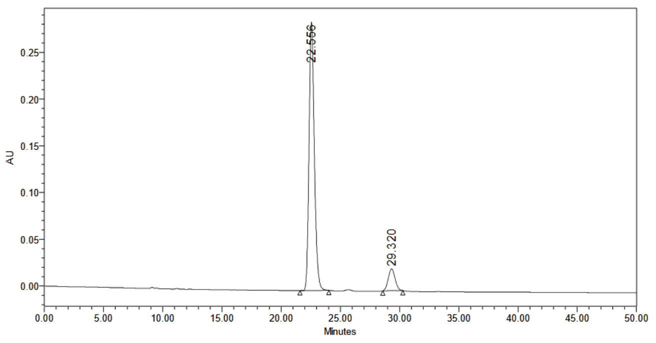

	RT	Area	% Area	Height
1	22.556	8945580	90.52	287368
2	29.320	936608	9.48	23536

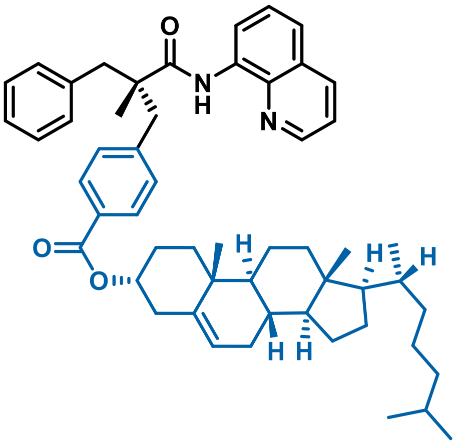

3bi

The product **3bi** was synthesized according to the general procedure using 3a (36.1 mg) as the substrate. The purification was performed by column chromatography (PE/DCM = 40:60) yielding the title compound as a white solid (145.0 mg, 61% yield). The enantiomeric excess was determined by HPLC analysis on a Daicel Chiralpak IA-5 column: 90:10 Hexane/IPA, flow rate 0.5mL/min, λ= 250.0 nm: T_RMajor = 29.09 min, T_RMinor = 34.87 min. d.r = 6.0:1.0.

^{1}H NMR (400 MHz, $CDCl_3$) δ 9.88 (s, 1H), 8.85 (dd, *J* = 7.6, 1.4 Hz, 1H), 8.60 (dd, *J* = 4.2, 1.7 Hz, 1H), 8.10 (dd, *J* = 8.3, 1.7 Hz, 1H), 7.90 – 7.76 (m, 2H), 7.62 – 7.54 (m, 1H), 7.49 (dd, *J* = 8.3, 1.5 Hz, 1H), 7.36 (dd, *J* = 8.2, 4.2 Hz, 1H), 7.28 – 7.23 (m, 2H), 7.22 – 7.09 (m, 5H), 5.44 – 5.35 (m, 1H), 4.76 (ddt, *J* = 11.9, 8.2, 4.3 Hz, 1H), 3.55 (d, *J* = 13.0 Hz, 1H), 3.47 (d, *J* = 13.2 Hz, 1H), 2.81 (d, *J* = 10.0 Hz, 1H), 2.78 (d, *J* = 9.9 Hz, 1H), 2.37 (d, *J* = 7.8 Hz, 2H), 2.05 – 1.77 (m, 5H), 1.54 – 1.06 (m, 24H), 1.02 (s, 3H), 0.91 (d, *J* = 6.5 Hz, 3H), 0.87 (d, *J* = 1.9 Hz, 3H), 0.85 (d, *J* = 1.9 Hz, 3H), 0.67 (s, 3H).

^{13}C NMR (101 MHz, $CDCl_3$) δ 174.3, 166.1, 148.2, 143.2, 139.8, 138.8, 137.5, 136.3, 134.2, 130.5 (2C), 130.4 (2C), 129.4 (2C), 129.1, 128.2 (2C), 127.9, 127.5, 126.7, 122.8, 121.7, 121.6, 116.5, 74.5, 56.8, 56.2, 50.1, 50.1, 46.9, 46.5, 42.4, 39.9, 39.6, 38.3, 37.1, 36.8, 36.3, 35.9, 32.0, 32.0, 29.8, 28.4, 28.2, 28.0, 24.4, 24.0, 23.0, 22.7, 21.2, 19.8, 19.5, 18.8.

HRMS (ESI) [M + Na] calculated for $[C_{54}H_{68}N_2NaO_3]^+$ = 815.5122 found: 815.5102.

$[\alpha]_D^{20}$= -69.7 (c = 0.31, $CHCl_3$).

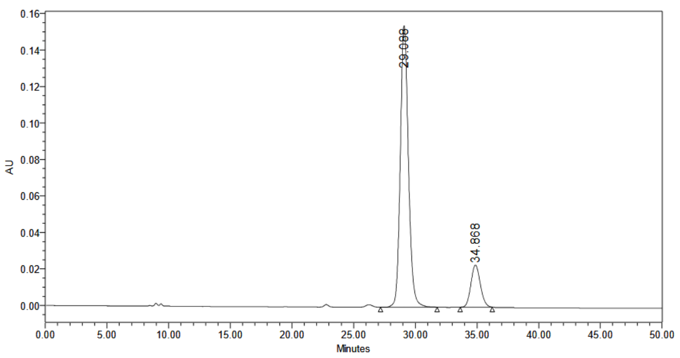

	RT	Area	% Area	Height
1	29.088	7032528	85.20	154395
2	34.868	1221786	14.80	23102

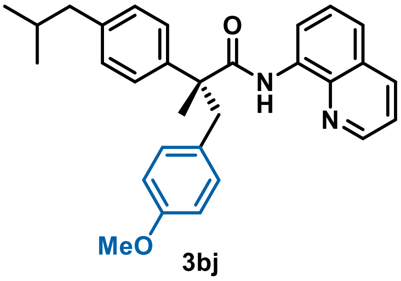

3bj

The product **3bj** was synthesized according to the general procedure using 2-(4-isobutylphenyl)-2-methyl-N-(quinolin-8-yl)propanamide (103.9 mg) as the substrate. The purification was performed by column chromatography (PE/DCM = 40:60) yielding the title compound as a white solid (61.1 mg, 45% yield). The enantiomeric excess was determined by HPLC analysis on a Daicel Chiralpak IC-5 column: 95:05 Hexane/IPA, flow rate 0.5mL/min, λ= 250.0 nm: T_RMajor = 40.64 min, T_RMinor = 46.60 min. er = 92.0:8.0.

^{1}H NMR (400 MHz, $CDCl_3$) δ 9.88 (s, 1H), 8.81 (dd, *J* = 7.6, 1.4 Hz, 1H), 8.53 (dd, *J* = 4.2, 1.7 Hz, 1H), 8.08 (dd, *J* = 8.3, 1.7 Hz, 1H), 7.56 – 7.50 (m, 1H), 7.45 (dd, *J* = 8.3, 1.4 Hz, 1H), 7.36 – 7.29 (m, 3H), 7.16 – 7.12 (m, 2H), 6.77 (m, 2H), 6.67 – 6.63 (m, 2H), 3.73 (s, 3H), 3.53 (d, *J* = 13.5 Hz, 1H), 3.34 (d, *J* = 13.5 Hz, 1H), 2.49 (d, *J* = 7.2 Hz, 2H), 1.94 – 1.81 (m, 1H), 1.65 (s, 3H), 0.92 (d, *J* = 2.1 Hz, 3H), 0.91 (d, *J* = 2.1 Hz, 3H).

^{13}C NMR (101 MHz, $CDCl_3$) δ 175.8, 158.2, 148.2, 140.7, 140.3, 138.8, 136.2, 134.9, 131.7, 129.8, 129.4, 128.0, 127.5, 127.3, 121.5, 121.3, 116.1, 113.1, 77.5, 77.4, 77.2, 76.8, 55.2, 52.8, 45.1, 44.5, 30.4, 29.9, 23.1, 22.5, 22.4.

HRMS (ESI) [M + Na] calculated for $[C_{30}H_{32}N_2NaO_2]^+$ = 475.2361 found: 475.3168.

$[\alpha]_D^{20}$= -21.7 (c = 0.36, $CHCl_3$).

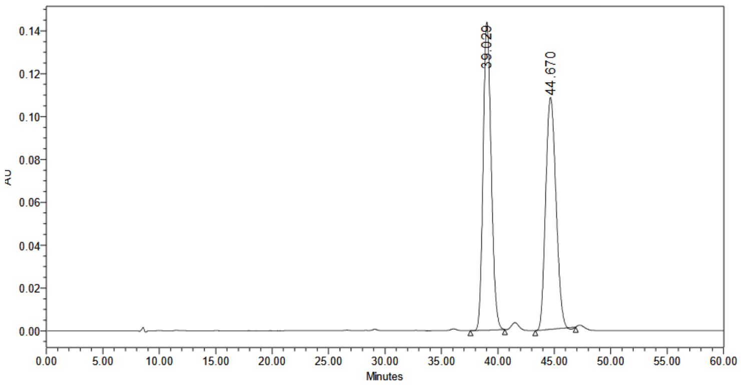

	RT	Area	% Area	Height
1	39.029	7106765	50.77	143810
2	44.670	6892470	49.23	108223

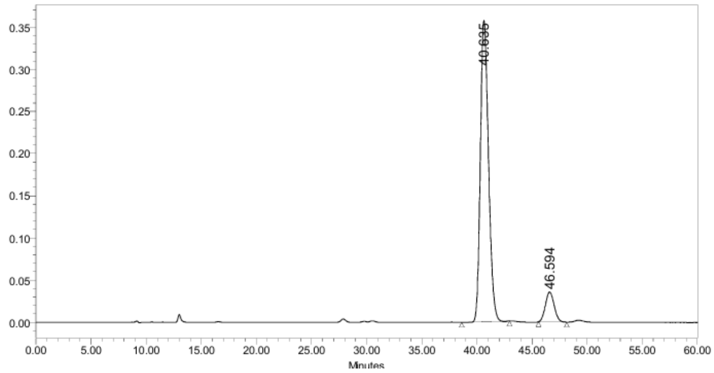

	RT	Area	% Area	Height
1	40.635	18292894	91.79	357587
2	46.601	1635157	8.21	32489

6. Gram-scale Arylation and post-transformation

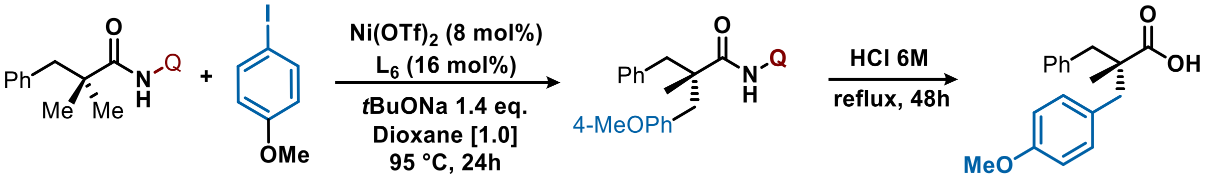

1aa
1.0g

2a
2 eq.

3aa
82%, (1.1g) 92:8 *e.r*
800mg 99:1 e.r
after recrystalization

4
81%, 99:1 *e.r*

Inside an argon-filled glovebox, an oven-dried Schlenk tube equipped with a magnetic stir bar was charged with **1aa** (1.00 g, 3.29 mmol, 1.0 equiv), **Ni(OTf)$_2$** (93.78 mg, 0.2628 mmol, 0.08 equiv), **4-methoxyphenyl iodide** (1.54 g, 6.57 mmol, 2.0 equiv), **L$_6$** (266.71 mg, 0.5256 mmol, 0.16 equiv), and **sodium *t*BuONa** (416.76 mg, 4.34 mmol, 1.32 equiv). The solids were suspended in dry, degassed 1,4-dioxane (6 mL), and the Schlenk tube was sealed, removed from the glovebox and stirred at 95°C for 24 h. After completion, the reaction mixture was quenched with water (10 mL), and extracted with ethyl acetate (3 × 15 mL). The combined organic layers were washed with brine, dried over anhydrous Na_2SO_4, filtered, and concentrated under reduced pressure. The crude residue was purified by flash column chromatography to afford the desired product as a solid (1.10 g, 2.68 mmol, 81.56% yield). Components of the reaction, including the starting **1aa,** ligand **L$_6$** and iodoanisole **2a**, were successfully recovered after purification. The recovered ligand was subjected to a second reaction to assess its purity. It exhibited the expected reactivity and enantioselectivity, confirming its integrity after one catalysis. Recrystallization was performed with a mixture of diethyl ether and pentane (20:80) at 0°C overnight. Crystals were filtered and washed with cold pentane to afford **3aa** in an enantiomerically pure form (800.0 mg, 73% yield).

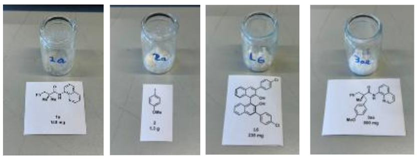

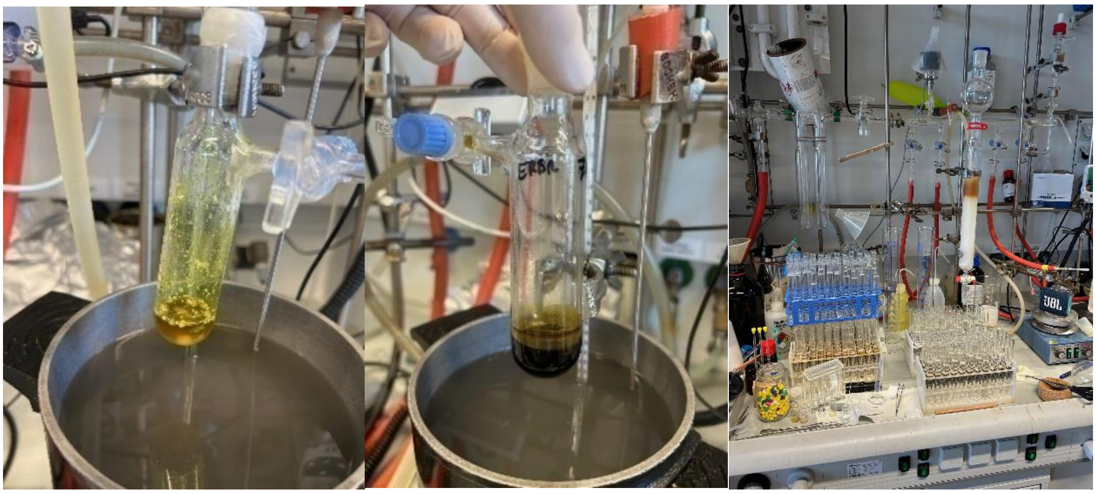

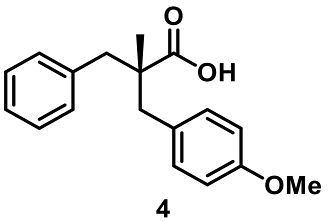

4

Compound **3aa** (100 mg, 0.27 mmol, 1.0 equiv) was dissolved in **6 M aqueous HCl** (5 mL, 1.5 mmol HCl, 5.6 equiv) in a round-bottom flask equipped with a reflux condenser. The mixture was heated at reflux (≈110°C) for **48 hours** under vigorous stirring. After cooling to room temperature, the aqueous phase was carefully **partially neutralized with saturated aqueous $NaHCO_3$ solution** until **pH ≈ 2** (monitored using pH paper). The resulting suspension was then extracted with ethyl acetate (3 × 10 mL). The combined organic layers were washed with brine (10 mL), dried over anhydrous sodium sulfate (Na_2SO_4), filtered, and concentrated under reduced pressure.

Purification of the crude product by flash chromatography (silica gel, hexane/ethyl acetate gradient) afforded the corresponding carboxylic acid **4** as a white solid in **81% yield** (67 mg). The enantiomeric excess was determined by HPLC analysis on a Daicel Chiralpak IC-5 column: 90:10 Hexane/IPA, flow rate 0.5mL/min, λ= 225.1 nm: T_RMajor = 14.76 min, T_RMinor = 13.18 min. er = 99.0:1.0.

^{1}H NMR (400 MHz, $CDCl_3$) δ 7.24 – 7.14 (m, 3H), 7.11 (dd, *J* = 7.8, 1.8 Hz, 2H), 7.05 – 6.99 (m, 2H), 6.76 – 6.72 (m, 2H), 3.71 (s, 3H), 3.15 (d, *J* = 13.3 Hz, 1H), 3.10 (d, *J* = 13.4 Hz, 1H), 2.64 (d, *J* = 11.1 Hz, 1H), 2.61 (d, *J* = 11.6 Hz, 1H), 0.95 (s, 3H).

^{13}C NMR (101 MHz, $CDCl_3$) δ 182.7, 158.5, 137.4, 131.4 (2C), 130.4 (2C), 129.3, 128.3 (2C), 126.8, 113.7 (2C), 55.3, 45.9, 45.3, 29.8, 19.7.

HRMS (ESI) [M + Na] calculated for $[C_{18}H_{20}NaO_3]^+$ = 307.1304 found: 307.1301.

Melting point = 114–118°C.

$[\alpha]_D^{20}$= -31.7 (c = 0.41, $CHCl_3$).

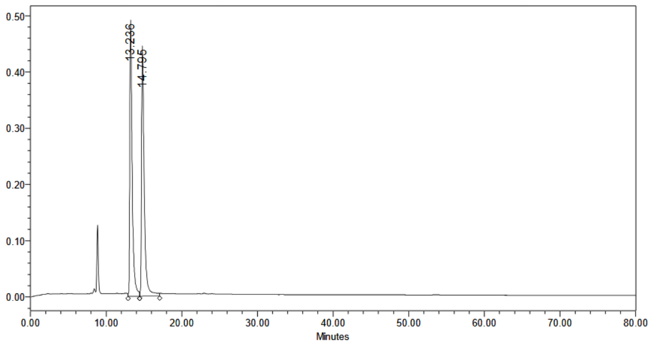

	RT	Area	% Area	Height
1	13.236	10615652	49.12	491313
2	14.795	10995231	50.88	444864

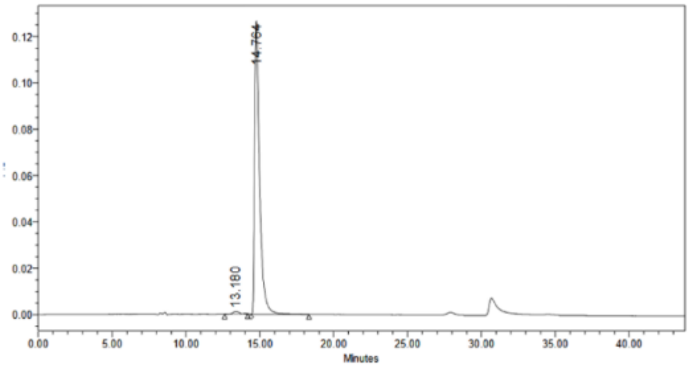

	RT	Area	% Area	Height
1	13.180	154449	0.53	13279
2	14.764	28836574	99.47	788375

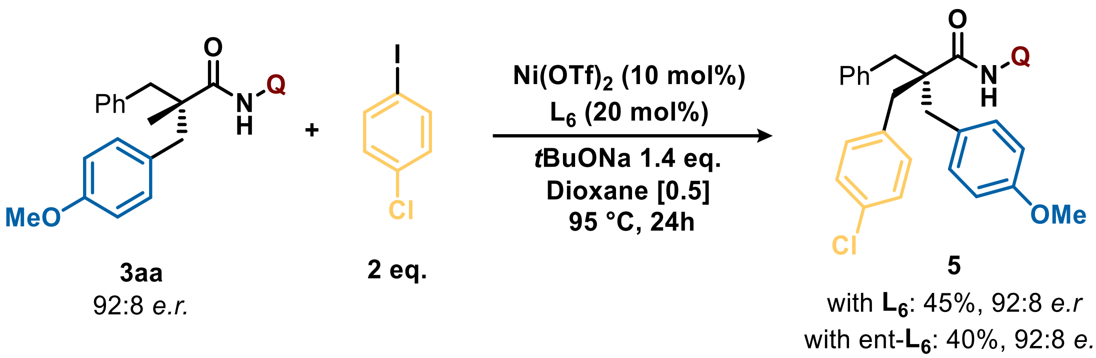

3aa
92:8 *e.r.*

2 eq.

5
with **L$_6$**: 45%, 92:8 *e.r*
with ent-**L$_6$**: 40%, 92:8 *e.r*

The product **5** was synthesized according to the general procedure using **3aa** (41.0 mg) as the substrate. The purification was performed by column chromatography (PE/DCM = 20:80) yielding the title compound as a colorless oil (23.4 mg, 45% yield). The enantiomeric excess was determined by HPLC analysis on a Daicel Chiralpak IC-5 column: 90:10 Hexane/IPA, flow rate 0.5mL/min, λ= 250.1 nm: T_RMajor = 28.17 min, T_RMinor = 29.13 min. er = 92.0:8.0.

^{1}H NMR (400 MHz, $CDCl_3$) δ 9.92 (s, 1H), 8.81 (dd, *J* = 7.6, 1.4 Hz, 1H), 8.48 (dd, *J* = 4.2, 1.7 Hz, 1H), 8.10 (dd, *J* = 8.3, 1.7 Hz, 1H), 7.63 – 7.53 (m, 1H), 7.49 (dd, *J* = 8.3, 1.4 Hz, 1H), 7.35 (dd, *J* = 8.3, 4.2 Hz, 1H), 7.22 – 7.04 (m, 11H), 6.77 – 6.63 (m, 2H), 3.69 (s, 3H), 3.21 (s, 2H), 3.16 (s, 4H).

^{13}C NMR (101 MHz, $CDCl_3$) δ 173.8, 158.4, 148.1, 138.7, 137.3, 136.1, 136.1, 134.2, 132.4, 132.0 (2C), 131.6 (2C), 130.6 (2C), 129.0, 128.3 (4C), 127.9, 127.5, 126.6, 121.6, 121.6, 116.4, 113.7 (2C), 55.2, 52.3, 41.6, 41.3, 41.1.

HRMS (ESI) [M + Na] calculated for $[C_{33}H_{29}ClN_2NaO_2]^+$ = 543.1809 found: 543.1787.

$[\alpha]_D^{20}$= -11.7 (c = 0.21, $CHCl_3$).

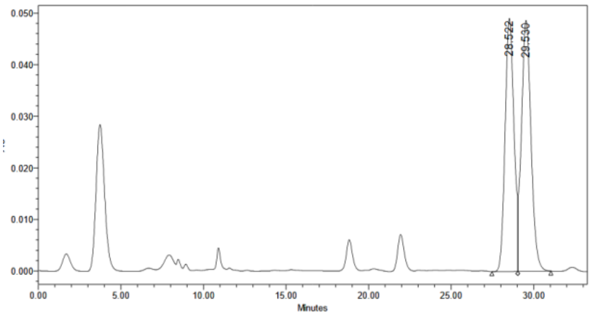

	RT	Area	% Area	Height
1	28.522	1823208	48.04	49104
2	29.530	1972017	51.96	48711

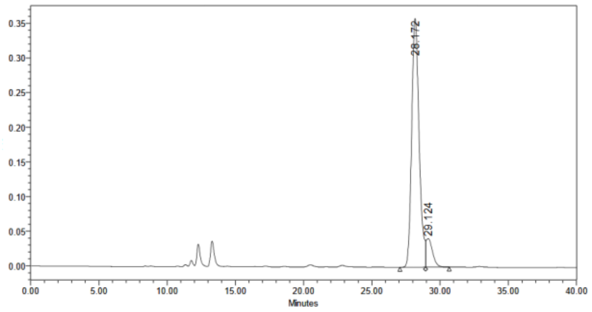

	RT	Area	% Area	Height
1	28.172	13819532	92.07	358999
2	29.124	1190116	7.93	41134

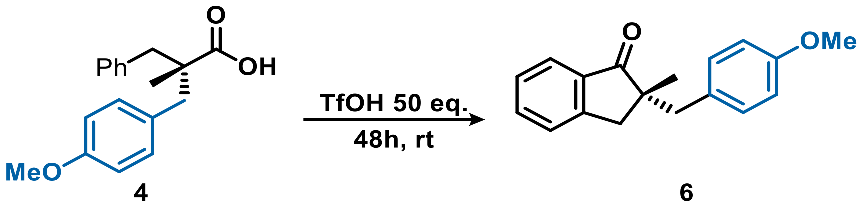

4
99:1 *e.r*

6
80%, 99:1 *e.r*

To a solution of compound **4** (50 mg, 0.17 mmol) in triflic acid (1.7 mL, 50 equiv) was stirred at room temperature for 48 hours under argon. The reaction was quenched with crushed ice and neutralized with saturated aqueous $NaHCO_3$. The mixture was extracted with CH_2Cl_2 (3 × 10 mL), dried over Na_2SO_4, filtered, and concentrated under reduced pressure. The crude product was purified by flash column chromatography (PE/EtOAc) to afford compound **6** as a white solid (40 mg, 80%, 99:1 e.r.). The enantiomeric excess was determined by HPLC analysis on a Daicel Chiralpak IC-5 column: 90:10 Hexane/IPA, flow rate 0.5mL/min, λ= 228.1 nm: TRMajor = 31.04 min, TRMinor = 26.03 min. er = 99.0:1.0.

^{1}H NMR (400 MHz, $CDCl_3$) δ 7.76 (dt, J = 7.6, 1.1 Hz, 1H), 7.59 – 7.51 (m, 1H), 7.39 – 7.31 (m, 2H), 7.11 – 7.04 (m, 2H), 6.85 – 6.69 (m, 2H), 3.75 (s, 3H), 3.26 (d, J = 17.1 Hz, 1H), 3.01 (d, J = 13.6 Hz, 1H), 2.80 – 2.72 (m, 2H), 1.26 (s, 3H).

^{13}C NMR (101 MHz, $CDCl_3$) δ 211.2, 158.3, 152.7, 135.9, 131.3 (2C), 130.0, 127.5, 126.7, 124.4, 113.6 (2C), 55.3, 50.7, 42.6, 39.0, 29.9, 24.7.

HRMS (ESI) [M + Na] calculated for $[C_{18}H_{18}NaO_2]^+$ = 289.1199 found: 289.1205.

$[\alpha]_D^{20}$= - 49.3 (c = 0.43, $CHCl_3$).

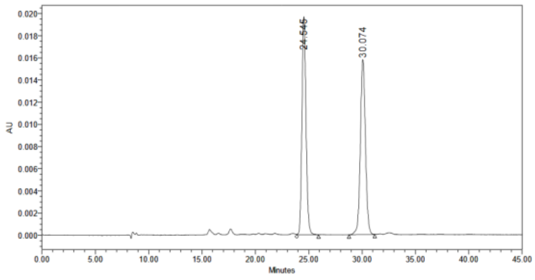

	RT	Area	% Area	Height
1	24.545	520117	49.63	19686
2	30.074	527961	50.37	15795

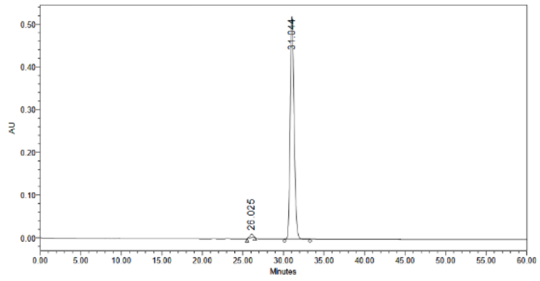

	RT	Area	% Area	Height
1	26.025	694194	0.83	69599
2	31.044	83320061	99.17	2421217

7. Experimental Mechanistic Studies and Control Experiments

7.1 Synthesis of [D]3aa

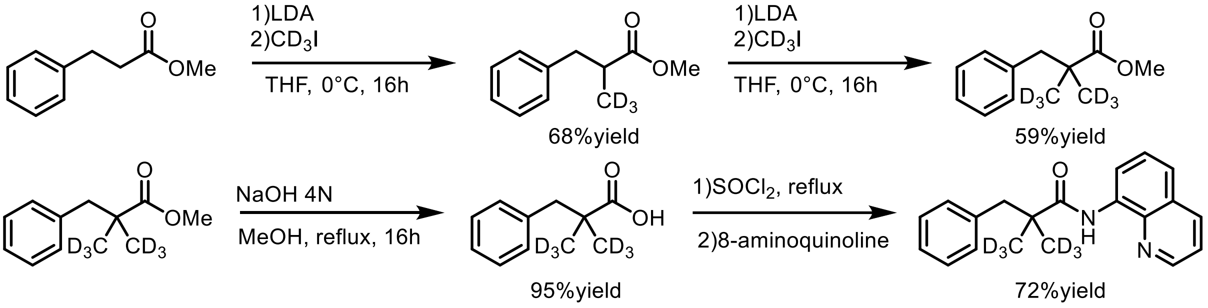

72%yield
[D]1aa

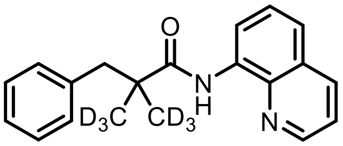

[D]1aa

[D]3aa was synthesized according to our general procedure. The alkylation was performed using CD_3I as electrophile according to the literature.[15] [D]3aa was obtained with a global yield of 27% (850.0 mg, 2,74mmol).

^{1}H NMR (400 MHz, $CDCl_3$) δ 10.16 (s, 1H), 8.84 (dd, J = 7.5, 1.5 Hz, 1H), 8.75 (dd, J = 4.2, 1.7 Hz, 1H), 8.14 (dd, J = 8.3, 1.7 Hz, 1H), 7.55 (dd, J = 8.3, 7.5 Hz, 1H), 7.50 (dd, J = 8.3, 1.5 Hz, 1H), 7.43 (dd, J = 8.3, 4.2 Hz, 1H), 7.22 – 7.12 (m, 5H), 3.04 (s, 2H).

^{13}C NMR (101 MHz, $CDCl_3$) δ 176.3, 148.3, 138.9, 138.1, 136.4, 134.6, 130.4 (2C), 128.1 (2C), 128.0, 127.6, 126.5, 121.6, 121.5, 116.4, 46.9, 44.7, 24.4(CD_3).

HRMS (ESI) [M + Na] calculated for $[C_{20}H_{14}D_6N_2NaO]^+$ = 333.1844 found: 333.1851.

[15] Wang, X., Xie, P., Qiu, R., Zhu, L., Liu, T., Li, Y., Iwasaki, T., Au, C.-T., Xu, X., Xia, Y., et al. (2017). Nickel-catalysed direct alkylation of thiophenes via double C(sp^3)–H/C(sp^2)–H bond cleavage: the importance of KH_2PO_4. Chem. Commun. 53, 8316–8319. https://doi.org/10.1039/C7CC04252C.

7.2 Intermolecular KIE experiment

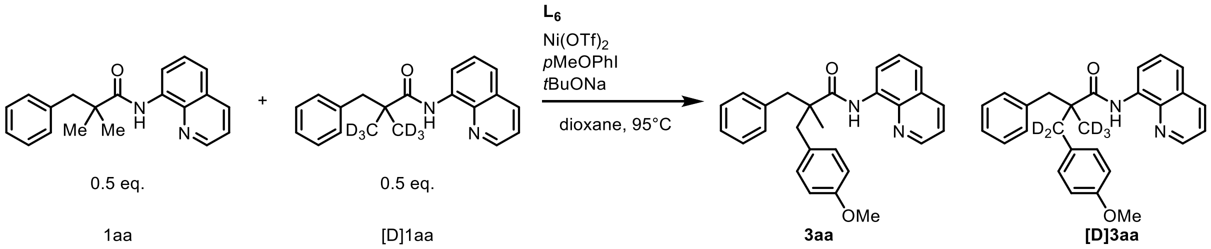

To an oven-dried 5 mL screw-capped vial, in a glove box 2-benzyl-2-(methyl-d$_3$)-N-(quinolin-8-yl)propanamide **[D]1aa** (15.5 mg, 0.05 mmol) and 2,2-dimethyl-3-phenyl-N-(quinolin-8-yl)propenamide (15.2 mg, 0.05 mmol), Ni(OTf)$_2$ (3.6 mg, 0.01 mmol), L$_6$ (10.15 mg, 0.02 mmol), NaO*t*Bu (13.5 mg,0.14 mmol) and 1,4-dioxane (0.2 mL) were added. The mixture was stirred for 4 h at 95°C followed by cooling. 30 mL of water was added to the reaction mixture and the aqueous layer was extracted with ethyl acetate (3 x 10 mL). The combined organic layers were washed with brine (20 mL), dried over $MgSO_4$, filtered and evaporated in vacuo. The residue was purified by column chromatography on silica gel (eluent: PE/EtOAc= 90/10) to afford the desired arylated product. The ratio of deuterium was determined by ^{1}H-NMR.

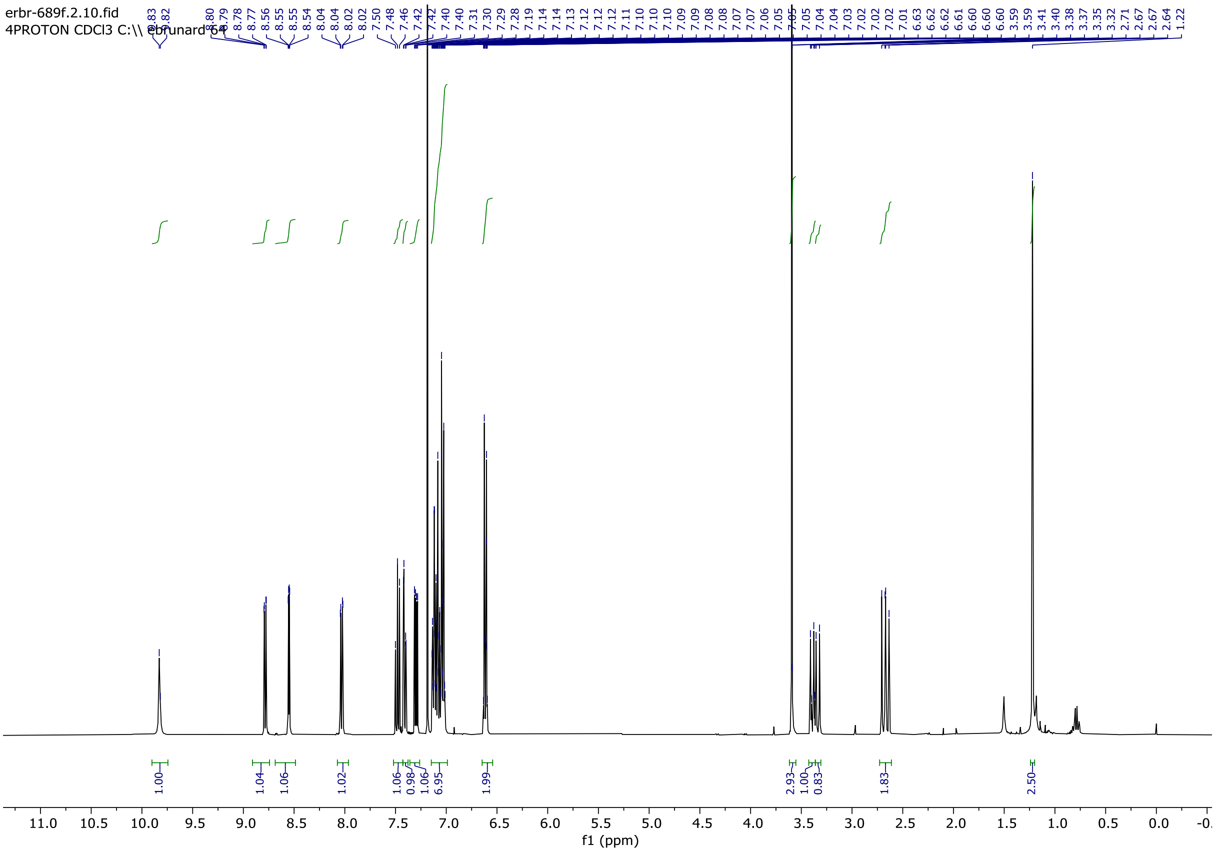

7.3 KIE for two parallel reactions

Inside the glovebox, several 5 mL Schlenk tubes, each tube was charged with either **1aa** (30.1 mg, 0.1 mmol) or **[d]1aa** (30.3 mg, 0.1 mmol), $Ni(OTf)_2$ (3.6 mg, 10 mol%), (*S*)-L_6 (10.15 mg, 20 mol%), NaO*t*Bu (13.5 mg, 0.14 mmol, 1.4 eq), and 4-Iodoanisol (46.7 mg, 0.2 mmol, 2.0 eq). Anhydrous 1,4-dioxane (0.2 mL) were then added. The tubes stirred at 95°C. Upon completion, each reaction mixture was diluted with dichloromethane and filtered through a Celite pad, which was subsequently washed with dichloromethane. The solvent was removed under vacuum. Yields of **3aa** or **[d]3aa** were determined by ^{1}H NMR spectroscopy using 1,3,5-trimethoxybenzene as an internal standard (k_H/k_D = 2.4).

Time (s)	Yield (%) of 3aa	Yield (%) of 3aaD3
3600	10	
5400	15,5	
7200	20	9
9000	25	11
10800	30	14
12600	35	16
14400	39	17
16200	43	18,5
18000	47	22

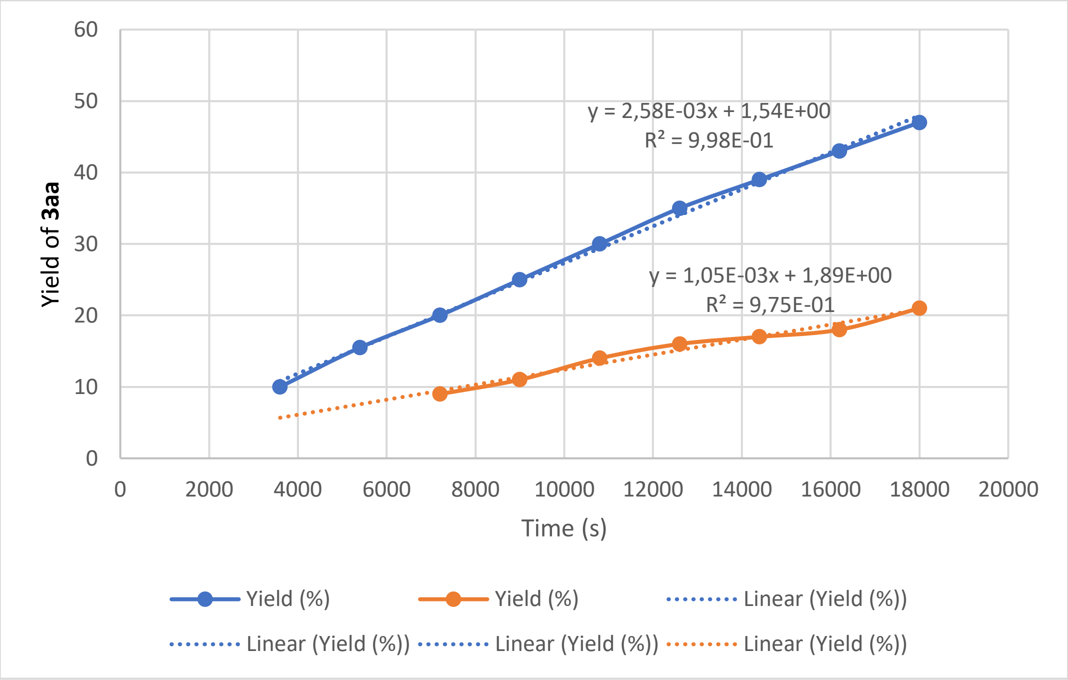

Inside the glovebox, several 5 mL Schlenk tubes, each tube was charged with either **1aa** (30.1 mg, 0.1 mmol) or **[d]1aa** (30.3 mg, 0.1 mmol), $Ni(OTf)_2$ (3.6 mg, 10 mol%), MesCOOH (3.3 mg, 20 mol%), Na_2CO_3 (21.2 mg, 0.2 mmol, 2.0 eq), and 4-Iodoanisol (46.7 mg, 0.2 mmol, 2.0 eq). Anhydrous 1,4-dioxane (0.2 mL) were then added. The tubes stirred at 95°C. Upon completion, each reaction mixture was diluted with dichloromethane and filtered through a Celite pad, which was subsequently washed with dichloromethane. The solvent was removed under vacuum. Yields of **3aa** or **[d]3aa** were determined by ^1H NMR spectroscopy using 1,3,5-trimethoxybenzene as an internal standard (k_H/k_D = 1.4).

Time (s)	Yield (%) of 3aa	Yield (%) of 3aaD3
3600	5	
5400	7	
7200	9	6,5
9000	11	8,4
10800	13	9,9
12600	15	11,4
14400	17	13
16200	18	13,7
18000	19	14,5

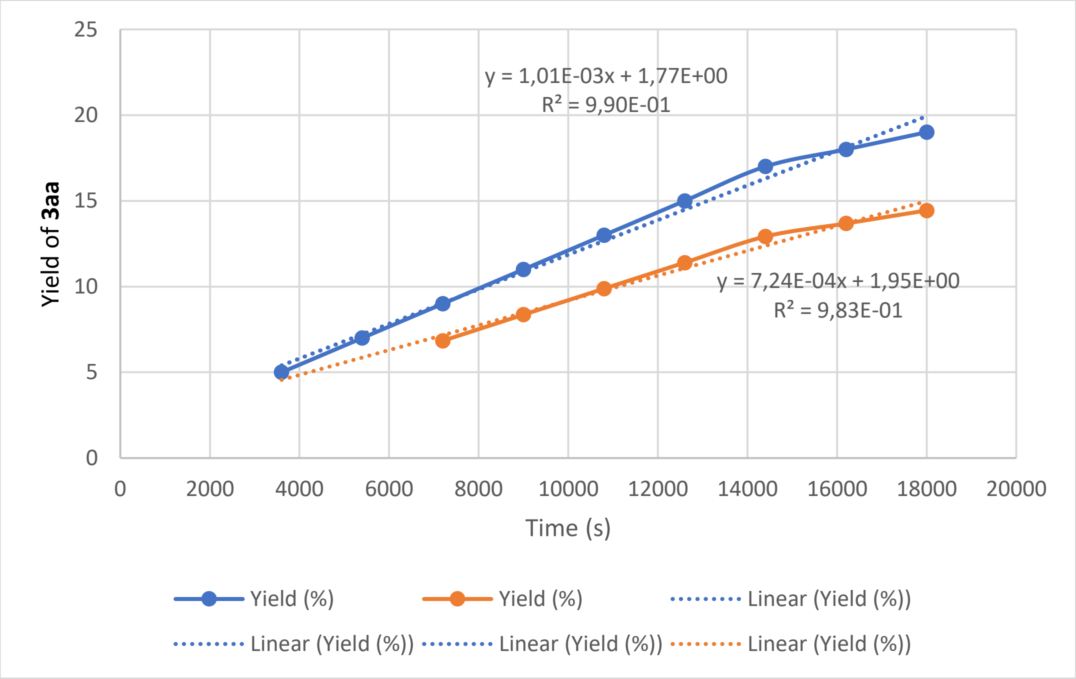

7.4 Investigating the reversibility of the C–H activation step through H/D exchange

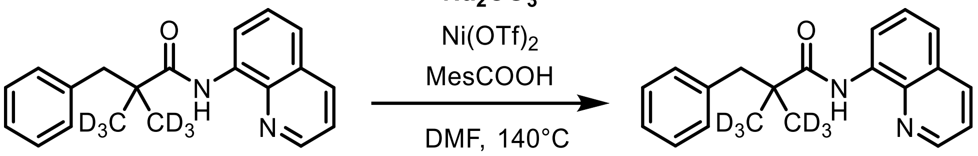

[D]1aa

Ni-MesCO$_2$H system

[D]1aa

To an oven-dried 5 mL screw-capped vial, in a glove box 2-benzyl-2-(methyl-d$_3$)-N-(quinolin-8-yl)propanamide **[D]1aa** (31.0 mg, 0.1 mmol), $Ni(OTf)_2$ (3.6 mg, 0.01 mmol), MesCOOH (3.3 mg, 0.02 mmol), Na_2CO_3 (22 mg,0.2 mmol) and DMF (0.2 mL) were added. The mixture was stirred for 16 h at 140°C followed by cooling. 15 mL of water was added to the reaction mixture and the aqueous layer was extracted with ethyl acetate (3 x 10 mL). The combined organic layers were washed with brine (20 mL), dried over $MgSO_4$, filtered and evaporated in vacuo. The residue was purified by column chromatography on silica gel (eluent: PE/EtOAc= 90/10) to afford the desired starting. The ratio of deuterium was determined by ^{1}H-NMR.

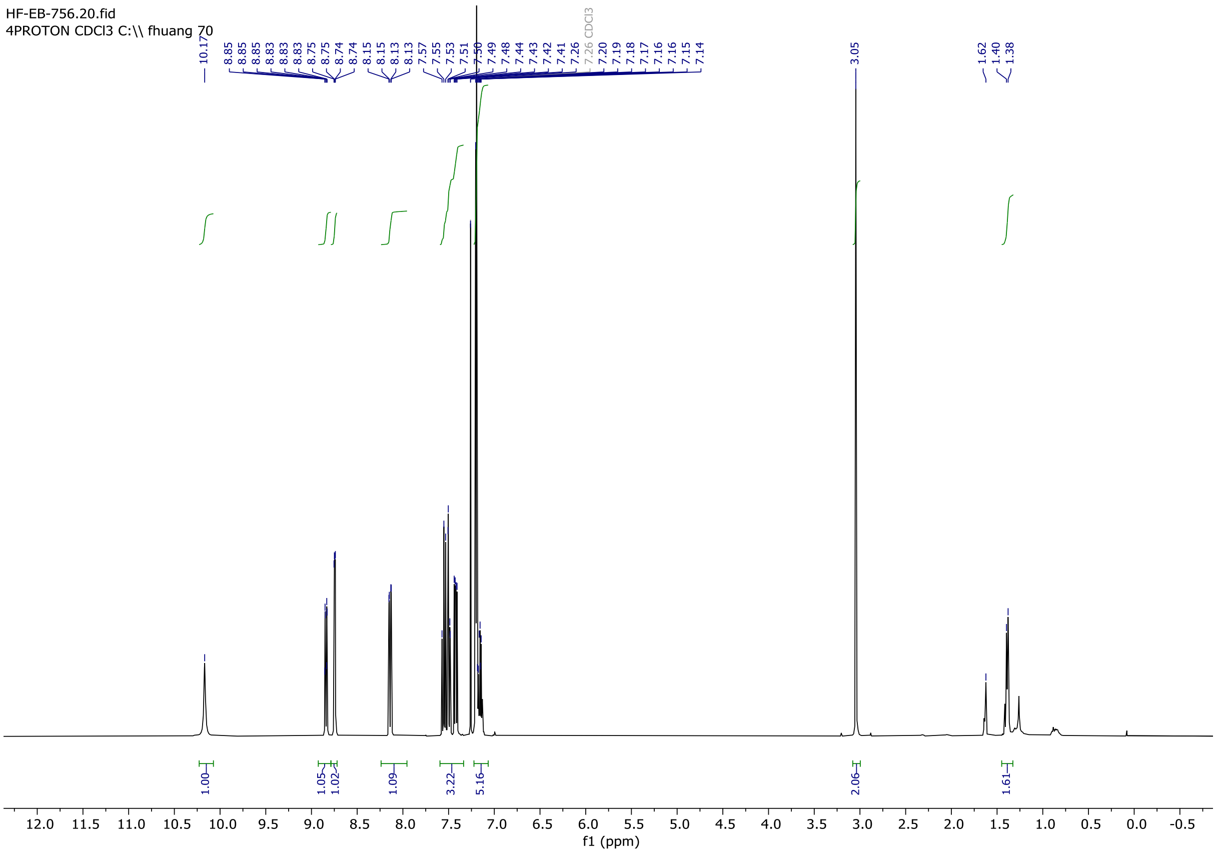

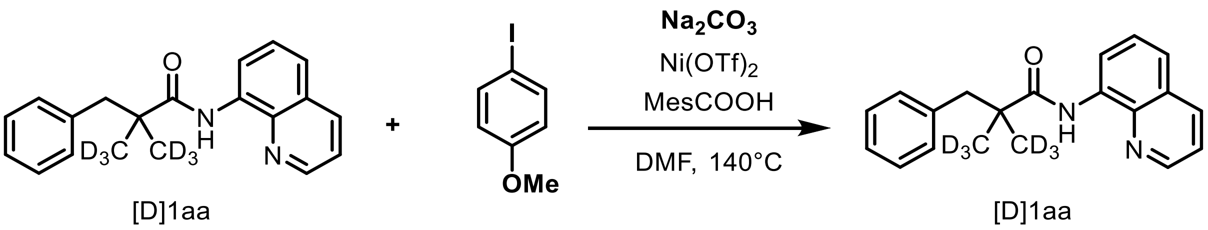

Ni-MesCO$_2$H +ArI system

To an oven-dried 5 mL screw-capped vial, in a glove box 2-benzyl-2-(methyl-d$_3$)-N-(quinolin-8-yl)propanamide **[D]1aa** (31.0 mg, 0.1 mmol), 4-iodoanisole (46.7 mg, 0.2 mmol), Ni(OTf)$_2$ (3.6 mg, 0.01 mmol), MesCOOH (3.3 mg, 0.02 mmol), Na$_2$CO$_3$ (22 mg,0.2 mmol) and DMF (0.2 mL) were added. The mixture was stirred for 16 h at 140°C followed by cooling. 15 mL of water was added to the reaction mixture and the aqueous layer was extracted with ethyl acetate (3 x 10 mL). The combined organic layers were washed with brine (20 mL), dried over MgSO$_4$, filtered and evaporated in vacuo. The residue was purified by column chromatography on silica gel (eluent: PE/EtOAc= 20/1) to afford the desired starting. The ratio of deuterium was determined by ^{1}H-NMR.

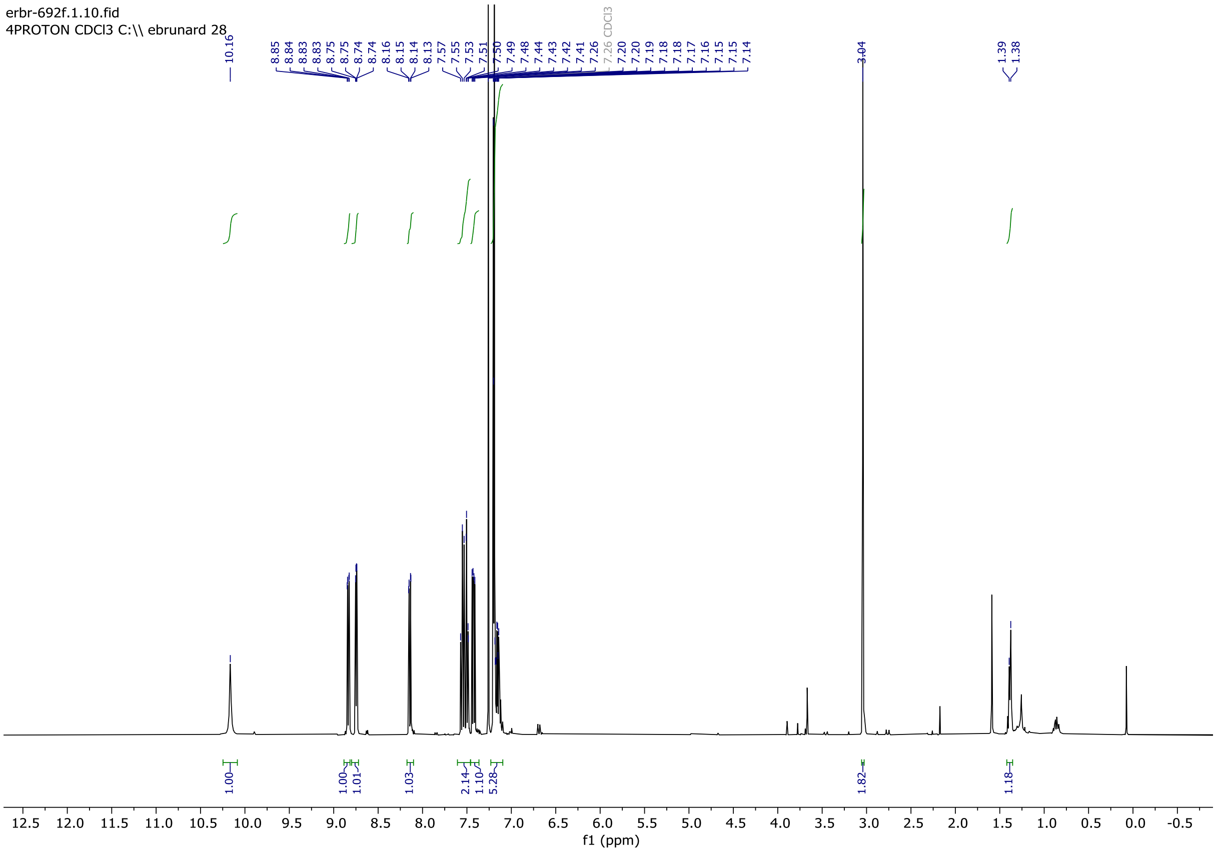

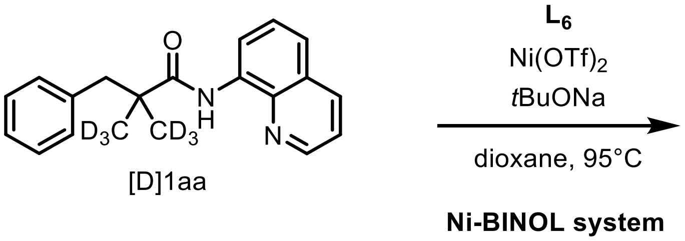

To an oven-dried 5 mL screw-capped vial, in a glove box 2-benzyl-2-(methyl-d_3)-N-(quinolin-8-yl)propanamide **[D]1aa** (31.0 mg, 0.1 mmol), $Ni(OTf)_2$ (3.6 mg, 0.01 mmol), L_6 (10.15 mg, 0.02 mmol), NaO*t*Bu (13.5 mg,0.14 mmol) and 1,4-dioxane (0.2 mL) were added. The mixture was stirred for 16 h at 95°C followed by cooling. 15 mL of water was added to the reaction mixture and the aqueous layer was extracted with ethyl acetate (3 x 10 mL). The combined organic layers were washed with brine (20 mL), dried over $MgSO_4$, filtered and evaporated in vacuo. The residue was purified by column chromatography on silica gel (eluent: PE/EtOAc= 20/1) to afford the desired starting. The ratio of deuterium was determined by ^{1}H-NMR.

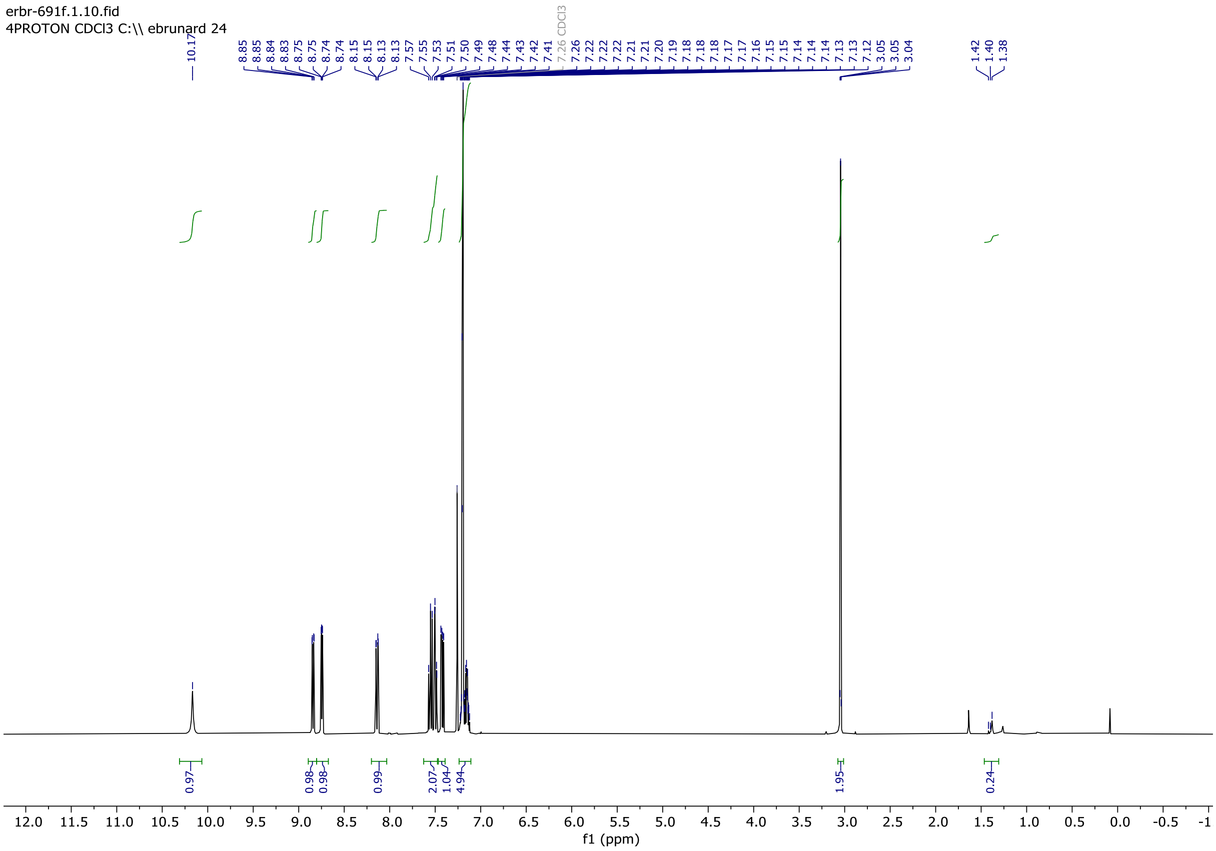

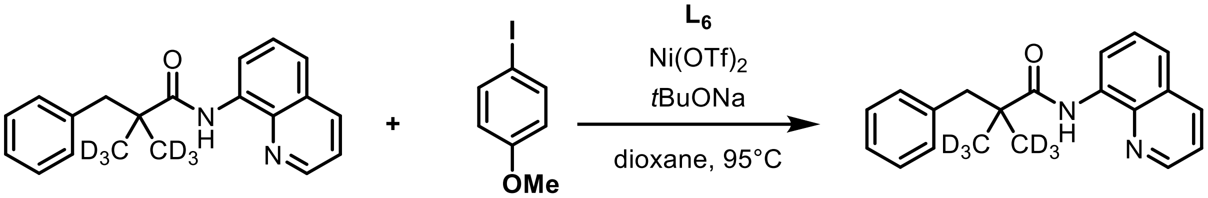

[D]1aa [D]1aa

Ni-BINOL system + ArI

To an oven-dried 5 mL screw-capped vial, in a glove box 2-benzyl-2-(methyl-d$_3$)-N-(quinolin-8-yl)propanamide **[D]1aa** (31.0 mg, 0.1 mmol), 4-iodoanisole (46.7 mg, 0.2 mmol), Ni(OTf)$_2$ (3.6 mg, 0.01 mmol), L$_6$ (10.15 mg, 0.02 mmol), NaO*t*Bu (13.5 mg,0.14 mmol) and 1,4-dioxane (0.2 mL) were added. The mixture was stirred for 16 h at 95°C followed by cooling. 15 mL of water was added to the reaction mixture and the aqueous layer was extracted with ethyl acetate (3 x 10 mL). The combined organic layers were washed with brine (20 mL), dried over $MgSO_4$, filtered and evaporated in vacuo. The residue was purified by column chromatography on silica gel (eluent: hexane/EtOAc= 20/1) to afford the desired starting. The ratio of deuterium was determined by ^{1}H-NMR.

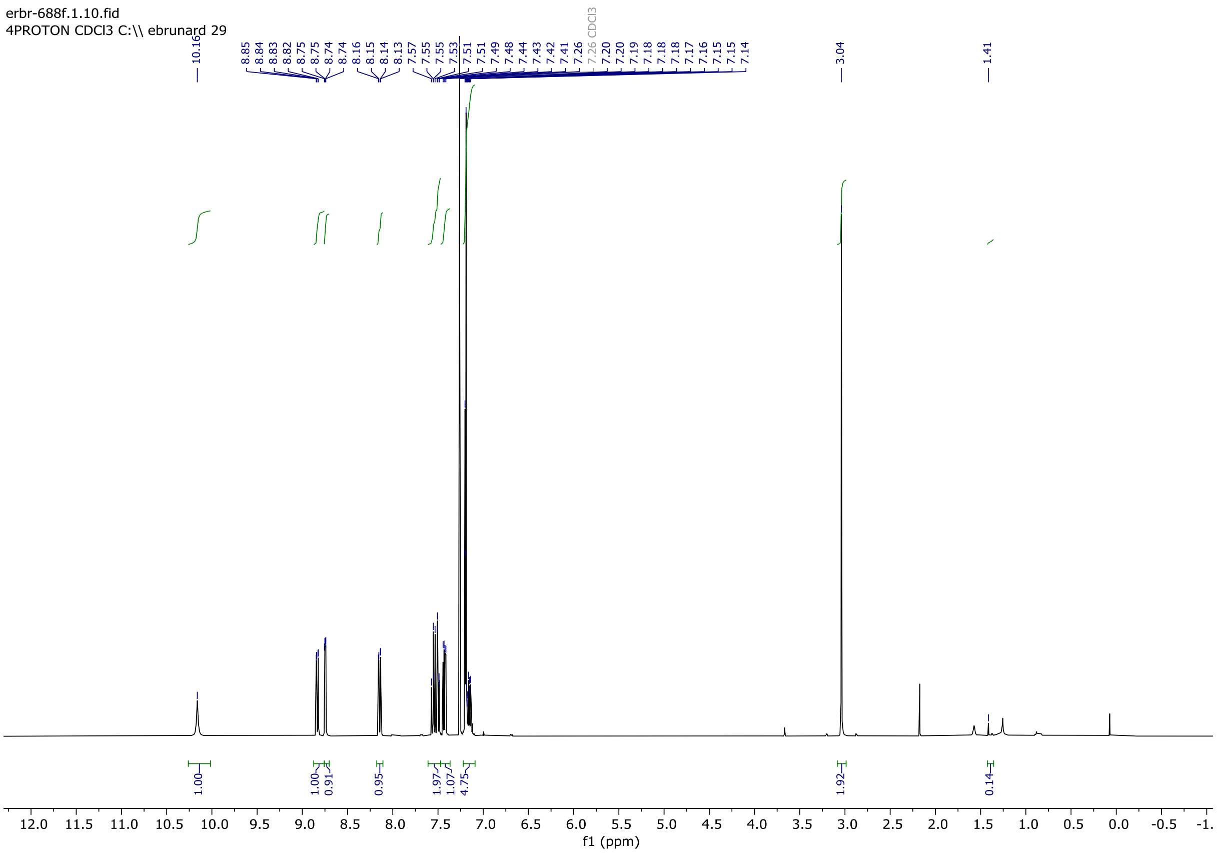

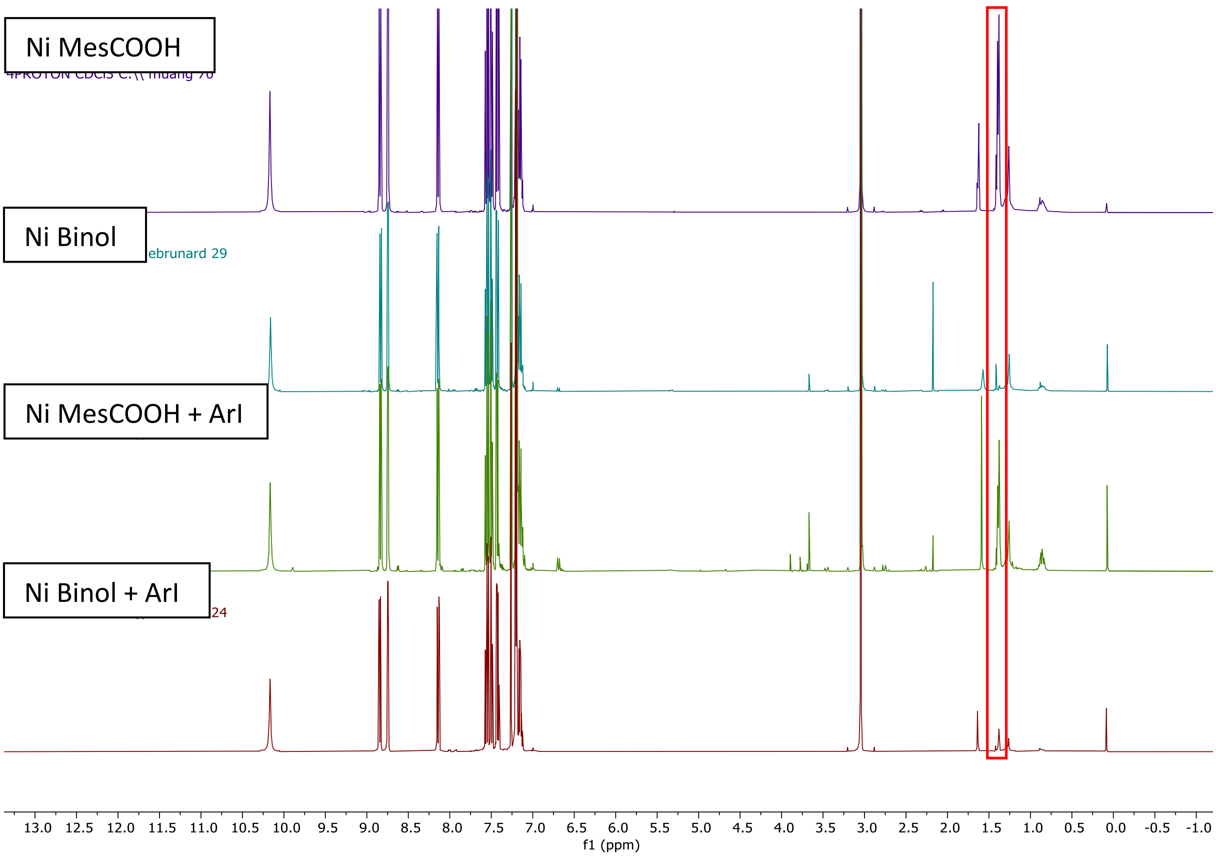

Ni MesCOOH
Ni Binol
Ni MesCOOH + ArI
Ni Binol + ArI
13.0 12.5 12.0 11.5 11.0 10.5 10.0 9.5 9.0 8.5 8.0 7.5 7.0 6.5 6.0 5.5 5.0 4.5 4.0 3.5 3.0 2.5 2.0 1.5 1.0 0.5 0.0 -0.5 -1.0
f1 (ppm)

7.5 Synthesis and Characterization of Ni(II) intermediates

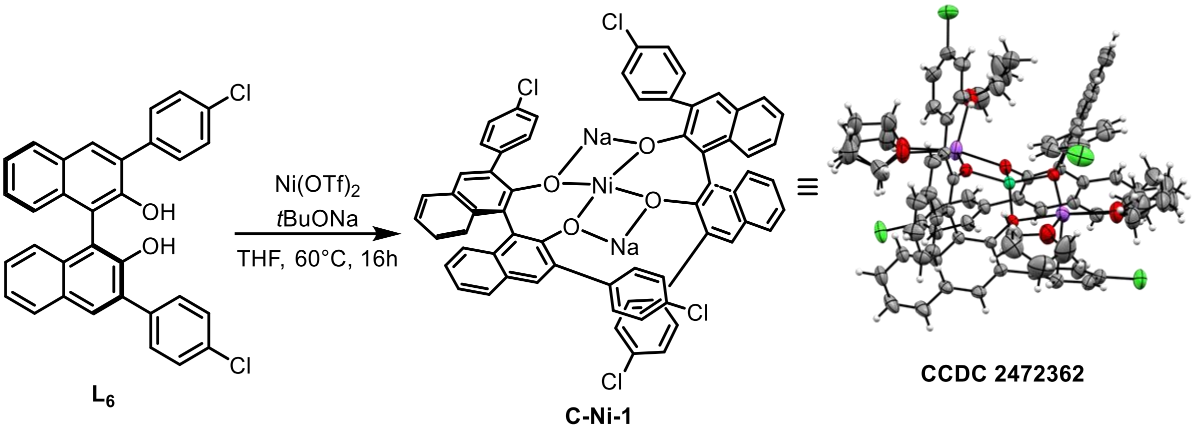

To the 10 mL Schlenk tube was added $Ni(OTf)_2$ (21.1 mg, 0.056 mmol), (S)-L_6 (30.0 mg, 0.056 mmol), and NaO*t*Bu (11.36 mg, 0.12 mmol, 2.0 eq), followed by addition of anhydrous THF (0.6 mL) and inside a glovebox. The mixture was stirred at 60°C under Ar for 16 h. The residue was recrystallized in THF at rt by slowly diffusion dry toluene to afford Ni(binolate)$_2$ **C-Ni-1** as a dark-green crystal. The structure of Ni(binolate)$_2$ **C-Ni-1** was confirmed by X-ray analysis.

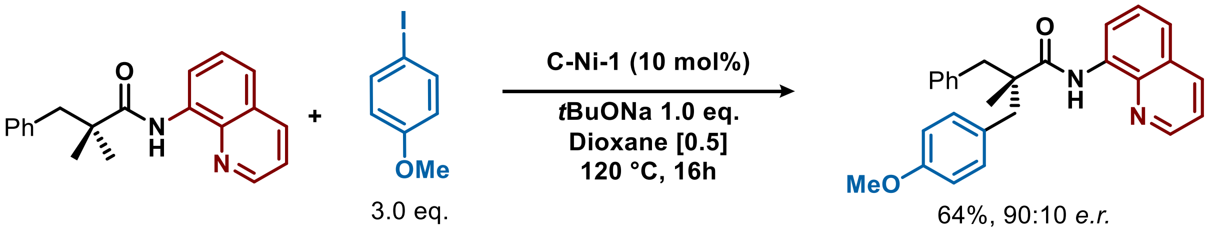

To an oven-dried 5 mL Schlenk flask, containing the **1aa** (0.1 mmol, 1.0 eq.), iodoanisol (70.2 mg, 0.3 mmol, 3.0 eq.), **C-Ni-1** (6.1 mg, 0.01 mmol, 0.1 eq.), *t*BuONa (9.6 mg, 0.1 mmol, 1.0 eq.) and dry freshly distilled 1,4-dioxane (0.2 mL) were added under Ar atmosphere inside a glovebox. The mixture was stirred for 16 h at 120°C outside of the glovebox followed by cooling. 5 mL of water was added to the reaction mixture, and the aqueous layer was extracted with ethyl acetate (3 x 10 mL). The combined organic phase was washed with brine (20 mL), dried over $NaSO_4$, filtered and evaporated in vacuo. The residue was purified by column chromatography on silica gel (eluent: PE/EtOAc=10/1 or PE/DCM=30/70) to afford the desired arylated product **3aa** in 64% yield (26.3 mg).

8. X-Ray Crystallographic Data

A single crystal of **3aa** suitable for X-ray crystallography was obtained by crystallization via evaporation from its diethyl ether/pentane solution (CCDC 2472361).

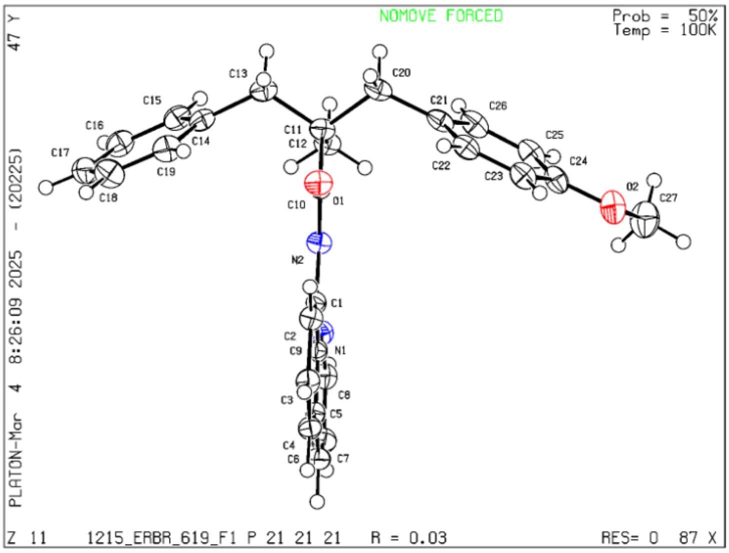

Identification code	1215_ERBR_619_F1
Empirical formula	C_{27} H_{26} N_2 O_2
Formula weight	410.50
Temperature	100(2) K
Wavelength	1.54178 A
Crystal system, space group	Orthorhombic, P2(1)2(1)2(1)
Unit cell dimensions	a = 9.1162(9) A alpha = 90 deg.
	b = 13.0562(14) A beta = 90 deg.
	c = 18.553(2) A gamma = 90 deg.
Volume	2208.2(4) A^3
Z, Calculated density	4, 1.235 Mg/m^3
Absorption coefficient	0.616 mm^-1
F(000)	872
Crystal size	0.500 x 0.500 x 0.380 mm
Theta range for data collection	4.140 to 72.227 deg.
Limiting indices -	11<=h<=11, -16<=k<=16, -22<=l<=22
Reflections collected / unique	43511 / 4337 [R(int) = 0.0315]
Completeness to theta = 67.679	99.8 %
Absorption correction	Semi-empirical from equivalents
Max. and min. transmission	0.7536 and 0.5968
Refinement method	Full-matrix least-squares on F^2
Data / restraints / parameters	4337 / 0 / 282
Goodness-of-fit on F^2	1.076
Final R indices [I>2sigma(I)]	R1 = 0.0254, wR2 = 0.0656
R indices (all data)	R1 = 0.0255, wR2 = 0.0658
Absolute structure parameter	0.01(4)
Extinction coefficient	n/a
Largest diff. peak and hole	0.141 and -0.156 e.A^-3

A single crystal of **C-Ni-1** suitable for X-ray crystallography was obtained by crystallization via THF at rt by slowly diffusion dry toluene to afford a dark-green crystal (CCDC 2472362).

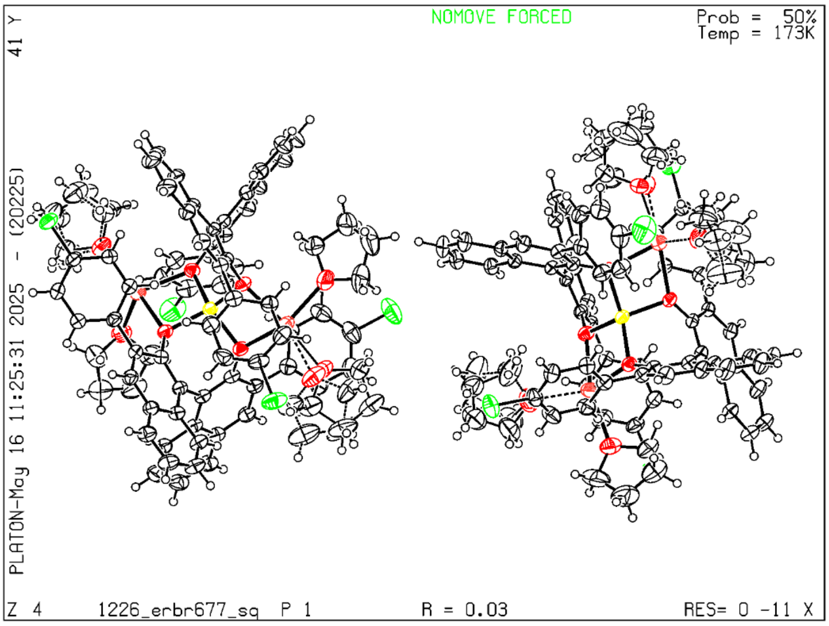

Identification code	1226_erbr677_sq
Empirical formula	$C_{80} H_{68} Cl_4 Na_2 Ni O_8$
Formula weight	1403.83
Temperature	173(2) K
Wavelength	1.54178 A
Crystal system, space group	Triclinic, P1
Unit cell dimensions	a = 13.7838(17) A alpha = 103.684(5) deg.
	b = 14.1054(19) A beta = 93.080(5) deg.
	c = 21.682(3) A gamma = 116.676(7) deg.
Volume	3597.3(9) A^3
Z, Calculated density	2, 1.296 Mg/m^3
Absorption coefficient	2.317 mm^-1
F(000)	1460
Crystal size	0.170 x 0.156 x 0.060 mm
Theta range for data collection	2.134 to 72.416 deg.
Limiting indices	-17<=h<=16, -17<=k<=17, -26<=l<=26
Reflections collected / unique	112956 / 26654 [R(int) = 0.0257]
Completeness to theta = 67.679	99.9 %
Absorption correction	Semi-empirical from equivalents
Max. and min. transmission	0.7536 and 0.6348
Refinement method	Full-matrix least-squares on F^2
Data / restraints / parameters	26654 / 883 / 1941
Goodness-of-fit on F^2	1.028
Final R indices [I>2sigma(I)]	R1 = 0.0283, wR2 = 0.0761
R indices (all data)	R1 = 0.0293, wR2 = 0.0769
Absolute structure parameter	0.021(2)
Extinction coefficient	n/a
Largest diff. peak and hole	0.306 and -0.293 e.A^-3

9.DFT Calculations

9.1. Computational details:

DFT calculations were performed using the Gaussian16 program package.[15] Geometry optimizations were carried out with the B3LYP functional,[16] including Grimme's D3 dispersion correction,[17] and using the 6-31G*[18] and LANL2DZ[19] (for Ni and I) basis set. Frequency calculations confirmed the nature of each stationary point as either a minimum (no imaginary frequencies) or a transition state (one imaginary frequency). Intrinsic Reaction Coordinate (IRC) calculations and relaxations to reactants and products were conducted as needed to further validate the reaction pathways.

Single-point energy refinements were performed at the B3LYP-D3/6-311+G**/LANL2DZ level of theory. Solvent effects were accounted for using the SMD implicit solvation model (1,4-dioxane) [20] in the single-point calculations at 373.15 K. All 3D structures were visualized using CYLview 1.0,[21] and non-covalent interaction (NCI) [22] plots were generated with the Multiwfn software.[23]

9.2. NCI Plots for the enantiodeterminig C-H activation step:

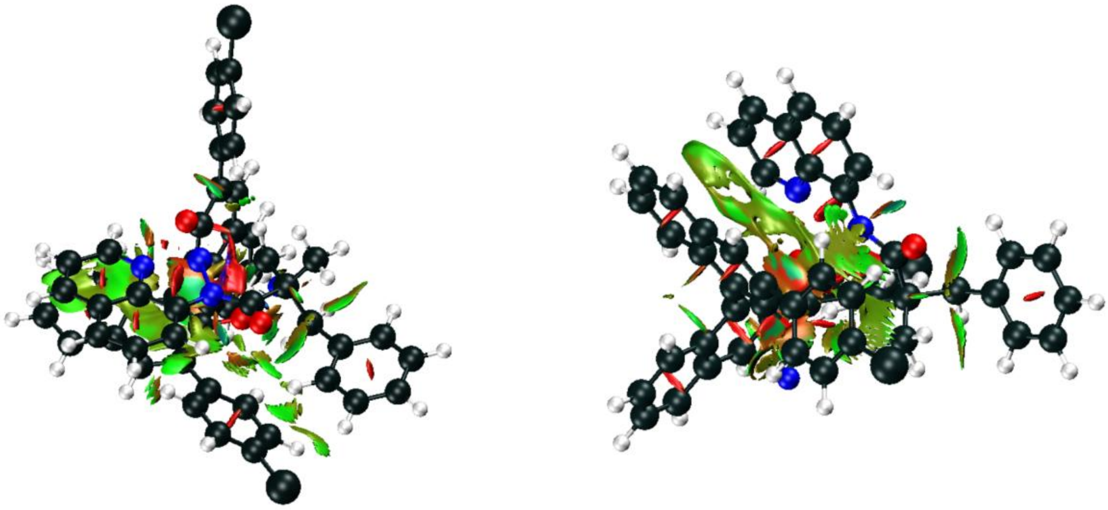

TS23 (26.7 kcal/mol) **TS23_Minor (27.4 kcal/mol)**

Figure S1: 3D-NCI plots for the enantiodetermining C-H activation step.

9.3. Complete Free Energy profile:

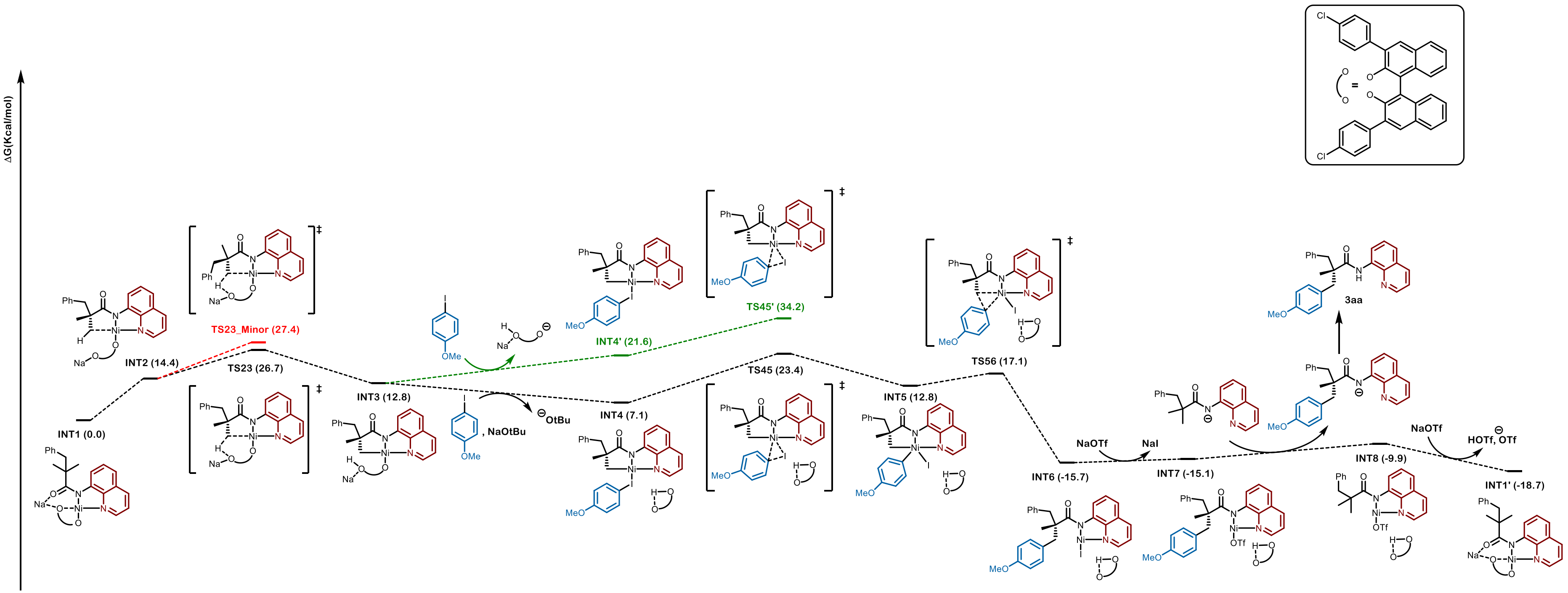

Figure S2: Free energy profile of the reaction mechanism at the B3LYP-D3/6-311+G**–LANL2DZ(Ni,I)–SMD(dioxane)//B3LYP-D3/6-31G*–LANL2DZ(Ni,I)level of theory at 373.15K. Energies in kcal/mol.

9.4. XYZ coordinates

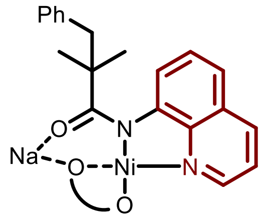

INT1

E (6-311+G**/LANL2DZ): -3592.033416 A.U.

G_{corr} (6-31G*/LANL2DZ): 0.668338 A.U.

28	2.388073000	2.279578000	-0.439373000
7	1.666095000	4.087169000	-0.220120000
7	0.754126000	1.767917000	0.440419000
8	2.595852000	6.180936000	-0.014430000
6	2.274993000	5.191767000	-0.722790000
6	0.862443000	4.097947000	0.920409000
6	0.481228000	5.162849000	1.733289000
6	-0.429444000	4.956417000	2.797095000
6	-0.974038000	3.718637000	3.075712000
6	-0.612615000	2.603354000	2.274083000
6	0.312883000	2.806978000	1.216988000
6	-1.097026000	1.280715000	2.455603000
6	-0.653019000	0.261348000	1.639365000
6	0.301044000	0.539256000	0.636615000
1	0.731236000	-0.220580000	-0.005903000
1	-1.011439000	-0.755240000	1.761439000
1	-1.815657000	1.084229000	3.247278000
1	-1.669120000	3.582730000	3.899111000
1	-0.697561000	5.808897000	3.415150000
1	0.888250000	6.147633000	1.548885000
6	7.403070000	1.606456000	-4.743710000
6	6.816862000	1.487183000	-3.502885000
6	5.490139000	0.985956000	-3.360781000
6	4.780127000	0.610726000	-4.549438000
6	5.419822000	0.728730000	-5.811138000
6	6.703554000	1.217536000	-5.913250000
1	8.412217000	2.001414000	-4.827644000
1	7.360528000	1.780663000	-2.610810000
6	4.848888000	0.860995000	-2.092793000
6	3.439598000	0.168087000	-4.433337000
1	4.864703000	0.443663000	-6.701790000
1	7.179852000	1.310145000	-6.885603000
6	2.783107000	0.150157000	-3.220804000
6	3.487904000	0.524279000	-2.020227000
1	2.899734000	-0.110286000	-5.335429000
6	5.577399000	1.039350000	-0.805663000
6	6.635486000	0.148224000	-0.432159000
6	5.135358000	2.011524000	0.092941000
6	7.092198000	-0.907012000	-1.275327000
6	7.278228000	0.300519000	0.843207000
6	5.695613000	2.058765000	1.420783000
6	8.138875000	-1.719995000	-0.900400000
1	6.603649000	-1.068073000	-2.228552000
6	8.360248000	-0.549520000	1.196556000
6	6.768271000	1.257960000	1.751607000
6	8.792629000	-1.537594000	0.342465000
1	8.464305000	-2.515103000	-1.566245000
1	8.834106000	-0.410023000	2.166019000
1	7.188373000	1.309028000	2.754220000
1	9.620048000	-2.184005000	0.621960000

8	2.838684000	0.525426000	-0.848170000
8	4.201913000	2.925354000	-0.237643000
6	5.054733000	2.912578000	2.455403000
6	5.768432000	3.890321000	3.172142000
6	3.680865000	2.756789000	2.740402000
6	5.133423000	4.703920000	4.120965000
6	3.032927000	3.562821000	3.674419000
6	3.766791000	4.541013000	4.354976000
6	1.363301000	-0.284986000	-3.153201000
6	0.353162000	0.575311000	-2.698553000
6	0.997249000	-1.572229000	-3.571777000
6	-0.977459000	0.166355000	-2.643909000
6	-0.330516000	-2.001067000	-3.524531000
6	-1.307476000	-1.125615000	-3.054106000
1	-1.750153000	0.839722000	-2.287431000
1	-0.603344000	-3.001973000	-3.841914000
1	1.970422000	3.449389000	3.861512000
1	5.691630000	5.457649000	4.666612000
1	3.115909000	2.003433000	2.200989000
1	6.831277000	4.016623000	2.981356000
1	1.767182000	-2.252153000	-3.925564000
1	0.611195000	1.577529000	-2.378537000
17	2.951505000	5.584487000	5.506870000
17	-2.985639000	-1.658195000	-2.971390000
6	2.601431000	5.253837000	-2.234412000
6	4.113314000	5.514101000	-2.402626000
1	4.688973000	4.648892000	-2.052879000
6	1.805554000	6.471678000	-2.762244000
1	2.074548000	7.371942000	-2.204102000
1	2.022903000	6.629138000	-3.822476000
1	0.725138000	6.310983000	-2.659340000
6	2.183228000	3.964637000	-2.993010000
1	2.793927000	3.133194000	-2.624454000
1	1.143292000	3.740930000	-2.733961000
1	4.354636000	5.680965000	-3.456167000
11	4.218892000	4.937060000	0.928651000
1	4.409103000	6.408330000	-1.842949000
6	2.327630000	4.018058000	-4.498704000
6	3.543982000	3.690024000	-5.113824000
6	1.245767000	4.377627000	-5.314924000
6	3.680939000	3.733119000	-6.501780000
6	1.377085000	4.422948000	-6.703934000
6	2.598110000	4.102899000	-7.302275000
1	4.384034000	3.375355000	-4.502709000
1	0.290506000	4.620420000	-4.854702000
1	4.631082000	3.457354000	-6.950218000
1	0.524346000	4.701112000	-7.318132000
1	2.700975000	4.132319000	-8.383965000

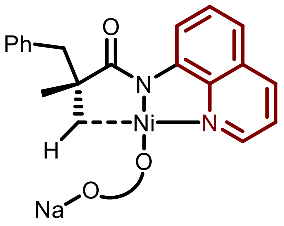

INT2

E (6-311+G**/LANL2DZ): -3592.012498 A.U.

G_{corr} (6-31G*/LANL2DZ): 0.669921 A.U.

28	-1.272909000	-0.348379000	0.293883000
7	-1.623042000	1.142161000	1.399360000
7	-1.634669000	-1.394077000	1.812484000
8	-2.115307000	3.377122000	1.664223000
6	-1.781456000	2.433131000	0.949120000
6	-1.817443000	0.779963000	2.733719000
6	-2.004769000	1.584853000	3.855660000
6	-2.144548000	0.993700000	5.134939000
6	-2.123247000	-0.372311000	5.331540000
6	-1.960859000	-1.228386000	4.212439000
6	-1.802437000	-0.630982000	2.934671000
6	-1.952707000	-2.644653000	4.263119000
6	-1.802162000	-3.382131000	3.107500000
6	-1.634935000	-2.718256000	1.877069000
1	-1.478481000	-3.234303000	0.939367000
1	-1.775951000	-4.465179000	3.127076000
1	-2.064468000	-3.140191000	5.224147000
1	-2.240069000	-0.802257000	6.322120000
1	-2.277582000	1.652920000	5.988802000
1	-2.035380000	2.657419000	3.738187000
6	4.826396000	-1.190174000	-2.520579000
6	3.783451000	-1.279832000	-1.617883000
6	2.452850000	-1.578872000	-2.050567000
6	2.244810000	-1.834247000	-3.450671000
6	3.341703000	-1.738038000	-4.350696000
6	4.606439000	-1.410552000	-3.905247000
1	5.828385000	-0.965737000	-2.164055000
1	3.954810000	-1.127093000	-0.556467000
6	1.341425000	-1.565176000	-1.153659000
6	0.935571000	-2.159297000	-3.888321000
1	3.164871000	-1.933647000	-5.406294000
1	5.434951000	-1.342699000	-4.604381000
6	-0.145021000	-2.189619000	-3.028635000
6	0.047580000	-1.838646000	-1.634066000
1	0.780702000	-2.365844000	-4.944844000
6	1.529590000	-1.207725000	0.284953000
6	1.508458000	-2.227939000	1.281010000
6	1.750340000	0.152486000	0.619312000
6	1.367992000	-3.609812000	0.970816000
6	1.635614000	-1.862291000	2.660290000
6	1.852866000	0.503603000	2.030839000
6	1.371205000	-4.569176000	1.963975000
1	1.266975000	-3.903618000	-0.069581000
6	1.628300000	-2.869617000	3.657166000
6	1.785589000	-0.489325000	2.985545000
6	1.503819000	-4.202513000	3.323526000
1	1.273831000	-5.618791000	1.696801000
1	1.726960000	-2.568711000	4.698185000
1	1.891942000	-0.224278000	4.035095000
1	1.506068000	-4.966312000	4.096394000
8	-1.022349000	-1.795597000	-0.852963000
8	1.834314000	1.078824000	-0.296480000

6	2.046498000	1.910806000	2.459281000
6	2.937648000	2.776113000	1.798508000
6	1.389316000	2.398799000	3.602508000
6	3.193100000	4.058868000	2.278245000
6	1.631327000	3.681308000	4.093668000
6	2.543936000	4.499420000	3.431374000
6	-1.480246000	-2.584080000	-3.547148000
6	-2.657984000	-1.898403000	-3.201761000
6	-1.587045000	-3.660653000	-4.444218000
6	-3.889237000	-2.256907000	-3.744507000
6	-2.812672000	-4.036339000	-4.992191000
6	-3.958044000	-3.325646000	-4.638819000
1	-4.790138000	-1.714982000	-3.476436000
1	-2.881115000	-4.875124000	-5.676683000
1	1.109505000	4.044989000	4.972501000
1	3.888138000	4.714898000	1.765005000
1	0.662091000	1.771927000	4.106985000
1	3.437430000	2.430057000	0.902695000
1	-0.698364000	-4.229710000	-4.701838000
1	-2.607347000	-1.079629000	-2.497610000
17	2.861067000	6.121210000	4.041932000
17	-5.512201000	-3.788243000	-5.321016000
6	-1.519562000	2.676765000	-0.555753000
6	-0.206246000	3.514241000	-0.728470000
1	0.603442000	2.961984000	-0.240310000
6	-2.732993000	3.420115000	-1.134580000
1	-2.914900000	4.335527000	-0.568696000
1	-2.557838000	3.683072000	-2.185245000
1	-3.634073000	2.796367000	-1.081680000
6	-1.311706000	1.356286000	-1.303818000
1	-1.151511000	1.495179000	-2.379597000
1	-0.367137000	0.871437000	-0.966768000
1	-2.209761000	0.716987000	-1.242295000
1	0.011338000	3.509656000	-1.809461000
6	-0.226333000	4.948083000	-0.254266000
6	-0.540505000	5.993351000	-1.132966000
6	0.098883000	5.263483000	1.071367000
6	-0.538156000	7.321223000	-0.700039000
6	0.108469000	6.587363000	1.507770000
6	-0.211219000	7.621246000	0.623589000
1	-0.789143000	5.764687000	-2.167855000
1	0.334818000	4.464034000	1.762846000
1	-0.784867000	8.118371000	-1.397093000
1	0.374399000	6.809653000	2.537002000
1	-0.200811000	8.653787000	0.963321000
11	2.471235000	1.057121000	-2.293151000

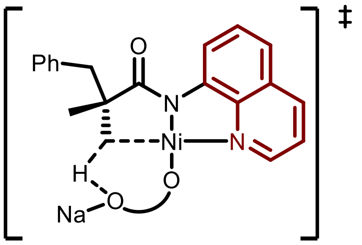

TS23
E (6-311+G**/LANL2DZ): -3591.985905 A.U.
G_{corr} (6-31G*/LANL2DZ): 0.663384 A.U.

28	-1.248819000	-0.219610000	0.349885000
7	-1.656011000	1.225433000	1.503937000
7	-1.573085000	-1.326965000	1.909527000
8	-2.131280000	3.469471000	1.761693000
6	-1.758683000	2.526635000	1.062930000

6	-1.905316000	0.840665000	2.821805000
6	-2.181267000	1.633021000	3.935898000
6	-2.330162000	1.036833000	5.210774000
6	-2.219135000	-0.324150000	5.415744000
6	-1.962377000	-1.170081000	4.306540000
6	-1.819032000	-0.572707000	3.025508000
6	-1.829200000	-2.580567000	4.374405000
6	-1.579288000	-3.311787000	3.232410000
6	-1.457115000	-2.643679000	1.996824000
1	-1.244773000	-3.156084000	1.066567000
1	-1.453628000	-4.388030000	3.265115000
1	-1.922130000	-3.074144000	5.338776000
1	-2.331513000	-0.757280000	6.405604000
1	-2.535735000	1.687664000	6.056914000
1	-2.264334000	2.702407000	3.813015000
6	4.889359000	-1.566161000	-2.568089000
6	3.851097000	-1.560103000	-1.657647000
6	2.501443000	-1.779190000	-2.077826000
6	2.261720000	-2.019915000	-3.476706000
6	3.357156000	-2.024775000	-4.382112000
6	4.645973000	-1.797783000	-3.944842000
1	5.906859000	-1.402752000	-2.222888000
1	4.044254000	-1.396698000	-0.601737000
6	1.393599000	-1.689052000	-1.181821000
6	0.921780000	-2.221585000	-3.905299000
1	3.158783000	-2.215684000	-5.434719000
1	5.473489000	-1.806377000	-4.648200000
6	-0.157055000	-2.159569000	-3.043575000
6	0.067958000	-1.843214000	-1.638836000
1	0.745929000	-2.405890000	-4.962811000
6	1.620013000	-1.368388000	0.260153000
6	1.736066000	-2.409345000	1.228696000
6	1.659777000	-0.030918000	0.651064000
6	1.659485000	-3.786278000	0.883158000
6	1.894039000	-2.058738000	2.607715000
6	1.845935000	0.325249000	2.030504000
6	1.736252000	-4.763887000	1.852294000
1	1.528917000	-4.055094000	-0.160548000
6	1.977797000	-3.089575000	3.580854000
6	1.954326000	-0.688622000	2.963519000
6	1.899020000	-4.415352000	3.215715000
1	1.673297000	-5.811348000	1.569140000
1	2.096834000	-2.810303000	4.625208000
1	2.100495000	-0.432474000	4.009877000
1	1.960179000	-5.195497000	3.969633000
8	-0.963034000	-1.723511000	-0.840604000
8	1.436233000	0.947740000	-0.270780000
6	1.898760000	1.740937000	2.467940000
6	2.668786000	2.697803000	1.785257000
6	1.209399000	2.147608000	3.620698000
6	2.762480000	4.010005000	2.242472000
6	1.287489000	3.458363000	4.088508000
6	2.073259000	4.378941000	3.398604000
6	-1.524656000	-2.422264000	-3.558406000
6	-2.635636000	-1.655394000	-3.164280000

6	-1.733287000	-3.449229000	-4.495301000
6	-3.899291000	-1.886834000	-3.701871000
6	-2.993040000	-3.697469000	-5.037665000
6	-4.069346000	-2.907431000	-4.637746000
1	-4.747511000	-1.283261000	-3.396405000
1	-3.141474000	-4.499199000	-5.753173000
1	0.733225000	3.764288000	4.969143000
1	3.355543000	4.744363000	1.708488000
1	0.576492000	1.439599000	4.143901000
1	3.196170000	2.408508000	0.883165000
1	-0.900792000	-4.082315000	-4.788943000
1	-2.506900000	-0.877109000	-2.425345000
17	2.172125000	6.037857000	3.973204000
17	-5.665014000	-3.209234000	-5.313855000
6	-1.377126000	2.746457000	-0.419895000
6	-0.093656000	3.644716000	-0.499018000
1	0.665158000	3.217302000	0.159925000
6	-2.560281000	3.444141000	-1.113550000
1	-2.817585000	4.369470000	-0.592618000
1	-2.307465000	3.684854000	-2.153821000
1	-3.442226000	2.792070000	-1.117298000
6	-1.072993000	1.388997000	-1.077387000
1	-0.589767000	1.504635000	-2.062498000
1	0.217437000	0.987966000	-0.508709000
1	-2.025274000	0.901997000	-1.347594000
1	0.288111000	3.533326000	-1.526848000
6	-0.243568000	5.124526000	-0.224912000
6	-0.391860000	6.030125000	-1.284574000
6	-0.202793000	5.631396000	1.081229000
6	-0.502631000	7.402644000	-1.052433000
6	-0.307024000	7.001435000	1.318709000
6	-0.458038000	7.893122000	0.253665000
1	-0.421318000	5.654524000	-2.306048000
1	-0.105481000	4.944549000	1.911803000
1	-0.617606000	8.085954000	-1.890361000
1	-0.262924000	7.369665000	2.339763000
1	-0.536758000	8.961167000	0.440193000
11	1.973296000	0.743074000	-2.366197000

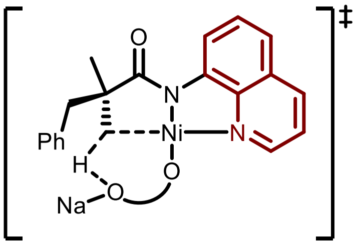

TS23_Minor

E (6-311+G**/LANL2DZ): -3591.984515 A.U.

G_{corr} (6-31G*/LANL2DZ): 0.663052 A.U.

28	-1.244114000	-0.166342000	0.408148000
7	-1.565190000	1.108227000	1.742335000
7	-1.476745000	-1.455519000	1.815690000
8	-1.714977000	3.339730000	2.314767000
6	-1.602194000	2.453094000	1.467114000
6	-1.677670000	0.572747000	3.020477000
6	-1.837859000	1.223483000	4.243890000
6	-1.892546000	0.472602000	5.442668000
6	-1.802244000	-0.904583000	5.463545000
6	-1.663951000	-1.606420000	4.238565000
6	-1.607232000	-0.853630000	3.035826000
6	-1.574801000	-3.015872000	4.115114000
6	-1.453856000	-3.596018000	2.870204000

6	-1.406413000	-2.774789000	1.725904000
1	-1.295387000	-3.169390000	0.723531000
1	-1.370694000	-4.670628000	2.754108000
1	-1.601363000	-3.629283000	5.012213000
1	-1.842192000	-1.458842000	6.396956000
1	-2.008463000	1.013310000	6.378554000
1	-1.912344000	2.300947000	4.260892000
6	4.846068000	-2.440628000	-2.307604000
6	3.791622000	-2.126481000	-1.477248000
6	2.445457000	-2.155645000	-1.954149000
6	2.229479000	-2.515387000	-3.329319000
6	3.340759000	-2.831349000	-4.156654000
6	4.625736000	-2.798060000	-3.660972000
1	5.860123000	-2.418802000	-1.917413000
1	3.970539000	-1.863205000	-0.440359000
6	1.330170000	-1.801301000	-1.127638000
6	0.897130000	-2.512745000	-3.823741000
1	3.154816000	-3.107177000	-5.192550000
1	5.467591000	-3.046825000	-4.300589000
6	-0.190943000	-2.178228000	-3.037499000
6	0.013280000	-1.799761000	-1.641543000
1	0.735384000	-2.765541000	-4.869604000
6	1.579839000	-1.337373000	0.270732000
6	1.778209000	-2.264917000	1.337385000
6	1.621835000	0.034214000	0.515930000
6	1.754164000	-3.672590000	1.139918000
6	1.973199000	-1.764543000	2.665839000
6	1.901390000	0.536446000	1.831423000
6	1.907374000	-4.539840000	2.200945000
1	1.607459000	-4.055965000	0.134731000
6	2.116755000	-2.684458000	3.737427000
6	2.038313000	-0.361652000	2.869608000
6	2.085370000	-4.044040000	3.514582000
1	1.888277000	-5.612935000	2.028747000
1	2.253835000	-2.292819000	4.742688000
1	2.225499000	0.012945000	3.873082000
1	2.198361000	-4.736966000	4.343897000
8	-1.040177000	-1.476866000	-0.938932000
8	1.316461000	0.915646000	-0.477378000
6	2.022275000	1.997101000	2.056379000
6	2.879516000	2.776008000	1.261291000
6	1.284800000	2.631398000	3.062553000
6	2.992739000	4.148676000	1.458942000
6	1.368641000	4.009641000	3.259053000
6	2.225227000	4.756243000	2.455355000
6	-1.557025000	-2.211201000	-3.620088000
6	-2.482090000	-1.174911000	-3.403076000
6	-1.942765000	-3.277777000	-4.448219000
6	-3.737408000	-1.190917000	-4.005333000
6	-3.199089000	-3.312457000	-5.052543000
6	-4.087801000	-2.262819000	-4.827845000
1	-4.441251000	-0.382541000	-3.837305000
1	-3.488970000	-4.146130000	-5.683148000
1	0.752011000	4.497440000	4.004970000
1	3.658054000	4.747462000	0.845885000

1	0.597022000	2.051156000	3.666917000
1	3.454483000	2.298680000	0.474233000
1	-1.257415000	-4.105658000	-4.606876000
1	-2.217085000	-0.352909000	-2.751350000
17	2.335153000	6.498554000	2.683473000
17	-5.674625000	-2.292689000	-5.584724000
6	-1.509551000	2.779090000	-0.035170000
6	-0.366428000	3.782199000	-0.250832000
1	0.597789000	3.319474000	-0.032562000
6	-2.877470000	3.387904000	-0.506125000
1	-2.846038000	3.396118000	-1.604268000
1	-3.670226000	2.682006000	-0.223329000
6	-1.268028000	1.477757000	-0.822098000
1	-0.887880000	1.692348000	-1.834873000
1	0.070912000	1.027408000	-0.489017000
1	-2.234539000	0.986190000	-1.029884000
1	-0.361225000	4.129259000	-1.293363000
11	1.533917000	0.361051000	-2.576155000
1	-0.492156000	4.649105000	0.400884000
6	-3.232615000	4.775876000	-0.020846000
6	-2.888017000	5.902359000	-0.780100000
6	-3.922140000	4.972522000	1.183287000
6	-3.208459000	7.190298000	-0.347143000
6	-4.249528000	6.256038000	1.618506000
6	-3.891999000	7.371053000	0.856221000
1	-2.362658000	5.767387000	-1.723177000
1	-4.183938000	4.112365000	1.791069000
1	-2.929332000	8.049517000	-0.951960000
1	-4.782185000	6.386386000	2.557104000
1	-4.147062000	8.371515000	1.196153000

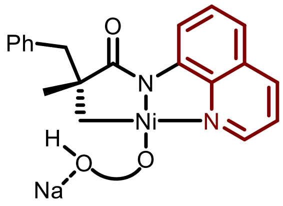

INT3

E (6-311+G**/LANL2DZ): -3592.013670 A.U.

G_{corr} (6-31G*/LANL2DZ): 0.669010 A.U.

28	-1.504797000	-0.020615000	0.177780000
7	-1.977235000	1.480998000	1.196189000
7	-1.510978000	-0.945981000	1.927490000
8	-2.828610000	3.640590000	1.113140000
6	-2.335856000	2.649913000	0.575154000
6	-2.071658000	1.268674000	2.563283000
6	-2.365533000	2.185369000	3.573925000
6	-2.371106000	1.769273000	4.924989000
6	-2.113516000	0.464559000	5.304376000
6	-1.837000000	-0.504215000	4.304696000
6	-1.798976000	-0.082475000	2.947865000
6	-1.603089000	-1.883061000	4.551157000
6	-1.360676000	-2.740428000	3.500404000
6	-1.311842000	-2.227532000	2.184593000
1	-1.088118000	-2.846702000	1.323830000
1	-1.173387000	-3.795949000	3.662125000
1	-1.626855000	-2.249831000	5.574734000
1	-2.141222000	0.165275000	6.348423000
1	-2.598455000	2.509820000	5.688182000
1	-2.590116000	3.208240000	3.307573000
6	4.854562000	-0.997326000	-2.626127000

6	3.854303000	-1.125415000	-1.686222000
6	2.508903000	-1.386091000	-2.077658000
6	2.231034000	-1.533210000	-3.477891000
6	3.284242000	-1.388873000	-4.420216000
6	4.572179000	-1.122975000	-4.008562000
1	5.873600000	-0.801518000	-2.302883000
1	4.082027000	-1.033662000	-0.629092000
6	1.427040000	-1.460736000	-1.143760000
6	0.902002000	-1.840002000	-3.874927000
1	3.055014000	-1.505198000	-5.477373000
1	5.371143000	-1.019589000	-4.737115000
6	-0.148322000	-1.962597000	-2.981059000
6	0.089260000	-1.673684000	-1.566717000
1	0.711376000	-1.978734000	-4.936726000
6	1.693066000	-1.316235000	0.319930000
6	1.740596000	-2.469325000	1.164490000
6	1.873261000	-0.072881000	0.894082000
6	1.582747000	-3.785336000	0.650909000
6	1.928807000	-2.306743000	2.575256000
6	2.021557000	0.117520000	2.299632000
6	1.612821000	-4.879059000	1.488205000
1	1.433071000	-3.914221000	-0.415713000
6	1.965641000	-3.455456000	3.410305000
6	2.036688000	-0.999725000	3.108703000
6	1.809392000	-4.716727000	2.882231000
1	1.487962000	-5.876749000	1.076412000
1	2.107850000	-3.316645000	4.479358000
1	2.139967000	-0.872783000	4.183068000
1	1.836032000	-5.588956000	3.529501000
8	-0.915313000	-1.619016000	-0.734309000
8	1.868913000	1.034955000	0.055187000
6	2.069093000	1.486856000	2.876915000
6	3.029761000	2.431751000	2.470453000
6	1.126943000	1.865821000	3.847331000
6	3.054200000	3.715234000	3.018371000
6	1.143252000	3.142043000	4.404211000
6	2.111919000	4.056221000	3.988110000
6	-1.474353000	-2.424860000	-3.467213000
6	-2.692815000	-1.950348000	-2.948257000
6	-1.532098000	-3.384279000	-4.496385000
6	-3.913793000	-2.387361000	-3.458360000
6	-2.745173000	-3.833236000	-5.013095000
6	-3.932946000	-3.323372000	-4.491152000
1	-4.845228000	-2.005041000	-3.053973000
1	-2.770806000	-4.578643000	-5.800835000
1	0.400967000	3.425270000	5.141947000
1	3.801182000	4.437145000	2.707325000
1	0.365174000	1.161216000	4.159658000
1	3.790161000	2.150660000	1.745713000
1	-0.612251000	-3.811822000	-4.884422000
1	-2.682521000	-1.241934000	-2.135349000
17	2.149399000	5.660630000	4.703086000
17	-5.472928000	-3.881949000	-5.133626000
6	-1.974055000	2.610020000	-0.920441000
6	-0.525056000	3.185184000	-1.037376000

1	0.114371000	2.489565000	-0.485541000
6	-2.952830000	3.468088000	-1.728413000
1	-2.983672000	4.490568000	-1.341307000
1	-2.661682000	3.491147000	-2.786906000
1	-3.964389000	3.053448000	-1.664043000
6	-1.949231000	1.129681000	-1.346358000
1	-1.457136000	0.996395000	-2.332669000
1	1.835683000	1.838793000	0.606294000
1	-2.982435000	0.800330000	-1.533133000
1	-0.251637000	3.157138000	-2.108759000
6	-0.258430000	4.572680000	-0.506039000
6	-0.354678000	5.698229000	-1.333418000
6	0.097014000	4.752722000	0.838565000
6	-0.108298000	6.976934000	-0.830481000
6	0.345278000	6.028028000	1.344251000
6	0.243069000	7.144087000	0.510292000
1	-0.630579000	5.570853000	-2.378007000
1	0.159950000	3.890479000	1.499183000
1	-0.189244000	7.840126000	-1.486018000
1	0.614987000	6.150919000	2.388234000
1	0.437187000	8.138105000	0.904311000
11	0.745783000	0.896145000	-1.971230000

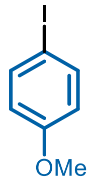

Iodoanisole

E (6-311+G**/LANL2DZ): -357.6544014 A.U.

G_{corr} (6-31G*/LANL2DZ): 0.087812 A.U.

6	-1.899454000	-0.024873000	-2.920884000
6	-3.114567000	-0.016126000	-2.234931000
6	-0.684341000	-0.016126000	-2.234931000
6	-3.110164000	-0.004781000	-0.838915000
6	-0.688745000	-0.004781000	-0.838915000
6	-1.899454000	0.003018000	-0.142163000
1	-4.055327000	-0.015591000	-2.774534000
1	0.256419000	-0.015590000	-2.774534000
1	-4.041201000	0.011989000	-0.280639000
1	0.242293000	0.011990000	-0.280639000
8	-1.899454000	0.052981000	1.234681000
6	-1.899454000	-1.227634000	1.862577000
1	-2.792801000	-1.807516000	1.589517000
1	-1.006108000	-1.807516000	1.589517000
1	-1.899454000	-1.047189000	2.940109000
53	-1.899454000	-0.043044000	-5.069214000

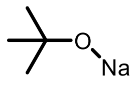

***t*BuONa**

E (6-311+G**): -395.4843634 A.U.

G_{corr} (6-31G*): 0.091672 A.U.

6	-1.316309000	3.894231000	-0.645256000
6	-1.372966000	3.846160000	0.899693000
1	-2.259263000	3.282162000	1.215791000
1	-0.486554000	3.324992000	1.282067000
1	-1.414180000	4.844066000	1.357398000
8	-1.266309000	2.613784000	-1.151909000
6	-2.575265000	4.620166000	-1.175034000
1	-2.545572000	4.650870000	-2.271268000
1	-3.471648000	4.062484000	-0.876678000

1	-2.664949000	5.649096000	-0.800514000
6	-0.058090000	4.681278000	-1.080882000
1	0.839526000	4.167532000	-0.714984000
1	-0.007084000	4.712032000	-2.176320000
1	-0.046811000	5.712821000	-0.702956000
11	-1.198647000	0.814498000	-1.860888000

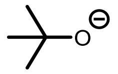

***t*BuO$^-$**

E (6-311+G**): -233.2025972 A.U.

G_{corr} (6-31G*): 0.09191 A.U.

6	-1.297463000	3.819394000	-0.649488000
6	-1.365825000	3.867163000	0.927091000
1	-2.243330000	3.291063000	1.253359000
1	-0.475925000	3.361415000	1.327888000
1	-1.424825000	4.877730000	1.373296000
8	-1.228600000	2.580204000	-1.110754000
6	-2.573466000	4.583935000	-1.179177000
1	-2.543009000	4.588329000	-2.277961000
1	-3.466071000	4.016677000	-0.879543000
1	-2.686966000	5.626769000	-0.827848000
6	-0.046088000	4.684678000	-1.072496000
1	0.860377000	4.189147000	-0.696892000
1	0.015797000	4.690336000	-2.169965000
1	-0.045784000	5.732043000	-0.716331000

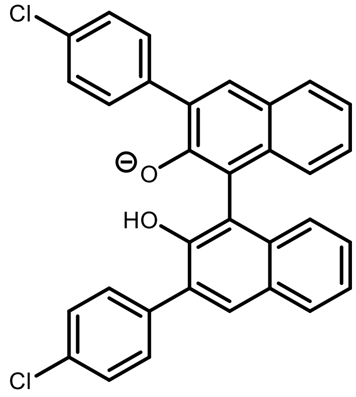

Binolate

E (6-311+G**): -2302.315709 A.U.

G_{corr} (6-31G*): 0.351346 A.U.

6	-0.119437000	1.470797000	0.714174000
6	0.186824000	0.994608000	1.968527000
6	-0.772459000	0.273890000	2.731590000
6	-2.069980000	0.057735000	2.162549000
6	-2.356161000	0.566926000	0.866059000
6	-1.404308000	1.258062000	0.152656000
1	0.628379000	2.021836000	0.148350000
1	1.165335000	1.162328000	2.403904000
6	-0.476373000	-0.236454000	4.028919000
6	-3.033094000	-0.659095000	2.910107000
1	-3.347135000	0.397501000	0.447717000
1	-1.633589000	1.643284000	-0.838385000
6	-2.768302000	-1.148562000	4.178028000
6	-1.469605000	-0.915003000	4.727020000
1	-4.023967000	-0.797813000	2.483123000
6	0.857143000	-0.068414000	4.676623000
6	1.756907000	-1.164665000	4.767734000
6	1.195267000	1.258060000	5.138132000
6	1.466298000	-2.469930000	4.260716000
6	3.039999000	-0.977079000	5.392275000
6	2.509344000	1.390744000	5.818648000
6	2.371381000	-3.503754000	4.371934000
1	0.510970000	-2.636839000	3.772068000
6	3.945605000	-2.060033000	5.484622000
6	3.359926000	0.310701000	5.898381000
6	3.629466000	-3.310560000	4.991197000
1	2.113907000	-4.483616000	3.973357000

1	4.909853000	-1.885171000	5.961484000
1	4.345692000	0.437454000	6.345835000
1	4.334380000	-4.134471000	5.072749000
8	-1.212122000	-1.403962000	5.975567000
8	0.425482000	2.239979000	4.978056000
6	2.913937000	2.689469000	6.402489000
6	2.527645000	3.919926000	5.830742000
6	3.720799000	2.740736000	7.556395000
6	2.954381000	5.134227000	6.365378000
6	4.156810000	3.946761000	8.102238000
6	3.770595000	5.138252000	7.494266000
6	-3.817448000	-1.889390000	4.921742000
6	-4.048848000	-1.682421000	6.293687000
6	-4.647184000	-2.804146000	4.250449000
6	-5.073611000	-2.346807000	6.963663000
6	-5.677716000	-3.477130000	4.905291000
6	-5.882799000	-3.238278000	6.261403000
1	-5.245795000	-2.173188000	8.020897000
1	-6.305513000	-4.185267000	4.374166000
1	4.774433000	3.962884000	8.994804000
1	2.654658000	6.072435000	5.908753000
1	3.996251000	1.815340000	8.054096000
1	1.868415000	3.902543000	4.973306000
1	-4.469426000	-3.006504000	3.198244000
1	-3.413518000	-0.994845000	6.838271000
17	4.313516000	6.683015000	8.180441000
17	-7.184671000	-4.087847000	7.107717000
1	-0.298872000	-1.107736000	6.178773000

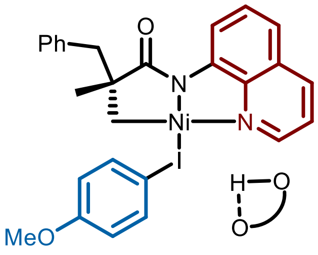

INT4

E (6-311+G**/LANL2DZ): -3787.419515 A.U.

G_{corr} (6-31G*/LANL2DZ): 0.78112 A.U.

1	-2.075444000	-3.932885000	-1.606412000
1	-3.785244000	-2.222080000	-2.248955000
1	-3.367560000	0.170664000	-1.673140000
28	-1.019991000	1.671207000	-0.078599000
6	0.260580000	-3.375515000	-0.275819000
6	1.356928000	-2.880953000	0.400436000
1	2.149910000	-3.559954000	0.705023000
6	1.492998000	-1.511903000	0.720944000
1	2.363161000	-1.151418000	1.249777000
6	-0.107956000	3.239920000	0.583842000
6	1.341562000	1.440295000	1.425190000
8	2.423219000	0.985887000	1.810716000
7	0.419438000	0.747399000	0.675499000
6	0.491526000	-0.601695000	0.373501000
6	-0.637546000	-1.100542000	-0.358962000
6	-0.763367000	-2.481470000	-0.679519000
6	-1.929803000	-2.881055000	-1.376698000
6	-2.870322000	-1.943010000	-1.737887000
6	-2.651290000	-0.593776000	-1.400444000
7	-1.584047000	-0.181949000	-0.729459000
1	0.167814000	-4.432732000	-0.509317000
53	-2.993904000	3.110739000	-1.196951000
6	0.821130000	2.848885000	1.744746000

6	0.000795000	2.764778000	3.051463000
1	0.611195000	2.433559000	3.893838000
1	-0.412508000	3.751606000	3.293075000
1	-0.828351000	2.060927000	2.941716000
6	1.989902000	3.880167000	1.875420000
1	1.587632000	4.850670000	1.558948000
1	2.762366000	3.607456000	1.146055000
1	0.469677000	3.591727000	-0.287070000
1	-0.800102000	4.038317000	0.882196000
6	-4.093311000	3.618750000	0.592983000
6	-5.296956000	4.312319000	0.478454000
6	-3.583271000	3.229144000	1.821904000
6	-5.989265000	4.634402000	1.641474000
1	-5.706987000	4.578812000	-0.489176000
6	-4.277654000	3.567406000	2.983791000
1	-2.671153000	2.651411000	1.901806000
6	-5.483262000	4.271680000	2.899791000
1	-6.940265000	5.155114000	1.593566000
1	-3.879682000	3.216531000	3.924504000
6	2.613215000	4.056339000	3.245712000
6	2.283588000	5.173933000	4.025039000
6	3.512191000	3.119040000	3.777482000
6	2.826772000	5.354554000	5.299097000
6	4.057761000	3.297205000	5.048647000
6	3.717783000	4.413951000	5.816679000
1	1.586081000	5.909679000	3.629139000
1	3.746266000	2.237250000	3.192465000
1	2.549655000	6.227380000	5.886220000
1	4.736905000	2.549975000	5.449633000
1	4.136643000	4.542851000	6.811751000
8	-6.231087000	4.639444000	3.973405000
6	-5.757059000	4.279549000	5.274329000
1	-5.667983000	3.193637000	5.387800000
1	-4.791231000	4.760261000	5.481902000
1	-6.511924000	4.648946000	5.969731000
6	-5.322816000	-3.576063000	1.008085000
6	-5.418698000	-2.351830000	1.634737000
6	-4.294666000	-1.743254000	2.265975000
6	-3.061708000	-2.477315000	2.254212000
6	-2.992348000	-3.734983000	1.597297000
6	-4.094816000	-4.278958000	0.977726000
1	-6.205979000	-4.011578000	0.545896000
1	-6.372549000	-1.839414000	1.665474000
6	-4.363547000	-0.466380000	2.929482000
6	-1.943523000	-1.962167000	2.952087000
1	-2.039614000	-4.260122000	1.597058000
1	-4.029573000	-5.245523000	0.482894000
6	-2.029800000	-0.810297000	3.708024000
6	-3.259923000	-0.065031000	3.700950000
1	-1.020458000	-2.536328000	2.947397000
6	-5.593104000	0.383999000	2.826186000
6	-6.132389000	0.732751000	1.545629000
6	-6.216291000	0.879632000	4.006338000
6	-5.466508000	0.457615000	0.315098000
6	-7.389318000	1.422790000	1.456294000

6	-7.509230000	1.547173000	3.893059000
6	-6.020879000	0.789978000	-0.901816000
1	-4.492932000	-0.013049000	0.353229000
6	-7.934712000	1.751368000	0.190100000
6	-8.057916000	1.776514000	2.652150000
6	-7.273770000	1.441751000	-0.978846000
1	-5.484215000	0.562896000	-1.820458000
1	-8.891541000	2.270723000	0.161733000
1	-9.034050000	2.251567000	2.572796000
1	-7.700418000	1.700447000	-1.944892000
8	-3.312986000	1.042778000	4.465023000
8	-5.658674000	0.783205000	5.177751000
6	-8.226952000	2.001686000	5.107448000
6	-8.233613000	1.248056000	6.296452000
6	-8.941914000	3.213419000	5.099061000
6	-8.946974000	1.669204000	7.416940000
6	-9.658806000	3.650796000	6.212674000
6	-9.658607000	2.867158000	7.364030000
6	-0.868870000	-0.384520000	4.534546000
6	-1.019922000	0.086067000	5.851630000
6	0.433422000	-0.490109000	4.023429000
6	0.087992000	0.414695000	6.629593000
6	1.553170000	-0.147525000	4.779867000
6	1.367640000	0.289946000	6.087977000
1	-0.038075000	0.764638000	7.649036000
1	2.541961000	-0.189803000	4.338594000
1	-10.196465000	4.593377000	6.192871000
1	-8.949030000	1.075942000	8.325731000
1	-8.892603000	3.848593000	4.220794000
1	-7.655479000	0.334008000	6.339948000
1	0.579593000	-0.815857000	3.002133000
1	-2.014005000	0.197788000	6.264342000
17	-10.559650000	3.411539000	8.788155000
17	2.770394000	0.688216000	7.087626000
1	-4.282034000	1.022277000	4.879420000

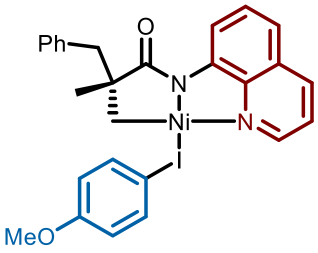

INT4‘

E (6-311+G**/LANL2DZ): -1485.045905 A.U.

G_{corr} (6-31G*/LANL2DZ): 0.395079 A.U.

1	0.259695000	5.055760000	0.672928000
1	2.013119000	3.530432000	1.602570000
1	1.712738000	1.066238000	1.376278000
28	-0.568641000	-0.753880000	-0.023092000
6	-2.039807000	4.211912000	-0.566965000
6	-3.120010000	3.576171000	-1.144476000
1	-1.978992000	5.295491000	-0.519789000
1	-3.928536000	4.169732000	-1.563648000
6	-3.221670000	2.169553000	-1.214166000
1	-4.080712000	1.699510000	-1.670205000
6	-3.151830000	-0.864872000	-1.105722000
8	-4.156341000	-0.507657000	-1.720993000
7	-2.164058000	-0.022734000	-0.644051000
6	-2.211528000	1.361037000	-0.689953000
6	-1.077405000	2.008364000	-0.097040000
6	-0.990026000	3.430343000	-0.023501000

6	0.158447000	3.975487000	0.600961000
6	1.126818000	3.139702000	1.113444000
6	0.960498000	1.745849000	0.992456000
7	-0.091610000	1.194257000	0.401975000
6	-2.841979000	-2.313889000	-0.706297000
6	-1.311194000	-2.426835000	-0.644648000
1	-1.004842000	-3.277617000	-0.017753000
1	-0.879369000	-2.570909000	-1.647675000
6	-3.424290000	-2.530540000	0.708471000
1	-2.921001000	-1.868108000	1.421385000
1	-4.499199000	-2.331327000	0.734201000
53	1.793577000	-1.879880000	0.544127000
6	2.996567000	-0.486513000	-0.568027000
6	2.424829000	0.175375000	-1.658013000
6	4.306274000	-0.238951000	-0.177568000
6	3.186302000	1.101204000	-2.358755000
1	1.394304000	-0.010847000	-1.942442000
6	5.072421000	0.691863000	-0.888281000
1	4.744554000	-0.752611000	0.671824000
6	4.513197000	1.366313000	-1.981516000
1	2.770088000	1.635891000	-3.206297000
1	6.093036000	0.877390000	-0.575313000
1	-3.265760000	-3.568785000	1.024192000
6	-3.431266000	-3.336721000	-1.731212000
1	-2.763300000	-4.207046000	-1.728041000
1	-3.359235000	-2.889635000	-2.729695000
6	-4.844471000	-3.830422000	-1.488211000
6	-5.966383000	-3.036761000	-1.773929000
6	-5.055468000	-5.110693000	-0.957199000
6	-7.255203000	-3.514106000	-1.532466000
6	-6.344162000	-5.590828000	-0.713415000
6	-7.451502000	-4.791915000	-1.001376000
1	-5.809863000	-2.037073000	-2.162768000
1	-4.197131000	-5.741597000	-0.733979000
1	-8.111543000	-2.883662000	-1.760267000
1	-6.481061000	-6.588107000	-0.302056000
1	-8.457142000	-5.160864000	-0.815470000
8	5.163043000	2.290456000	-2.736615000
6	6.511649000	2.602899000	-2.414996000
1	6.597452000	3.021742000	-1.403240000
1	7.160072000	1.720457000	-2.498463000
1	6.825996000	3.351979000	-3.143422000

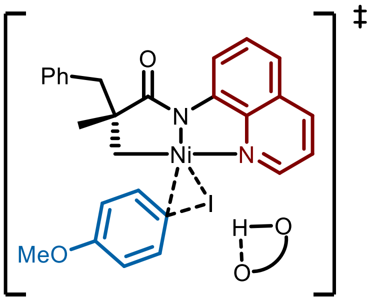

TS45

E (6-311+G**/LANL2DZ): -3787.387925 A.U.

G_{corr} (6-31G*/LANL2DZ): 0.775669 A.U.

1	0.484998000	-4.485620000	1.050897000
1	-1.610863000	-3.780889000	-0.123514000
1	-2.119177000	-1.356741000	-0.341504000
28	-0.392708000	1.360608000	0.255494000
6	2.357273000	-2.754098000	2.053607000
6	3.186619000	-1.721895000	2.441524000
1	4.106220000	-1.944540000	2.976940000
6	2.894477000	-0.372678000	2.148696000
1	3.582328000	0.410638000	2.429434000

6	0.284310000	3.098624000	-0.276753000
6	2.092930000	2.362524000	1.185378000
8	3.261577000	2.394355000	1.577704000
7	1.309149000	1.237827000	1.086670000
6	1.721963000	-0.029330000	1.471455000
6	0.822933000	-1.085698000	1.115452000
6	1.156049000	-2.450569000	1.365481000
6	0.254478000	-3.435123000	0.891814000
6	-0.899119000	-3.051977000	0.248111000
6	-1.184394000	-1.677653000	0.104010000
7	-0.355888000	-0.725161000	0.512314000
1	2.605403000	-3.791214000	2.261368000
53	-2.490661000	1.401481000	-1.283381000
6	1.304657000	3.599232000	0.746140000
6	0.605427000	4.164037000	2.000013000
1	1.340962000	4.451241000	2.756336000
1	0.017642000	5.052735000	1.742516000
1	-0.072391000	3.420615000	2.425499000
6	2.232472000	4.666641000	0.072194000
1	1.625376000	5.186903000	-0.679469000
1	3.015903000	4.129092000	-0.474560000
1	0.769608000	2.838425000	-1.228510000
1	-0.511417000	3.821732000	-0.467825000
6	-2.226441000	2.027750000	1.069075000
6	-2.543781000	3.385131000	1.229807000
6	-2.564756000	1.099706000	2.056463000
6	-3.074640000	3.824014000	2.429645000
1	-2.367523000	4.094453000	0.432083000
6	-3.070059000	1.551782000	3.278887000
1	-2.436137000	0.036893000	1.903733000
6	-3.345934000	2.913145000	3.464951000
1	-3.320447000	4.870192000	2.579439000
1	-3.292415000	0.830145000	4.053248000
6	2.859784000	5.713414000	0.971492000
6	2.344389000	7.016856000	0.994065000
6	3.953732000	5.417218000	1.799865000
6	2.897917000	8.000920000	1.816282000
6	4.509727000	6.398610000	2.621228000
6	3.986336000	7.694609000	2.634163000
1	1.496408000	7.263727000	0.358097000
1	4.336650000	4.402998000	1.808246000
1	2.478480000	9.004380000	1.816261000
1	5.355329000	6.148316000	3.257901000
1	4.421405000	8.456512000	3.276590000
8	-3.891523000	3.442518000	4.583138000
6	-4.402956000	2.544780000	5.574057000
1	-5.128062000	1.850526000	5.142058000
1	-3.596449000	1.968561000	6.043940000
1	-4.885252000	3.178959000	6.320036000
6	-6.814871000	-1.646298000	-0.633082000
6	-6.826974000	-1.044202000	0.605934000
6	-5.803606000	-1.290551000	1.566215000
6	-4.781789000	-2.234335000	1.212170000
6	-4.793742000	-2.835805000	-0.075832000
6	-5.779675000	-2.543726000	-0.991085000

1	-7.612995000	-1.435011000	-1.340684000
1	-7.630768000	-0.366968000	0.869680000
6	-5.775608000	-0.649117000	2.852750000
6	-3.780937000	-2.547579000	2.162294000
1	-4.010718000	-3.551886000	-0.320495000
1	-5.776719000	-3.010681000	-1.973001000
6	-3.804371000	-2.022032000	3.438657000
6	-4.855073000	-1.117290000	3.805333000
1	-2.998546000	-3.249951000	1.892720000
6	-6.669819000	0.505381000	3.168195000
6	-6.711158000	1.654752000	2.307212000
6	-7.433841000	0.484861000	4.369881000
6	-5.850528000	1.839181000	1.183620000
6	-7.640608000	2.713916000	2.586070000
6	-8.376953000	1.569143000	4.623122000
6	-5.929954000	2.956977000	0.381890000
1	-5.098238000	1.093597000	0.965322000
6	-7.708438000	3.846690000	1.734190000
6	-8.468907000	2.614742000	3.731283000
6	-6.875847000	3.975145000	0.644194000
1	-5.242254000	3.056328000	-0.454168000
1	-8.431908000	4.625443000	1.971428000
1	-9.198995000	3.404440000	3.900666000
1	-6.931853000	4.851704000	0.003216000
8	-4.864372000	-0.661085000	5.068323000
8	-7.292023000	-0.457479000	5.254633000
6	-9.218714000	1.561183000	5.843535000
6	-9.760600000	0.372200000	6.370213000
6	-9.527465000	2.763454000	6.505991000
6	-10.594255000	0.387507000	7.486363000
6	-10.359504000	2.796704000	7.624939000
6	-10.890832000	1.602172000	8.103399000
6	-2.701041000	-2.331017000	4.383973000
6	-2.927550000	-2.690718000	5.723581000
6	-1.371993000	-2.273233000	3.932828000
6	-1.867322000	-3.009923000	6.569838000
6	-0.297487000	-2.583601000	4.766068000
6	-0.561273000	-2.956787000	6.081177000
1	-2.049695000	-3.295113000	7.600830000
1	0.721707000	-2.523163000	4.398632000
1	-10.581421000	3.733615000	8.125762000
1	-11.010603000	-0.535620000	7.876830000
1	-9.089944000	3.692531000	6.152134000
1	-9.506687000	-0.569552000	5.901629000
1	-1.171575000	-1.958527000	2.915920000
1	-3.942544000	-2.709475000	6.100394000
17	-11.946089000	1.625666000	9.525622000
17	0.784510000	-3.360601000	7.155553000
1	-5.907062000	-0.580327000	5.306096000

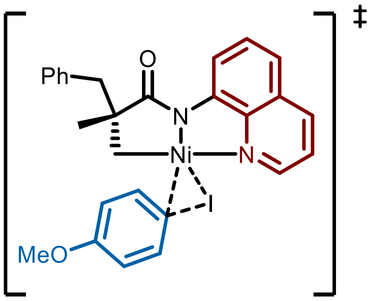

TS45'

E (6-311+G**/LANL2DZ): -1485.028806 A.U.

G_{corr} (6-31G*/LANL2DZ): 0.398154 A.U.

28	4.052295000	2.432702000	0.588935000
7	2.481715000	3.471376000	0.519616000
7	2.829345000	0.989455000	-0.289221000
8	1.451869000	5.412752000	1.260424000
6	2.451899000	4.708789000	1.124521000
6	1.381269000	2.878077000	-0.086562000
6	0.126666000	3.445672000	-0.314413000
6	-0.893996000	2.702953000	-0.947758000
6	-0.712253000	1.396955000	-1.350420000
6	0.542962000	0.777094000	-1.130727000
6	1.590669000	1.528665000	-0.516497000
6	0.824414000	-0.566838000	-1.474312000
6	2.066009000	-1.100129000	-1.203764000
6	3.044894000	-0.280683000	-0.608351000
1	4.035534000	-0.668987000	-0.394982000
1	2.306487000	-2.130778000	-1.443330000
1	0.048473000	-1.168093000	-1.941878000
1	-1.511528000	0.832333000	-1.821958000
1	-1.854785000	3.185248000	-1.108034000
1	-0.056241000	4.457749000	0.014031000
6	3.853552000	5.112777000	1.587397000
6	3.807314000	5.958376000	2.906202000
1	4.722231000	5.726519000	3.465726000
6	4.496044000	5.902497000	0.428628000
1	3.916289000	6.802039000	0.206242000
1	5.513138000	6.213938000	0.696322000
6	4.604141000	3.806983000	1.845842000
1	4.280070000	3.341345000	2.787509000
1	5.688656000	3.944202000	1.875861000
53	6.132337000	0.907670000	1.023132000
6	5.751919000	2.459348000	-0.746821000
6	5.318264000	1.949480000	-1.981326000
6	6.597413000	3.570063000	-0.712050000
6	5.612563000	2.640428000	-3.148949000
6	6.899625000	4.258573000	-1.886867000
6	6.394919000	3.805662000	-3.113371000
1	4.729080000	1.045750000	-2.031903000
1	6.996589000	3.929290000	0.227124000
1	5.246298000	2.289143000	-4.108340000
1	7.519504000	5.145223000	-1.825506000
8	6.616731000	4.403899000	-4.312921000
6	7.382686000	5.601240000	-4.339763000
1	6.914416000	6.390219000	-3.736345000
1	8.407610000	5.431691000	-3.983529000
1	7.411208000	5.913621000	-5.384921000
1	4.548794000	5.282215000	-0.469583000
1	2.970207000	5.588868000	3.509536000
6	3.716105000	7.464560000	2.761891000
6	4.857148000	8.255312000	2.956514000
6	2.512110000	8.104023000	2.428167000
6	4.806601000	9.644530000	2.821896000
6	2.458465000	9.491925000	2.293945000

6	3.603620000	10.268945000	2.489490000
1	5.797701000	7.775875000	3.221459000
1	1.631694000	7.496578000	2.252157000
1	5.704767000	10.236542000	2.980686000
1	1.516345000	9.969248000	2.035100000
1	3.557545000	11.350137000	2.385896000

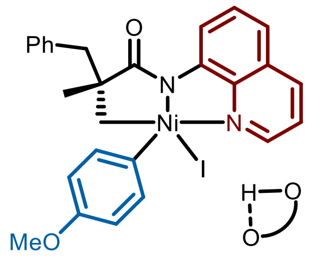

INT5

E (6-311+G**/LANL2DZ): -3787.405043 A.U.

G_{corr} (6-31G*/LANL2DZ): 0.775711 A.U.

1	-1.711348000	-3.480381000	-0.191661000
1	-3.460907000	-2.120357000	-1.321022000
1	-3.140428000	0.368450000	-1.483650000
28	-1.016627000	2.379104000	-0.206450000
6	0.522848000	-2.443682000	1.046845000
6	1.507944000	-1.714546000	1.679304000
1	2.294068000	-2.231387000	2.223687000
6	1.523000000	-0.300992000	1.667512000
1	2.293231000	0.245423000	2.190536000
6	-0.178936000	4.125710000	0.001741000
6	1.230142000	2.713413000	1.467228000
8	2.189074000	2.427817000	2.178363000
7	0.433289000	1.788388000	0.820151000
6	0.546792000	0.404590000	0.966190000
6	-0.469612000	-0.342084000	0.292305000
6	-0.510605000	-1.761759000	0.354984000
6	-1.614172000	-2.401463000	-0.258987000
6	-2.577210000	-1.653989000	-0.900240000
6	-2.424733000	-0.255426000	-0.963729000
7	-1.409330000	0.376333000	-0.391885000
1	0.509224000	-3.529337000	1.088474000
53	-2.289344000	3.127002000	-2.359102000
6	0.787559000	4.151175000	1.184399000
6	0.140058000	4.719548000	2.461342000
1	0.849377000	4.681737000	3.291963000
1	-0.149446000	5.764961000	2.300716000
1	-0.753364000	4.155650000	2.733272000
6	2.018075000	5.027890000	0.748104000
1	1.600731000	5.901768000	0.231480000
1	2.582468000	4.463958000	-0.005012000
1	0.347763000	4.135232000	-0.960335000
1	-0.927248000	4.916886000	0.019746000
6	-2.274352000	2.750089000	1.215418000
6	-3.153542000	3.826170000	1.118679000
6	-2.274896000	1.900630000	2.312888000
6	-4.058320000	4.041304000	2.153988000
1	-3.164247000	4.472580000	0.252395000
6	-3.185493000	2.126066000	3.354806000
1	-1.601427000	1.056583000	2.389314000
6	-4.070210000	3.210385000	3.283551000
1	-4.780604000	4.849001000	2.094124000
1	-3.221061000	1.422892000	4.179449000
6	2.955271000	5.523876000	1.830072000
6	2.748263000	6.781372000	2.414943000
6	4.055960000	4.767390000	2.256323000

6	3.603110000	7.267856000	3.405610000
6	4.915876000	5.251326000	3.242402000
6	4.692797000	6.502186000	3.823449000
1	1.904847000	7.386103000	2.088838000
1	4.218747000	3.785352000	1.827618000
1	3.419270000	8.244855000	3.846107000
1	5.761397000	4.646454000	3.560936000
1	5.363031000	6.876714000	4.593286000
8	-4.971302000	3.541656000	4.244496000
6	-4.892325000	2.878948000	5.506634000
1	-5.074437000	1.807603000	5.419258000
1	-3.910951000	3.046810000	5.971714000
1	-5.678185000	3.322862000	6.120089000
6	-6.266379000	-3.118665000	-0.039758000
6	-6.222161000	-2.157090000	0.946789000
6	-5.178429000	-2.125064000	1.917773000
6	-4.192159000	-3.167126000	1.852029000
6	-4.273349000	-4.154243000	0.832928000
6	-5.277821000	-4.131121000	-0.109085000
1	-7.079814000	-3.109298000	-0.761403000
1	-7.000395000	-1.405508000	0.997695000
6	-5.105718000	-1.128318000	2.952822000
6	-3.158639000	-3.198141000	2.818199000
1	-3.521881000	-4.942810000	0.818354000
1	-5.328691000	-4.894141000	-0.882269000
6	-3.126943000	-2.305387000	3.869647000
6	-4.142568000	-1.293295000	3.964125000
1	-2.404843000	-3.980440000	2.755298000
6	-6.043046000	0.037582000	2.983774000
6	-6.181255000	0.904710000	1.848532000
6	-6.757670000	0.322473000	4.176877000
6	-5.350814000	0.827071000	0.692107000
6	-7.167283000	1.947881000	1.864821000
6	-7.728023000	1.407023000	4.181038000
6	-5.523062000	1.672375000	-0.381875000
1	-4.547520000	0.100995000	0.678382000
6	-7.332777000	2.792145000	0.736662000
6	-7.931956000	2.147208000	3.039145000
6	-6.534234000	2.660630000	-0.376980000
1	-4.856107000	1.611354000	-1.235788000
1	-8.097529000	3.566196000	0.781443000
1	-8.683571000	2.934678000	3.034069000
1	-6.652886000	3.322056000	-1.231191000
8	-4.127077000	-0.500153000	5.045074000
8	-6.529517000	-0.322658000	5.290651000
6	-8.445226000	1.761742000	5.427637000
6	-8.931072000	0.788234000	6.320981000
6	-8.664325000	3.113063000	5.752765000
6	-9.632856000	1.146859000	7.469940000
6	-9.363353000	3.489442000	6.900099000
6	-9.847234000	2.496645000	7.747831000
6	-2.042173000	-2.389262000	4.880871000
6	-2.292872000	-2.322655000	6.263007000
6	-0.715667000	-2.570526000	4.460523000
6	-1.259377000	-2.454634000	7.188203000

6	0.331682000	-2.696840000	5.372568000
6	0.046249000	-2.642453000	6.733963000
1	-1.462261000	-2.409636000	8.253236000
1	1.354102000	-2.820404000	5.030134000
1	-9.517902000	4.536626000	7.139562000
1	-10.009051000	0.387067000	8.147540000
1	-8.253840000	3.884173000	5.107110000
1	-8.737794000	-0.255988000	6.111950000
1	-0.494960000	-2.584190000	3.399531000
1	-3.305293000	-2.157682000	6.608540000
17	-10.733453000	2.956412000	
9.210709000			
17	1.361047000	-2.804153000	7.905997000
1	-5.187137000	-0.406826000	5.300665000

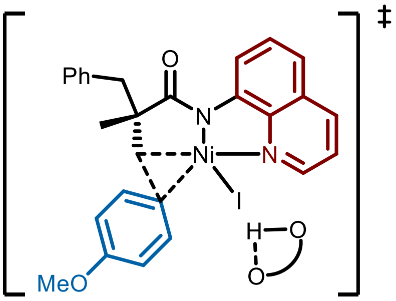

TS56

E (6-311+G**/LANL2DZ): -3787.400831 A.U.

G_{corr} (6-31G*/LANL2DZ): 0.778461 A.U.

1	0.484998000	-4.485620000	1.050897000
1	-1.610863000	-3.780889000	-0.123514000
1	-2.119177000	-1.356741000	-0.341504000
28	-0.392708000	1.360608000	0.255494000
6	2.357273000	-2.754098000	2.053607000
6	3.186619000	-1.721895000	2.441524000
1	4.106220000	-1.944540000	2.976940000
6	2.894477000	-0.372678000	2.148696000
1	3.582328000	0.410638000	2.429434000
6	0.284310000	3.098624000	-0.276753000
6	2.092930000	2.362524000	1.185378000
8	3.261577000	2.394355000	1.577704000
7	1.309149000	1.237827000	1.086670000
6	1.721963000	-0.029330000	1.471455000
6	0.822933000	-1.085698000	1.115452000
6	1.156049000	-2.450569000	1.365481000
6	0.254478000	-3.435123000	0.891814000
6	-0.899119000	-3.051977000	0.248111000
6	-1.184394000	-1.677653000	0.104010000
7	-0.355888000	-0.725161000	0.512314000
1	2.605403000	-3.791214000	2.261368000
53	-2.490661000	1.401481000	-1.283381000
6	1.304657000	3.599232000	0.746140000
6	0.605427000	4.164037000	2.000013000
1	1.340962000	4.451241000	2.756336000
1	0.017642000	5.052735000	1.742516000
1	-0.072391000	3.420615000	2.425499000
6	2.232472000	4.666641000	0.072194000
1	1.625376000	5.186903000	-0.679469000
1	3.015903000	4.129092000	-0.474560000
1	0.769608000	2.838425000	-1.228510000
1	-0.511417000	3.821732000	-0.467825000
6	-2.226441000	2.027750000	1.069075000
6	-2.543781000	3.385131000	1.229807000
6	-2.564756000	1.099706000	2.056463000
6	-3.074640000	3.824014000	2.429645000
1	-2.367523000	4.094453000	0.432083000

6	-3.070059000	1.551782000	3.278887000
1	-2.436137000	0.036893000	1.903733000
6	-3.345934000	2.913145000	3.464951000
1	-3.320447000	4.870192000	2.579439000
1	-3.292415000	0.830145000	4.053248000
6	2.859784000	5.713414000	0.971492000
6	2.344389000	7.016856000	0.994065000
6	3.953732000	5.417218000	1.799865000
6	2.897917000	8.000920000	1.816282000
6	4.509727000	6.398610000	2.621228000
6	3.986336000	7.694609000	2.634163000
1	1.496408000	7.263727000	0.358097000
1	4.336650000	4.402998000	1.808246000
1	2.478480000	9.004380000	1.816261000
1	5.355329000	6.148316000	3.257901000
1	4.421405000	8.456512000	3.276590000
8	-3.891523000	3.442518000	4.583138000
6	-4.402956000	2.544780000	5.574057000
1	-5.128062000	1.850526000	5.142058000
1	-3.596449000	1.968561000	6.043940000
1	-4.885252000	3.178959000	6.320036000
6	-6.814871000	-1.646298000	-0.633082000
6	-6.826974000	-1.044202000	0.605934000
6	-5.803606000	-1.290551000	1.566215000
6	-4.781789000	-2.234335000	1.212170000
6	-4.793742000	-2.835805000	-0.075832000
6	-5.779675000	-2.543726000	-0.991085000
1	-7.612995000	-1.435011000	-1.340684000
1	-7.630768000	-0.366968000	0.869680000
6	-5.775608000	-0.649117000	2.852750000
6	-3.780937000	-2.547579000	2.162294000
1	-4.010718000	-3.551886000	-0.320495000
1	-5.776719000	-3.010681000	-1.973001000
6	-3.804371000	-2.022032000	3.438657000
6	-4.855073000	-1.117290000	3.805333000
1	-2.998546000	-3.249951000	1.892720000
6	-6.669819000	0.505381000	3.168195000
6	-6.711158000	1.654752000	2.307212000
6	-7.433841000	0.484861000	4.369881000
6	-5.850528000	1.839181000	1.183620000
6	-7.640608000	2.713916000	2.586070000
6	-8.376953000	1.569143000	4.623122000
6	-5.929954000	2.956977000	0.381890000
1	-5.098238000	1.093597000	0.965322000
6	-7.708438000	3.846690000	1.734190000
6	-8.468907000	2.614742000	3.731283000
6	-6.875847000	3.975145000	0.644194000
1	-5.242254000	3.056328000	-0.454168000
1	-8.431908000	4.625443000	1.971428000
1	-9.198995000	3.404440000	3.900666000
1	-6.931853000	4.851704000	0.003216000
8	-4.864372000	-0.661085000	5.068323000
8	-7.292023000	-0.457479000	5.254633000
6	-9.218714000	1.561183000	5.843535000
6	-9.760600000	0.372200000	6.370213000

6	-9.527465000	2.763454000	6.505991000
6	-10.594255000	0.387507000	7.486363000
6	-10.359504000	2.796704000	7.624939000
6	-10.890832000	1.602172000	8.103399000
6	-2.701041000	-2.331017000	4.383973000
6	-2.927550000	-2.690718000	5.723581000
6	-1.371993000	-2.273233000	3.932828000
6	-1.867322000	-3.009923000	6.569838000
6	-0.297487000	-2.583601000	4.766068000
6	-0.561273000	-2.956787000	6.081177000
1	-2.049695000	-3.295113000	7.600830000
1	0.721707000	-2.523163000	4.398632000
1	-10.581421000	3.733615000	8.125762000
1	-11.010603000	-0.535620000	7.876830000
1	-9.089944000	3.692531000	6.152134000
1	-9.506687000	-0.569552000	5.901629000
1	-1.171575000	-1.958527000	2.915920000
1	-3.942544000	-2.709475000	6.100394000
17	-11.946089000	1.625666000	9.525622000
17	0.784510000	-3.360601000	7.155553000
1	-5.907062000	-0.580327000	5.306096000

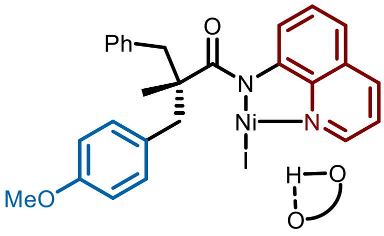

INT6

E (6-311+G**/LANL2DZ): -3787.456739 A.U.

G_{corr} (6-31G*/LANL2DZ): 0.782066 A.U.

1	-2.101562000	-3.031254000	-1.665032000
1	-3.941476000	-1.401647000	-2.139942000
1	-3.710322000	0.941585000	-1.328209000
28	-1.661687000	2.351127000	0.388838000
6	0.101008000	-2.482827000	-0.132722000
6	1.088096000	-2.012751000	0.705073000
1	1.893887000	-2.674568000	1.012875000
6	1.089391000	-0.690957000	1.204709000
1	1.869737000	-0.371631000	1.875838000
6	-0.230038000	4.471480000	1.448728000
6	0.859437000	2.160202000	2.023134000
8	1.701682000	1.535622000	2.679879000
7	-0.054683000	1.562665000	1.189264000
6	0.082069000	0.211189000	0.846268000
6	-0.937801000	-0.274672000	-0.026619000
6	-0.949769000	-1.611569000	-0.510147000
6	-2.051418000	-2.008258000	-1.305010000
6	-3.061324000	-1.114448000	-1.576705000
6	-2.955905000	0.203926000	-1.101823000
7	-1.922614000	0.610721000	-0.371119000
1	0.095596000	-3.509601000	-0.487713000
53	-2.567631000	3.635073000	-1.767421000
6	0.944602000	3.698192000	2.074957000
6	1.159674000	4.138366000	3.535863000
1	1.985823000	3.586413000	3.985475000
1	1.388260000	5.210341000	3.574471000
1	0.264570000	3.962977000	4.140088000
6	2.195062000	4.088028000	1.196378000
1	2.184459000	5.183385000	1.116433000
1	2.015096000	3.700032000	0.185657000
1	-0.127012000	4.481101000	0.355886000

1	-0.106812000	5.521317000	1.751586000
6	-1.666292000	4.064429000	1.795781000
6	-2.689978000	4.939605000	1.313140000
6	-2.046364000	3.185479000	2.851619000
6	-3.972817000	4.912734000	1.802614000
1	-2.429872000	5.657074000	0.543252000
6	-3.343015000	3.143576000	3.358213000
1	-1.317931000	2.518604000	3.296344000
6	-4.313680000	4.025679000	2.847293000
1	-4.742822000	5.571126000	1.415646000
1	-3.584195000	2.444490000	4.150879000
6	3.560043000	3.646470000	1.670078000
6	4.313908000	4.449769000	2.535828000
6	4.116412000	2.435102000	1.236255000
6	5.578960000	4.050477000	2.971314000
6	5.381346000	2.033455000	1.662457000
6	6.117649000	2.838980000	2.535111000
1	3.902407000	5.398105000	2.873633000
1	3.541964000	1.796179000	0.571301000
1	6.143772000	4.687973000	3.647291000
1	5.791447000	1.087835000	1.316397000
1	7.103393000	2.525573000	2.869987000
8	-5.572251000	4.129759000	3.291246000
6	-5.961474000	3.404135000	4.466420000
1	-5.770146000	2.338254000	4.358148000
1	-5.423873000	3.788180000	5.342135000
1	-7.032944000	3.572502000	4.571218000
6	-5.145788000	-3.209848000	0.580034000
6	-5.465964000	-2.134105000	1.380469000
6	-4.533939000	-1.607723000	2.320278000
6	-3.224990000	-2.194419000	2.365355000
6	-2.929145000	-3.305298000	1.532118000
6	-3.869167000	-3.816107000	0.664318000
1	-5.887793000	-3.607948000	-0.108550000
1	-6.455406000	-1.693401000	1.332138000
6	-4.878976000	-0.575137000	3.252252000
6	-2.270192000	-1.675212000	3.275575000
1	-1.936517000	-3.745111000	1.594611000
1	-3.633401000	-4.677991000	0.043816000
6	-2.614912000	-0.730884000	4.224471000
6	-3.967201000	-0.255458000	4.261520000
1	-1.257066000	-2.069230000	3.252684000
6	-6.195857000	0.122639000	3.186386000
6	-6.533003000	0.914745000	2.047590000
6	-7.052025000	0.087402000	4.322559000
6	-5.602426000	1.234627000	1.013083000
6	-7.840359000	1.503563000	1.960967000
6	-8.397580000	0.642090000	4.189855000
6	-5.949524000	2.062244000	-0.033830000
1	-4.588264000	0.856051000	1.089348000
6	-8.178983000	2.312629000	0.846435000
6	-8.751080000	1.295149000	3.027072000
6	-7.256731000	2.595458000	-0.137082000
1	-5.203772000	2.347755000	-0.770093000
1	-9.182033000	2.734675000	0.800925000

1	-9.759694000	1.689830000	2.916008000
1	-7.515156000	3.241303000	-0.972252000
8	-4.335577000	0.560682000	5.274418000
8	-6.641872000	-0.372442000	5.462828000
6	-9.359629000	0.542065000	5.312518000
6	-9.392204000	-0.580723000	6.163459000
6	-10.296110000	1.564339000	5.554066000
6	-10.333596000	-0.690541000	7.184301000
6	-11.244921000	1.470869000	6.571968000
6	-11.257188000	0.335951000	7.378205000
6	-1.594266000	-0.185263000	5.155702000
6	-1.838578000	-0.003357000	6.529111000
6	-0.334279000	0.180390000	4.655851000
6	-0.857294000	0.519699000	7.368823000
6	0.656121000	0.716875000	5.479633000
6	0.380186000	0.879474000	6.833773000
1	-1.051715000	0.654510000	8.427958000
1	1.604825000	1.019859000	5.053327000
1	-11.955620000	2.272601000	6.745274000
1	-10.350675000	-1.564836000	7.827147000
1	-10.271210000	2.464516000	4.946696000
1	-8.655694000	-1.361635000	6.025676000
1	-0.121906000	0.069505000	3.599769000
1	-2.808368000	-0.261755000	6.934111000
17	-12.452388000	0.203436000	8.678077000
17	1.615431000	1.569918000	7.896807000
1	-5.305902000	0.231092000	5.515809000

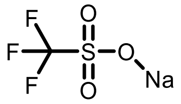

OTfNa
E (6-311+G**): -1124.028786 A.U.
G_{corr} (6-31G*): -0.00626 A.U.

6	0.782047000	-0.027207000	-0.076452000
9	1.301031000	-1.148549000	0.420716000
9	1.552803000	1.005584000	0.259648000
9	0.786434000	-0.129718000	-1.441439000
16	-0.985855000	0.220098000	0.462027000
8	-1.363191000	1.442666000	-0.329466000
8	-1.647419000	-0.985791000	-0.147146000
8	-0.986225000	0.329261000	1.915132000
11	-2.027136000	0.154361000	-2.042358000

NaI
E (6-311+G**/LANL2DZ): -173.7755144 A.U.
G_{corr} (6-31G*/LANL2DZ): -0.023923 A.U.

11	-0.911460000	0.000000000	-4.445976000
53	-0.911460000	0.000000000	-7.155976000

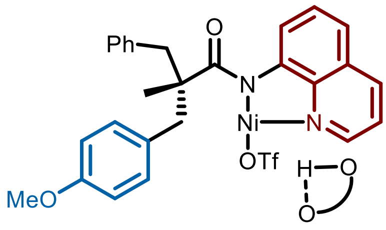

INT7
E (6-311+G**/LANL2DZ): -4737.713849 A.U.
G_{corr} (6-31G*/LANL2DZ): 0.804599 A.U.

1	-2.184103000	-2.845493000	-1.782198000
1	-4.038090000	-1.186693000	-2.081888000
1	-3.781343000	1.091843000	-1.111116000
28	-1.688666000	2.363665000	0.650429000

6	0.107589000	-2.369729000	-0.347115000
6	1.141976000	-1.932118000	0.450024000
1	1.970409000	-2.600184000	0.671868000
6	1.168580000	-0.636705000	1.016566000
1	1.989450000	-0.351404000	1.653241000
6	0.018573000	4.524033000	1.535064000
6	0.961388000	2.143355000	2.032874000
8	1.779446000	1.451872000	2.647327000
7	0.010927000	1.612871000	1.183220000
6	0.133788000	0.270860000	0.772293000
6	-0.936144000	-0.185856000	-0.050354000
6	-0.970599000	-1.490642000	-0.611317000
6	-2.114257000	-1.844469000	-1.367590000
6	-3.133157000	-0.936349000	-1.540969000
6	-3.015085000	0.346736000	-0.977626000
7	-1.951106000	0.702065000	-0.266665000
1	0.089022000	-3.375957000	-0.756130000
6	1.135581000	3.667887000	2.144735000
6	1.369334000	4.046975000	3.619256000
1	2.173846000	3.448477000	4.047785000
1	1.640448000	5.107156000	3.692785000
1	0.470763000	3.886618000	4.221451000
6	2.413209000	4.009238000	1.281525000
1	2.476059000	5.105161000	1.249400000
1	2.208921000	3.679004000	0.254757000
1	0.088464000	4.482013000	0.442888000
1	0.245954000	5.568229000	1.792759000
6	-1.432711000	4.268419000	1.925972000
6	-2.369759000	5.244261000	1.468801000
6	-1.900921000	3.346147000	2.902196000
6	-3.665559000	5.269313000	1.909884000
1	-2.050033000	5.962582000	0.720883000
6	-3.234112000	3.339028000	3.336415000
1	-1.225827000	2.631946000	3.360532000
6	-4.120663000	4.305471000	2.843153000
1	-4.379694000	5.983926000	1.518037000
1	-3.554034000	2.595452000	4.058401000
6	3.746219000	3.456775000	1.728359000
6	4.540788000	4.153477000	2.648269000
6	4.233152000	2.247576000	1.212849000
6	5.776959000	3.650359000	3.058173000
6	5.469266000	1.742881000	1.613183000
6	6.245804000	2.441555000	2.541224000
1	4.184036000	5.099121000	3.049513000
1	3.626417000	1.691031000	0.503549000
1	6.374111000	4.205237000	3.777665000
1	5.825344000	0.800858000	1.203439000
1	7.208965000	2.047970000	2.856345000
8	-5.410709000	4.421471000	3.188097000
6	-5.979880000	3.444965000	4.073378000
1	-5.854464000	2.437062000	3.678483000
1	-5.519085000	3.502135000	5.065822000
1	-7.041178000	3.687106000	4.133739000
6	-5.131906000	-3.323236000	0.618259000
6	-5.515981000	-2.256352000	1.402170000

6	-4.599283000	-1.609231000	2.281020000
6	-3.249820000	-2.098474000	2.305871000
6	-2.887093000	-3.204082000	1.491334000
6	-3.804239000	-3.812460000	0.663701000
1	-5.862769000	-3.804735000	-0.027561000
1	-6.542284000	-1.910222000	1.376144000
6	-4.985152000	-0.543135000	3.162734000
6	-2.313240000	-1.500051000	3.183709000
1	-1.859943000	-3.559178000	1.535479000
1	-3.515204000	-4.665531000	0.053615000
6	-2.688409000	-0.530474000	4.094673000
6	-4.057636000	-0.094429000	4.113315000
1	-1.288689000	-1.863821000	3.173615000
6	-6.360146000	0.040332000	3.119414000
6	-6.868432000	0.620596000	1.913443000
6	-7.137560000	0.078218000	4.310270000
6	-6.057009000	0.876025000	0.769117000
6	-8.240314000	1.038064000	1.849699000
6	-8.534349000	0.491220000	4.220352000
6	-6.575717000	1.451029000	-0.371750000
1	-5.000392000	0.642695000	0.827483000
6	-8.754393000	1.595716000	0.650683000
6	-9.042951000	0.915972000	3.010986000
6	-7.946377000	1.797009000	-0.446586000
1	-5.929462000	1.675153000	-1.214672000
1	-9.803235000	1.888138000	0.626022000
1	-10.094709000	1.186376000	2.933683000
1	-8.343239000	2.246906000	-1.352912000
8	-4.429666000	0.791912000	5.056340000
8	-6.623170000	-0.210359000	5.469394000
6	-9.398661000	0.476141000	5.424751000
6	-9.282029000	-0.520375000	6.413832000
6	-10.392422000	1.455642000	5.605900000
6	-10.135784000	-0.553535000	7.514174000
6	-11.255048000	1.437782000	6.701734000
6	-11.119963000	0.425145000	7.647499000
6	-1.685958000	0.047625000	5.027504000
6	-1.979631000	0.336839000	6.374259000
6	-0.382803000	0.308937000	4.572683000
6	-1.012758000	0.869352000	7.224516000
6	0.596195000	0.847132000	5.408753000
6	0.266458000	1.125800000	6.731265000
1	-1.250806000	1.088209000	8.260458000
1	1.581637000	1.062161000	5.014625000
1	-12.011849000	2.205583000	6.826616000
1	-10.038622000	-1.331565000	8.264619000
1	-10.480676000	2.263499000	4.885197000
1	-8.500289000	-1.262552000	6.317944000
1	-0.121294000	0.111970000	3.540325000
1	-2.977108000	0.153500000	6.749655000
17	-12.204025000	0.390159000	9.047418000
17	1.486577000	1.830936000	7.801667000
1	-5.390127000	0.428819000	5.378810000
8	-4.269336000	2.963676000	-2.415639000

8	-3.298419000	3.096692000	-0.136176000
6	-1.917865000	4.119622000	-2.204037000
9	-2.061006000	4.650601000	-3.420588000
9	-1.231201000	2.967139000	-2.316353000
9	-1.180156000	4.966712000	-1.455991000
16	-3.600815000	3.833934000	-1.438216000
8	-4.128028000	5.180790000	-1.216986000

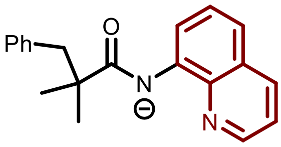

1aa$^-$

E (6-311+G**): -958.7114786 A.U.

G_{corr} (6-31G*): 0.29283 A.U.

7	4.321056000	9.108708000	-7.068201000
7	5.342147000	7.424650000	-8.954759000
8	4.195897000	10.781850000	-5.414285000
6	3.715139000	9.997772000	-6.273434000
6	5.658024000	8.854146000	-7.014040000
6	6.587813000	9.362391000	-6.079521000
6	7.943960000	8.998762000	-6.098109000
6	8.460308000	8.126556000	-7.037460000
6	7.589652000	7.583975000	-8.013539000
6	6.191262000	7.934140000	-8.020271000
6	8.044482000	6.686985000	-9.014079000
6	7.164528000	6.186274000	-9.945348000
6	5.812794000	6.595457000	-9.865778000
1	5.091159000	6.215142000	-10.592652000
1	7.487109000	5.496934000	-10.723038000
1	9.097425000	6.406573000	-9.028194000
1	9.513873000	7.852160000	-7.045731000
1	8.605908000	9.425552000	-5.344051000
1	6.217486000	10.057826000	-5.339307000
6	2.179465000	10.114880000	-6.564073000
6	2.045889000	11.208277000	-7.646400000
1	2.464116000	12.156621000	-7.287708000
1	2.597430000	10.908259000	-8.543925000
1	0.993504000	11.373420000	-7.921475000
6	1.426338000	10.603651000	-5.294396000
1	1.971001000	11.475346000	-4.918394000
1	0.422403000	10.935501000	-5.596123000
6	1.579327000	8.803510000	-7.090456000
1	0.508056000	8.931351000	-7.312444000
1	2.108364000	8.484545000	-7.990187000
1	1.679103000	8.001856000	-6.351879000
6	1.283687000	9.585868000	-4.182911000
6	0.053167000	8.961878000	-3.933739000
6	2.377972000	9.233498000	-3.374528000
6	-0.091433000	8.018096000	-2.914318000
6	2.236937000	8.290309000	-2.356487000
6	1.003214000	7.677931000	-2.118375000
1	-0.803721000	9.218916000	-4.554054000
1	3.336687000	9.701995000	-3.574244000
1	-1.058607000	7.548419000	-2.744343000
1	3.099028000	8.029519000	-1.745821000
1	0.897663000	6.942255000	-1.323546000

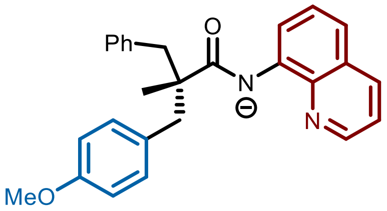

3aa⁻

E (6-311+G**): -1304.394301 A.U.

G_{corr} (6-31G*): 0.399701 A.U.

7	4.639792000	10.034795000	12.758157000
7	4.330304000	7.781181000	14.243672000
8	5.145152000	5.874506000	15.359908000
8	7.891764000	11.840721000	14.258417000
6	5.054189000	7.667237000	13.094857000
6	5.682085000	6.508189000	12.597685000
6	6.416595000	6.513329000	11.399017000
6	6.583555000	7.659385000	10.645937000
6	5.993230000	8.866410000	11.095356000
6	6.150608000	10.091760000	10.398292000
6	5.570955000	11.240924000	10.883885000
6	4.815906000	11.148912000	12.076955000
6	5.213336000	8.886583000	12.305793000
6	4.463962000	6.932301000	15.261989000
6	3.805798000	7.461817000	16.574568000
6	2.726058000	8.518777000	16.318646000
6	3.245406000	6.277365000	17.410591000
6	2.026879000	5.599715000	16.824401000
6	0.748384000	5.819418000	17.354586000
6	-0.379253000	5.200218000	16.810569000
6	-0.245748000	4.343696000	15.716832000
6	1.023271000	4.117322000	15.176297000
6	2.147523000	4.736038000	15.722607000
6	4.983327000	8.082710000	17.408337000
6	5.796878000	9.120760000	16.667966000
6	6.830796000	8.725239000	15.801588000
6	7.513637000	9.648325000	15.023286000
6	7.177481000	11.005245000	15.089134000
6	6.176335000	11.428300000	15.965220000
6	5.501678000	10.481667000	16.745144000
6	7.366800000	13.131374000	14.028562000
1	5.601774000	5.604472000	13.186052000
1	6.874593000	5.582172000	11.065001000
1	7.166737000	7.657900000	9.726639000
1	6.740805000	10.103814000	9.482514000
1	5.680976000	12.195463000	10.373394000
1	4.333092000	12.042543000	12.480180000
1	3.140451000	9.357964000	15.758730000
1	2.304372000	8.876173000	17.271758000
1	1.911633000	8.100937000	15.719634000
1	4.053522000	5.544489000	17.501909000
1	3.002496000	6.641749000	18.419320000
1	0.637523000	6.490546000	18.204362000
1	-1.361125000	5.388984000	17.240676000
1	-1.120846000	3.859733000	15.287997000
1	1.138772000	3.455712000	14.320260000
1	3.134348000	4.574967000	15.299013000
1	4.563552000	8.520144000	18.326461000
1	5.639582000	7.257243000	17.709185000
1	7.043443000	7.665591000	15.687893000
1	8.277120000	9.335657000	14.317589000
1	5.899452000	12.474984000	16.036030000

1	4.702114000	10.815867000	17.403484000
1	6.321786000	13.084784000	13.693607000
1	7.979217000	13.573846000	13.237706000
1	7.427826000	13.771254000	14.923071000

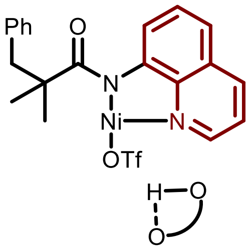

INT8

E (6-311+G**/LANL2DZ): -4392.020935 A.U.

G_{corr} (6-31G*/LANL2DZ): 0.69579 A.U.

1	-1.583902000	-3.266603000	-2.140359000
1	-3.490475000	-1.678053000	-2.464517000
1	-3.482597000	0.494098000	-1.228604000
28	-1.720140000	1.547708000	0.961428000
6	0.580490000	-2.782286000	-0.484239000
6	1.545466000	-2.327890000	0.390703000
1	2.430449000	-2.932692000	0.571380000
6	1.434388000	-1.094342000	1.076087000
1	2.211830000	-0.763531000	1.747615000
6	-1.307298000	2.944972000	2.885797000
6	0.768312000	1.557583000	2.405888000
8	1.886075000	1.154215000	2.724229000
7	-0.042209000	0.910575000	1.498011000
6	0.304736000	-0.297343000	0.894009000
6	-0.676890000	-0.751782000	-0.029409000
6	-0.564257000	-1.980955000	-0.727246000
6	-1.617975000	-2.317595000	-1.612093000
6	-2.668709000	-1.446574000	-1.797057000
6	-2.702025000	-0.234608000	-1.082637000
7	-1.747821000	0.079838000	-0.215655000
1	0.682631000	-3.735784000	-0.994568000
6	0.220755000	2.880730000	2.967113000
6	0.654459000	3.013013000	4.433646000
1	1.734336000	2.882926000	4.525444000
1	0.376784000	4.001079000	4.820746000
1	0.164028000	2.251965000	5.047324000
6	0.793413000	4.047665000	2.089118000
1	0.354630000	4.977042000	2.475378000
1	0.408211000	3.923022000	1.070474000
1	-1.663842000	3.146640000	1.845140000
1	-1.696078000	3.855255000	3.357474000
6	2.297286000	4.176717000	2.044818000
6	2.981153000	4.976929000	2.969163000
6	3.043103000	3.496886000	1.072292000
6	4.372067000	5.089381000	2.931429000
6	4.432150000	3.607999000	1.027276000
6	5.102775000	4.404557000	1.958673000
1	2.415490000	5.514480000	3.726584000
1	2.526309000	2.867244000	0.352166000
1	4.883455000	5.714182000	3.659720000
1	4.991746000	3.069971000	0.266092000
1	6.185959000	4.492152000	1.925039000
6	-5.341482000	-3.408965000	0.430252000
6	-5.524578000	-2.261429000	1.172098000
6	-4.465092000	-1.675465000	1.926054000
6	-3.215181000	-2.385295000	1.944517000
6	-3.054740000	-3.563718000	1.166205000

6	-4.087890000	-4.065984000	0.408286000
1	-6.177008000	-3.821138000	-0.130949000
1	-6.498551000	-1.789529000	1.190563000
6	-4.608364000	-0.452415000	2.674739000
6	-2.180472000	-1.947386000	2.803888000
1	-2.089305000	-4.065734000	1.190291000
1	-3.953029000	-4.969934000	-0.181424000
6	-2.340099000	-0.849610000	3.626194000
6	-3.543294000	-0.064523000	3.512608000
1	-1.277985000	-2.549931000	2.868466000
6	-5.876233000	0.343937000	2.653891000
6	-6.502407000	0.744602000	1.427066000
6	-6.470588000	0.723857000	3.890985000
6	-5.880780000	0.603056000	0.156041000
6	-7.801951000	1.350919000	1.442682000
6	-7.802040000	1.317380000	3.886894000
6	-6.492616000	0.997776000	-1.012633000
1	-4.886057000	0.188519000	0.134406000
6	-8.423585000	1.727921000	0.223627000
6	-8.430531000	1.581818000	2.690323000
6	-7.790853000	1.559413000	-0.988272000
1	-5.960289000	0.907808000	-1.956538000
1	-9.413599000	2.179226000	0.269972000
1	-9.439768000	1.989948000	2.687994000
1	-8.268849000	1.874115000	-1.912358000
8	-3.621812000	1.052053000	4.250653000
8	-5.853824000	0.567819000	5.029334000
6	-8.478554000	1.648720000	5.164378000
6	-8.370510000	0.826283000	6.302948000
6	-9.287939000	2.794751000	5.267161000
6	-9.060574000	1.118618000	7.477354000
6	-9.985143000	3.103121000	6.435354000
6	-9.867161000	2.254625000	7.532549000
6	-1.312381000	-0.535876000	4.655517000
6	-1.663708000	-0.059216000	5.935106000
6	0.047988000	-0.775947000	4.406811000
6	-0.697758000	0.170418000	6.912144000
6	1.029792000	-0.536407000	5.366798000
6	0.645550000	-0.065214000	6.617494000
1	-0.984206000	0.533664000	7.893721000
1	2.077158000	-0.679483000	5.127212000
1	-10.599804000	3.995450000	6.497285000
1	-8.973533000	0.471715000	8.344515000
1	-9.356661000	3.473366000	4.421982000
1	-7.724766000	-0.041182000	6.261649000
1	0.360086000	-1.126843000	3.431798000
1	-2.703387000	0.135294000	6.160282000
17	-10.743344000	2.632955000	
9.025375000			
17	1.876833000	0.264124000	7.842708000
1	-4.608750000	0.970374000	4.715625000
8	-3.569966000	2.551581000	-2.038200000
8	-3.338921000	2.394762000	0.450781000
6	-2.135896000	4.393551000	-0.808617000
9	-2.235954000	5.280541000	-1.799587000

9	-1.010181000	3.664515000	-1.000889000
9	-1.996376000	5.053812000	0.354434000
16	-3.640318000	3.282886000	-0.764893000
8	-4.759187000	4.180075000	-0.508675000
1	-1.828211000	2.115914000	3.376334000

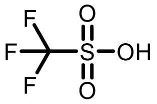

HOTf
E (6-311+G**): -962.2280856 A.U.
G_{corr} (6-31G*): 0.005347 A.U.

6	0.790198000	-0.022325000	-0.133648000
9	1.124741000	-1.236352000	0.311104000
9	1.541167000	0.898672000	0.464371000
9	0.960124000	0.036624000	-1.449988000
16	-1.001649000	0.304318000	0.293466000
8	-1.410083000	1.500483000	-0.412040000
8	-1.678927000	-0.971389000	-0.468949000
8	-1.142524000	0.119513000	1.730988000
1	-1.813031000	-1.678552000	0.191813000

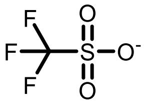

$^-$OTf
E (6-311+G**): -961.7809796 A.U.
G_{corr} (6-31G*): -0.005335 A.U.

6	0.914641000	0.000004000	-0.000008000
9	1.417863000	-1.009386000	0.746635000
9	1.417860000	1.151314000	0.500824000
9	1.417874000	-0.141921000	-1.247484000
16	-0.955058000	-0.000009000	0.000011000
8	-1.271853000	1.163438000	-0.860576000
8	-1.271815000	-1.327035000	-0.577264000
8	-1.271804000	0.163565000	1.437892000

10. NMR spectrum

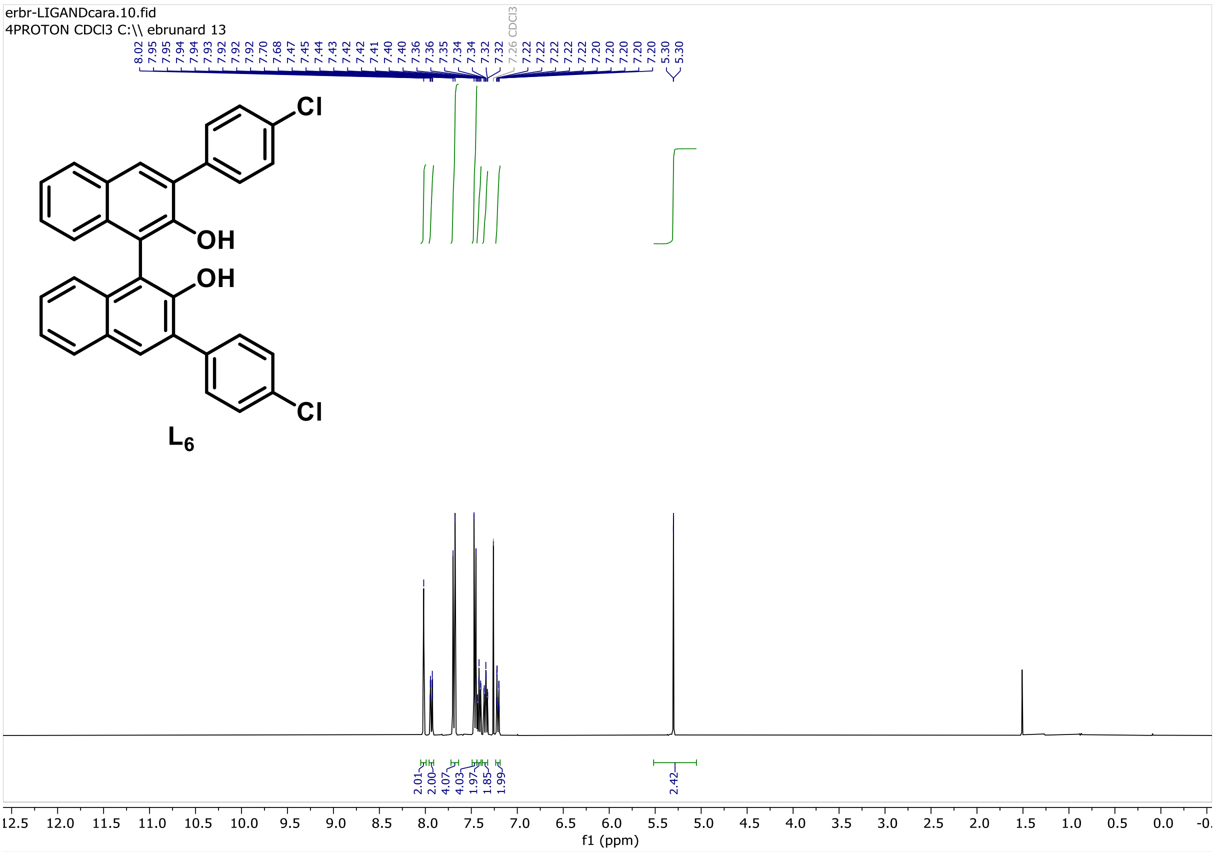

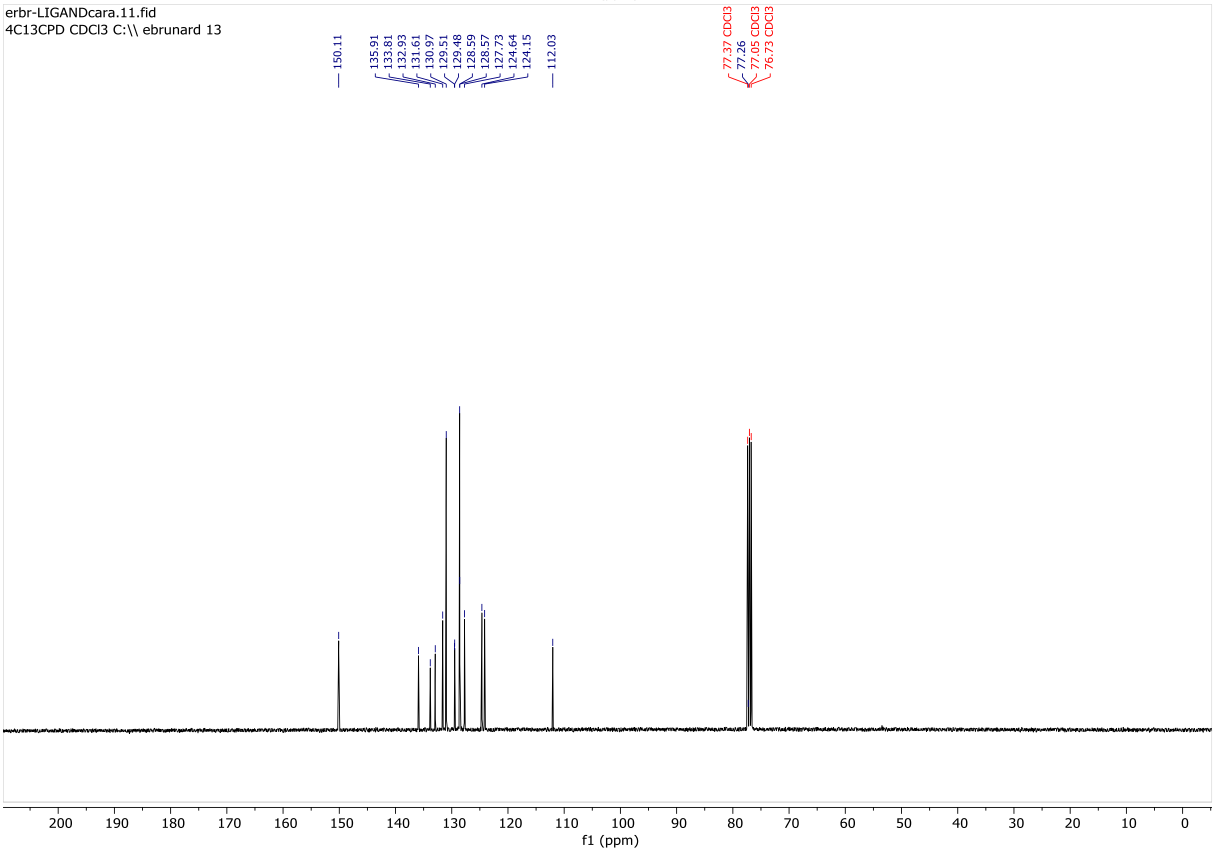

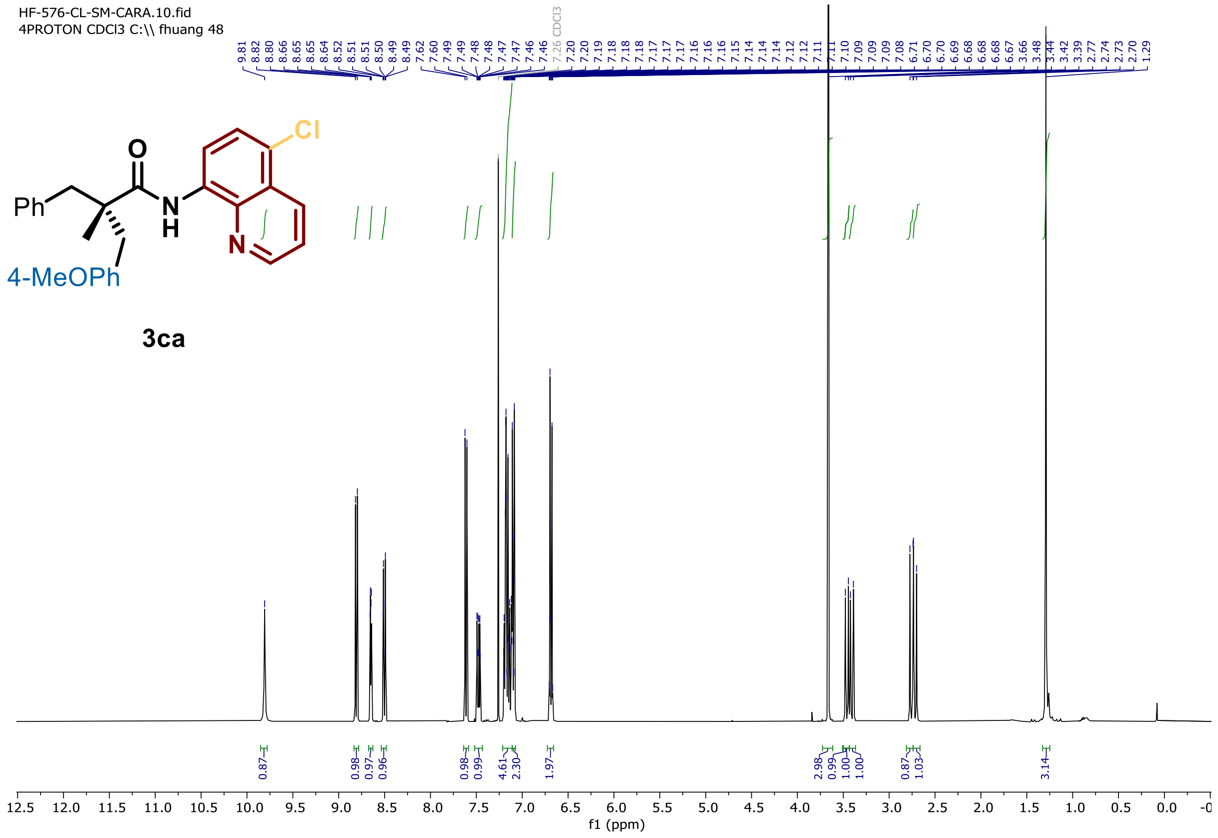
HF-576-CL-SM-CARA.10.fid
4PROTON CDCl3 C:\\ fhuang 48
3ca
f1 (ppm)

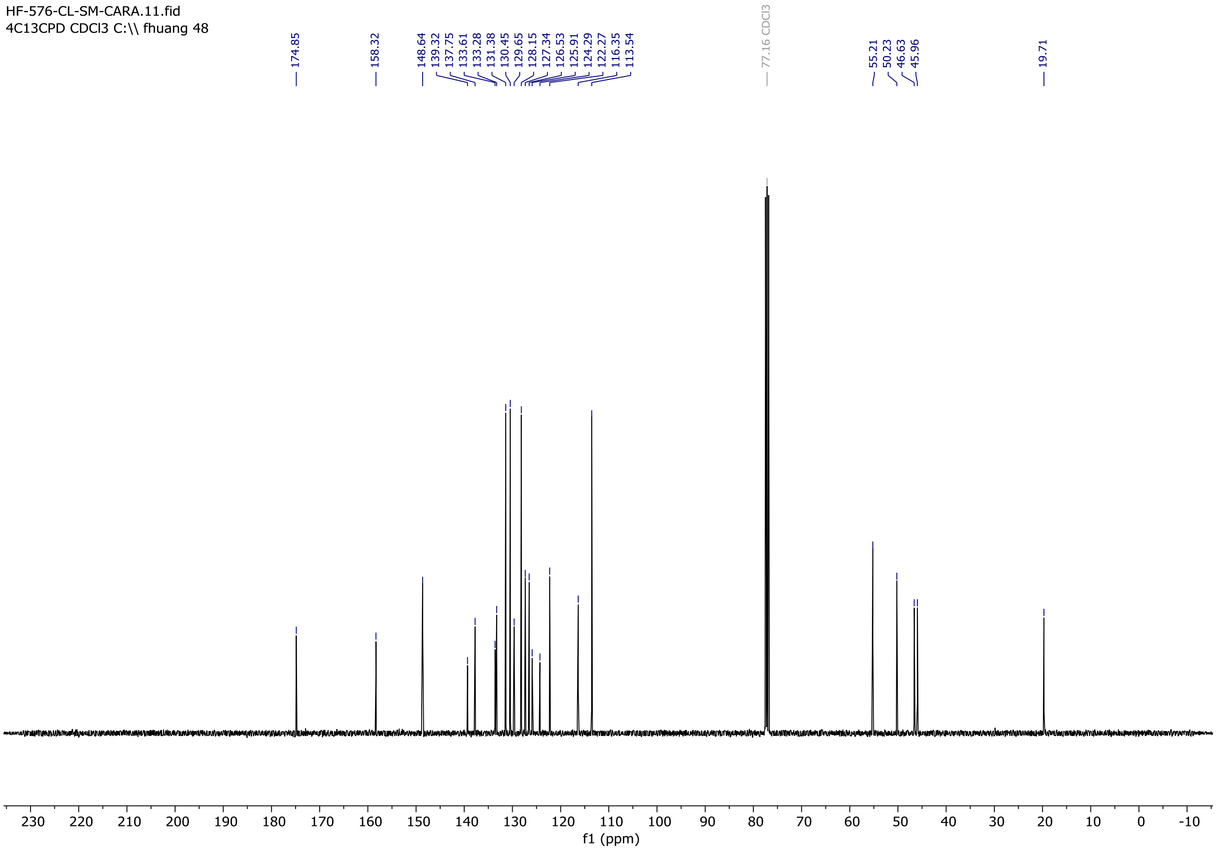
HF-576-CL-SM-CARA.11.fid
4C13CPD CDCl3 C:\\ fhuang 48
f1 (ppm)

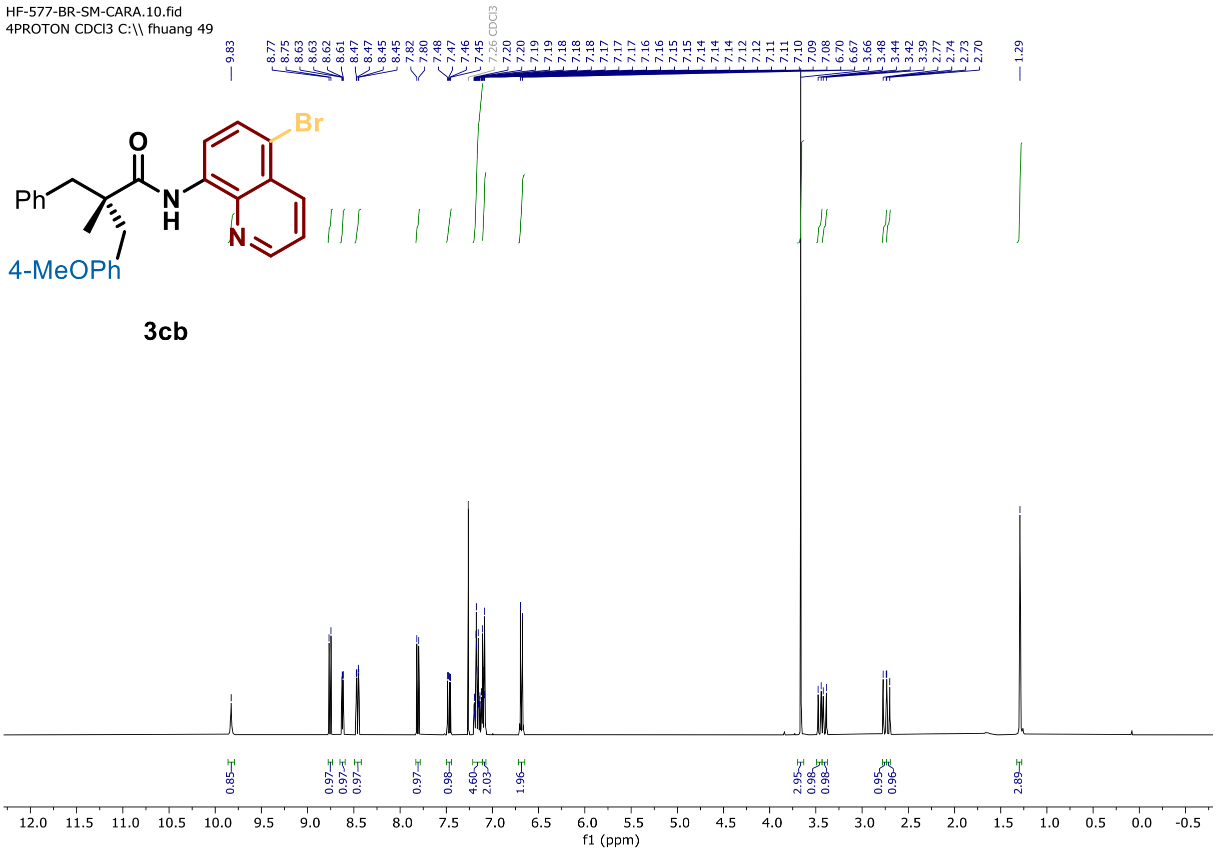
HF-577-BR-SM-CARA.10.fid
4PROTON CDCl3 C:\\ fhuang 49
9.83
8.77
8.75
8.63
8.63
8.62
8.61
8.47
8.47
8.45
8.45
7.82
7.80
7.48
7.47
7.46
7.45
7.26 CDCl3
7.20
7.20
7.19
7.19
7.18
7.18
7.18
7.17
7.17
7.16
7.16
7.15
7.15
7.14
7.14
7.12
7.12
7.11
7.11
7.10
7.09
7.08
6.70
6.67
3.66
3.48
3.44
3.42
3.39
2.77
2.74
2.73
2.70
1.29
Br
O
Ph
N
H
N
4-MeOPh
0.85
0.97
0.97
0.97
0.97
0.98
4.60
2.03
1.96
2.95
0.98
0.98
0.95
0.96
2.89
f1 (ppm)

3cb

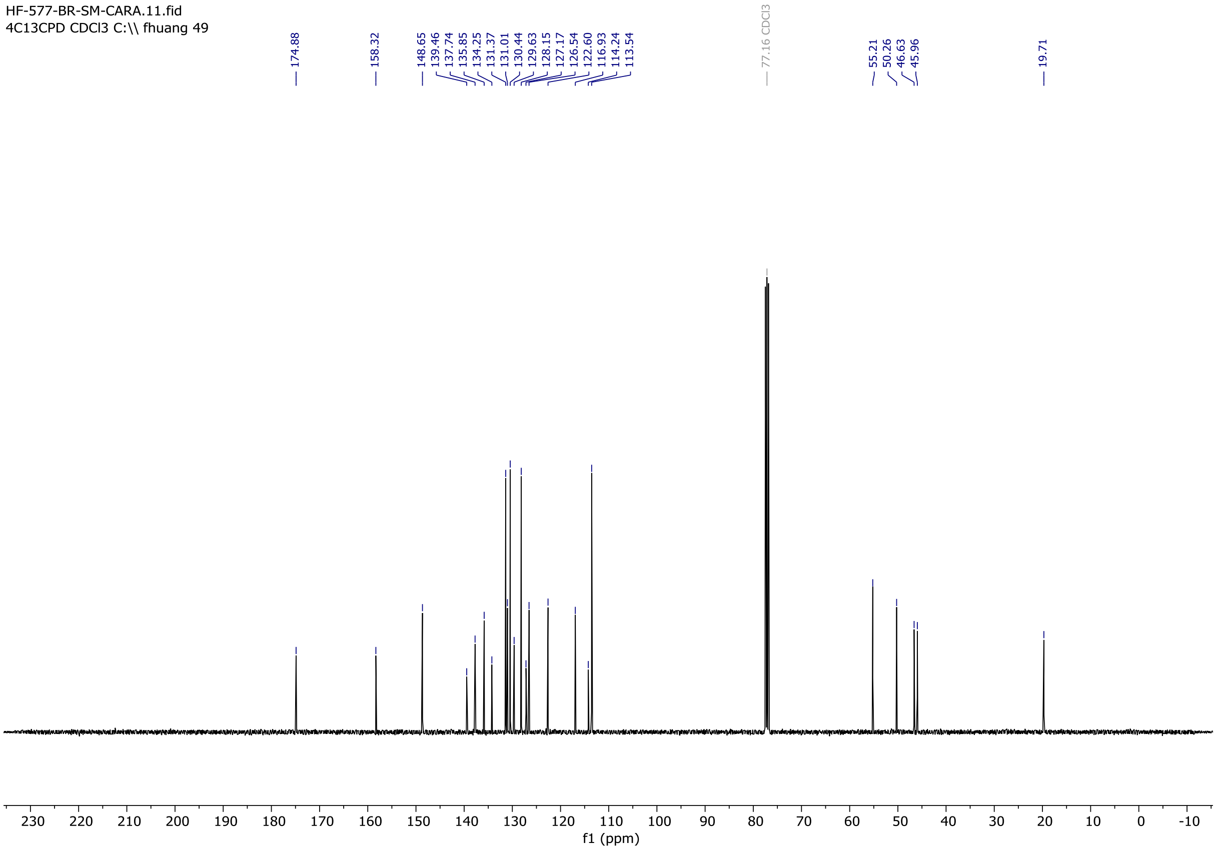
HF-577-BR-SM-CARA.11.fid
4C13CPD CDCl3 C:\\ fhuang 49
174.88
158.32
148.65
139.46
137.74
135.85
134.25
131.37
131.01
130.44
129.63
128.15
127.17
126.54
122.60
116.93
114.24
113.54
77.16 CDCl3
55.21
50.26
46.63
45.96
19.71
f1 (ppm)

HF-595-F-SM-CARA.10.fid
4PROTON CDCl3 C:\\ fhuang 38

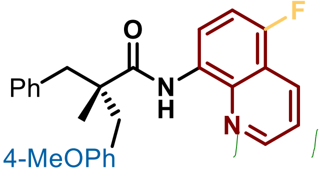

3cc

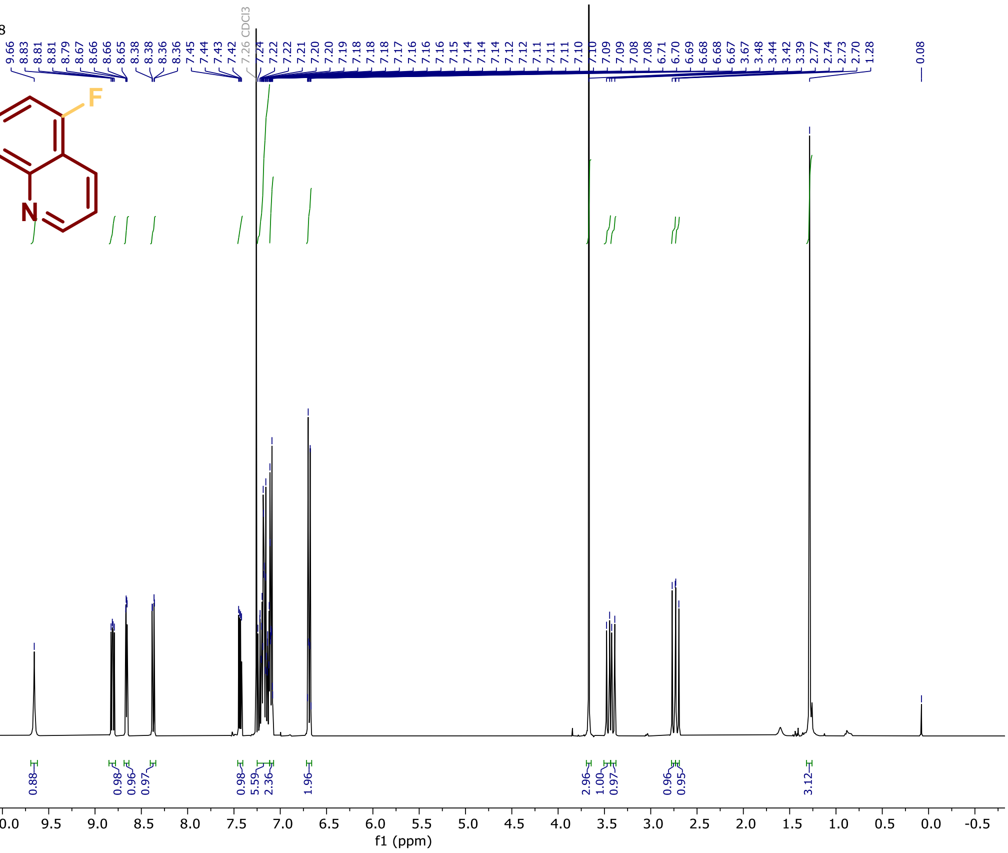

HF-595-F-SM-CARA.13.fid
4C13CPD CDCl3 C:\\ fhuang 38

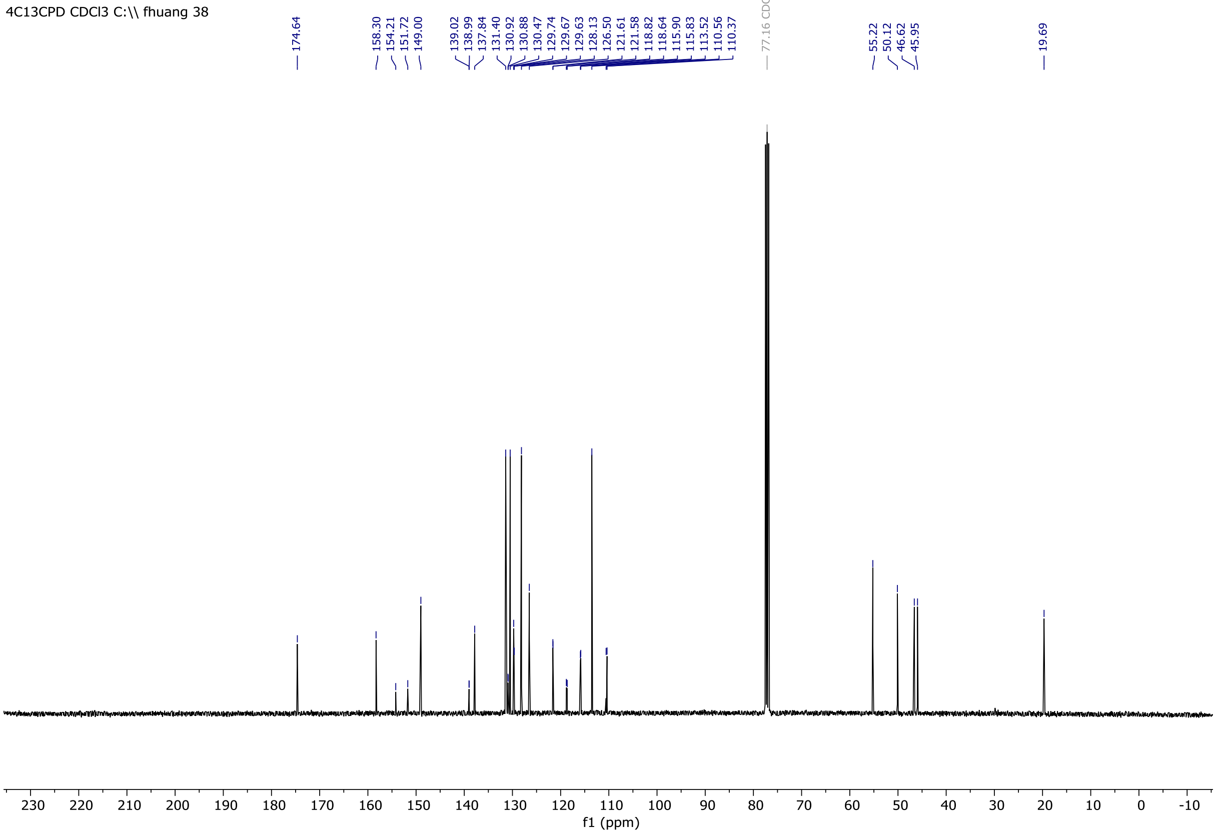

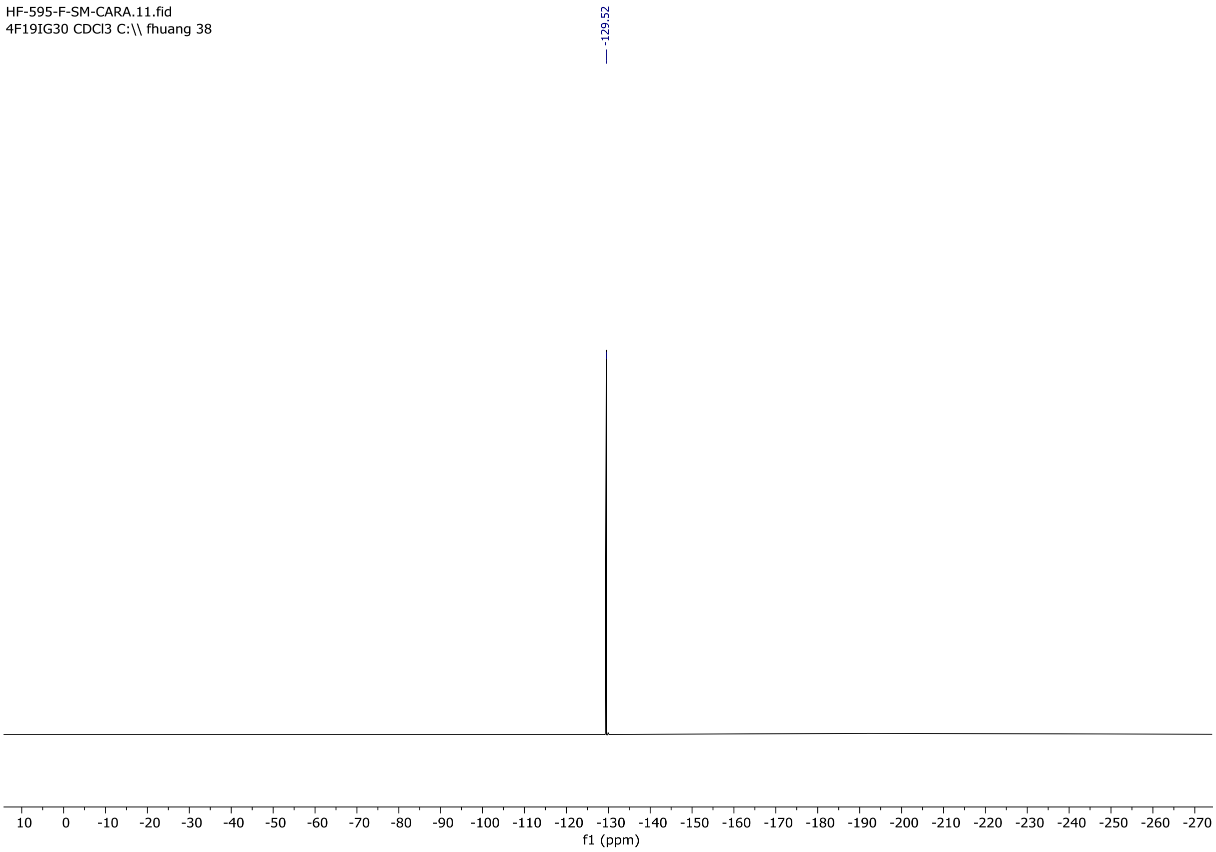
HF-595-F-SM-CARA.11.fid
4F19IG30 CDCl3 C:\\ fhuang 38
-129.52
10 0 -10 -20 -30 -40 -50 -60 -70 -80 -90 -100 -110 -120 -130 -140 -150 -160 -170 -180 -190 -200 -210 -220 -230 -240 -250 -260 -270
f1 (ppm)

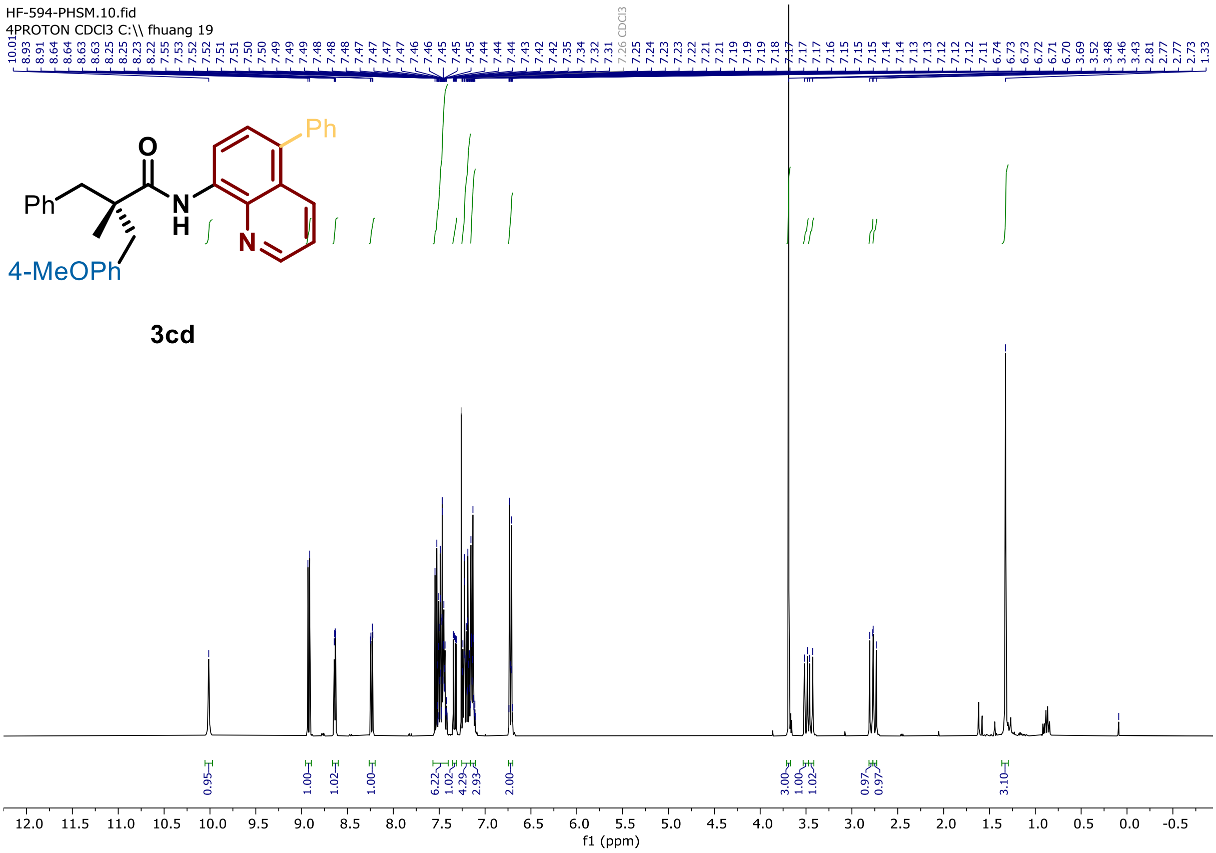
HF-594-PHSM.10.fid
4PROTON CDCl3 C:\\ fhuang 19
10.01 8.93 8.91 8.64 8.63 8.63 8.25 8.25 8.23 8.22 7.55 7.53 7.52 7.52 7.51 7.51 7.50 7.49 7.49 7.49 7.48 7.48 7.48 7.47 7.47 7.47 7.46 7.45 7.45 7.45 7.44 7.44 7.43 7.42 7.42 7.35 7.34 7.32 7.31 7.26 CDCl3 7.25 7.24 7.23 7.23 7.22 7.21 7.21 7.19 7.19 7.19 7.18 7.17 7.17 7.17 7.16 7.15 7.15 7.15 7.14 7.14 7.13 7.13 7.12 7.12 7.12 7.11 6.74 6.73 6.73 6.72 6.71 6.70 3.69 3.52 3.48 3.46 3.43 2.81 2.77 2.77 2.73 1.33
Ph
O
N
H
Ph
N
4-MeOPh
3cd
0.95 1.00 1.02 1.00 6.22 1.02 4.29 2.93 2.00 3.00 1.00 1.02 0.97 0.97 3.10
12.0 11.5 11.0 10.5 10.0 9.5 9.0 8.5 8.0 7.5 7.0 6.5 6.0 5.5 5.0 4.5 4.0 3.5 3.0 2.5 2.0 1.5 1.0 0.5 0.0 -0.5
f1 (ppm)

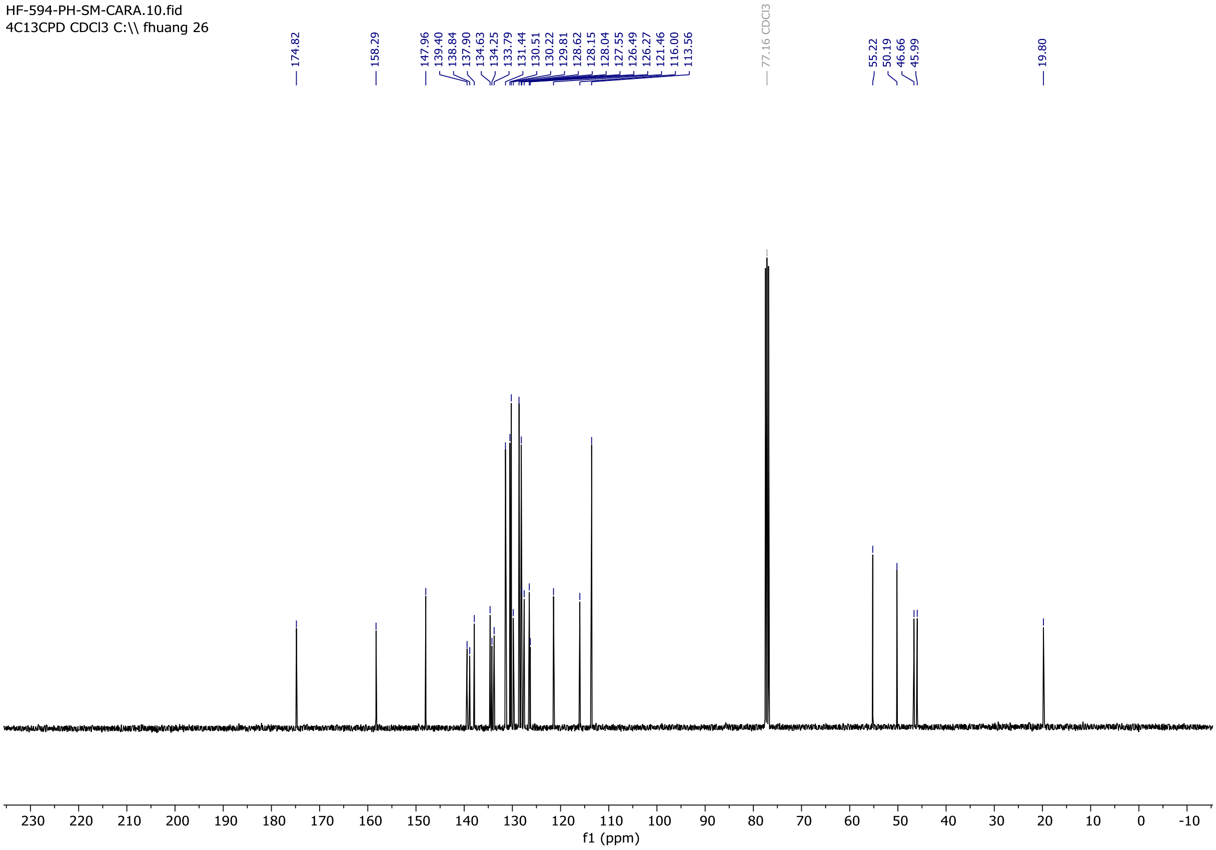
HF-594-PH-SM-CARA.10.fid
4C13CPD CDCl3 C:\\ fhuang 26
174.82 158.29 147.96 139.40 138.84 137.90 134.63 134.25 133.79 131.44 130.51 130.22 129.81 128.62 128.15 128.04 127.55 126.49 126.27 121.46 116.00 113.56 77.16 CDCl3 55.22 50.19 46.66 45.99 19.80
230 220 210 200 190 180 170 160 150 140 130 120 110 100 90 80 70 60 50 40 30 20 10 0 -10
f1 (ppm)

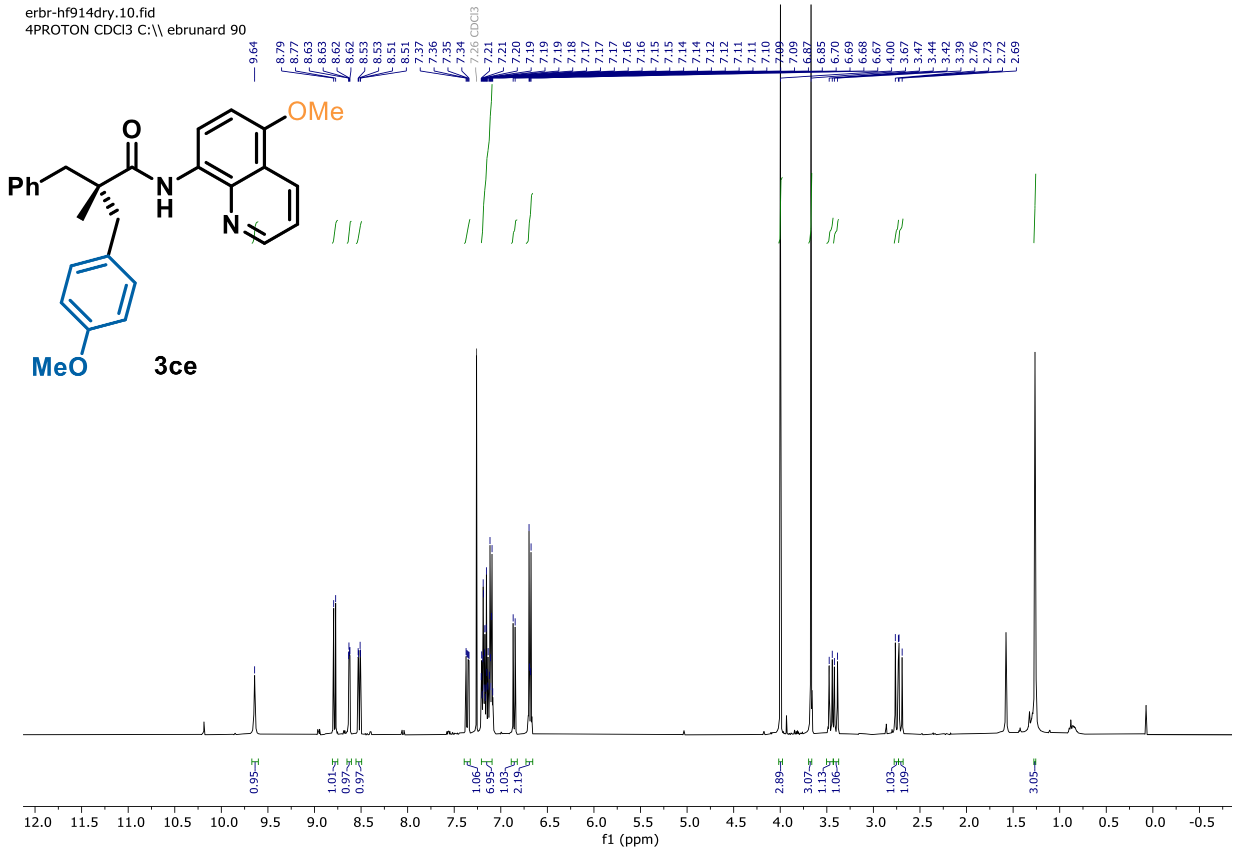
erbr-hf914dry.10.fid
4PROTON CDCl3 C:\\ ebrunard 90
OMe
O
Ph
N
H
N
MeO
3ce
f1 (ppm)

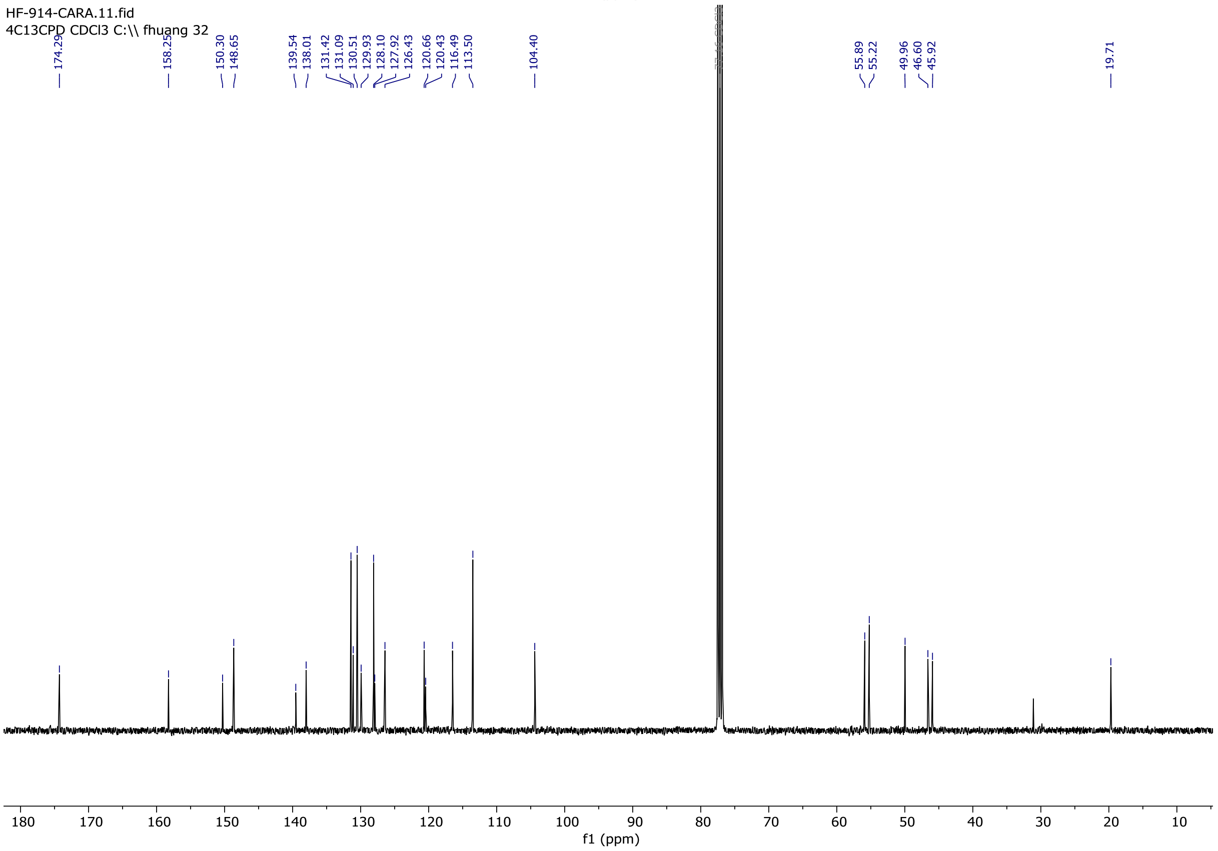
HF-914-CARA.11.fid
4C13CPD CDCl3 C:\\ fhuang 32
174.29
158.25
150.30
148.65
139.54
138.01
131.42
131.09
130.51
129.93
128.10
127.92
126.43
120.66
120.43
116.49
113.50
104.40
55.89
55.22
49.96
46.60
45.92
19.71
f1 (ppm)

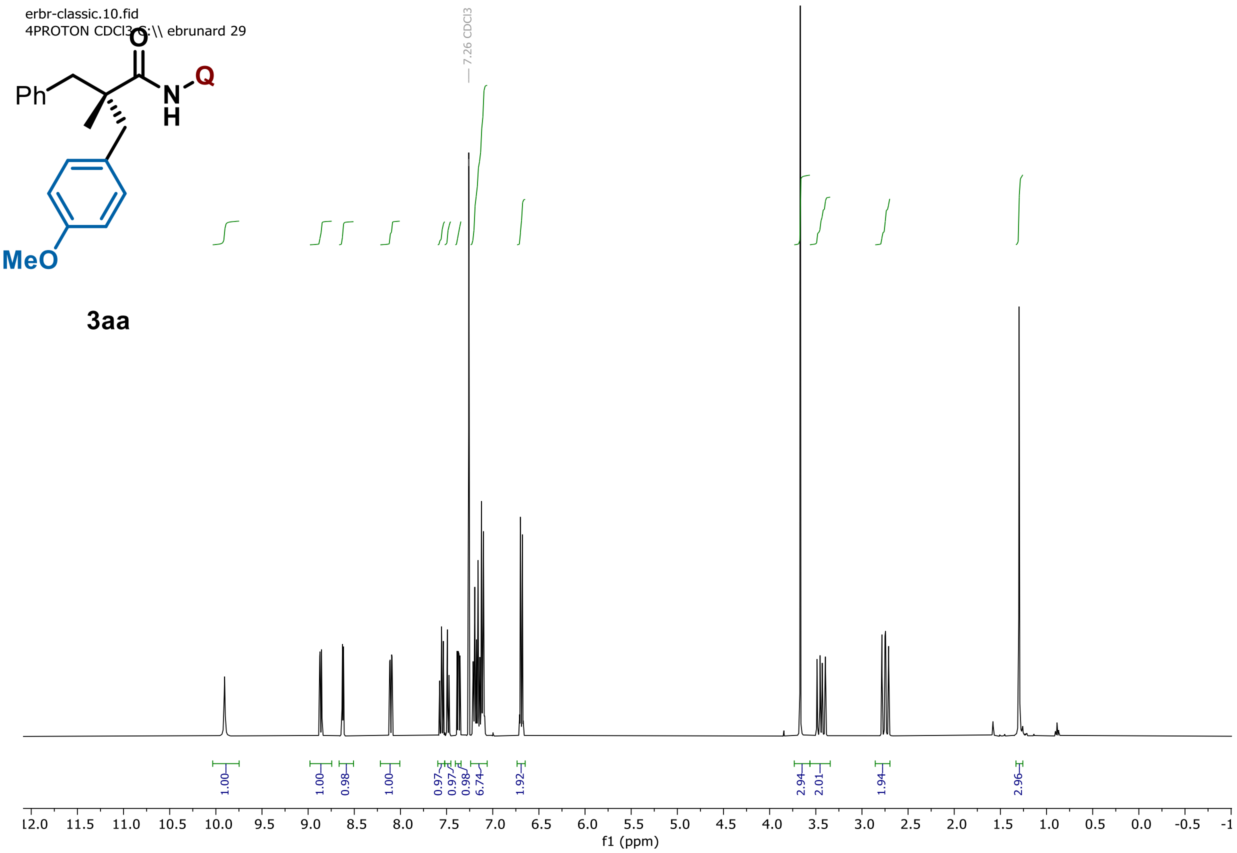
erbr-classic.10.fid
4PROTON CDCl3 C:\\ ebrunard 29
Ph
O
N
H
Q
MeO
3aa
7.26 CDCl3
1.00
1.00
0.98
1.00
0.97
0.97
0.98
6.74
1.92
2.94
2.01
1.94
2.96
f1 (ppm)

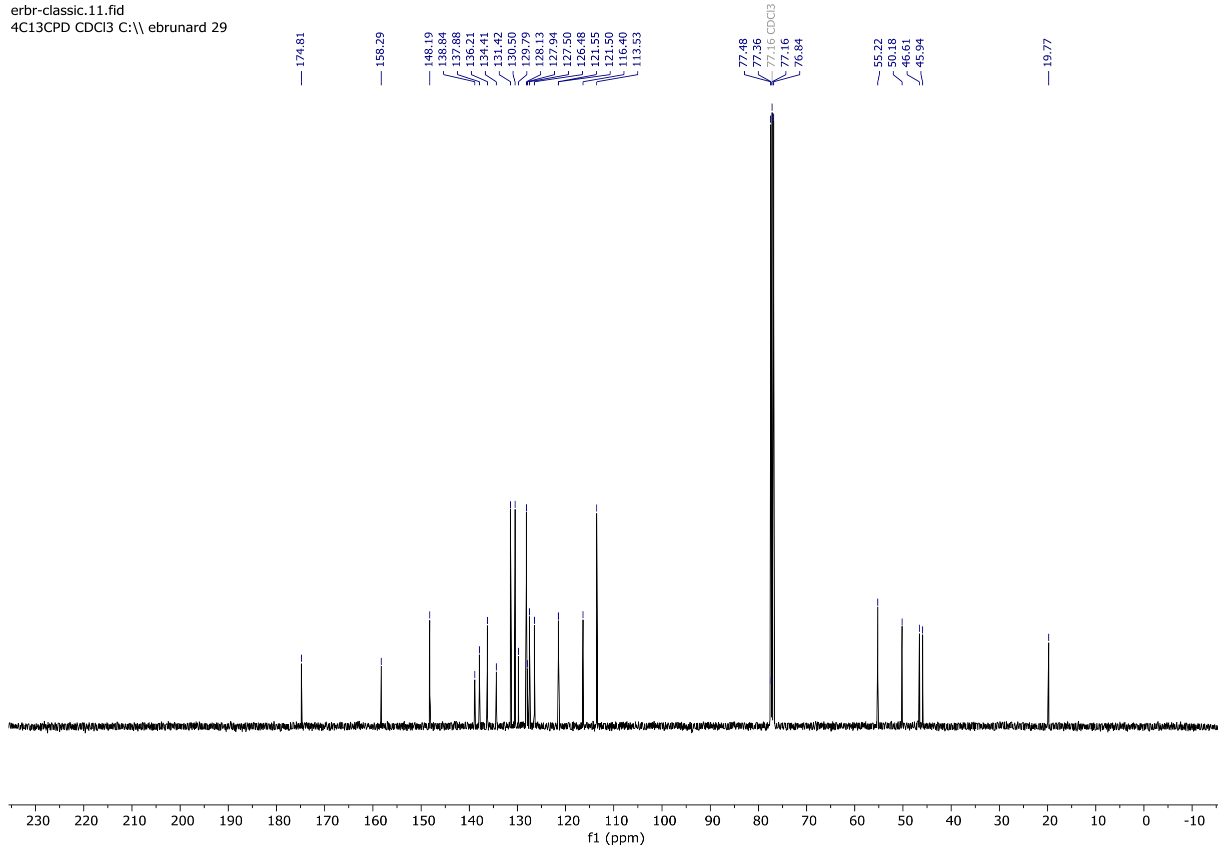
erbr-classic.11.fid
4C13CPD CDCl3 C:\\ ebrunard 29
174.81
158.29
148.19
138.84
137.88
136.21
134.41
131.42
130.50
129.79
128.13
127.94
127.50
126.48
121.55
121.50
116.40
113.53
77.48
77.36
77.16 CDCl3
77.16
76.84
55.22
50.18
46.61
45.94
19.77
f1 (ppm)

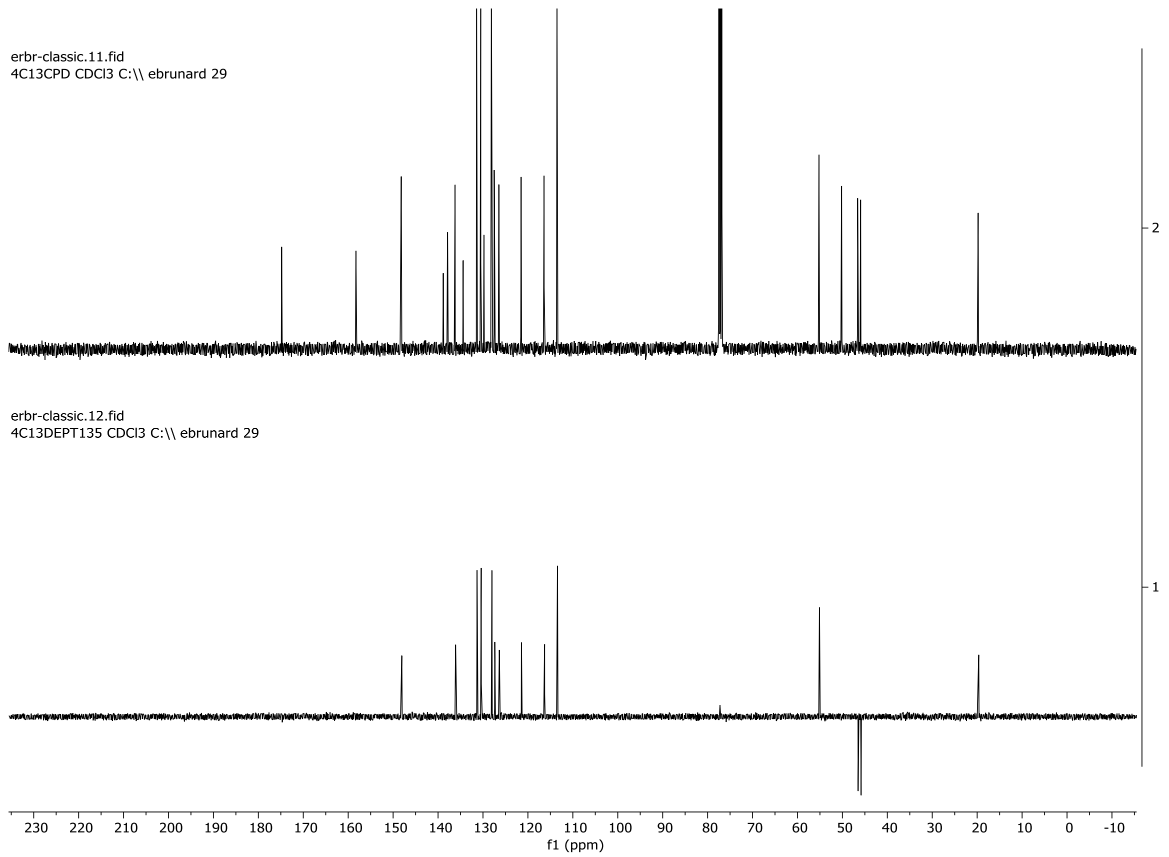
erbr-classic.11.fid
4C13CPD CDCl3 C:\\ ebrunard 29
erbr-classic.12.fid
4C13DEPT135 CDCl3 C:\\ ebrunard 29
2
1
230 220 210 200 190 180 170 160 150 140 130 120 110 100 90 80 70 60 50 40 30 20 10 0 -10
f1 (ppm)

erbr-622f.1cara.10.fid
4PROTON CDCl3 C:\\ ebrunard 9

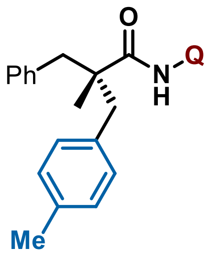

3ab

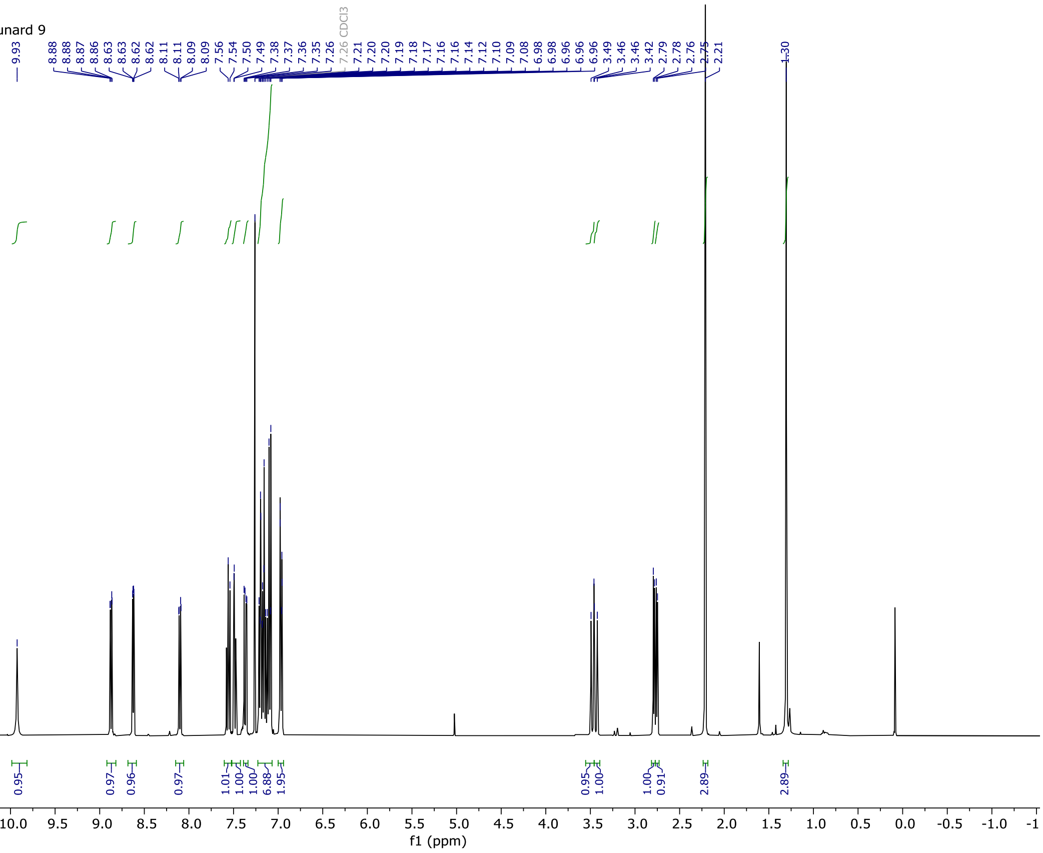

erbr-622f.1cara.11.fid
4C13CPD CDCl3 C:\\ ebrunard 9

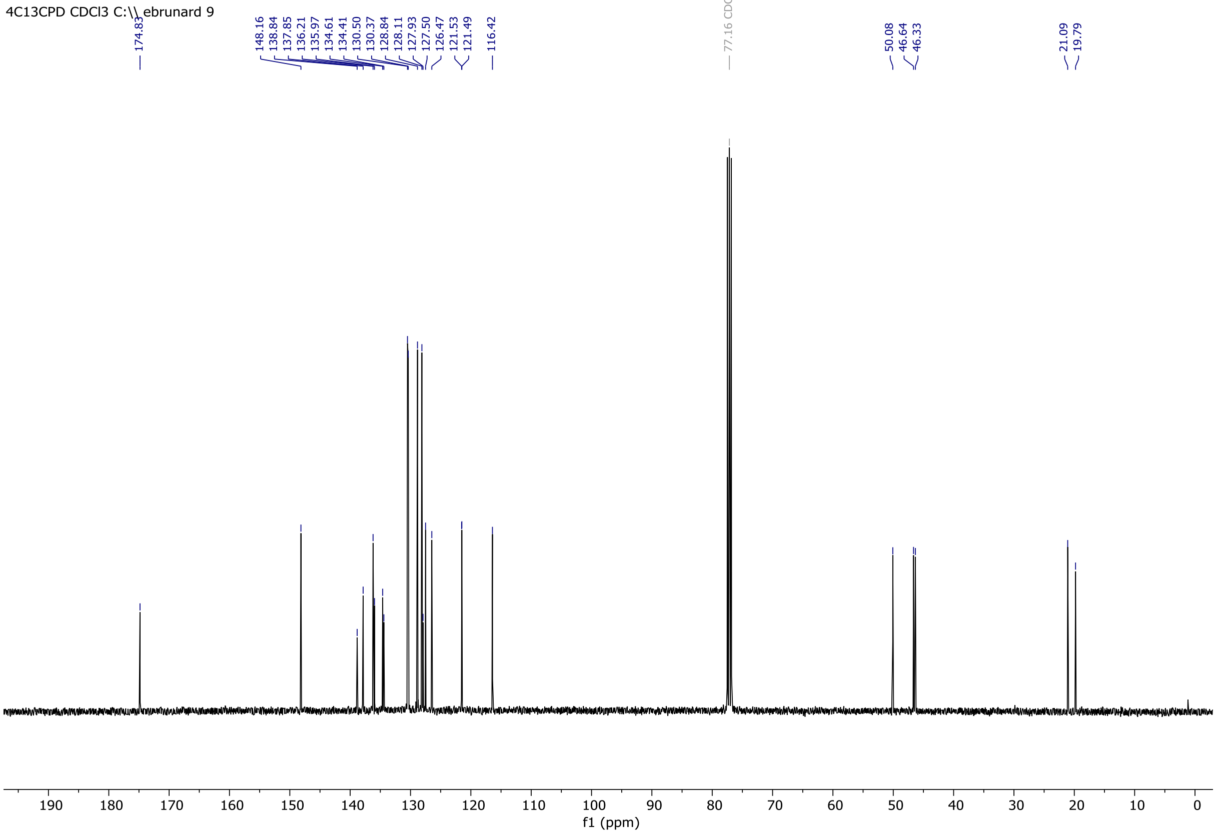

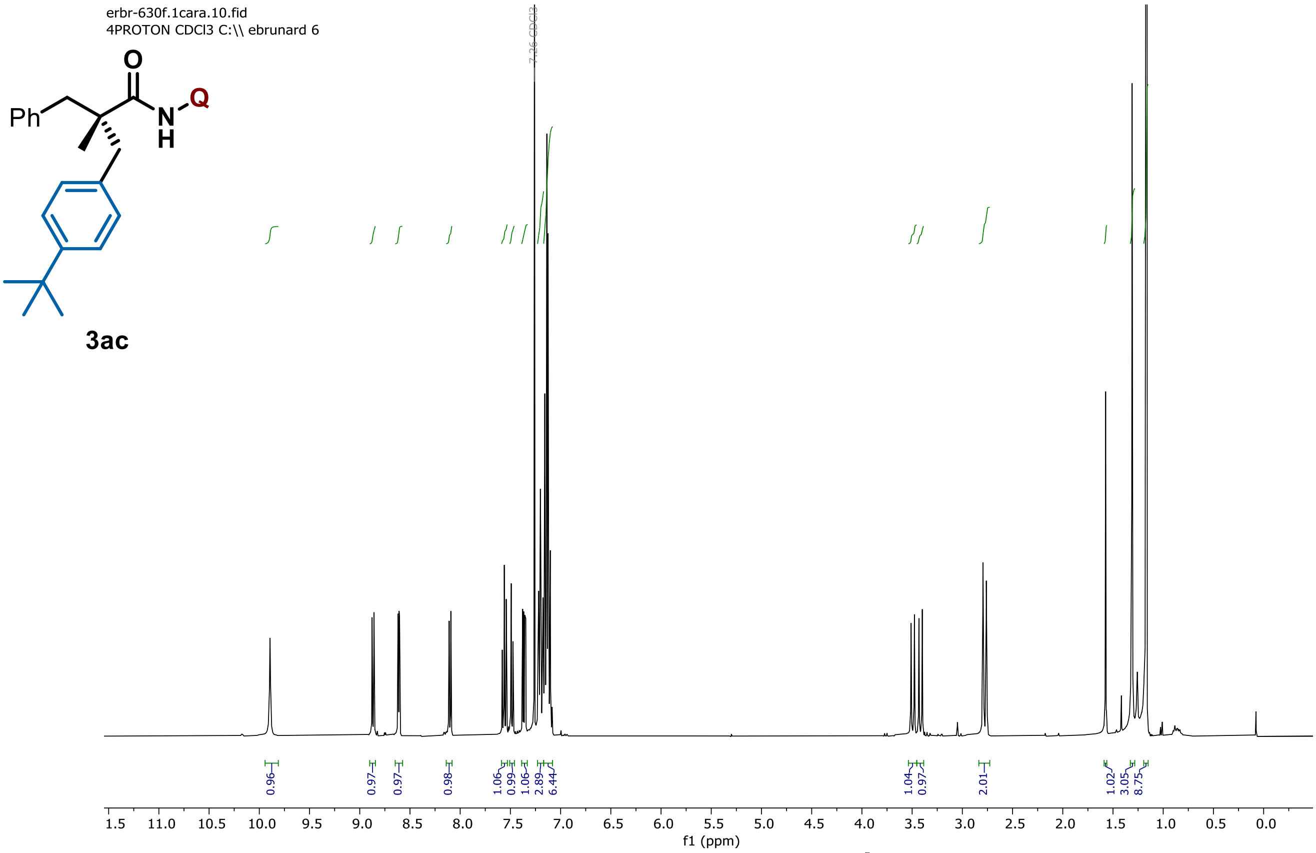

3ac
erbr-630f.1cara.10.fid
4PROTON CDCl3 C:\\ ebrunard 6
7.26 CDCl3
0.96
0.97
0.97
0.98
1.06
0.99
1.06
2.89
6.44
1.04
0.97
2.01
1.02
3.05
8.75
f1 (ppm)

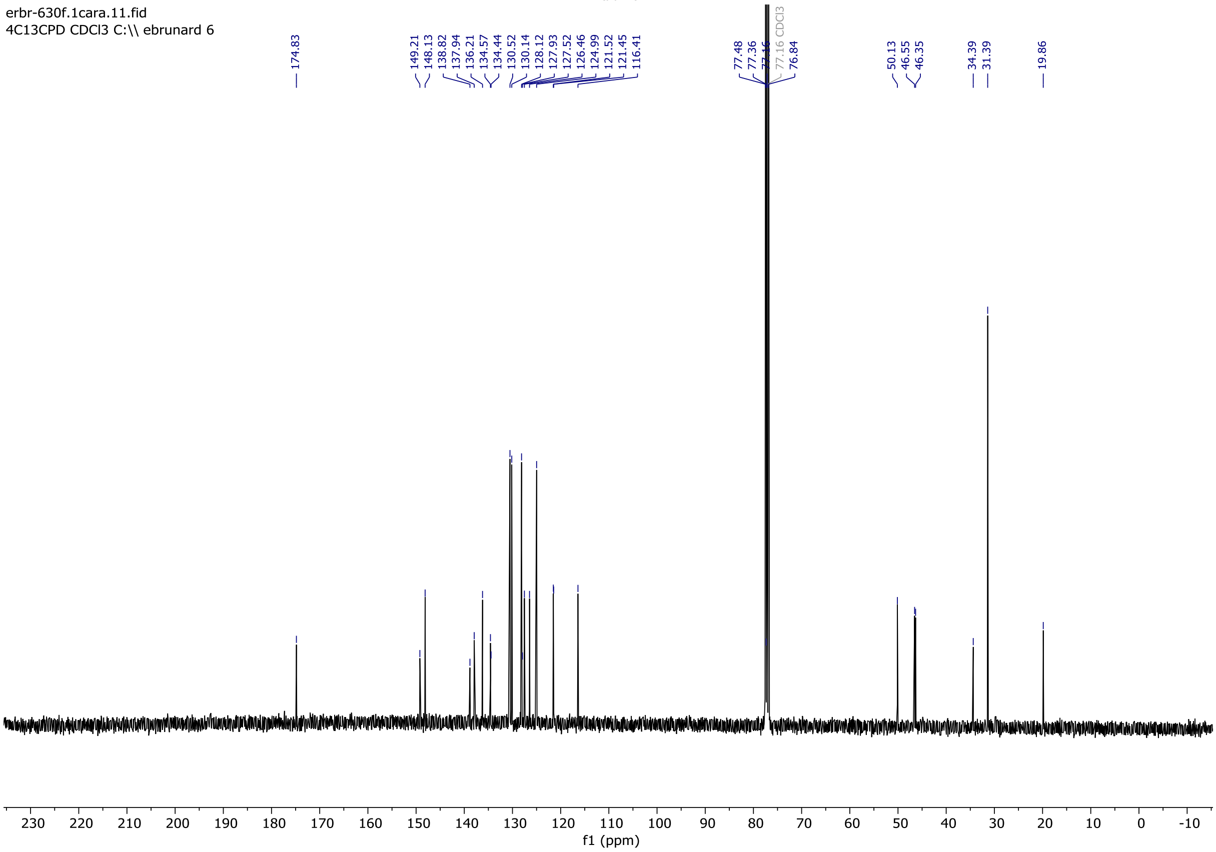
erbr-630f.1cara.11.fid
4C13CPD CDCl3 C:\\ ebrunard 6
174.83
149.21
148.13
138.82
137.94
136.21
134.57
134.44
130.52
130.14
128.12
127.93
127.52
126.46
124.99
121.52
121.45
116.41
77.48
77.36
77.16 CDCl3
76.84
50.13
46.55
46.35
34.39
31.39
19.86
f1 (ppm)

erbr-644f.2cara.10.fid
4PROTON CDCl3 C:\\ ebrunard 3

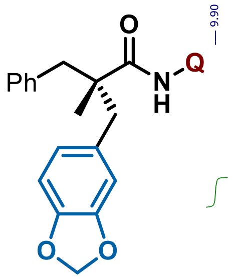

3ad

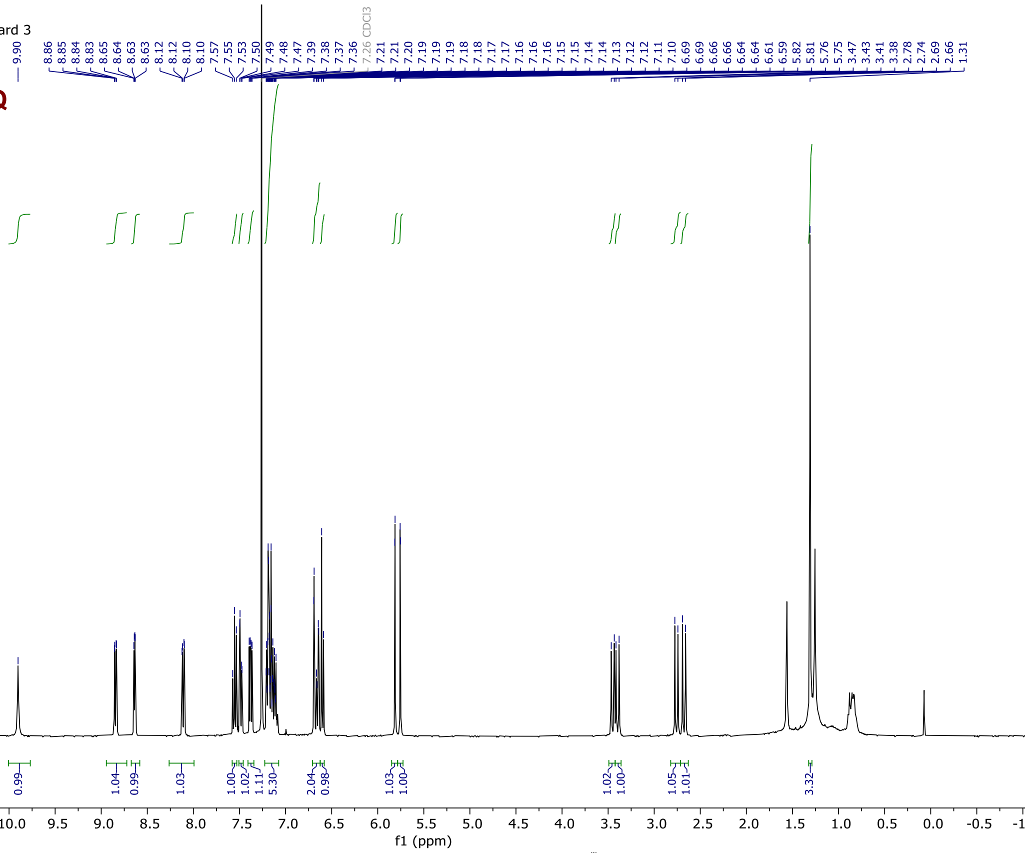

erbr-644f.2cara.11.fid
4C13CPD CDCl3 C:\\ ebrunard 3

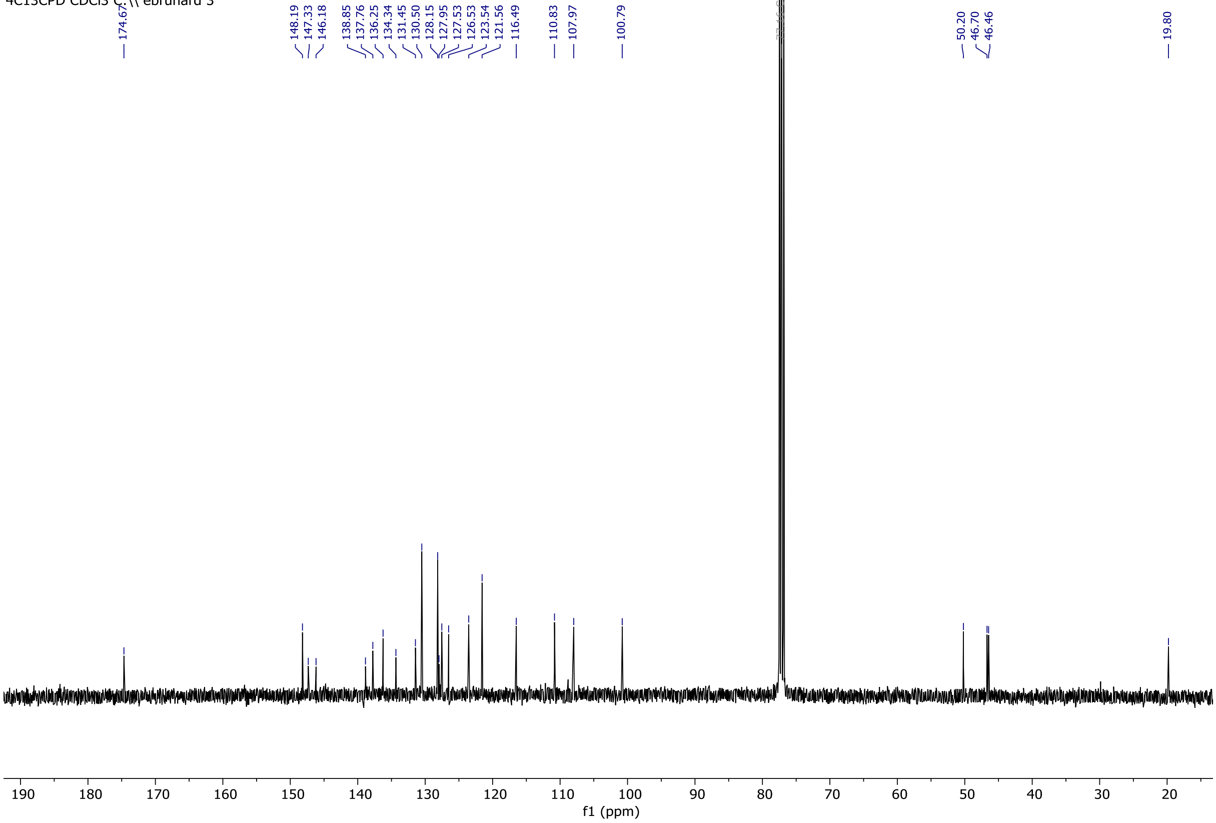

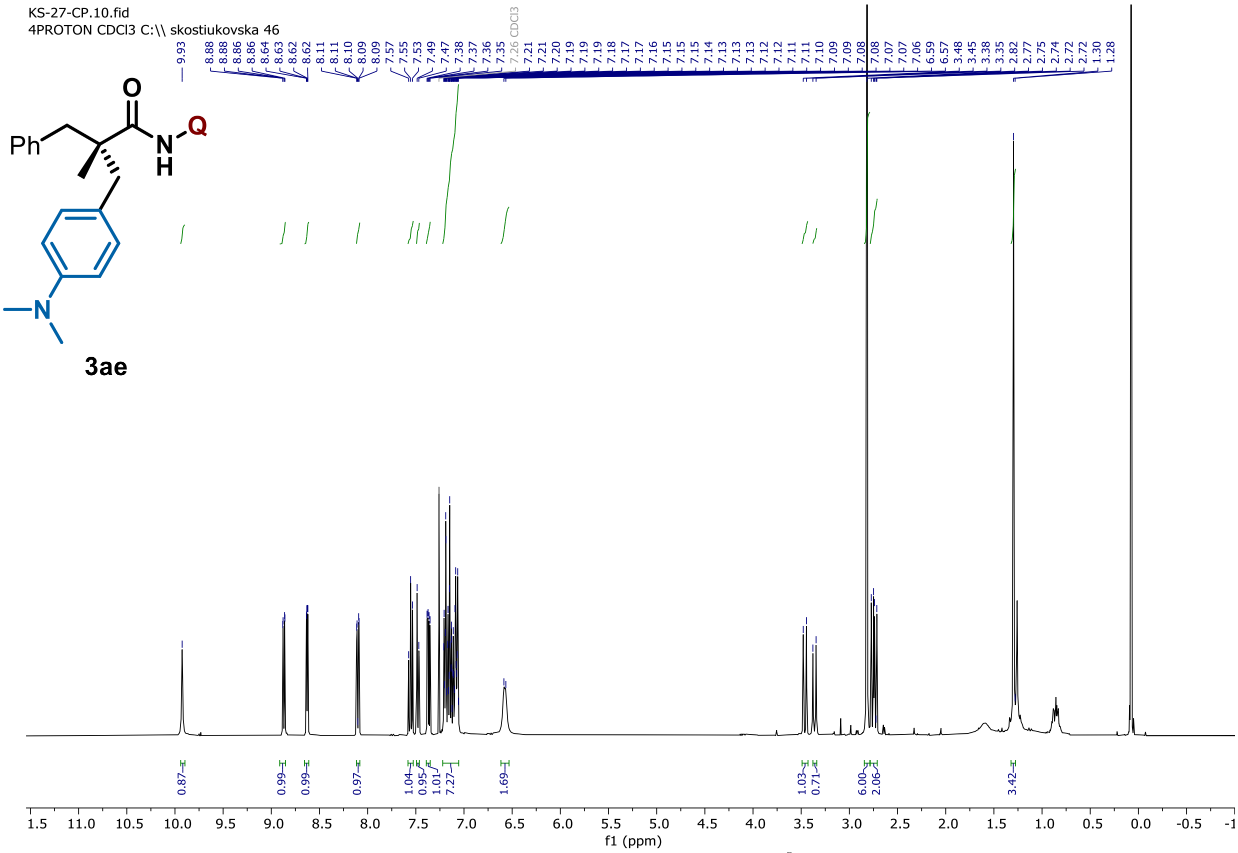

KS-27-CP.10.fid
4PROTON CDCl3 C:\\ skostiukovska 46
f1 (ppm)

3ae

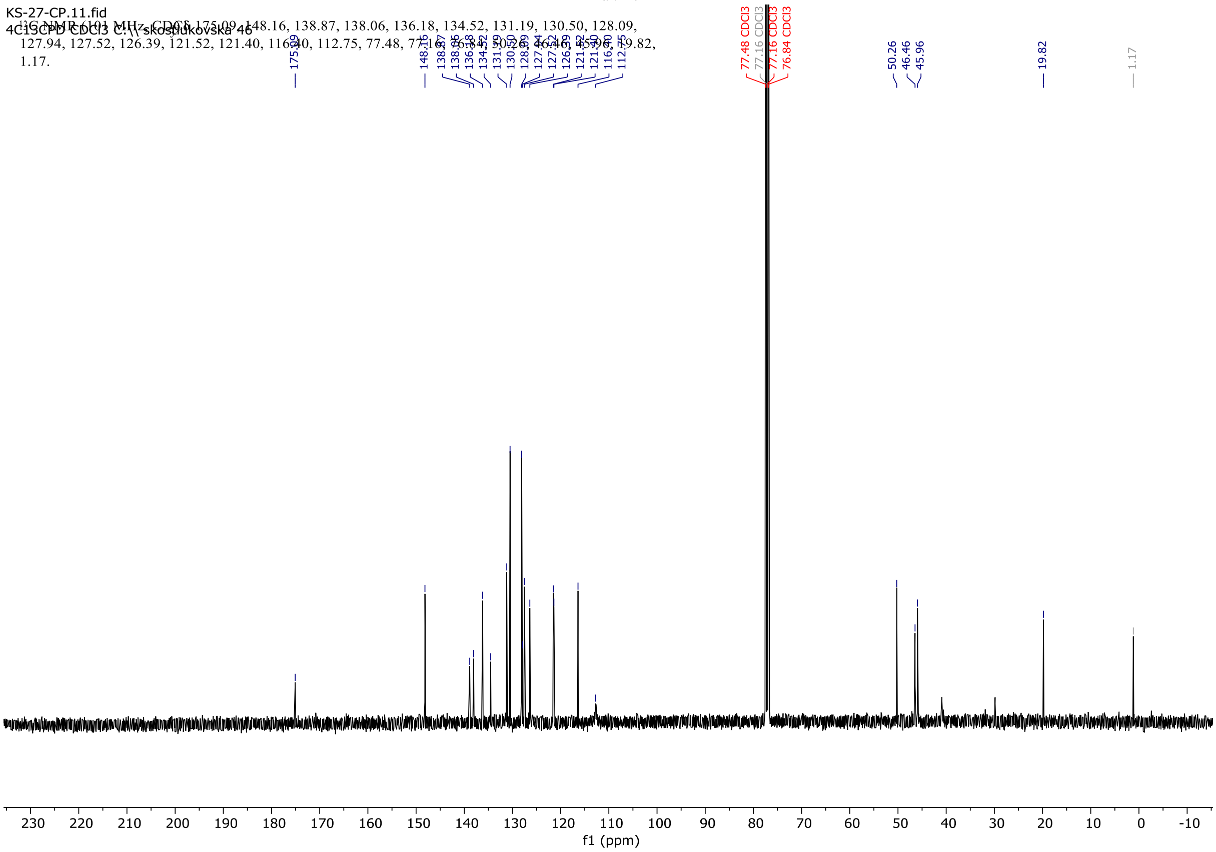

KS-27-CP.11.fid
4C13CPD CDCl3 C:\\ skostiukovska 46
[illegible] 175.09, 148.16, 138.87, 138.06, 136.18, 134.52, 131.19, 130.50, 128.09, 127.94, 127.52, 126.39, 121.52, 121.40, 116.40, 112.75, 77.48, 77.16, [illegible] 19.82, 1.17.
f1 (ppm)

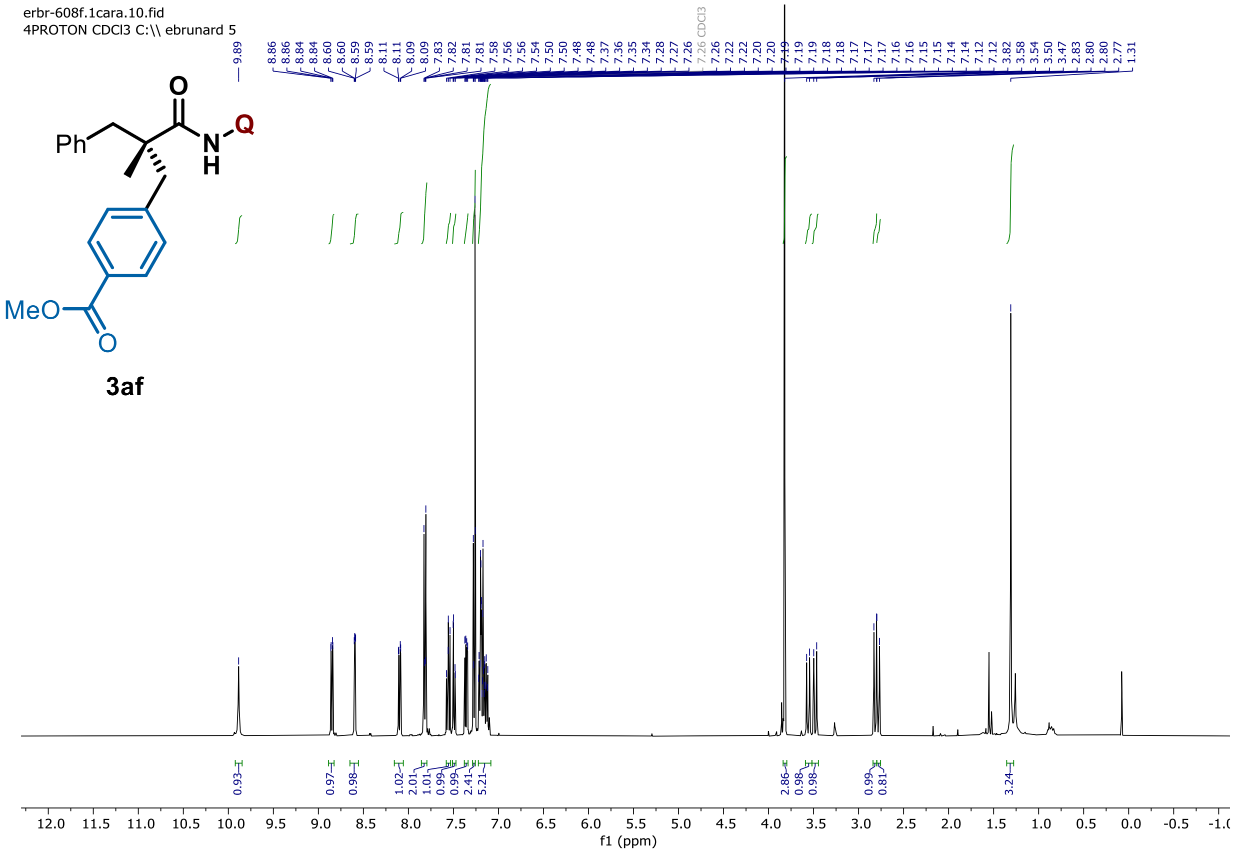
erbr-608f.1cara.10.fid
4PROTON CDCl3 C:\\ ebrunard 5
f1 (ppm)
Ph
O
N
H
Q
MeO
O

3af

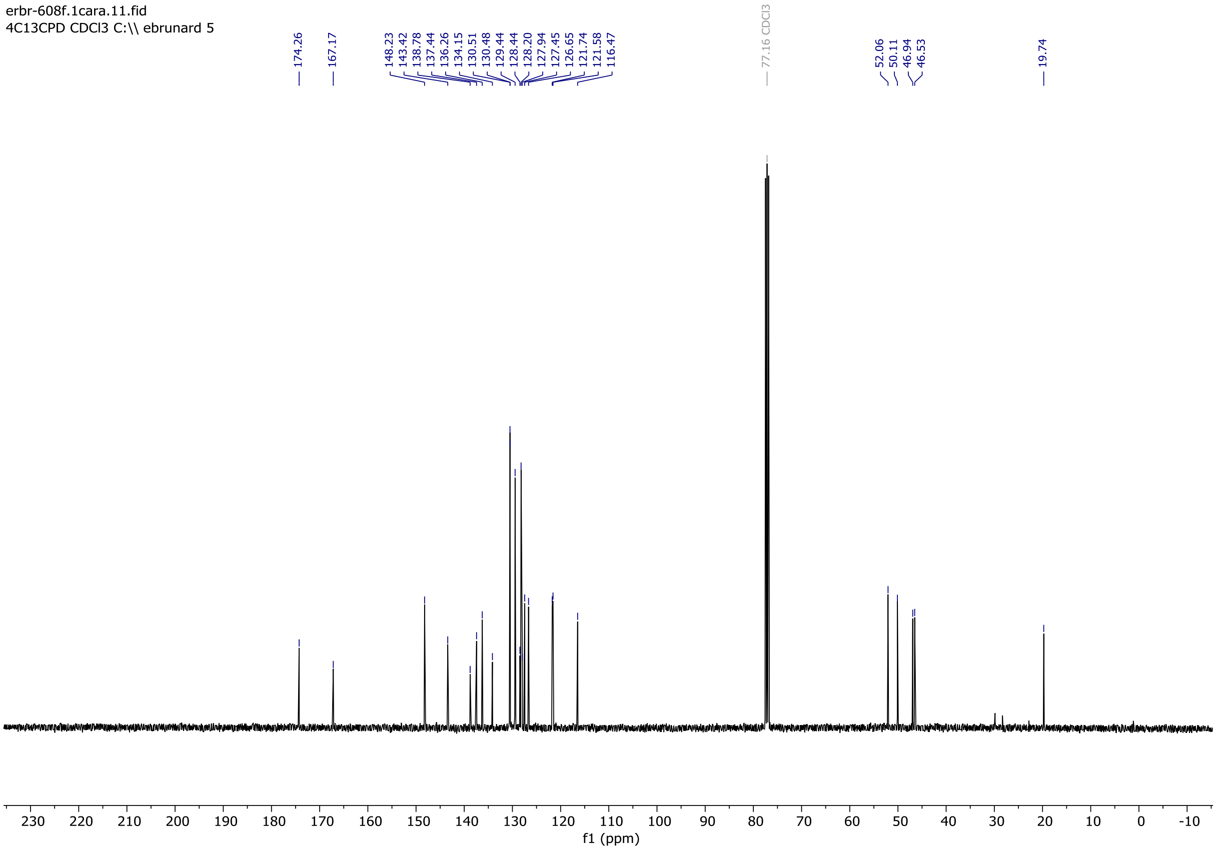
erbr-608f.1cara.11.fid
4C13CPD CDCl3 C:\\ ebrunard 5
f1 (ppm)

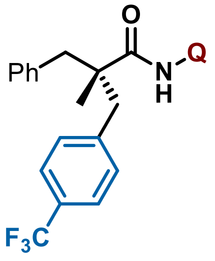
Ph
O
N
H
Q
F3C

3ag

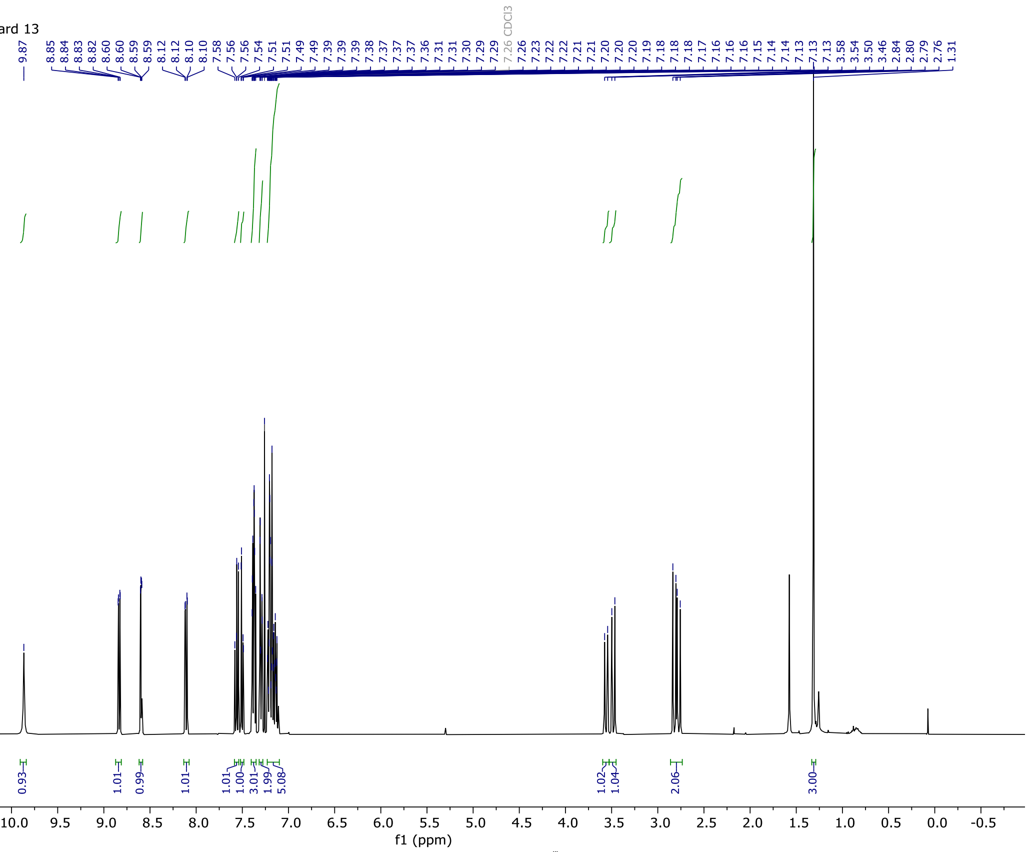
KS-37-PPPCARA.10.fid
4PROTON CDCl3 C:\\ ebrunard 13
9.87
8.85 8.84 8.83 8.82 8.60 8.59 8.59 8.12 8.12 8.10 8.10 7.58 7.56 7.56 7.54 7.51 7.51 7.49 7.39 7.39 7.39 7.38 7.37 7.37 7.37 7.36 7.31 7.31 7.30 7.29 7.29 7.26 CDCl3 7.26 7.23 7.22 7.22 7.21 7.21 7.20 7.20 7.20 7.19 7.18 7.18 7.18 7.17 7.16 7.16 7.16 7.15 7.14 7.14 7.13 7.13 7.13 3.58 3.54 3.50 3.46 2.84 2.80 2.79 2.76 1.31
0.93 1.01 0.99 1.01 1.01 1.00 3.01 1.99 5.08 1.02 1.04 2.06 3.00
12.0 11.5 11.0 10.5 10.0 9.5 9.0 8.5 8.0 7.5 7.0 6.5 6.0 5.5 5.0 4.5 4.0 3.5 3.0 2.5 2.0 1.5 1.0 0.5 0.0 -0.5
f1 (ppm)

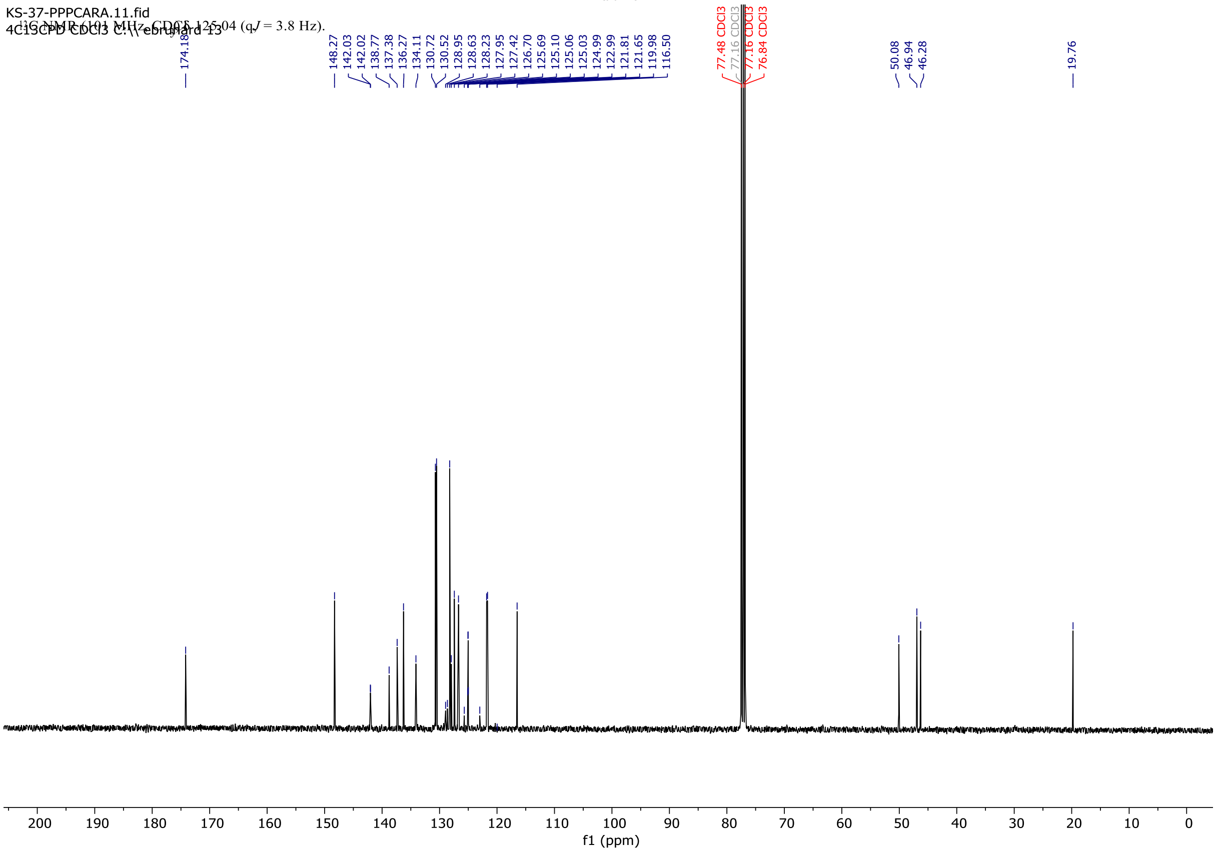
KS-37-PPPCARA.11.fid
174.18
148.27 142.03 142.02 138.77 137.38 136.27 134.11 130.72 130.52 128.95 128.63 128.23 127.95 127.42 126.70 125.69 125.10 125.06 125.03 124.99 122.99 121.81 121.65 119.98 116.50
77.48 CDCl3 77.16 CDCl3 77.16 CDCl3 76.84 CDCl3
50.08 46.94 46.28
19.76
200 190 180 170 160 150 140 130 120 110 100 90 80 70 60 50 40 30 20 10 0
f1 (ppm)

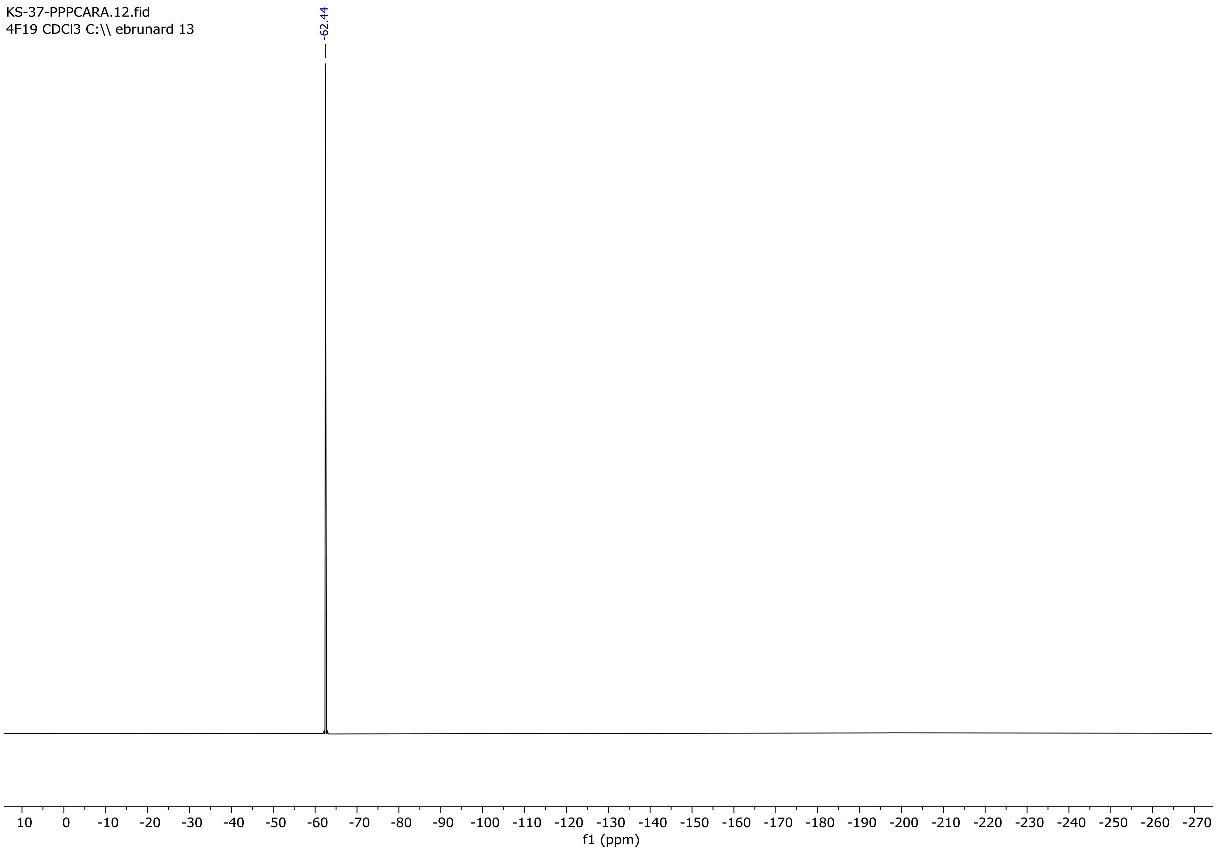
KS-37-PPPCARA.12.fid
4F19 CDCl3 C:\\ ebrunard 13
-62.44
10 0 -10 -20 -30 -40 -50 -60 -70 -80 -90 -100 -110 -120 -130 -140 -150 -160 -170 -180 -190 -200 -210 -220 -230 -240 -250 -260 -270
f1 (ppm)

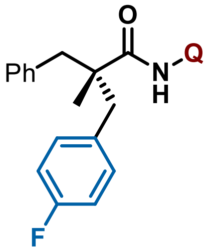

3ah

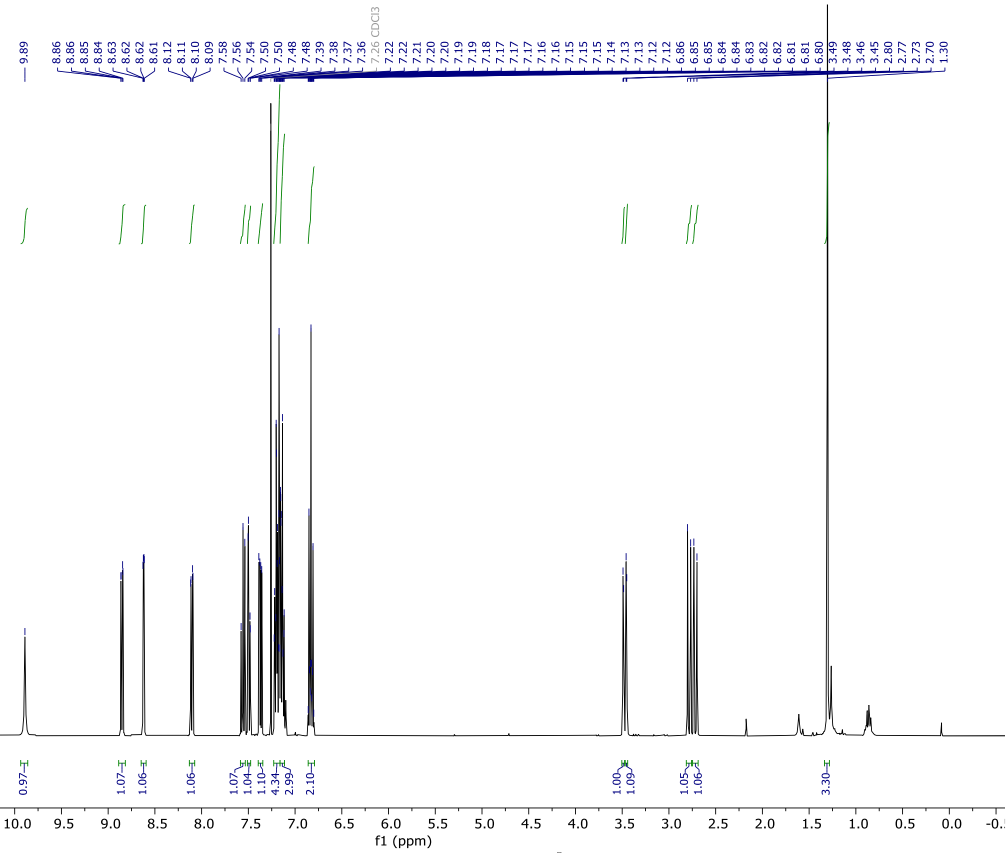
HF-562-PF-cara.10.fid
4PROTON CDCl3 C:\\ fhuang 9
f1 (ppm)

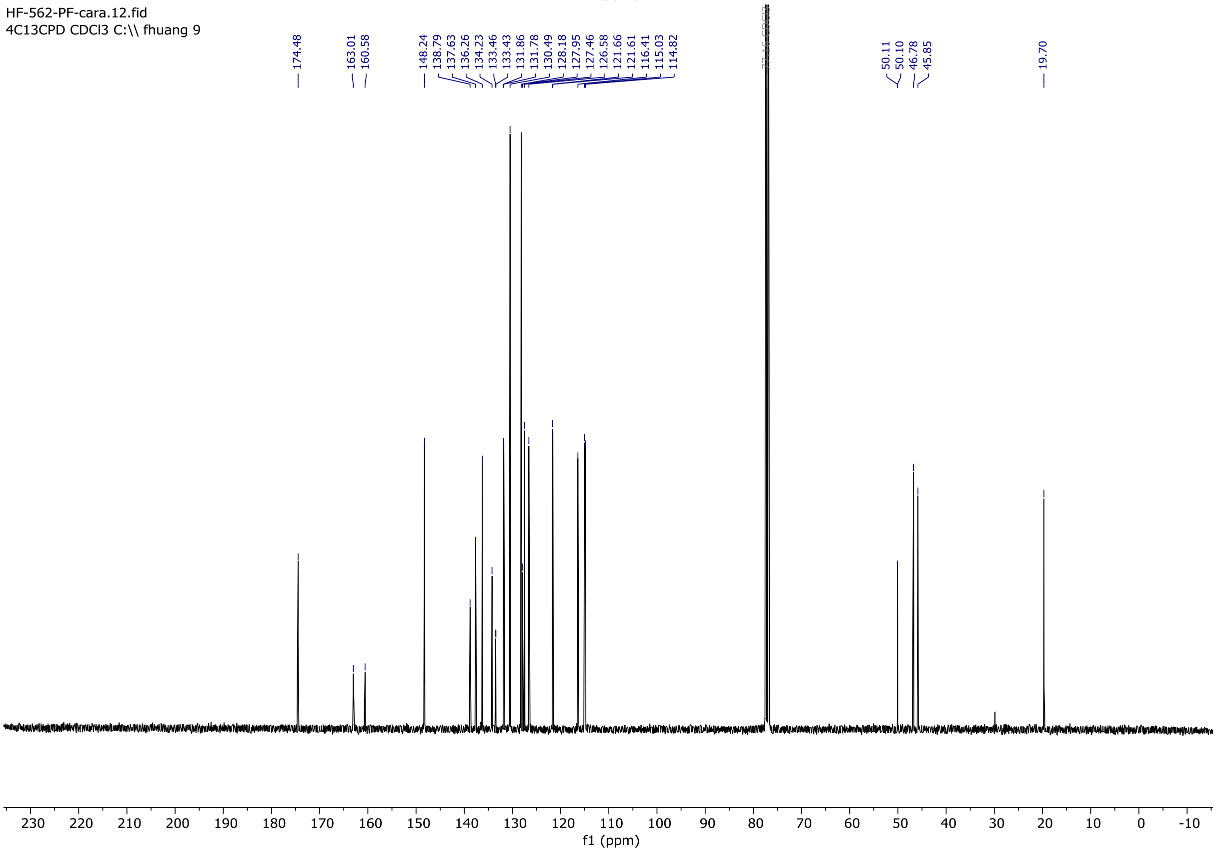
HF-562-PF-cara.12.fid
4C13CPD CDCl3 C:\\ fhuang 9
174.48
163.01
160.58
148.24
138.79
137.63
136.26
134.23
133.46
133.43
131.86
131.78
130.49
128.18
127.95
127.46
126.58
121.66
121.61
116.41
115.03
114.82
50.11
50.10
46.78
45.85
19.70
f1 (ppm)

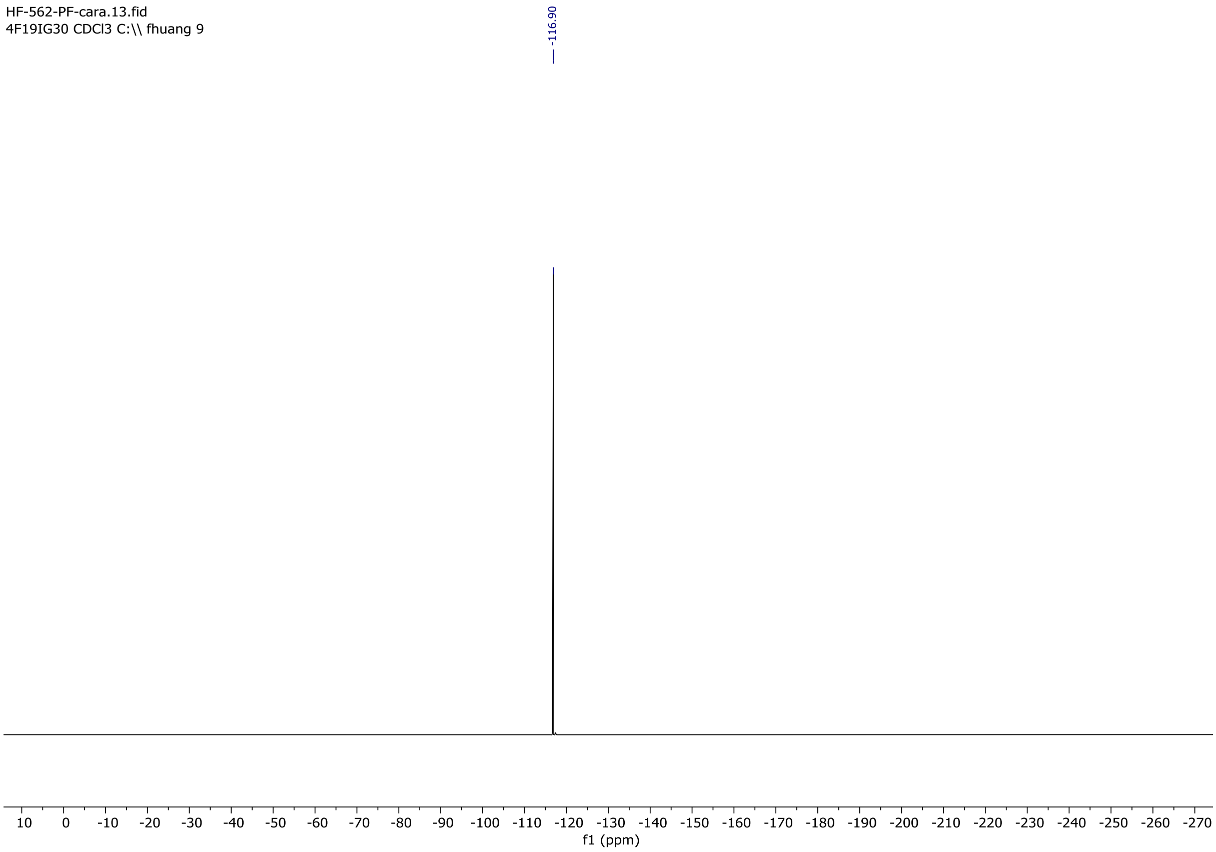
HF-562-PF-cara.13.fid
4F19IG30 CDCl3 C:\\ fhuang 9
-116.90
10 0 -10 -20 -30 -40 -50 -60 -70 -80 -90 -100 -110 -120 -130 -140 -150 -160 -170 -180 -190 -200 -210 -220 -230 -240 -250 -260 -270
f1 (ppm)

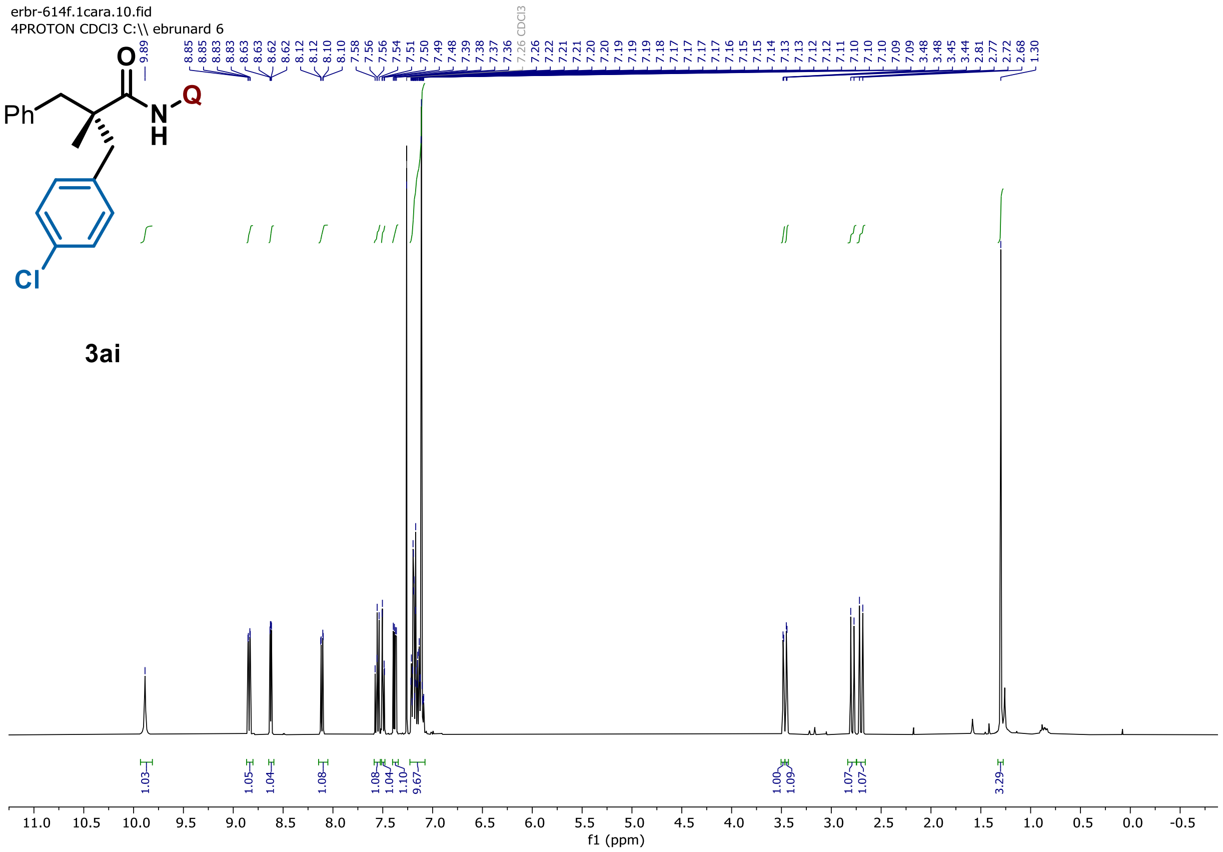
erbr-614f.1cara.10.fid
4PROTON CDCl3 C:\\ ebrunard 6
9.89
8.85 8.85 8.83 8.83 8.63 8.63 8.62 8.62 8.12 8.12 8.10 8.10 7.58 7.56 7.56 7.54 7.51 7.50 7.49 7.48 7.39 7.38 7.37 7.36
7.26 CDCl3
7.26 7.22 7.21 7.21 7.20 7.20 7.19 7.19 7.19 7.18 7.17 7.17 7.17 7.17 7.16 7.15 7.15 7.14 7.13 7.13 7.12 7.12 7.11 7.10 7.10 7.10 7.09 3.48 3.48 3.45 3.44 2.81 2.77 2.72 2.68 1.30
O
N
Q
H
Ph
Cl
3ai
1.03 1.05 1.04 1.08 1.08 1.04 1.10 9.67 1.00 1.09 1.07 1.07 3.29
11.0 10.5 10.0 9.5 9.0 8.5 8.0 7.5 7.0 6.5 6.0 5.5 5.0 4.5 4.0 3.5 3.0 2.5 2.0 1.5 1.0 0.5 0.0 -0.5
f1 (ppm)

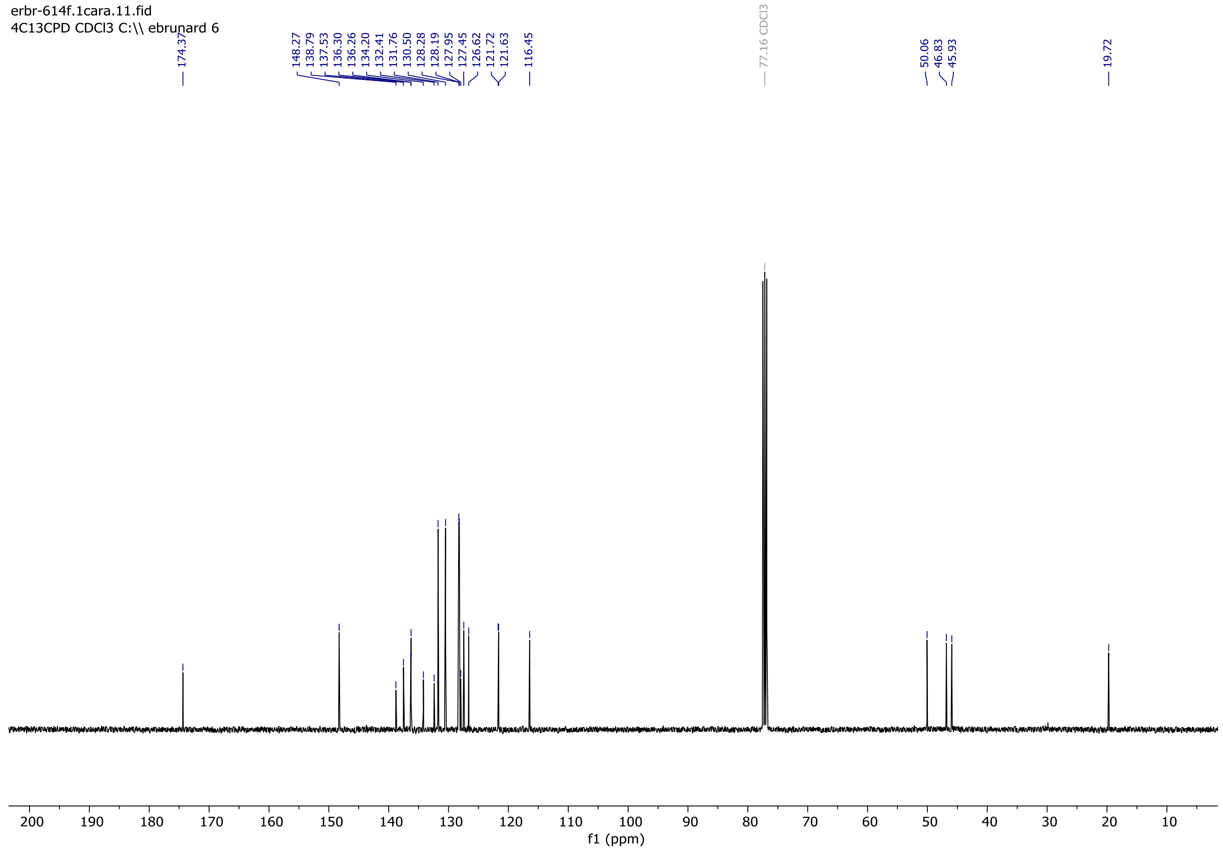
erbr-614f.1cara.11.fid
4C13CPD CDCl3 C:\\ ebrunard 6
174.37
148.27 138.79 137.53 136.30 136.26 134.20 132.41 131.76 130.50 128.28 128.19 127.95 127.45 126.62 121.72 121.63
116.45
77.16 CDCl3
50.06 46.83 45.93
19.72
200 190 180 170 160 150 140 130 120 110 100 90 80 70 60 50 40 30 20 10
f1 (ppm)

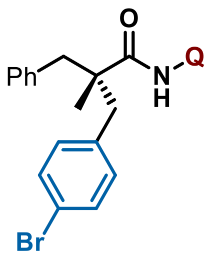
Ph
O
N
H
Q
Br

3aj

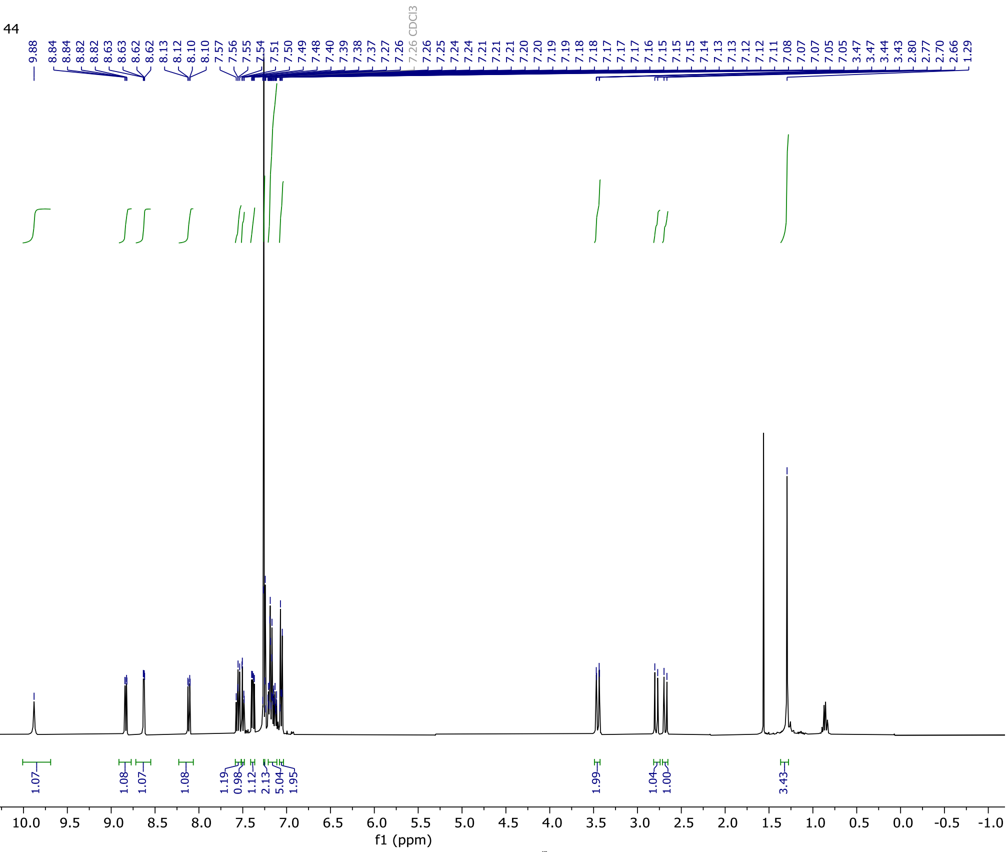
erbr-609f1carabis.10.fid
4PROTON CDCl3 C:\\ ebrunard 44
9.88
8.84
8.84
8.82
8.82
8.63
8.63
8.62
8.62
8.13
8.12
8.10
8.10
7.57
7.56
7.55
7.54
7.51
7.50
7.49
7.48
7.40
7.39
7.38
7.37
7.27
7.26 CDCl3
7.26
7.25
7.24
7.24
7.21
7.21
7.21
7.20
7.19
7.19
7.18
7.18
7.17
7.17
7.17
7.16
7.15
7.15
7.15
7.14
7.13
7.13
7.12
7.12
7.11
7.08
7.07
7.07
7.05
7.05
3.47
3.44
3.43
2.80
2.77
2.70
2.66
1.29
1.07
1.08
1.07
1.08
1.19
0.98
1.12
2.13
5.04
1.95
1.99
1.04
1.00
3.43
12.5
12.0
11.5
11.0
10.5
10.0
9.5
9.0
8.5
8.0
7.5
7.0
6.5
6.0
5.5
5.0
4.5
4.0
3.5
3.0
2.5
2.0
1.5
1.0
0.5
0.0
-0.5
-1.0
f1 (ppm)

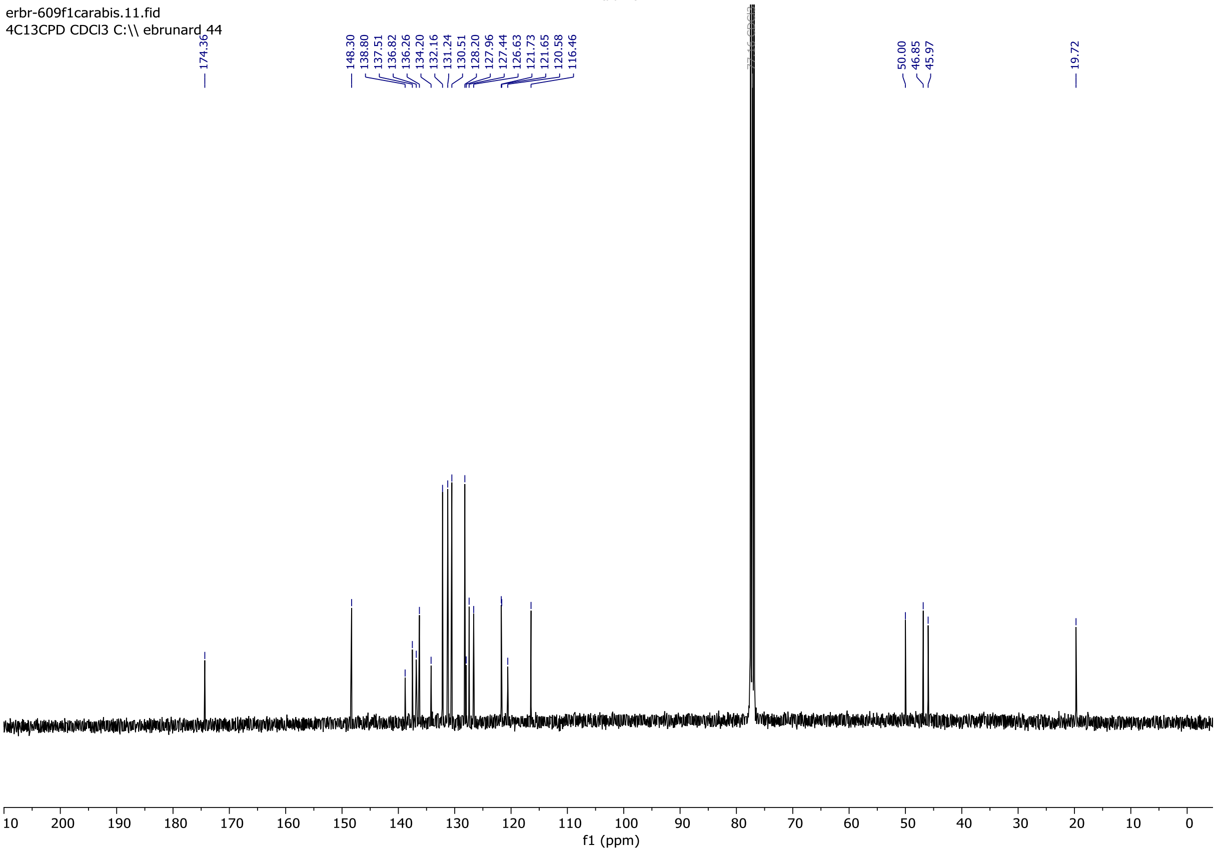
erbr-609f1carabis.11.fid
4C13CPD CDCl3 C:\\ ebrunard 44
174.36
148.30
138.80
137.51
136.82
136.26
134.20
132.16
131.24
130.51
128.20
127.96
127.44
126.63
121.73
121.65
120.58
116.46
50.00
46.85
45.97
19.72
210
200
190
180
170
160
150
140
130
120
110
100
90
80
70
60
50
40
30
20
10
0
f1 (ppm)

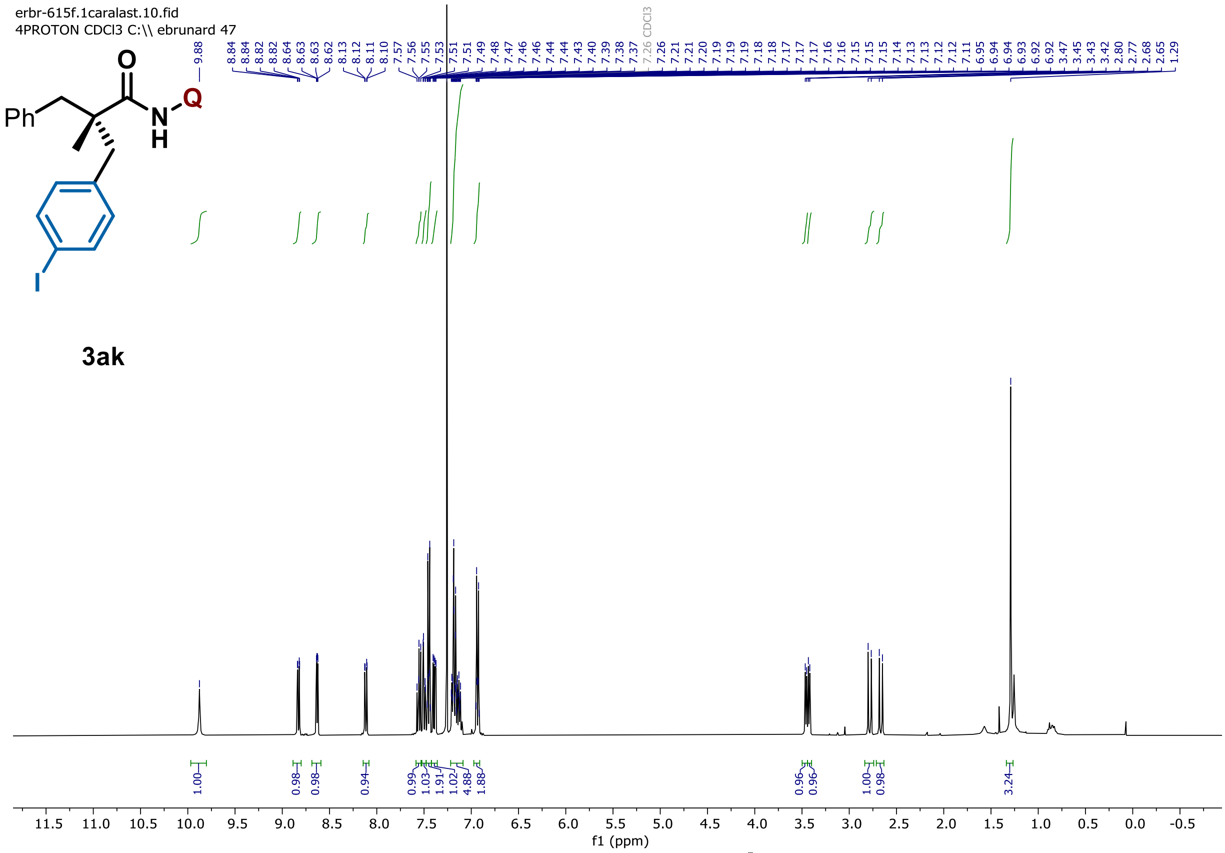
erbr-615f.1caralast.10.fid
4PROTON CDCl3 C:\\ ebrunard 47
O
Ph
N
Q
H
I
3ak
9.88
8.84 8.84 8.82 8.64 8.63 8.63 8.62 8.13 8.12 8.11 8.10 7.57 7.56 7.55 7.53 7.51 7.51 7.49 7.48 7.47 7.46 7.46 7.44 7.44 7.43 7.40 7.39 7.38 7.37
7.26 CDCl3
7.26 7.21 7.21 7.20 7.19 7.19 7.19 7.18 7.18 7.17 7.17 7.17 7.16 7.15 7.15 7.15 7.14 7.13 7.13 7.12 7.12 7.11 6.95 6.94 6.94 6.93 6.92 6.92 3.47 3.45 3.43 3.42 2.80 2.77 2.68 2.65 1.29
1.00 0.98 0.98 0.94 0.99 1.03 1.91 1.02 4.88 1.88 0.96 0.96 1.00 0.98 3.24
11.5 11.0 10.5 10.0 9.5 9.0 8.5 8.0 7.5 7.0 6.5 6.0 5.5 5.0 4.5 4.0 3.5 3.0 2.5 2.0 1.5 1.0 0.5 0.0 -0.5
f1 (ppm)

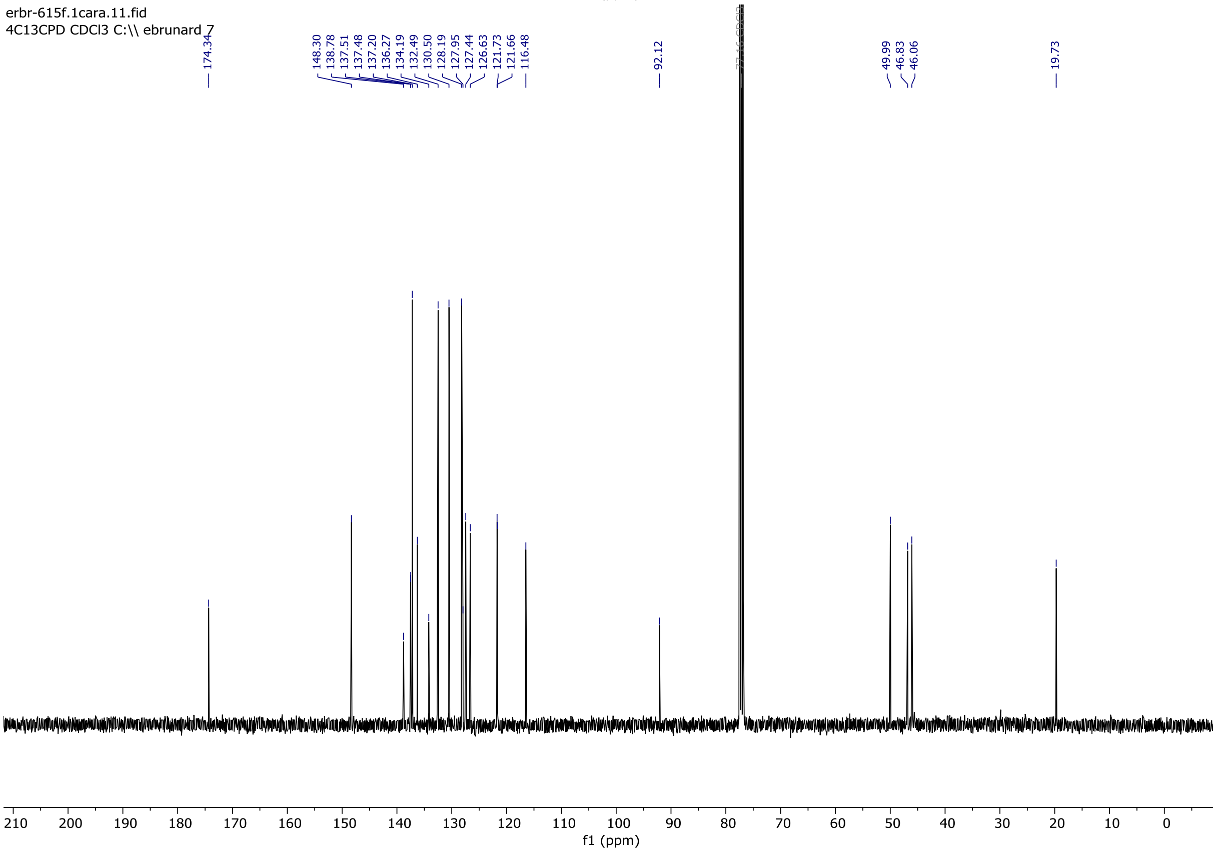
erbr-615f.1cara.11.fid
4C13CPD CDCl3 C:\\ ebrunard 7
174.34
148.30 138.78 137.51 137.48 137.20 136.27 134.19 132.49 130.50 128.19 127.95 127.44 126.63 121.73 121.66 116.48
92.12
49.99 46.83 46.06
19.73
210 200 190 180 170 160 150 140 130 120 110 100 90 80 70 60 50 40 30 20 10 0
f1 (ppm)

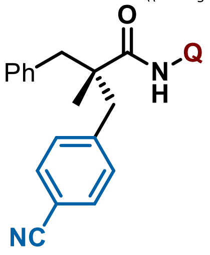

3al

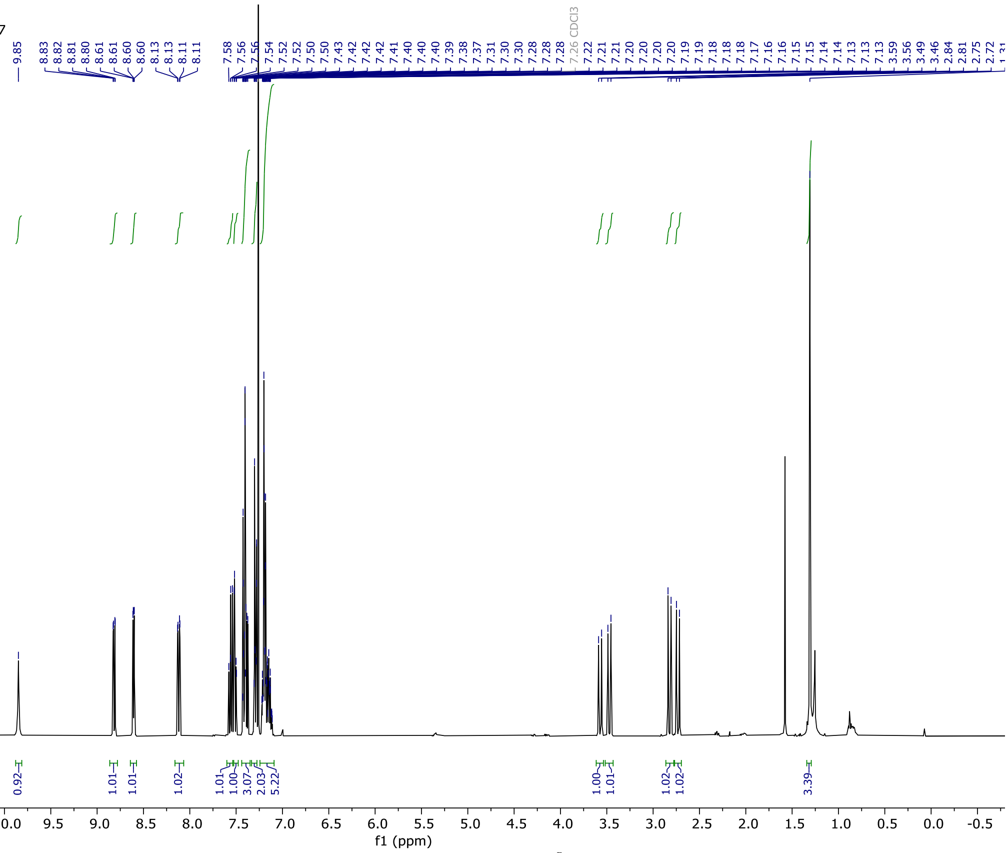

HF-635-CN-CARA.10.fid
4PROTON CDCl3 C:\\ fhuang 47
9.85
8.83
8.82
8.81
8.80
8.61
8.61
8.60
8.60
8.13
8.13
8.11
8.11
7.58
7.56
7.56
7.54
7.52
7.52
7.50
7.50
7.43
7.42
7.42
7.41
7.40
7.40
7.39
7.38
7.37
7.31
7.30
7.28
7.28
7.26 CDCl3
7.22
7.21
7.21
7.20
7.20
7.20
7.19
7.19
7.18
7.18
7.18
7.17
7.16
7.16
7.15
7.15
7.14
7.14
7.13
7.13
7.13
3.59
3.56
3.49
3.46
2.84
2.81
2.75
2.72
0.92
1.01
1.01
1.02
1.01
1.00
3.07
2.03
5.22
1.00
1.01
1.02
1.02
3.39
12.0 11.5 11.0 10.5 10.0 9.5 9.0 8.5 8.0 7.5 7.0 6.5 6.0 5.5 5.0 4.5 4.0 3.5 3.0 2.5 2.0 1.5 1.0 0.5 0.0 -0.5
f1 (ppm)

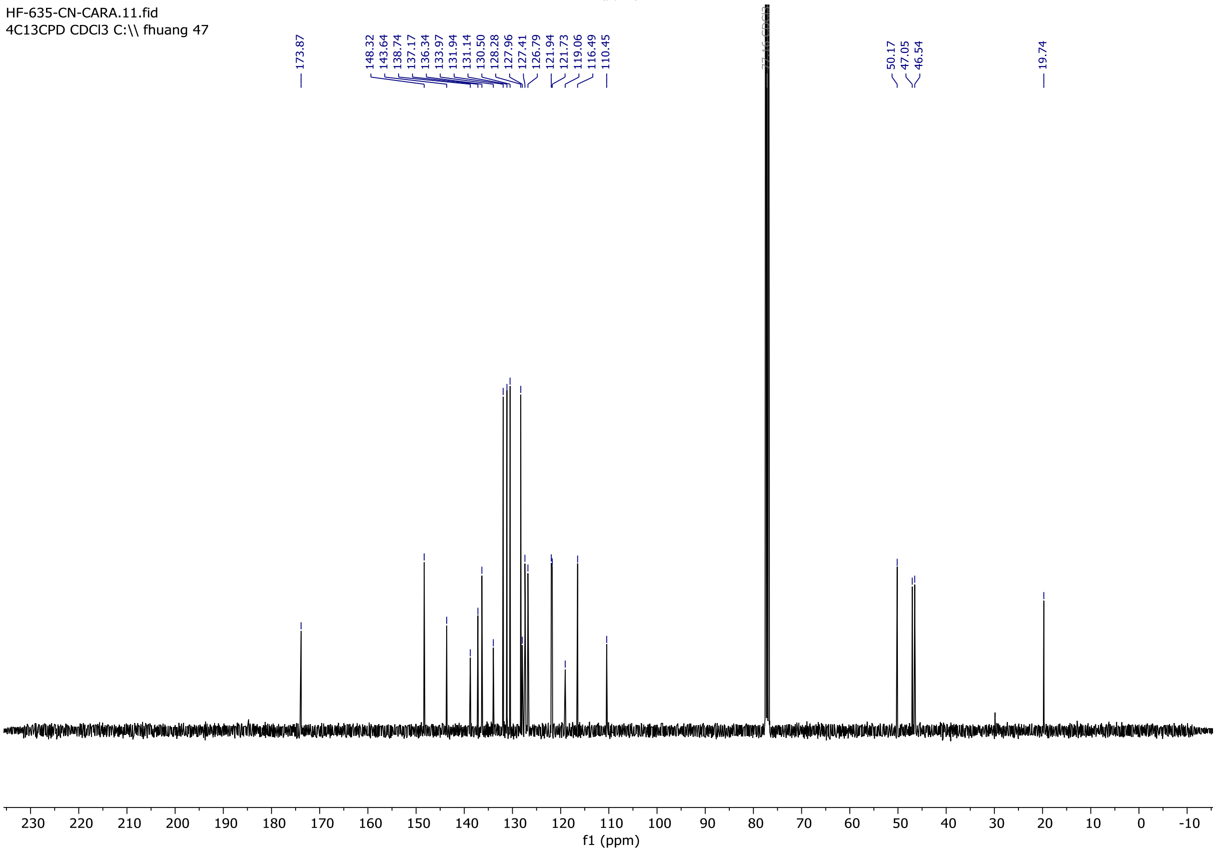

HF-635-CN-CARA.11.fid
4C13CPD CDCl3 C:\\ fhuang 47
173.87
148.32
143.64
138.74
137.17
136.34
133.97
131.94
131.14
130.50
128.28
127.96
127.41
126.79
121.94
121.73
119.06
116.49
110.45
50.17
47.05
46.54
19.74
230 220 210 200 190 180 170 160 150 140 130 120 110 100 90 80 70 60 50 40 30 20 10 0 -10
f1 (ppm)

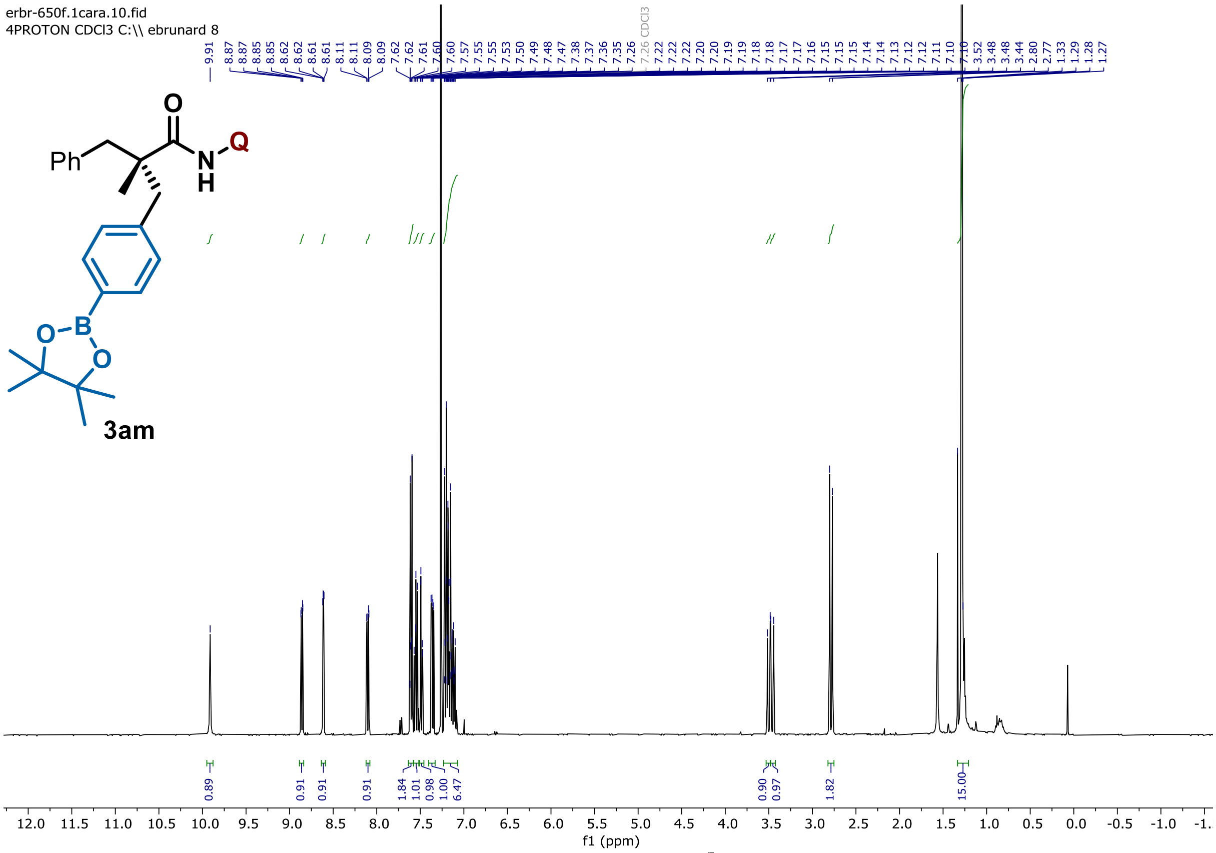
erbr-650f.1cara.10.fid
4PROTON CDCl3 C:\\ ebrunard 8
9.91 8.87 8.87 8.85 8.85 8.62 8.62 8.61 8.61 8.11 8.11 8.09 8.09 7.62 7.62 7.61 7.60 7.60 7.57 7.55 7.55 7.53 7.50 7.49 7.48 7.47 7.38 7.37 7.36 7.35 7.26 CDCl3 7.22 7.22 7.22 7.20 7.20 7.19 7.19 7.18 7.18 7.17 7.17 7.16 7.15 7.15 7.15 7.14 7.13 7.12 7.12 7.11 7.10 7.10 3.52 3.48 3.48 3.44 2.80 2.77 1.33 1.29 1.28 1.27
Ph
O
N
H
Q
O
B
O
3am
0.89 0.91 0.91 0.91 1.84 1.01 0.98 1.00 6.47 0.90 0.97 1.82 15.00
f1 (ppm)

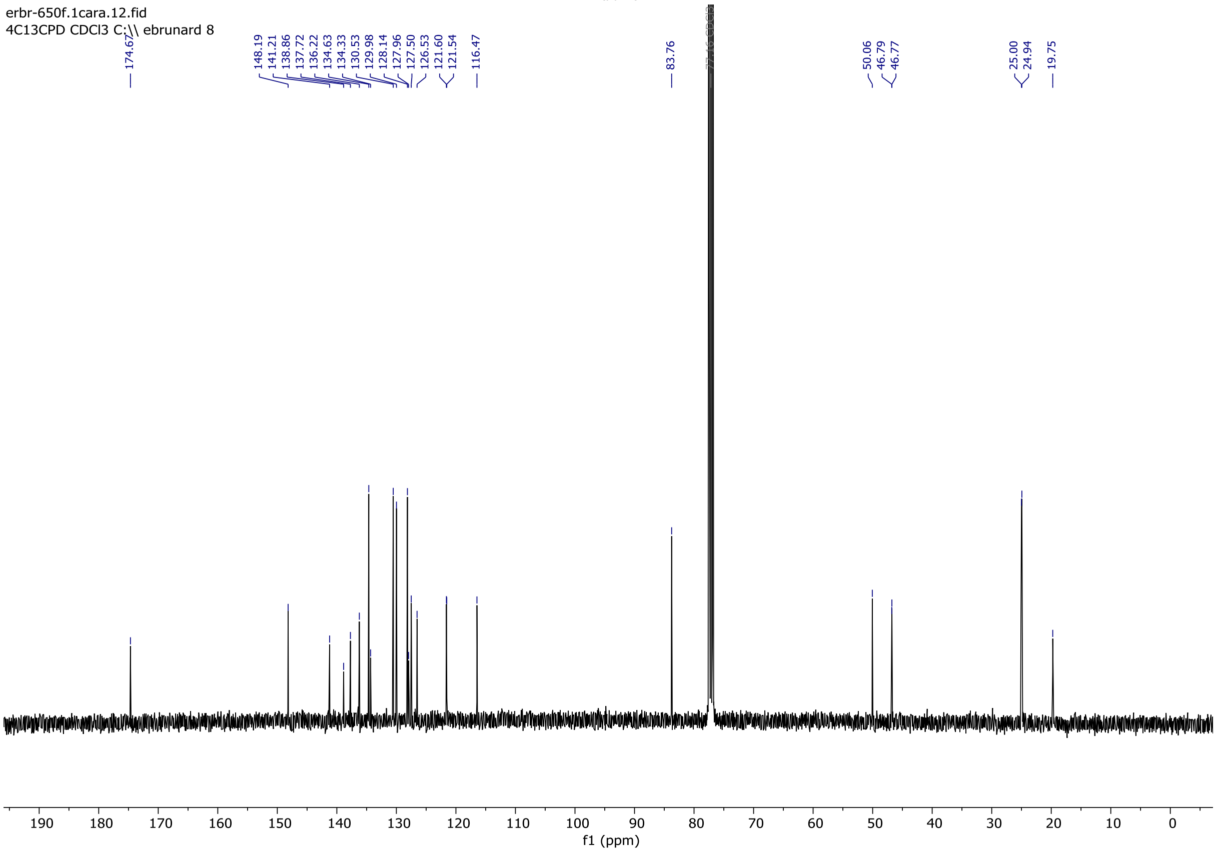
erbr-650f.1cara.12.fid
4C13CPD CDCl3 C:\\ ebrunard 8
174.67 148.19 141.21 138.86 137.72 136.22 134.63 134.33 130.53 129.98 128.14 127.96 127.50 126.53 121.60 121.54 116.47 83.76 50.06 46.79 46.77 25.00 24.94 19.75
f1 (ppm)

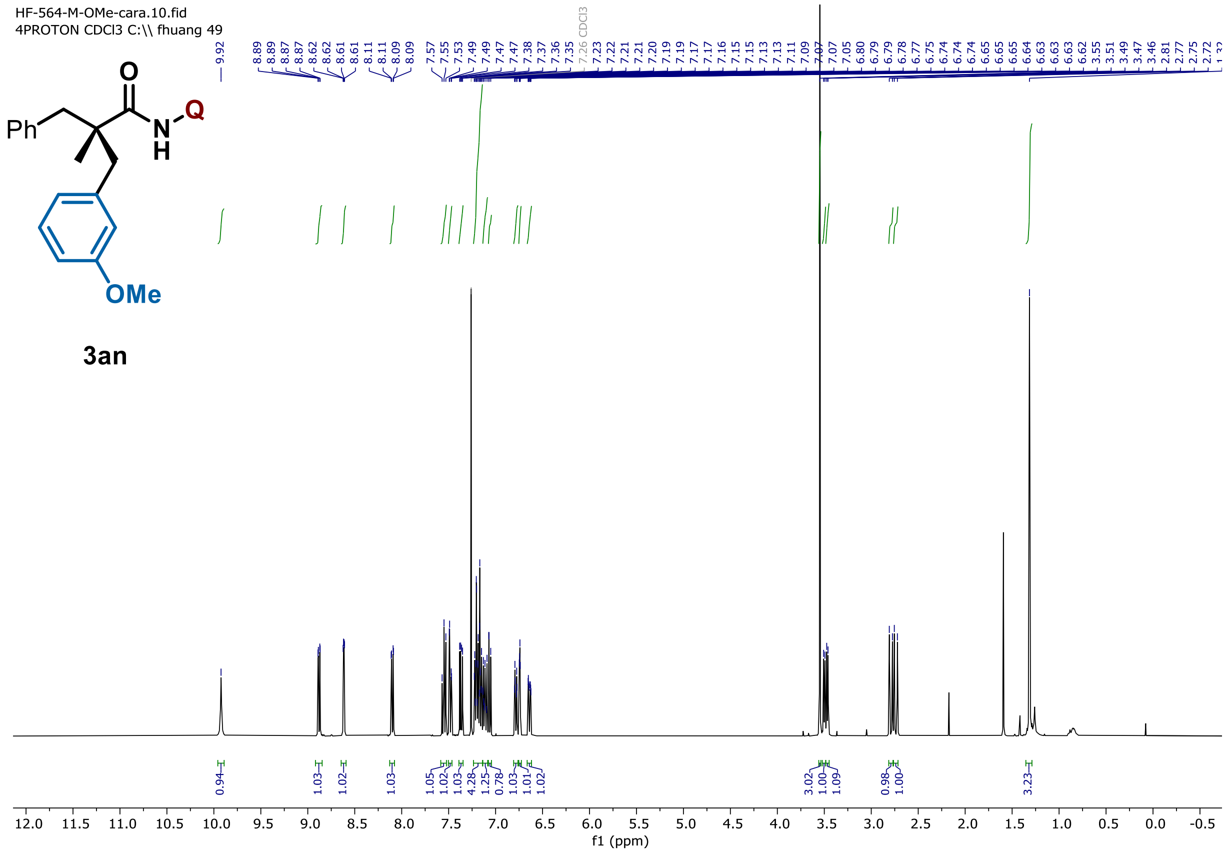
HF-564-M-OMe-cara.10.fid
4PROTON CDCl3 C:\\ fhuang 49
3an
f1 (ppm)

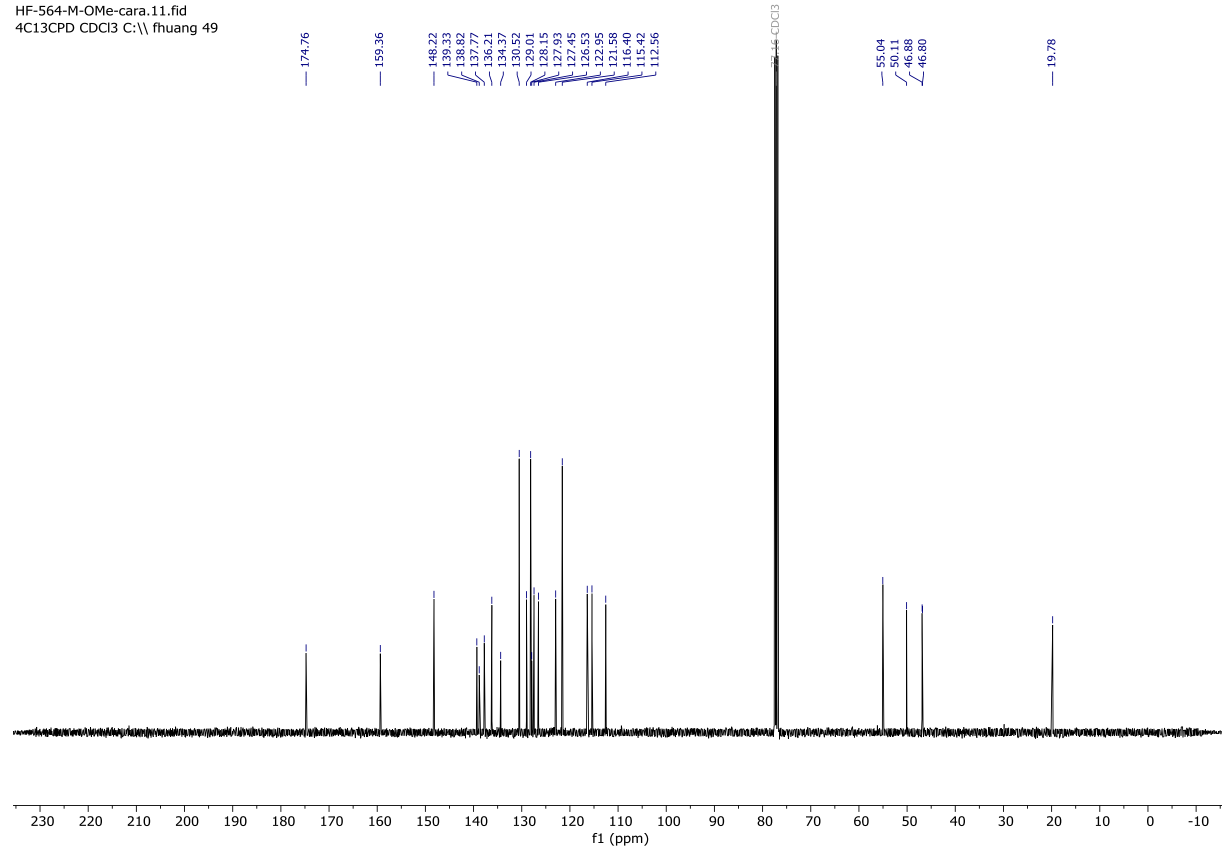
HF-564-M-OMe-cara.11.fid
4C13CPD CDCl3 C:\\ fhuang 49
174.76
159.36
148.22
139.33
138.82
137.77
136.21
134.37
130.52
129.01
128.15
127.93
127.45
126.53
122.95
121.58
116.40
115.42
112.56
55.04
50.11
46.88
46.80
19.78
f1 (ppm)

erbr-633f.1caralast.10.fid
4PROTON CDCl3 C:\\ ebrunard 50

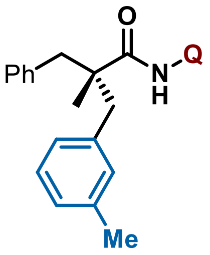

3ao

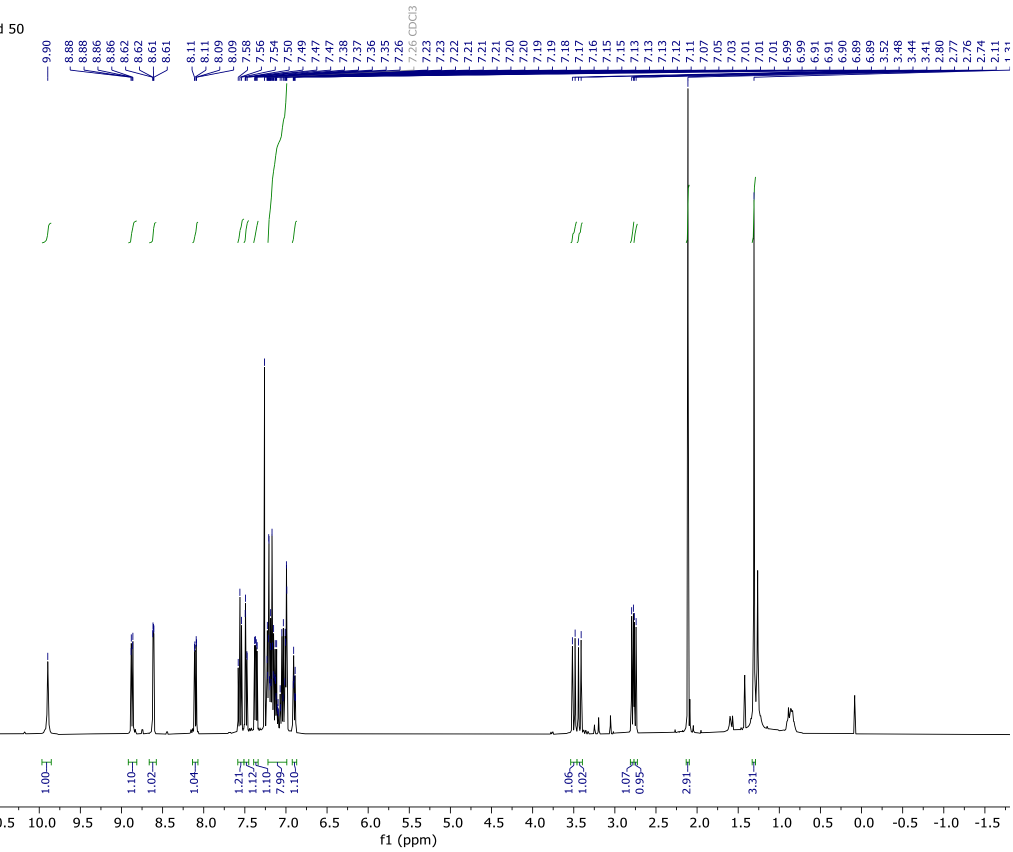

erbr-633f.1cara.11.fid
4C13CPD CDCl3 C:\\ ebrunard 3

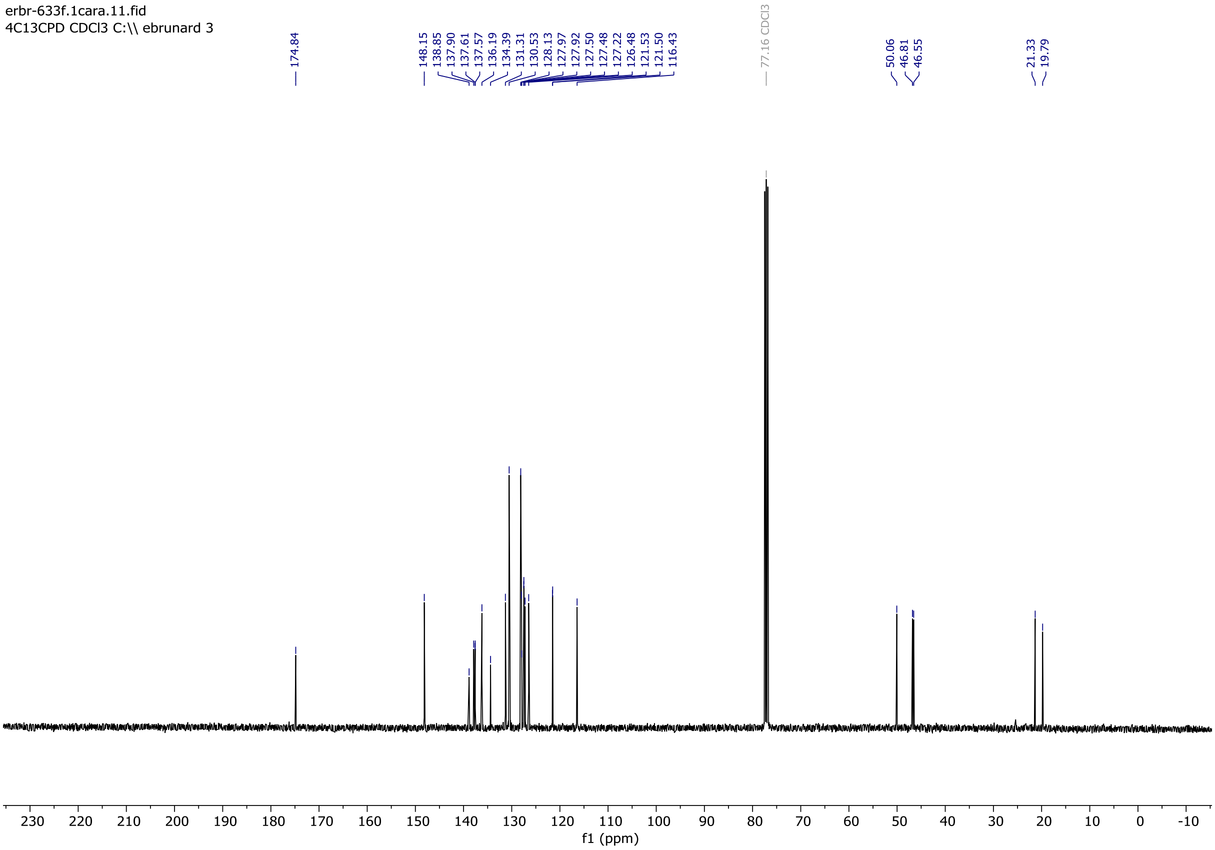

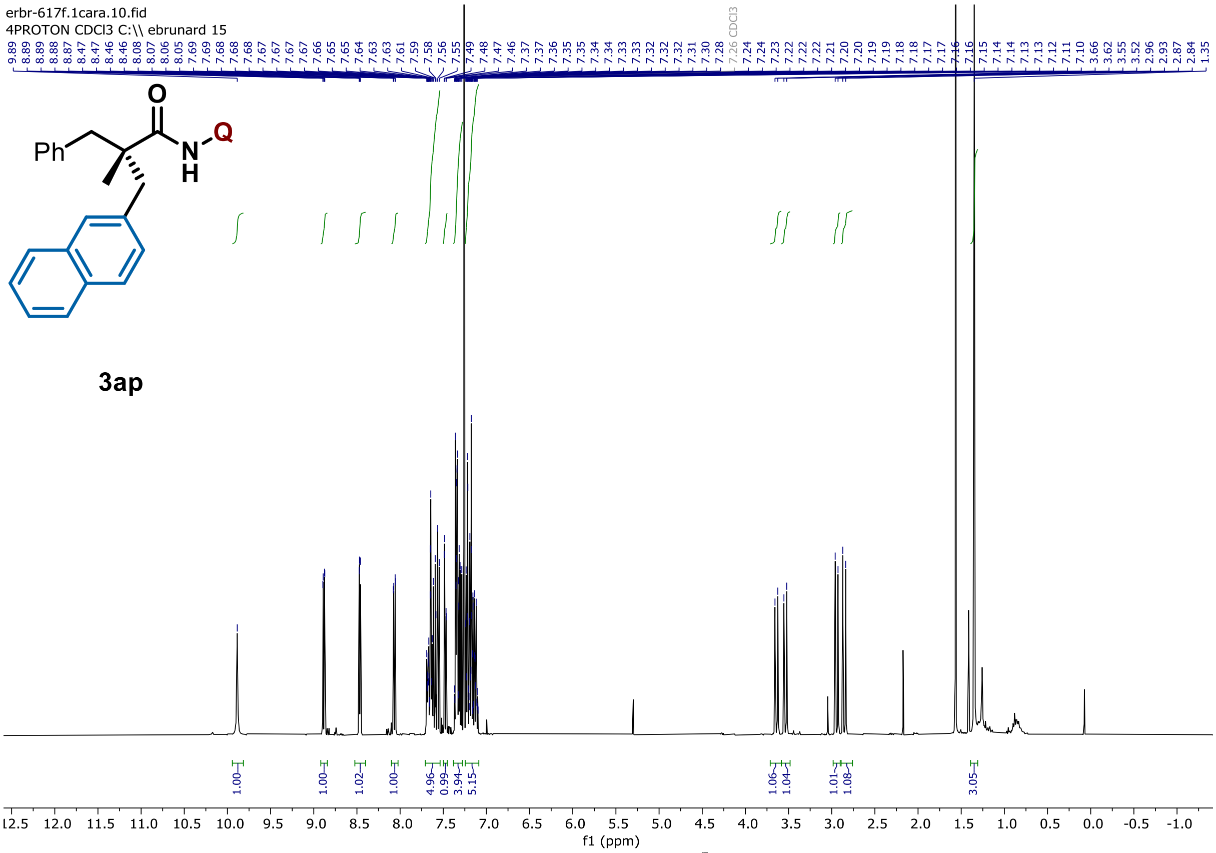

erbr-617f.1cara.10.fid
4PROTON CDCl3 C:\\ ebrunard 15
9.89 8.89 8.89 8.88 8.87 8.47 8.47 8.46 8.46 8.08 8.07 8.06 8.05 7.69 7.69 7.68 7.68 7.67 7.67 7.67 7.66 7.65 7.65 7.64 7.63 7.63 7.61 7.59 7.58 7.56 7.55 7.49 7.48 7.47 7.46 7.37 7.37 7.36 7.35 7.35 7.34 7.34 7.33 7.33 7.32 7.32 7.32 7.31 7.30 7.28 7.26 CDCl3 7.24 7.24 7.23 7.22 7.22 7.22 7.22 7.21 7.21 7.20 7.20 7.19 7.19 7.18 7.18 7.17 7.17 7.16 7.16 7.15 7.14 7.14 7.13 7.13 7.12 7.11 7.10 3.66 3.62 3.55 3.52 2.96 2.93 2.87 2.84 1.35
1.00 1.00 1.02 1.00 4.96 0.99 3.94 5.15 1.06 1.04 1.01 1.08 3.05
f1 (ppm)
Ph
O
N
Q
H

3ap

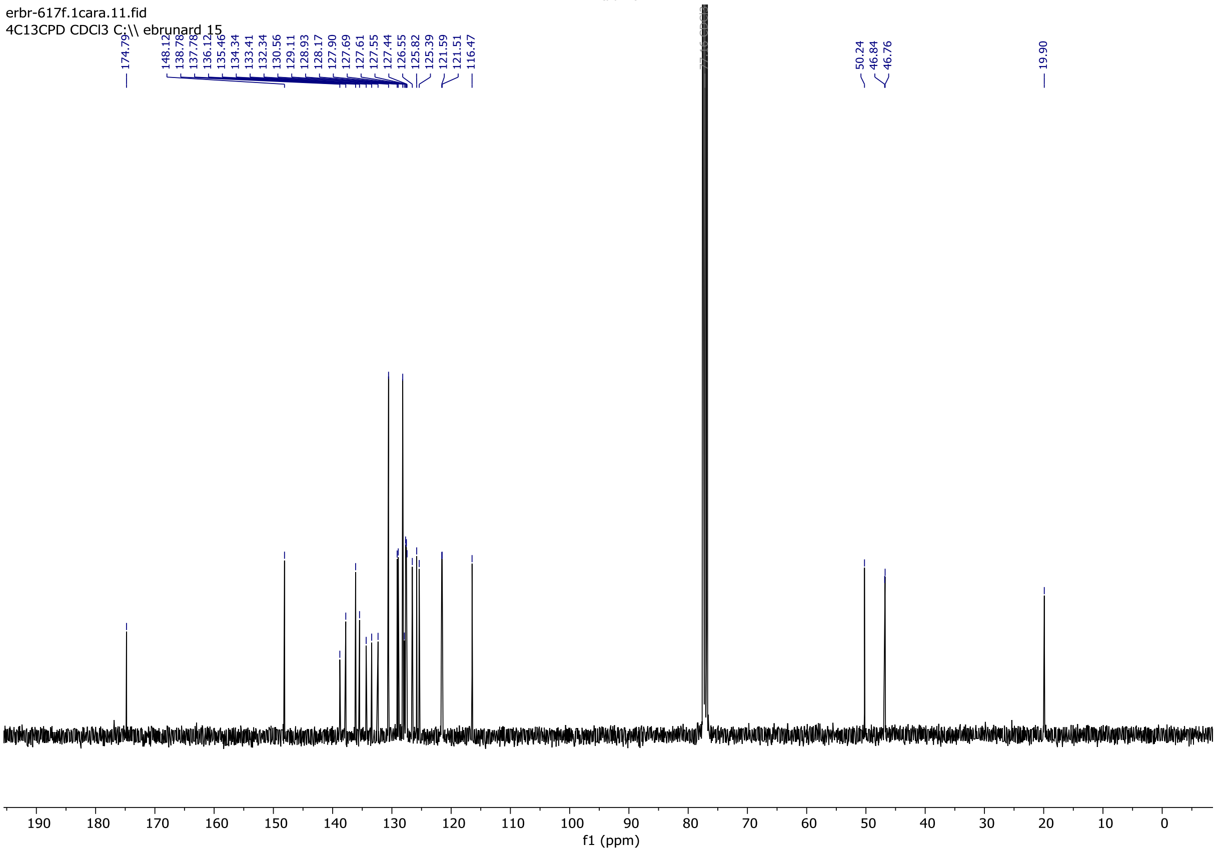

erbr-617f.1cara.11.fid
4C13CPD CDCl3 C:\\ ebrunard 15
174.79 148.12 138.78 137.78 136.12 135.46 134.34 133.41 132.34 130.56 129.11 128.93 128.17 127.90 127.69 127.61 127.55 127.44 126.55 125.82 125.39 121.59 121.51 116.47 50.24 46.84 46.76 19.90
f1 (ppm)

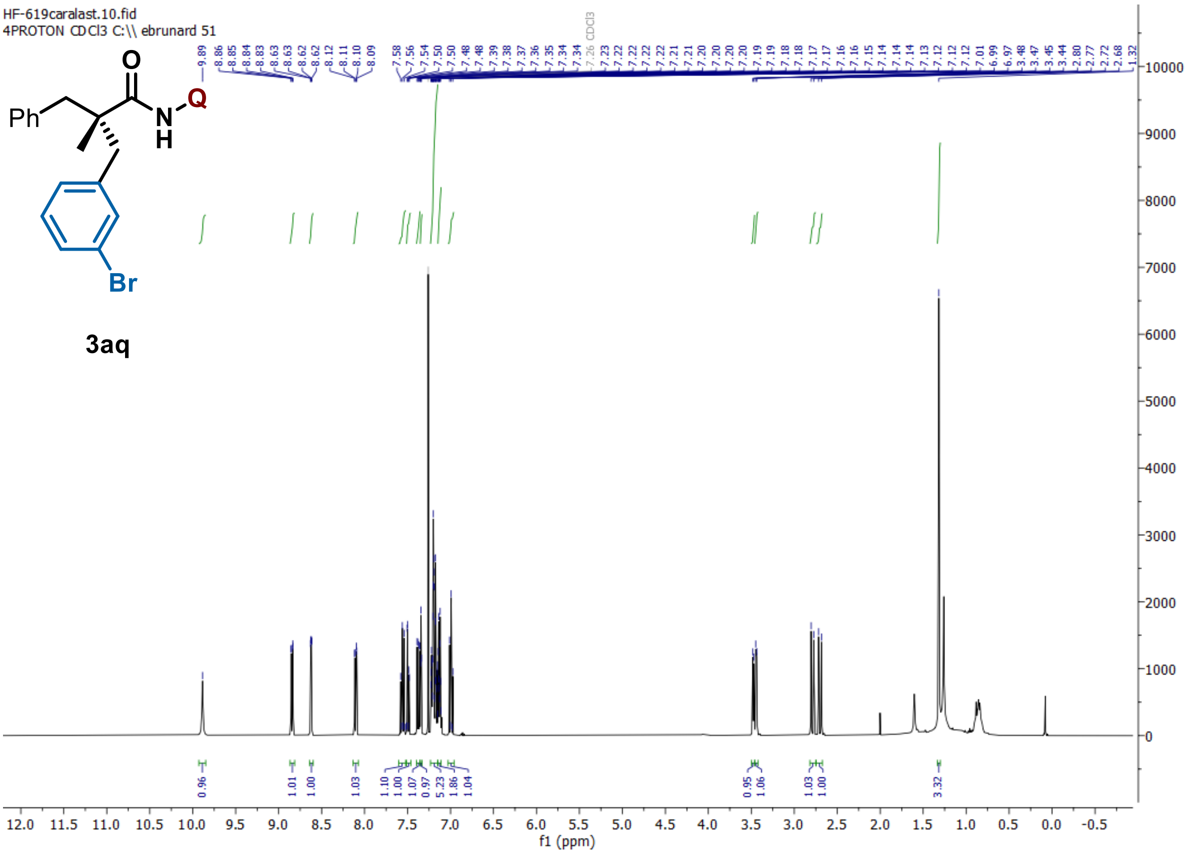
Ph
O
N
H
Q
Br
3aq
HF-619caralast.10.fid
4PROTON CDCl3 C:\\ ebrunard 51
9.89
8.86
8.85
8.84
8.83
8.63
8.63
8.62
8.62
8.12
8.11
8.10
8.09
7.58
7.56
7.54
7.50
7.50
7.48
7.48
7.39
7.38
7.37
7.36
7.35
7.34
7.34
7.26 CDCl3
7.23
7.22
7.22
7.22
7.22
7.21
7.21
7.20
7.20
7.20
7.19
7.19
7.18
7.18
7.17
7.17
7.16
7.16
7.15
7.14
7.14
7.14
7.13
7.12
7.12
7.12
7.01
6.99
6.97
3.48
3.47
3.45
3.44
2.80
2.77
2.72
2.68
1.32
0.96
1.01
1.00
1.03
1.10
1.00
1.07
0.97
5.23
1.86
1.04
0.95
1.06
1.03
1.00
3.32
f1 (ppm)

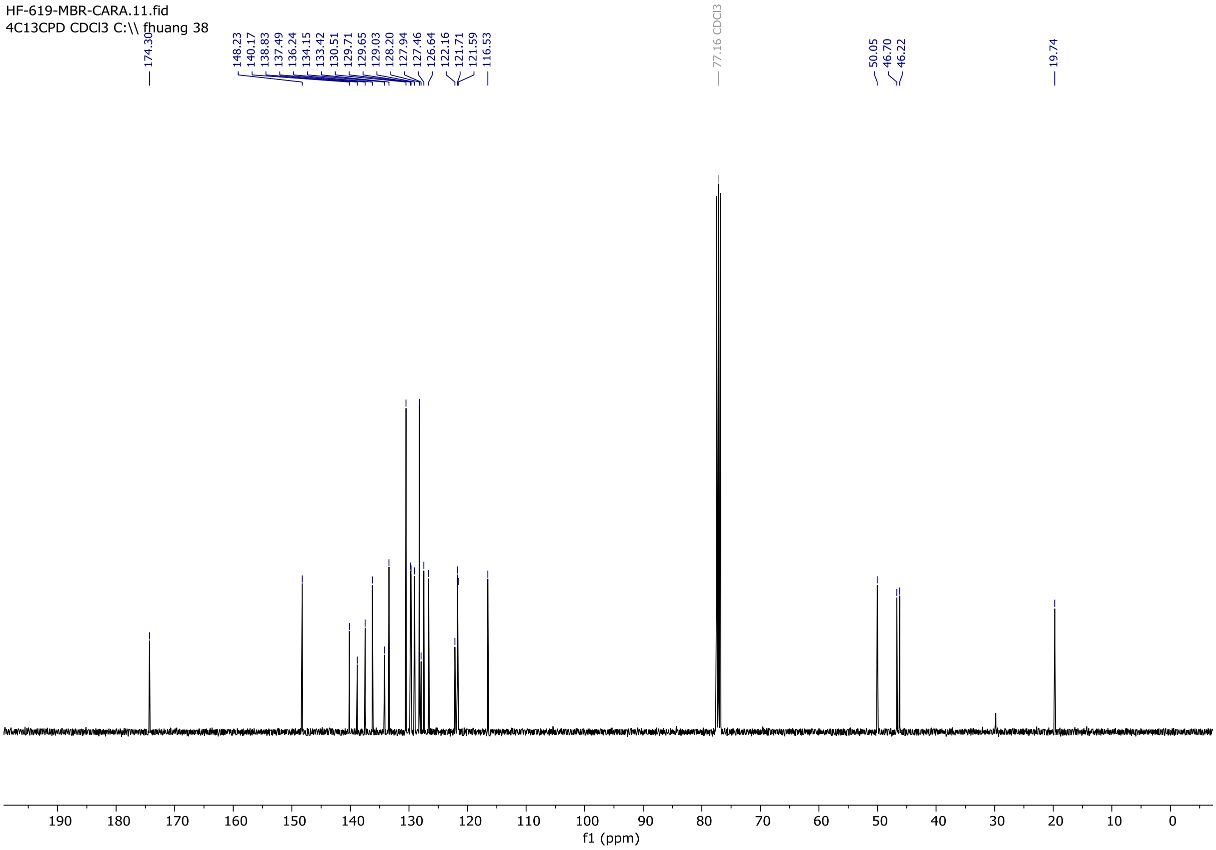
HF-619-MBR-CARA.11.fid
4C13CPD CDCl3 C:\\ fhuang 38
174.30
148.23
140.17
138.83
137.49
136.24
134.15
133.42
130.51
129.71
129.65
129.03
128.20
127.94
127.46
126.64
122.16
121.71
121.59
116.53
77.16 CDCl3
50.05
46.70
46.22
19.74
f1 (ppm)

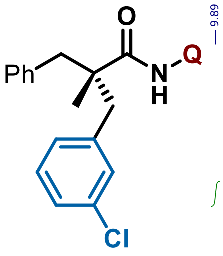
Ph
O
N
Q
H
Cl

3ar

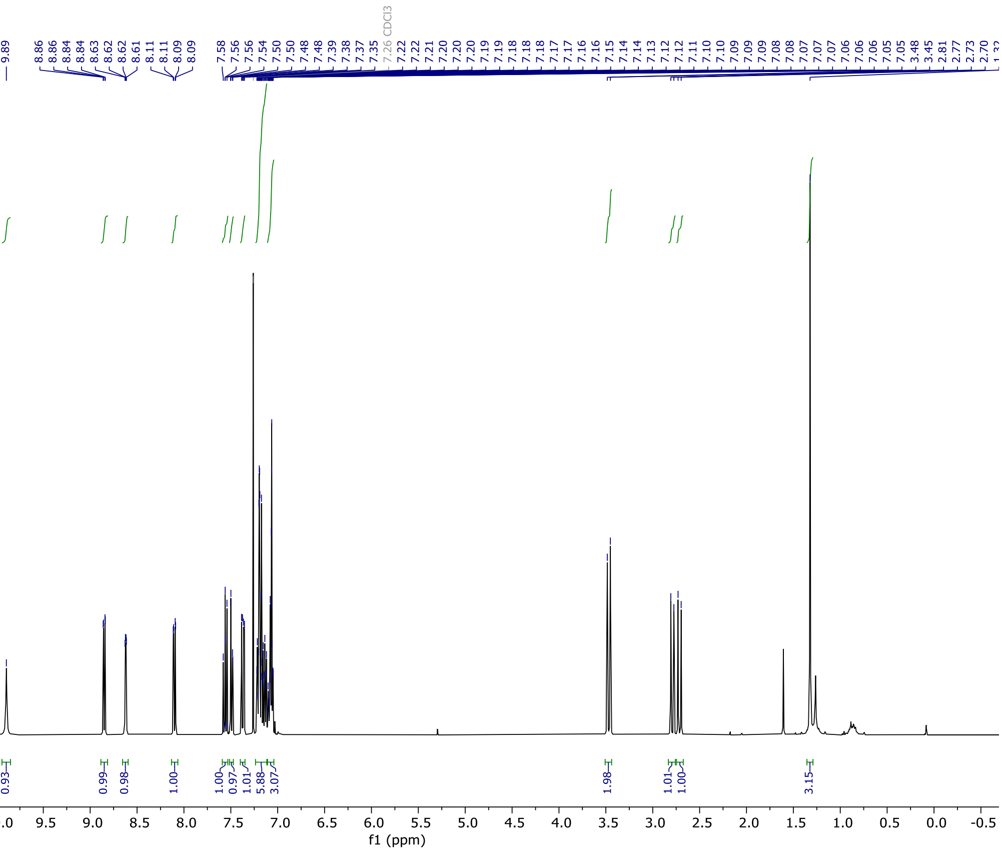
HF-620-MCL-CARA.10.fid
4PROTON CDCl3 C:\\ fhuang 39
9.89
8.86 8.86 8.84 8.84 8.63 8.62 8.62 8.61 8.11 8.11 8.09 8.09
7.58 7.56 7.56 7.54 7.50 7.50 7.48 7.48 7.39 7.38 7.37 7.35
7.26 CDCl3
7.22 7.22 7.22 7.21 7.20 7.20 7.19 7.19 7.18 7.18 7.18 7.17 7.17 7.16 7.16 7.15 7.14 7.14 7.13 7.12 7.12 7.11 7.10 7.10 7.09 7.09 7.09 7.08 7.08 7.07 7.07 7.06 7.06 7.06 7.05 7.05
3.48 3.45 2.81 2.77 2.73 2.70
0.93 0.99 0.98 1.00 1.00 0.97 1.01 5.88 3.07 1.98 1.01 1.00 3.15
12.0 11.5 11.0 10.5 10.0 9.5 9.0 8.5 8.0 7.5 7.0 6.5 6.0 5.5 5.0 4.5 4.0 3.5 3.0 2.5 2.0 1.5 1.0 0.5 0.0 -0.5
f1 (ppm)

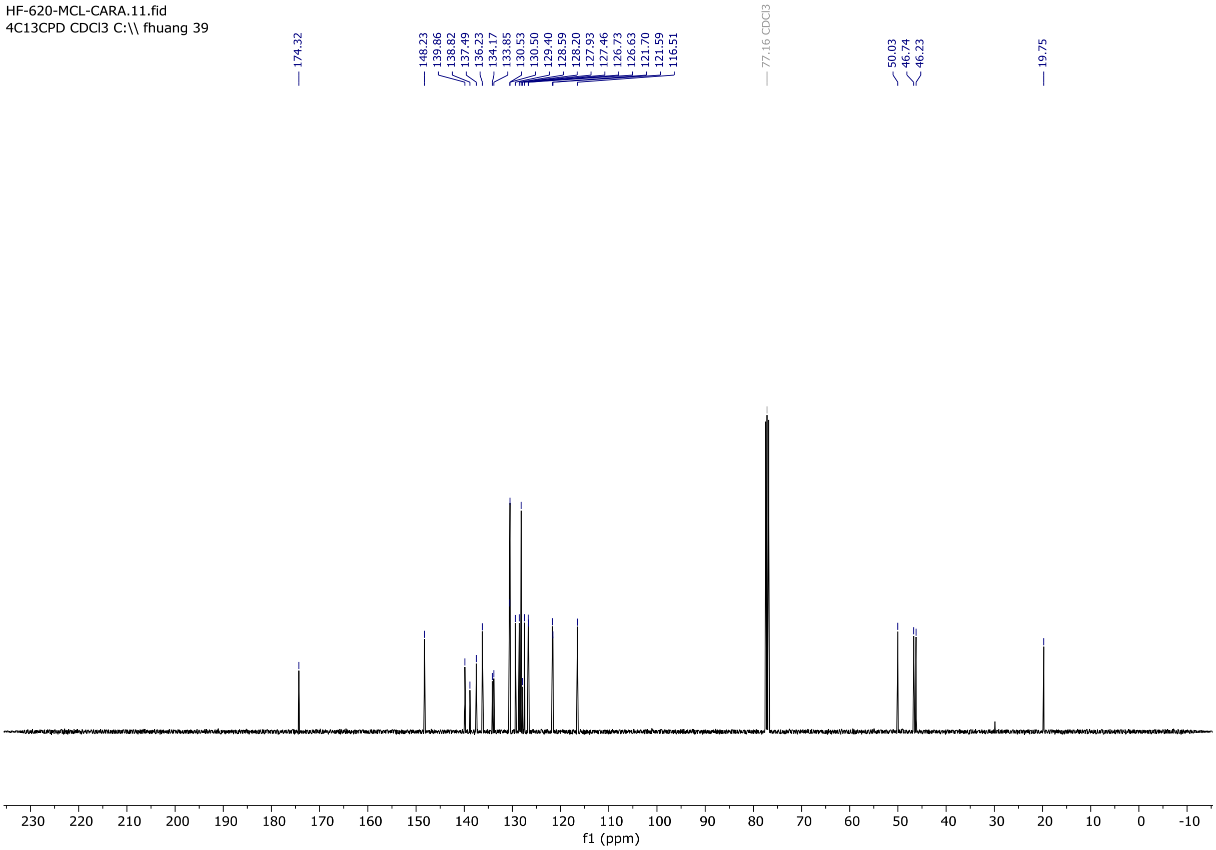
HF-620-MCL-CARA.11.fid
4C13CPD CDCl3 C:\\ fhuang 39
174.32
148.23 139.86 138.82 137.49 136.23 134.17 133.85 130.53 130.50 129.40 128.59 128.20 127.93 127.46 126.73 126.63 121.70 121.59 116.51
77.16 CDCl3
50.03 46.74 46.23
19.75
230 220 210 200 190 180 170 160 150 140 130 120 110 100 90 80 70 60 50 40 30 20 10 0 -10
f1 (ppm)

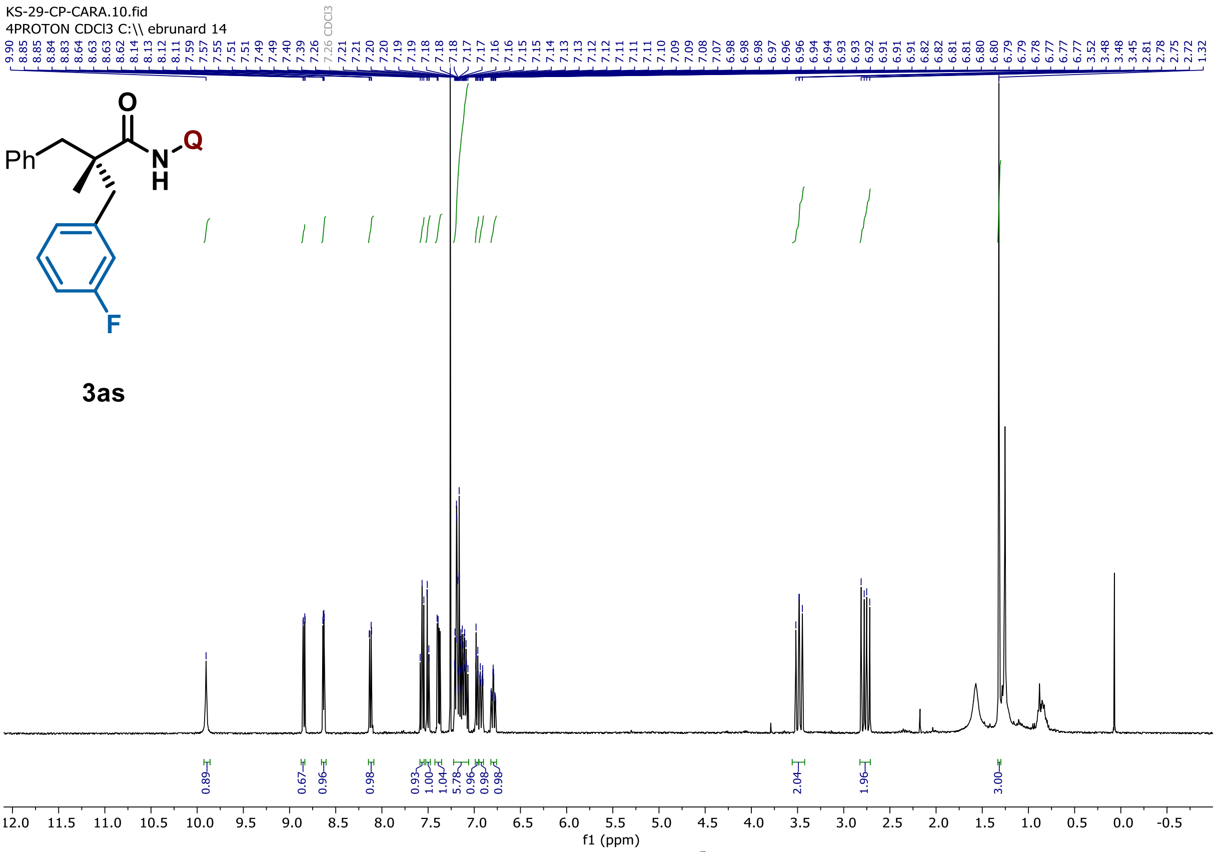

3as
KS-29-CP-CARA.10.fid
4PROTON CDCl3 C:\\ ebrunard 14
f1 (ppm)

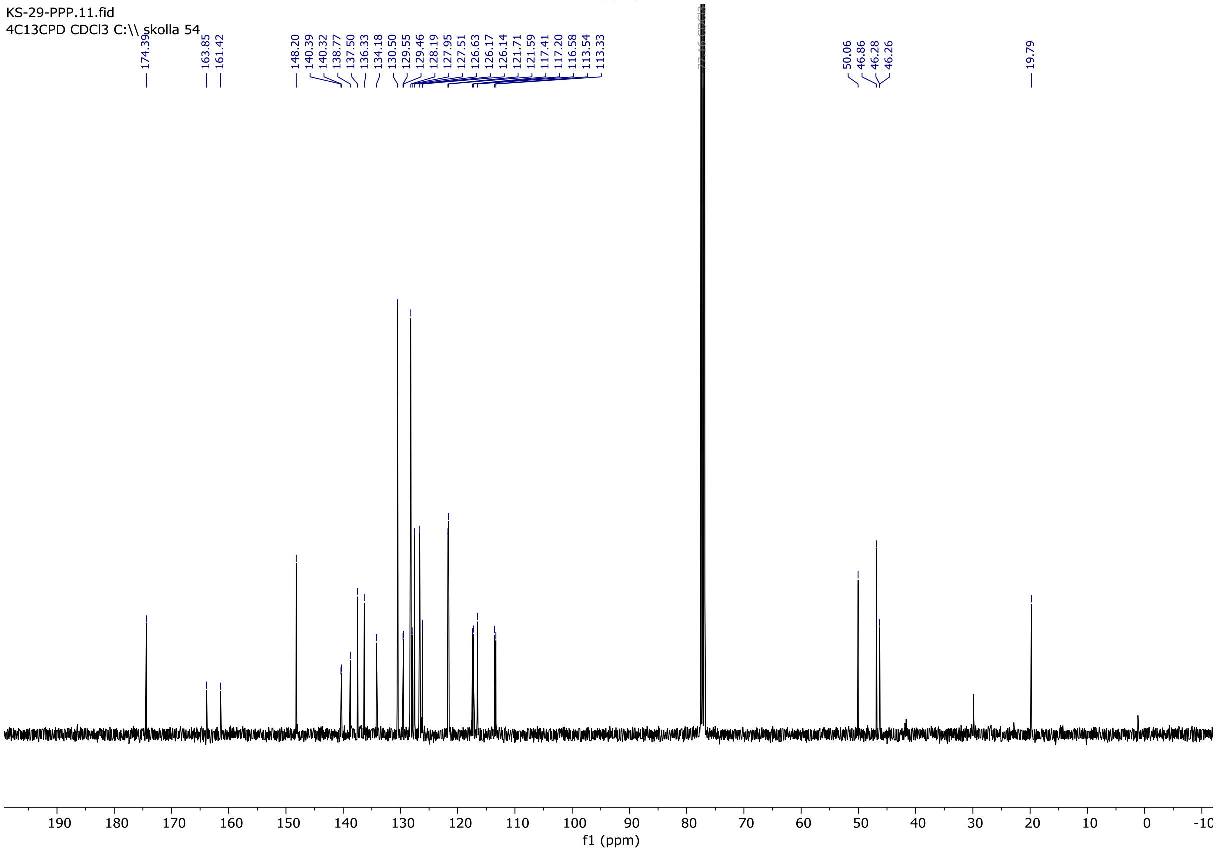
KS-29-PPP.11.fid
4C13CPD CDCl3 C:\\ skolla 54
174.39
163.85
161.42
148.20
140.39
140.32
138.77
137.50
136.33
134.18
130.50
129.55
129.46
128.19
127.95
127.51
126.63
126.17
126.14
121.71
121.59
117.41
117.20
116.58
113.54
113.33
50.06
46.86
46.28
46.26
19.79
f1 (ppm)

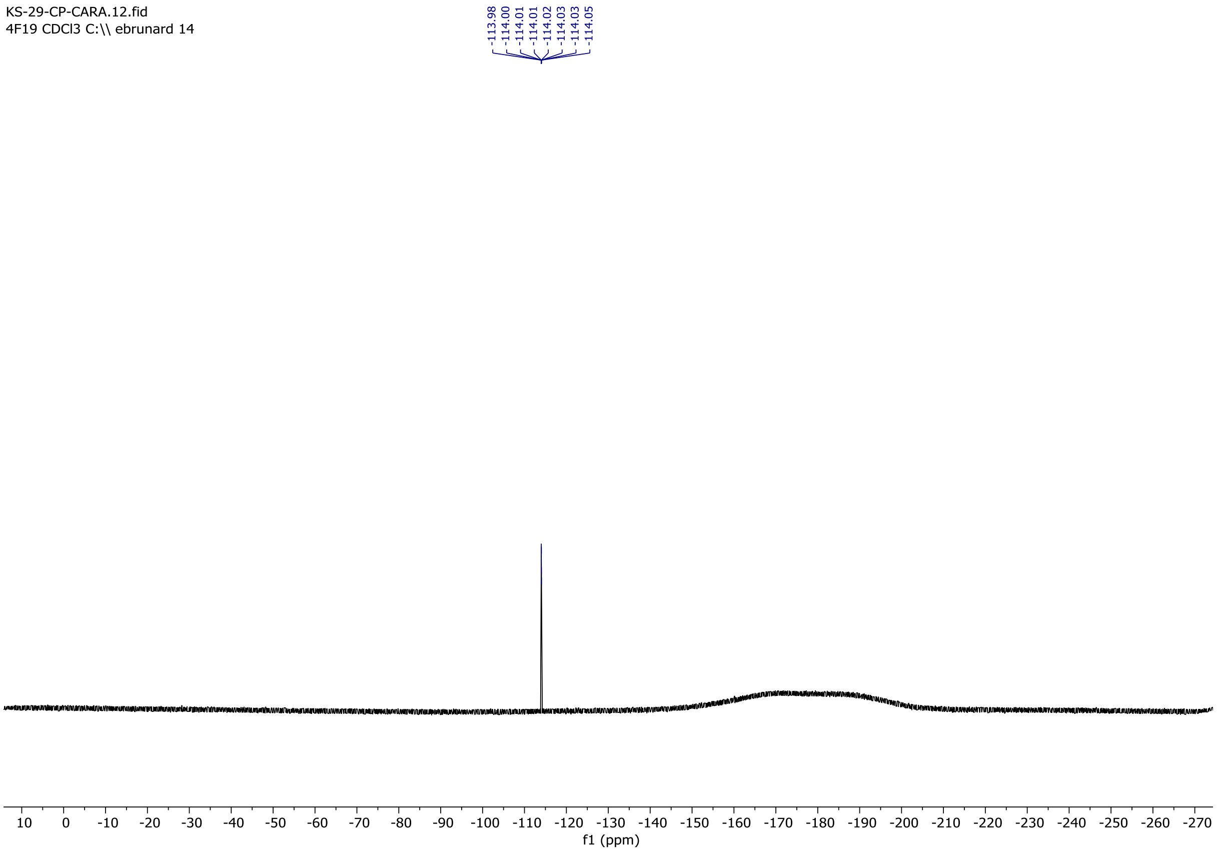
KS-29-CP-CARA.12.fid
4F19 CDCl3 C:\\ ebrunard 14
-113.98
-114.00
-114.01
-114.01
-114.02
-114.03
-114.03
-114.05
10 0 -10 -20 -30 -40 -50 -60 -70 -80 -90 -100 -110 -120 -130 -140 -150 -160 -170 -180 -190 -200 -210 -220 -230 -240 -250 -260 -270
f1 (ppm)

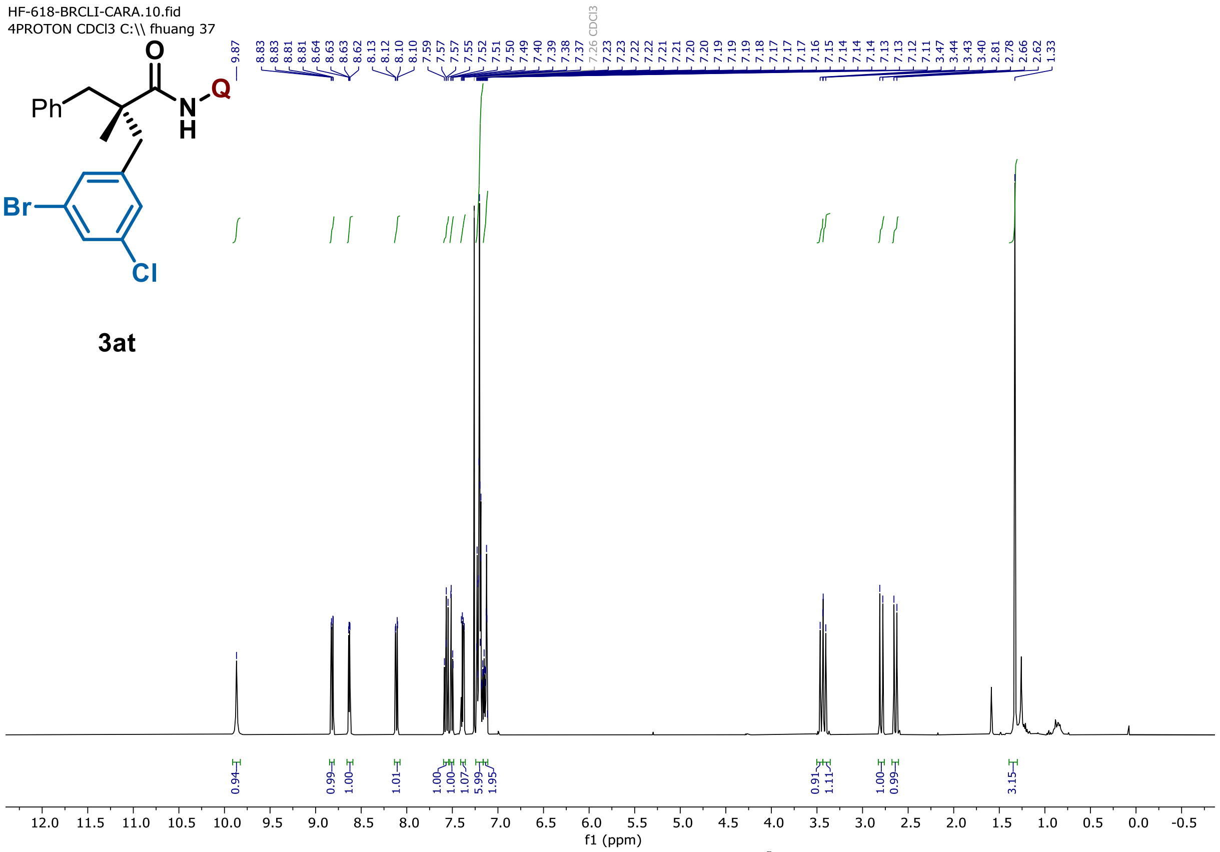
HF-618-BRCLI-CARA.10.fid
4PROTON CDCl3 C:\\ fhuang 37
Ph
O
N
H
Q
Br
Cl
3at
9.87
8.83 8.83 8.81 8.64 8.63 8.63 8.62 8.13 8.12 8.10 8.10 7.59 7.57 7.57 7.55 7.52 7.51 7.50 7.49 7.40 7.39 7.38 7.37
7.26 CDCl3
7.23 7.23 7.22 7.22 7.21 7.21 7.20 7.19 7.19 7.19 7.18 7.17 7.17 7.17 7.16 7.15 7.14 7.14 7.14 7.13 7.13 7.12 7.11 3.47 3.44 3.43 3.40 2.81 2.78 2.66 2.62 1.33
0.94 0.99 1.00 1.01 1.00 1.00 1.07 5.99 1.95 0.91 1.11 1.00 0.99 3.15
12.0 11.5 11.0 10.5 10.0 9.5 9.0 8.5 8.0 7.5 7.0 6.5 6.0 5.5 5.0 4.5 4.0 3.5 3.0 2.5 2.0 1.5 1.0 0.5 0.0 -0.5
f1 (ppm)

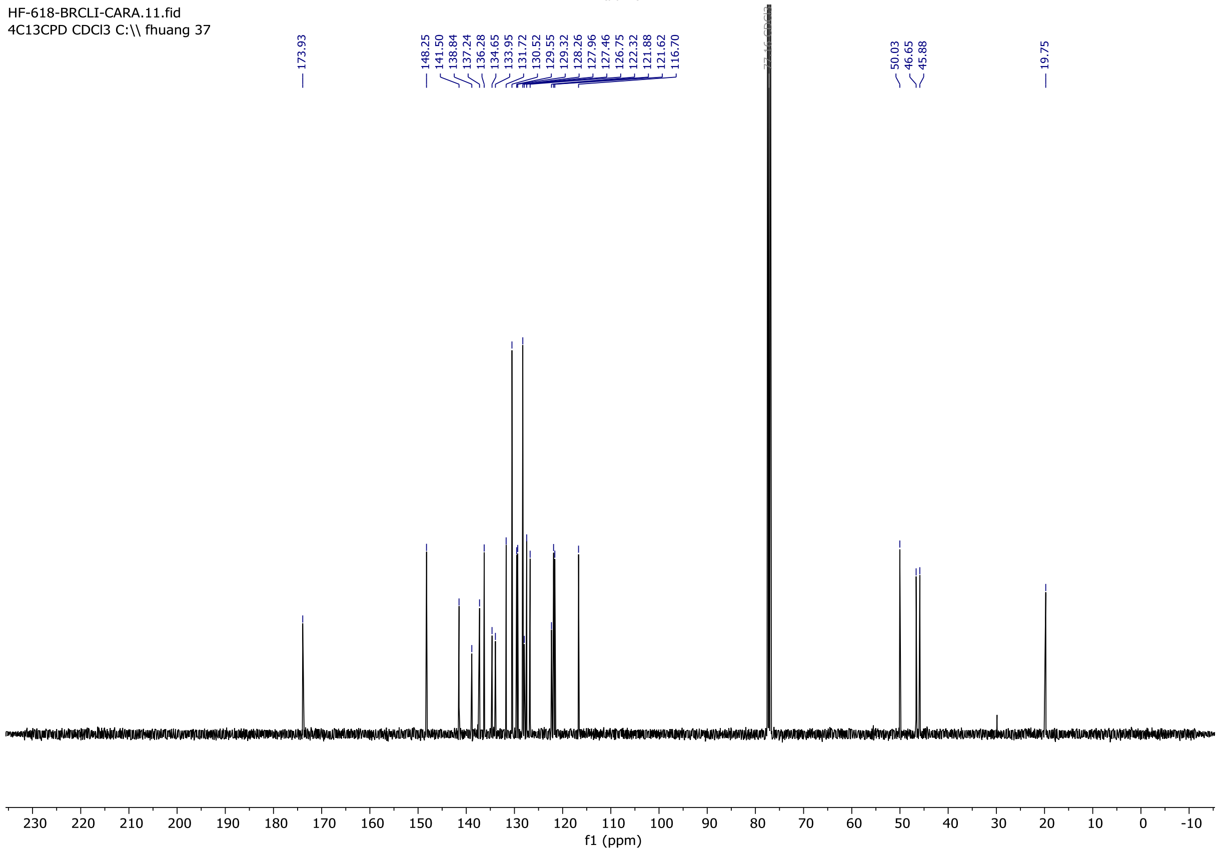
HF-618-BRCLI-CARA.11.fid
4C13CPD CDCl3 C:\\ fhuang 37
173.93
148.25 141.50 138.84 137.24 136.28 134.65 133.95 131.72 130.52 129.55 129.32 128.26 127.96 127.46 126.75 122.32 121.88 121.62 116.70
50.03 46.65 45.88
19.75
230 220 210 200 190 180 170 160 150 140 130 120 110 100 90 80 70 60 50 40 30 20 10 0 -10
f1 (ppm)

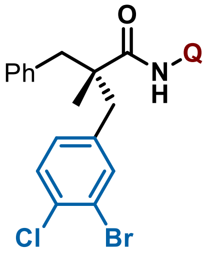

3au

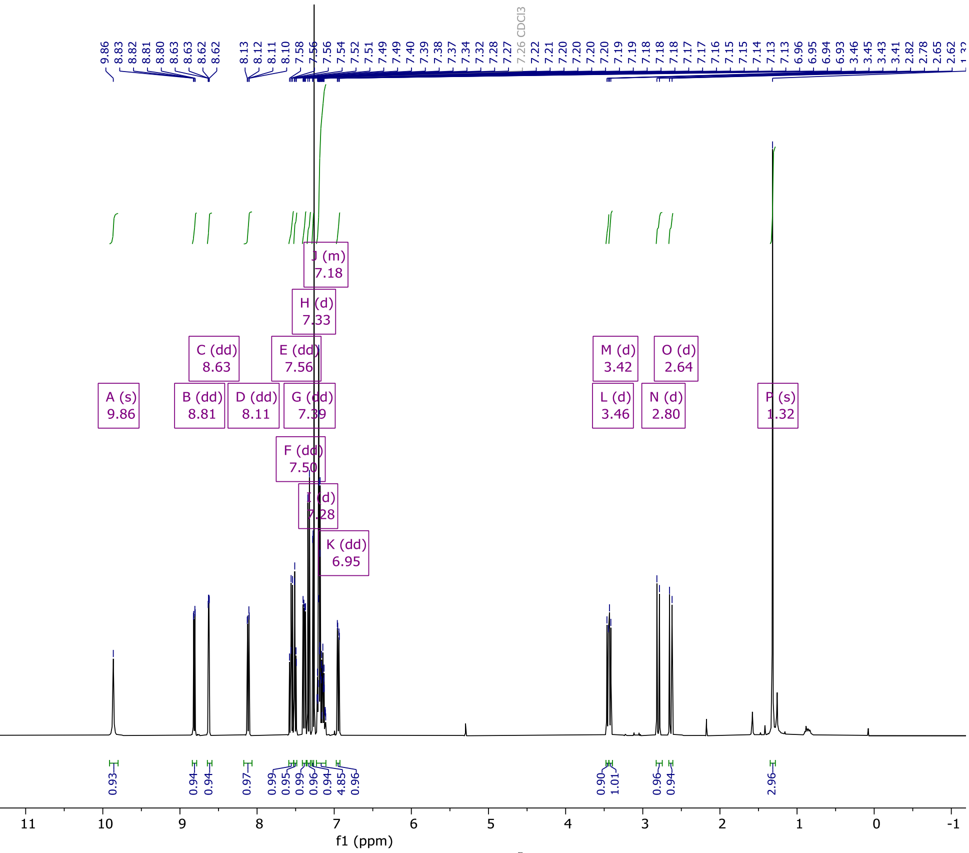

erbr-618f.1cara.10.fid
4PROTON CDCl3 C:\\ ebrunard 4
A (s) 9.86
B (dd) 8.81
C (dd) 8.63
D (dd) 8.11
E (dd) 7.56
F (dd) 7.50
G (dd) 7.39
H (d) 7.33
J (m) 7.18
K (dd) 6.95
L (d) 3.46
M (d) 3.42
N (d) 2.80
O (d) 2.64
P (s) 1.32
f1 (ppm)

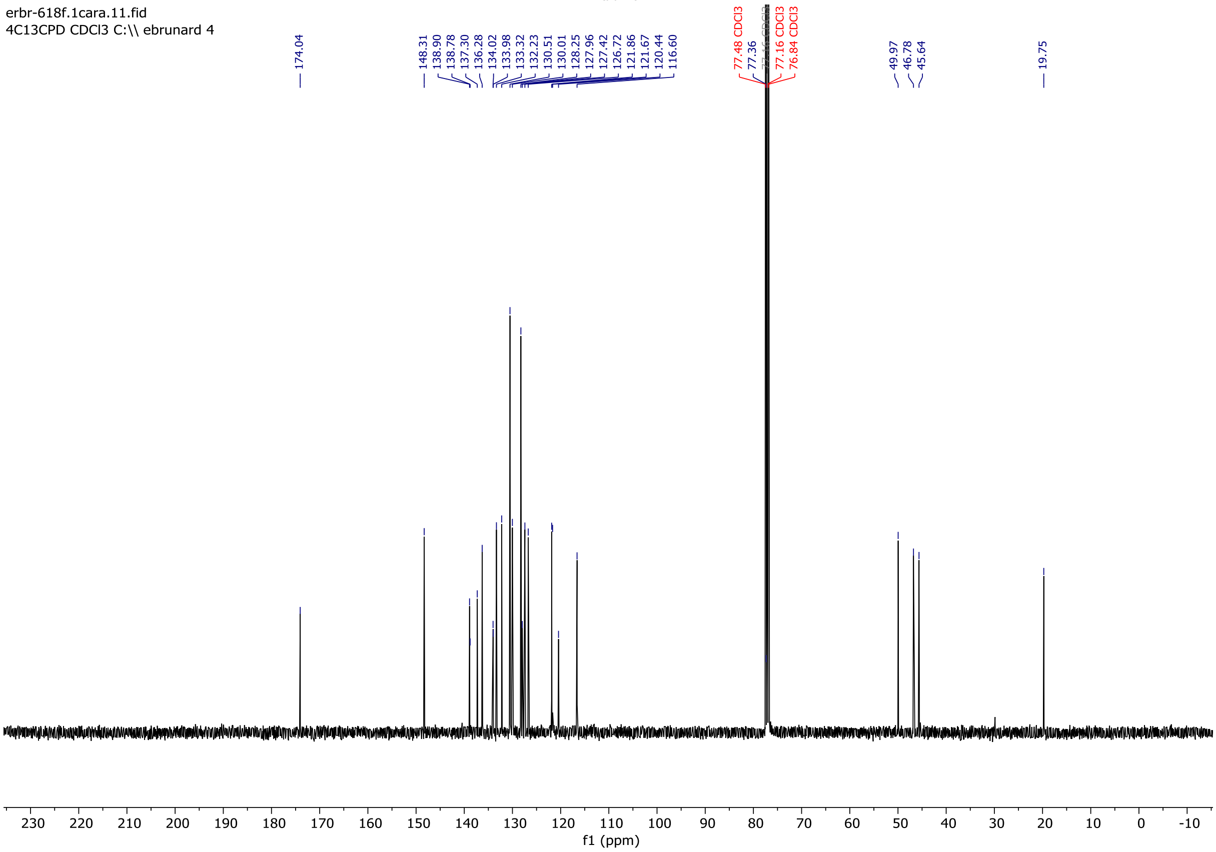

erbr-618f.1cara.11.fid
4C13CPD CDCl3 C:\\ ebrunard 4
174.04
148.31
138.90
138.78
137.30
136.28
134.02
133.98
133.32
132.23
130.51
130.01
128.25
127.96
127.42
126.72
121.86
121.67
120.44
116.60
77.48 CDCl3
77.36
77.16 CDCl3
76.84 CDCl3
49.97
46.78
45.64
19.75
f1 (ppm)

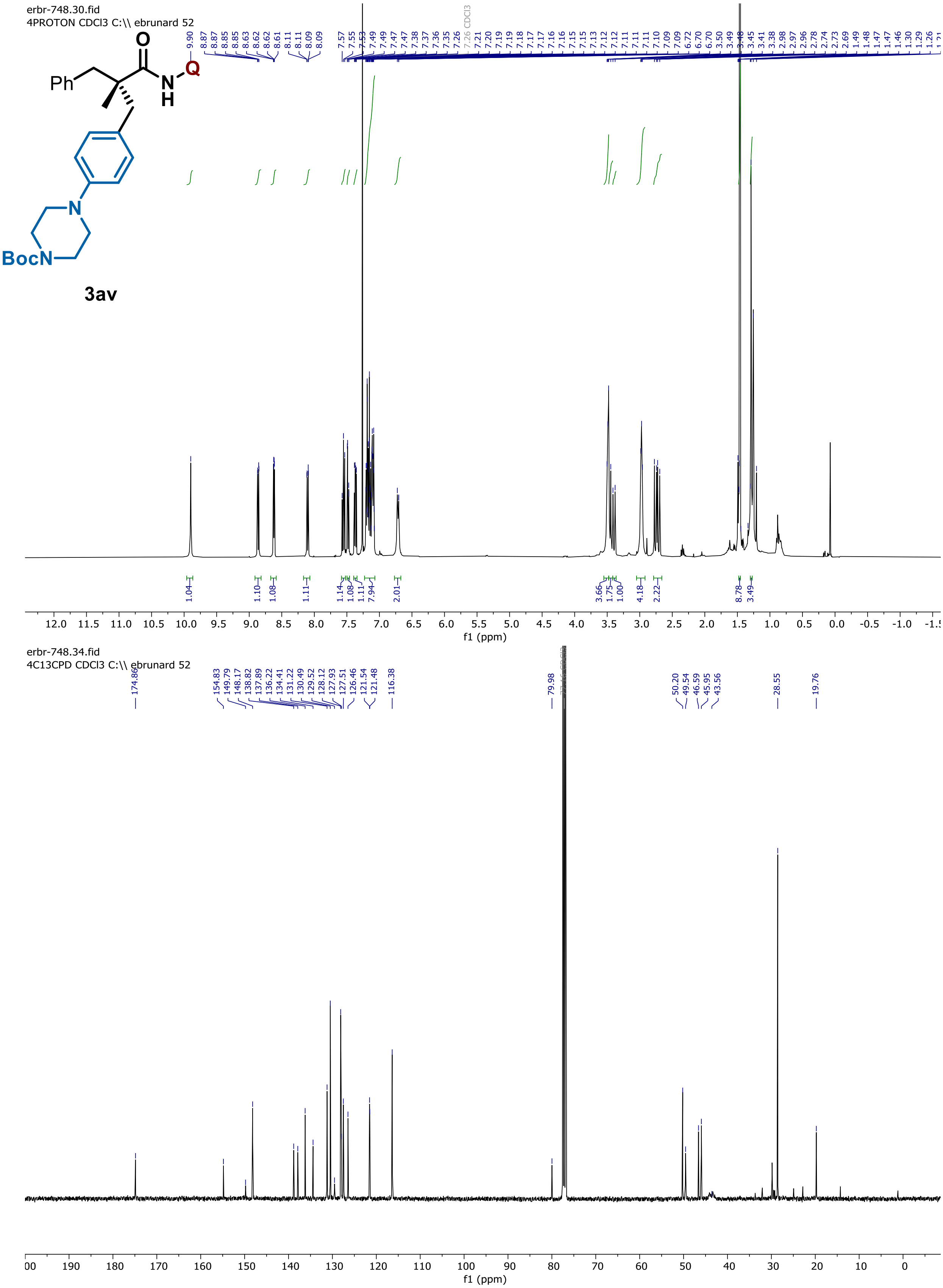
erbr-748.30.fid
4PROTON CDCl3 C:\\ ebrunard 52
9.90
8.87
8.87
8.85
8.85
8.63
8.62
8.62
8.61
8.11
8.11
8.09
8.09
7.57
7.55
7.53
7.49
7.49
7.47
7.47
7.38
7.37
7.36
7.35
7.26 CDCl3
7.21
7.20
7.19
7.19
7.18
7.17
7.17
7.16
7.16
7.15
7.15
7.13
7.12
7.12
7.11
7.11
7.11
7.10
7.09
7.09
6.72
6.72
6.70
6.70
3.50
3.49
3.48
3.45
3.41
3.38
2.98
2.97
2.96
2.78
2.74
2.73
2.69
1.49
1.48
1.47
1.47
1.46
1.30
1.29
1.26
Ph
O
N
Q
H
N
BocN
3av
1.04
1.10
1.08
1.11
1.14
1.08
1.11
7.94
2.01
3.66
1.75
1.00
4.18
2.22
8.78
3.49
f1 (ppm)
erbr-748.34.fid
4C13CPD CDCl3 C:\\ ebrunard 52
174.86
154.83
149.79
148.17
138.82
137.89
136.22
134.41
131.22
130.49
129.52
128.12
127.93
127.51
126.46
121.54
121.48
116.38
79.98
50.20
49.54
46.59
45.95
43.56
28.55
19.76
f1 (ppm)

HF-551-OO-ME-cara.10.fid
4PROTON CDCl3 C:\\ fhuang 8

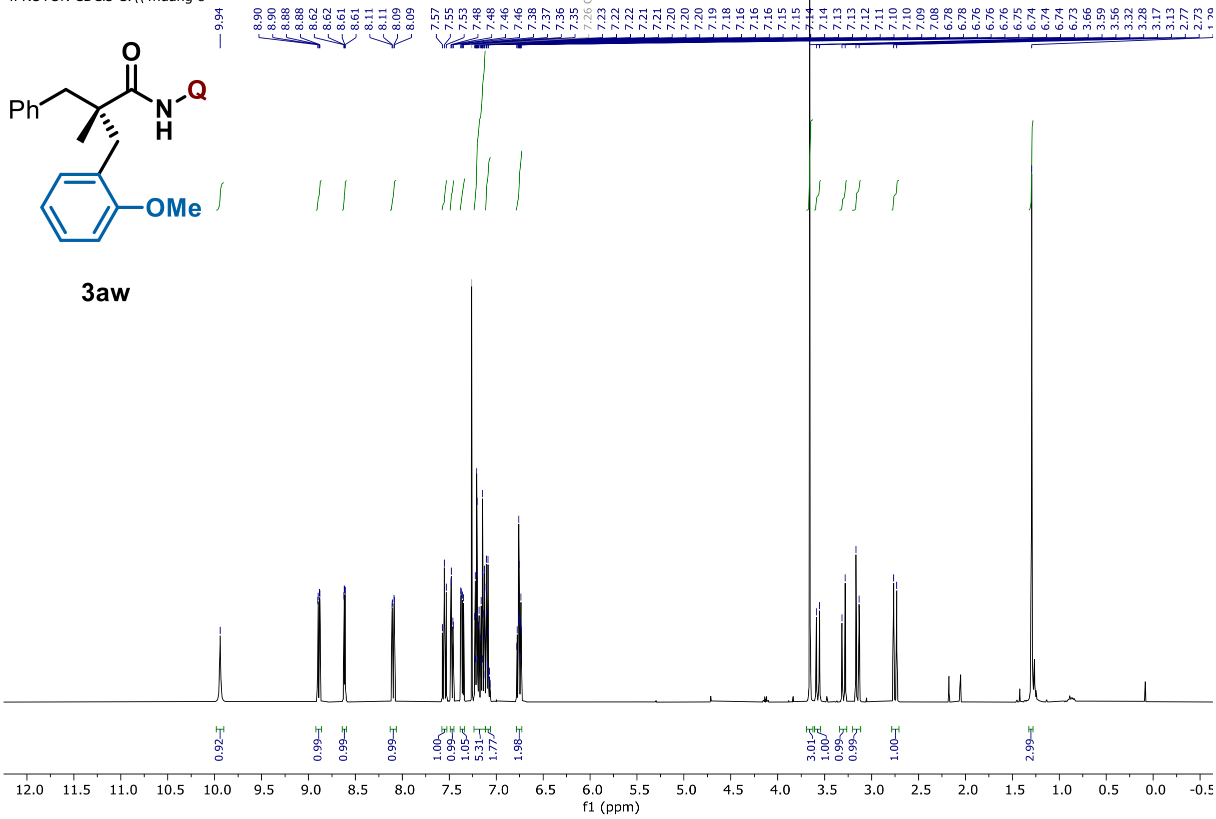

HF-551-OO-ME-cara.11.fid
4C13CPD CDCl3 C:\\ fhuang 8

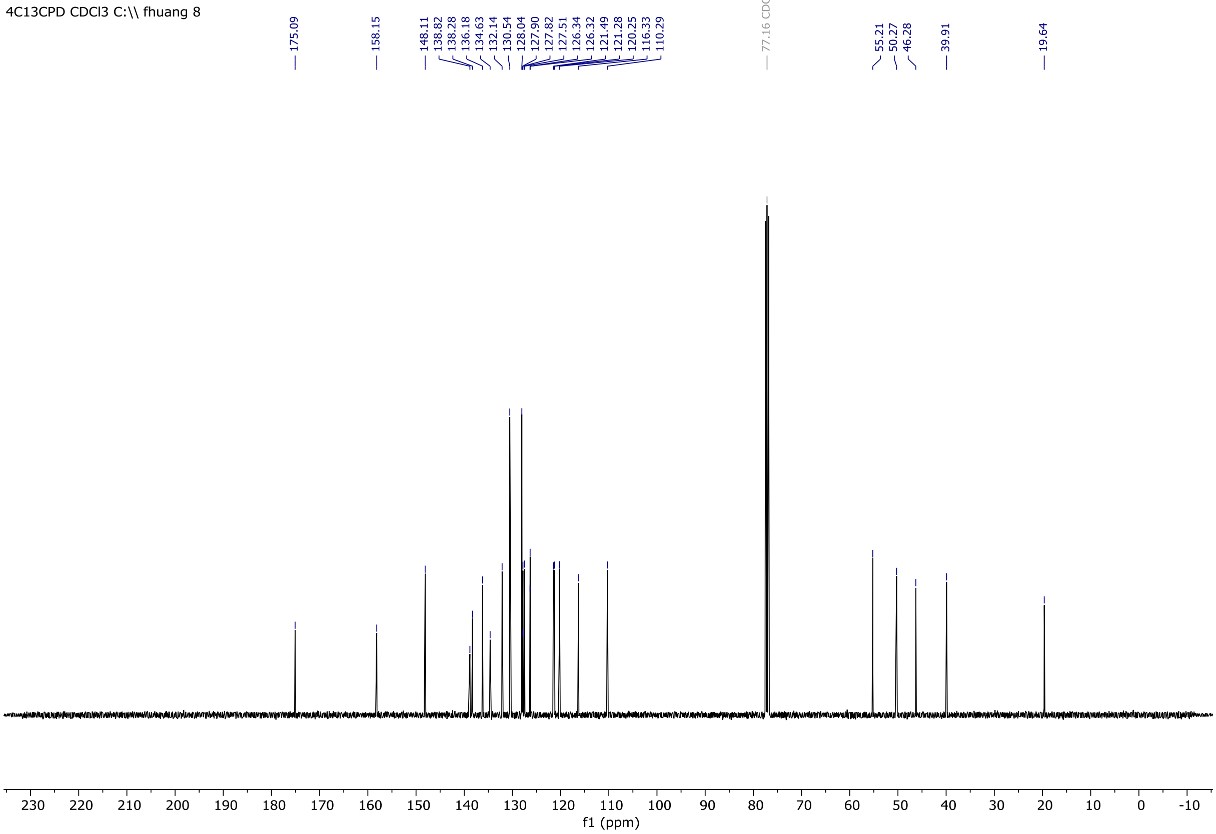

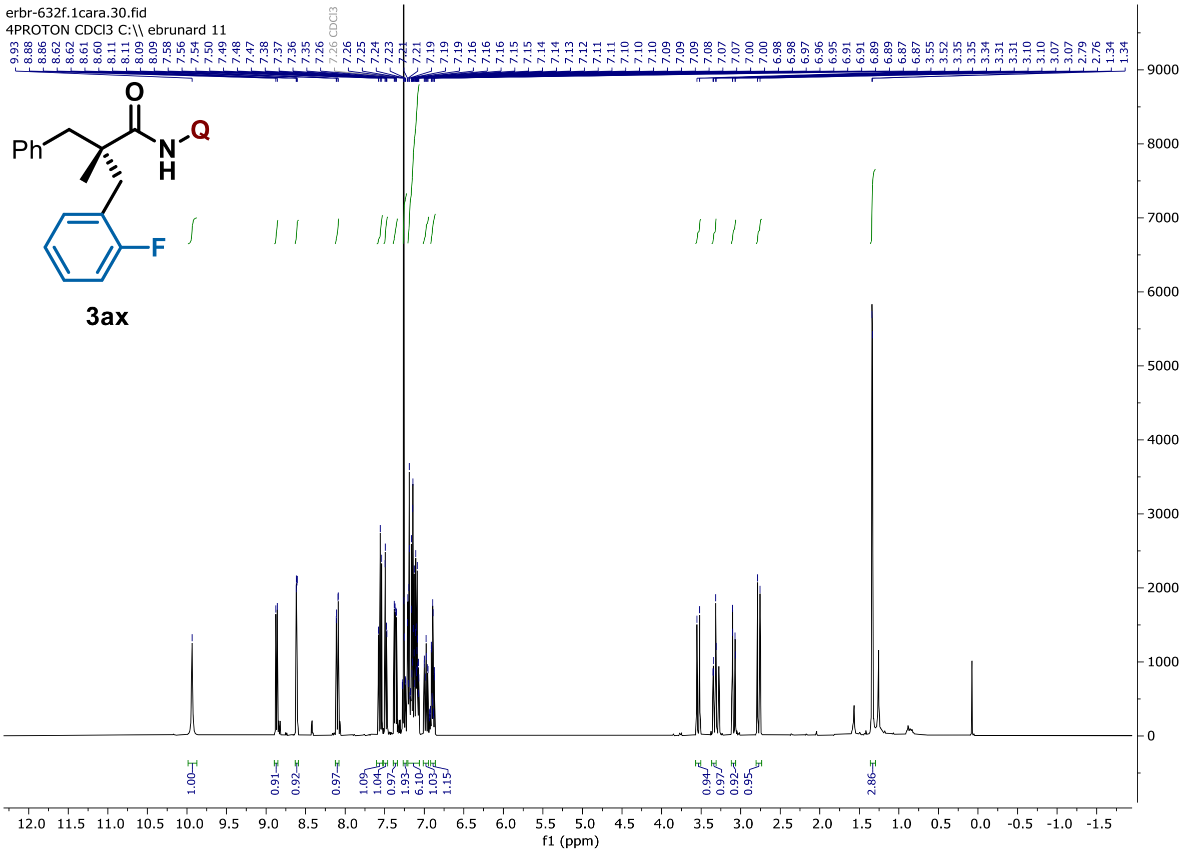
erbr-632f.1cara.30.fid
4PROTON CDCl3 C:\\ ebrunard 11
9.93 8.88 8.86 8.62 8.61 8.60 8.11 8.11 8.09 8.09 7.58 7.56 7.54 7.50 7.49 7.48 7.47 7.38 7.37 7.36 7.35 7.26 7.26 CDCl3 7.26 7.25 7.24 7.23 7.21 7.21 7.19 7.19 7.16 7.16 7.15 7.15 7.14 7.14 7.13 7.12 7.11 7.11 7.10 7.10 7.09 7.09 7.08 7.07 7.07 7.00 7.00 6.98 6.98 6.97 6.96 6.95 6.91 6.91 6.89 6.89 6.87 6.87 3.55 3.52 3.35 3.35 3.34 3.31 3.31 3.10 3.10 3.07 3.07 2.79 2.76 1.34 1.34
O
Ph
N
H
Q
F
3ax
1.00 0.91 0.92 0.97 1.09 1.04 0.97 1.93 6.10 1.03 1.15 0.94 0.97 0.92 0.95 2.86
12.0 11.5 11.0 10.5 10.0 9.5 9.0 8.5 8.0 7.5 7.0 6.5 6.0 5.5 5.0 4.5 4.0 3.5 3.0 2.5 2.0 1.5 1.0 0.5 0.0 -0.5 -1.0 -1.5
f1 (ppm)
9000 8000 7000 6000 5000 4000 3000 2000 1000 0

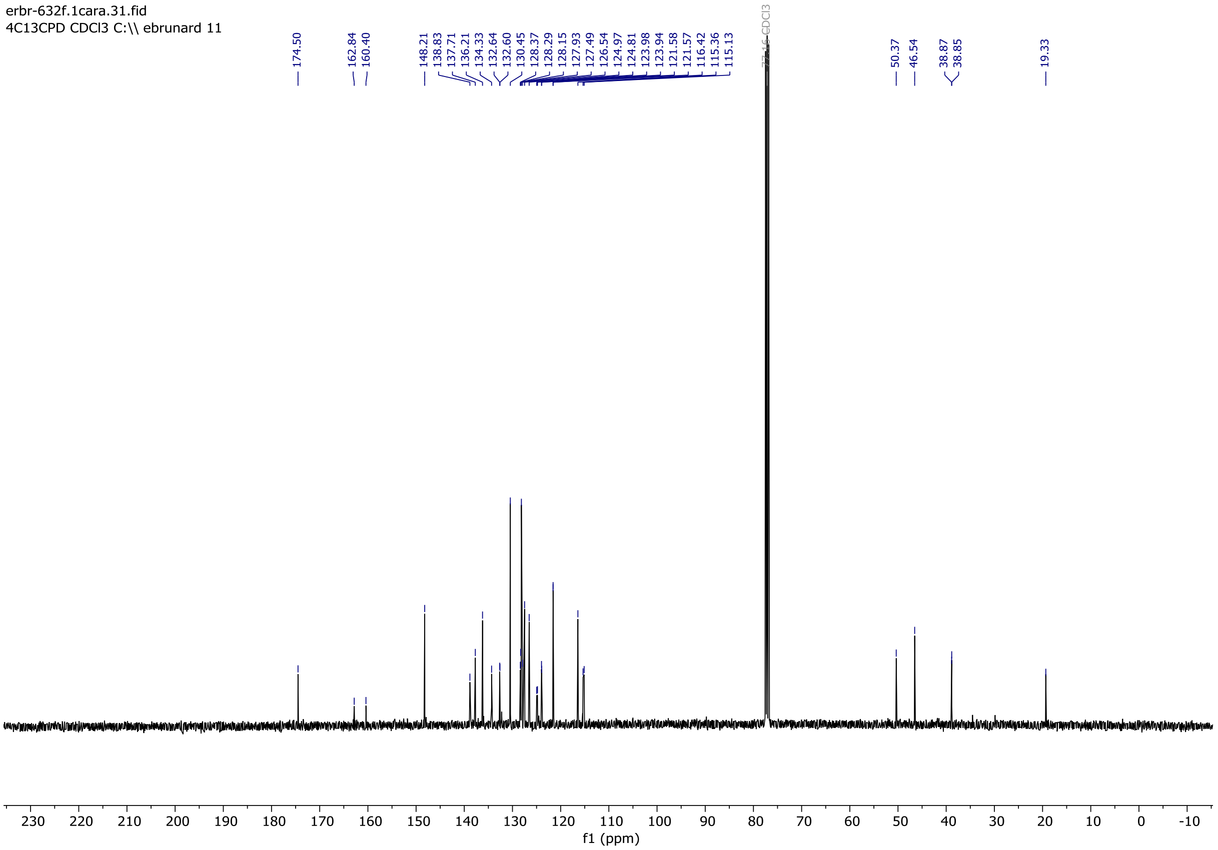
erbr-632f.1cara.31.fid
4C13CPD CDCl3 C:\\ ebrunard 11
174.50 162.84 160.40 148.21 138.83 137.71 136.21 134.33 132.64 132.60 130.45 128.37 128.29 128.15 127.93 127.49 126.54 124.97 124.81 123.98 123.94 121.58 121.57 116.42 115.36 115.13 77.16 CDCl3 50.37 46.54 38.87 38.85 19.33
230 220 210 200 190 180 170 160 150 140 130 120 110 100 90 80 70 60 50 40 30 20 10 0 -10
f1 (ppm)

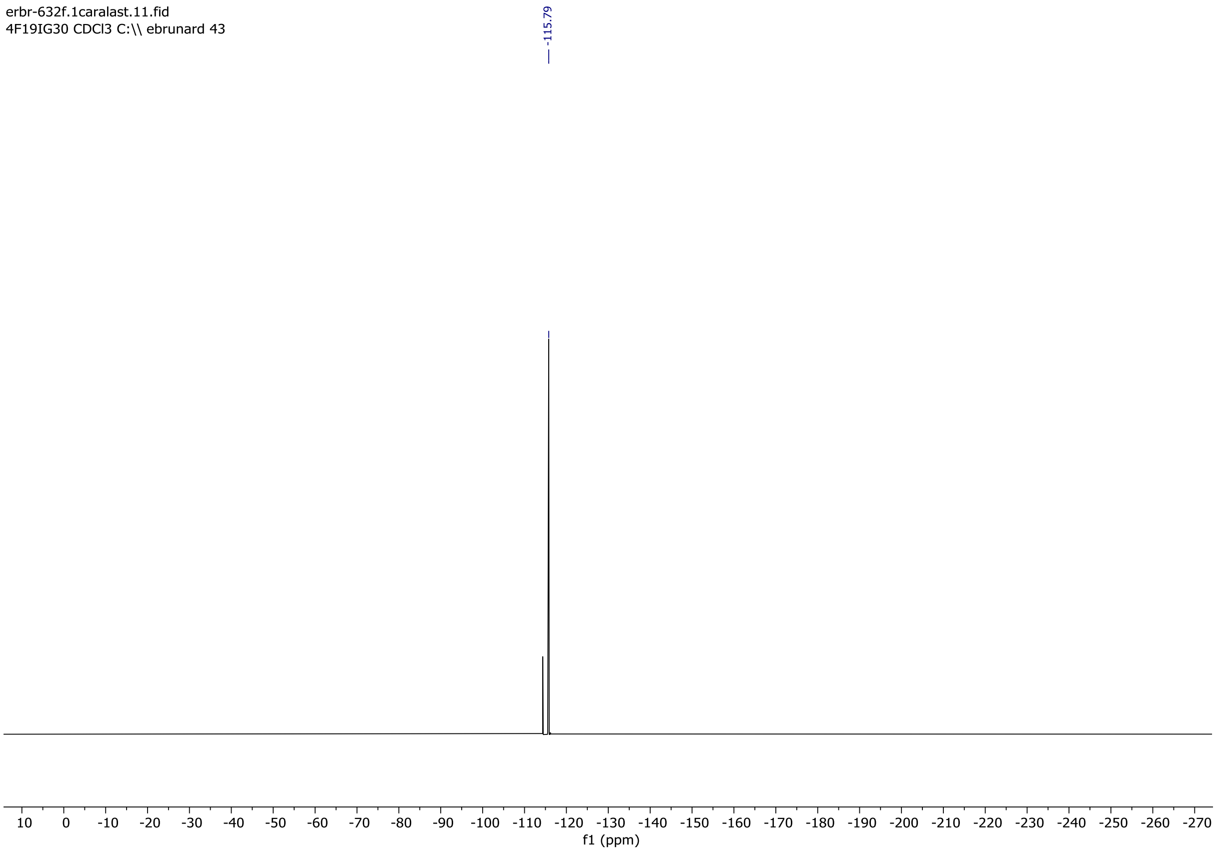
erbr-632f.1caralast.11.fid
4F19IG30 CDCl3 C:\\ ebrunard 43
-115.79
10 0 -10 -20 -30 -40 -50 -60 -70 -80 -90 -100 -110 -120 -130 -140 -150 -160 -170 -180 -190 -200 -210 -220 -230 -240 -250 -260 -270
f1 (ppm)

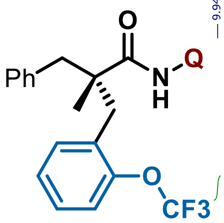

3ay

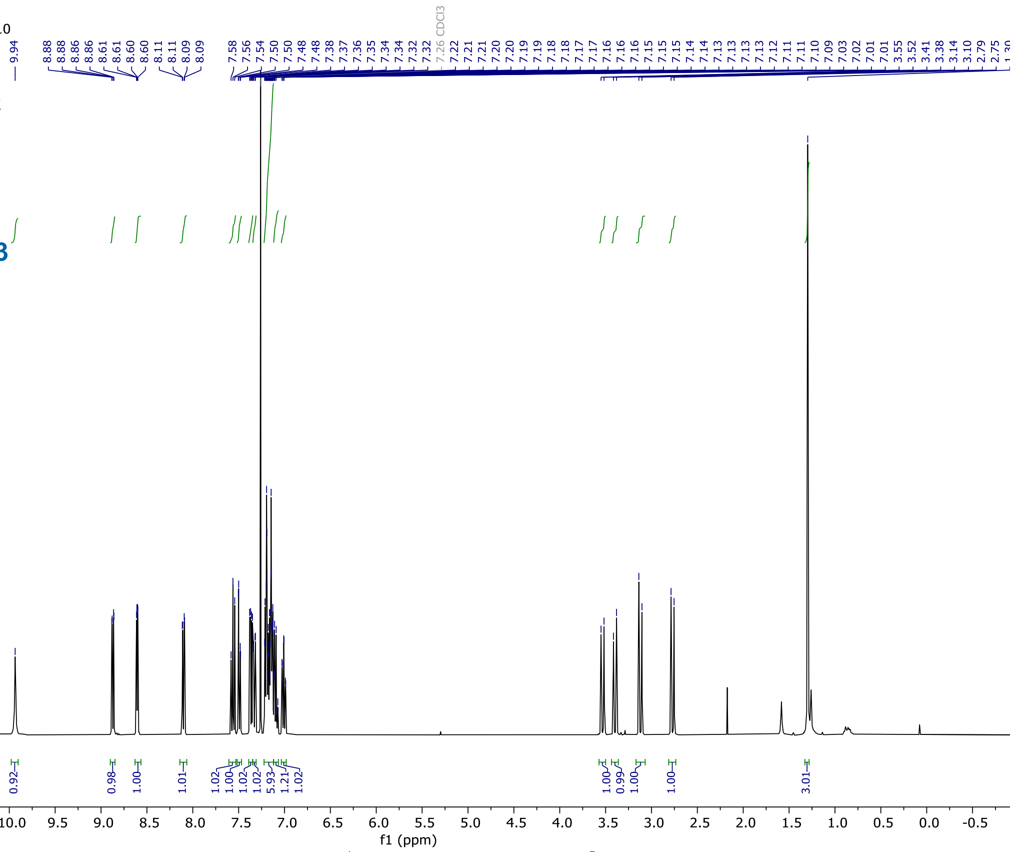
HF-563-O-OCF3-cara.10.fid
4PROTON CDCl3 C:\\ fhuang 10
9.94
8.88 8.88 8.86 8.86 8.61 8.60 8.60 8.11 8.11 8.09 8.09
7.58 7.56 7.54 7.50 7.50 7.48 7.48 7.38 7.37 7.36 7.35 7.34 7.34 7.32 7.32
7.26 CDCl3
7.22 7.21 7.21 7.20 7.20 7.19 7.19 7.18 7.18 7.17 7.17 7.16 7.16 7.15 7.15 7.14 7.14 7.13 7.13 7.13 7.12 7.11 7.11 7.10 7.09 7.03 7.02 7.01 7.01
3.55 3.52 3.41 3.38 3.14 3.10 2.79 2.75
0.92 0.98 1.00 1.01 1.02 1.00 1.02 1.02 5.93 1.21 1.02 1.00 0.99 1.00 1.00 3.01
12.0 11.5 11.0 10.5 10.0 9.5 9.0 8.5 8.0 7.5 7.0 6.5 6.0 5.5 5.0 4.5 4.0 3.5 3.0 2.5 2.0 1.5 1.0 0.5 0.0 -0.5
f1 (ppm)

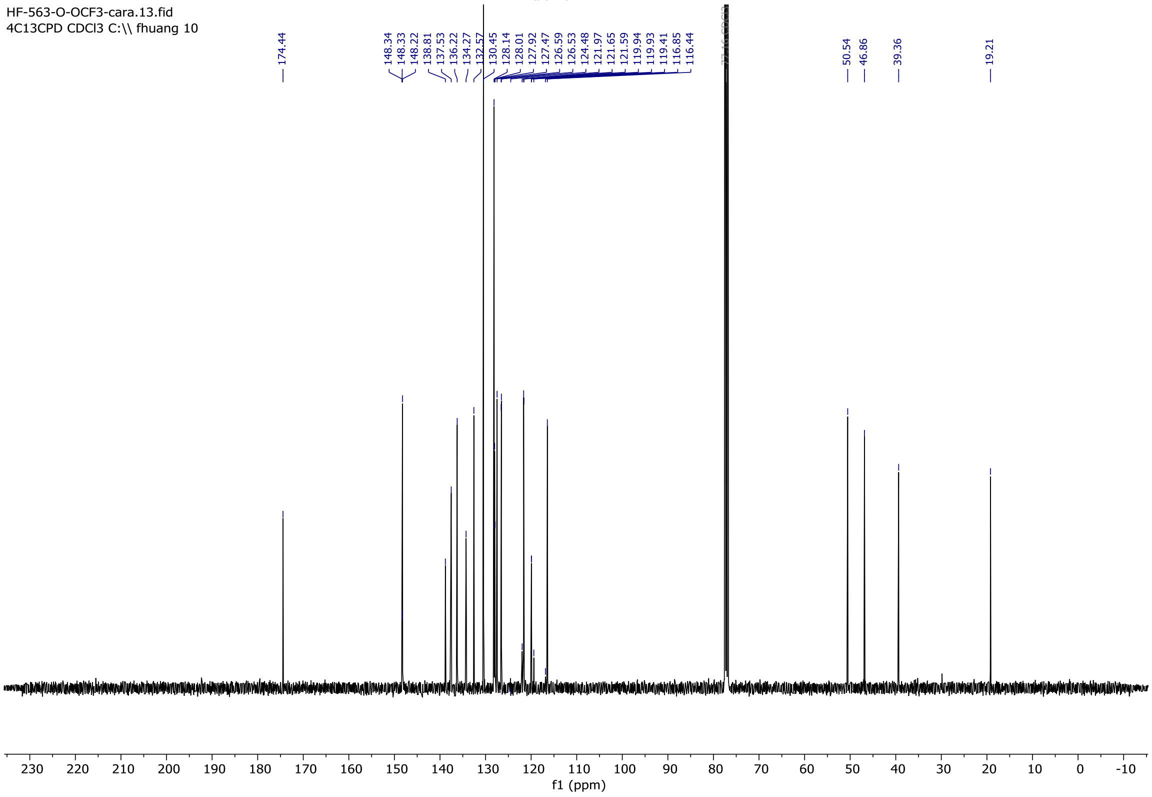
HF-563-O-OCF3-cara.13.fid
4C13CPD CDCl3 C:\\ fhuang 10
174.44
148.34 148.33 148.22 138.81 137.53 136.22 134.27 132.57 130.45 128.14 128.01 127.92 127.47 126.59 126.53 124.48 121.97 121.65 121.59 119.94 119.93 119.41 116.85 116.44
50.54 46.86 39.36 19.21
230 220 210 200 190 180 170 160 150 140 130 120 110 100 90 80 70 60 50 40 30 20 10 0 -10
f1 (ppm)

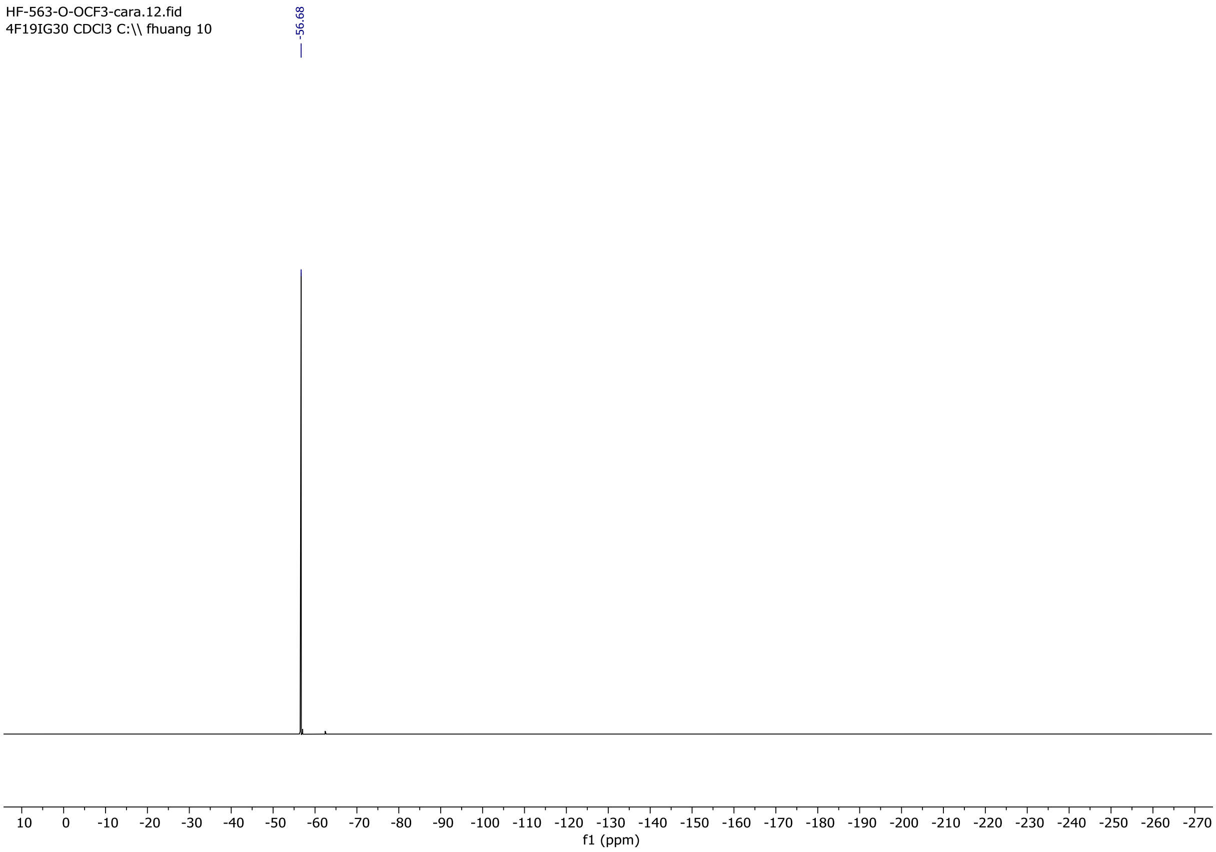
HF-563-O-OCF3-cara.12.fid
4F19IG30 CDCl3 C:\\ fhuang 10
-56.68
10 0 -10 -20 -30 -40 -50 -60 -70 -80 -90 -100 -110 -120 -130 -140 -150 -160 -170 -180 -190 -200 -210 -220 -230 -240 -250 -260 -270
f1 (ppm)

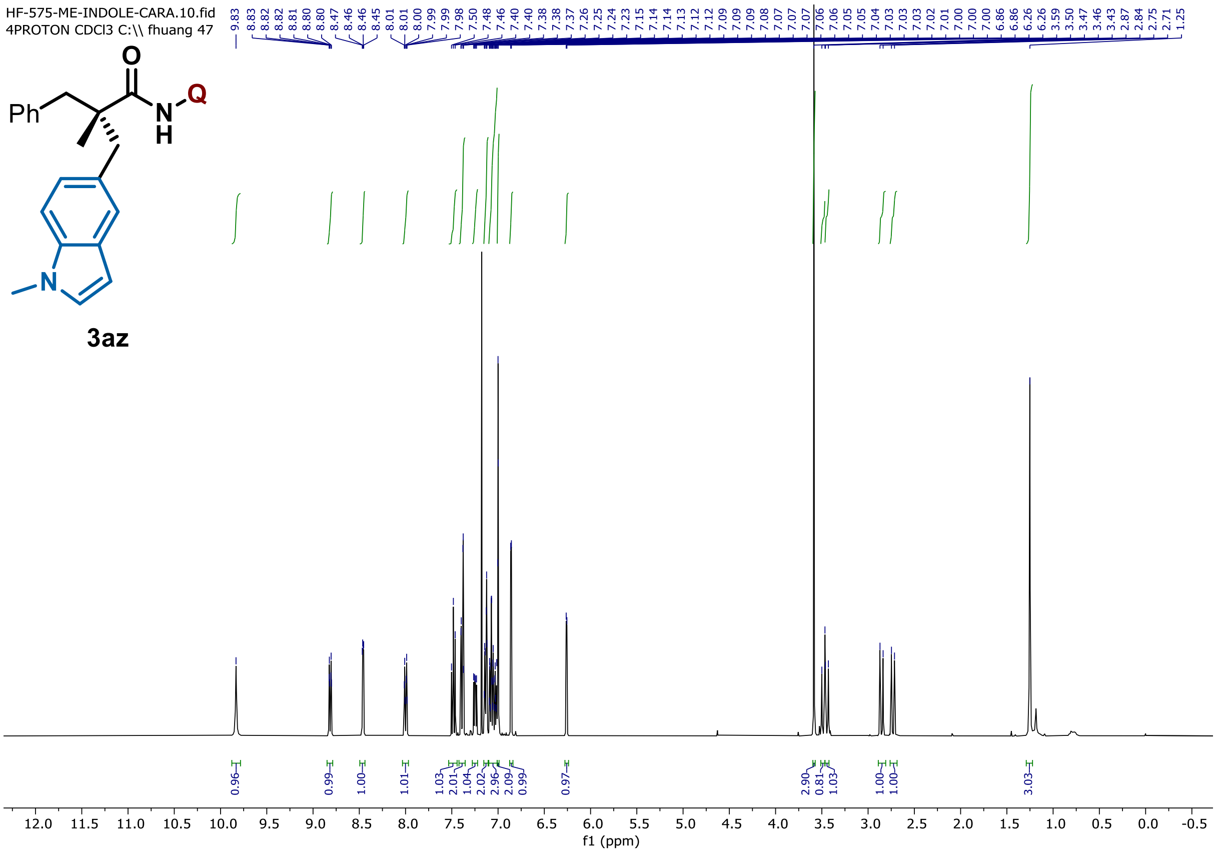
HF-575-ME-INDOLE-CARA.10.fid
4PROTON CDCl3 C:\\ fhuang 47
9.83 8.83 8.82 8.81 8.80 8.80 8.47 8.46 8.46 8.45 8.01 8.01 8.00 7.99 7.99 7.98 7.50 7.48 7.46 7.40 7.40 7.38 7.38 7.37 7.26 7.25 7.24 7.23 7.15 7.14 7.13 7.12 7.12 7.09 7.09 7.09 7.08 7.07 7.07 7.07 7.07 7.06 7.06 7.05 7.05 7.04 7.03 7.03 7.03 7.02 7.01 7.00 7.00 7.00 6.86 6.86 6.26 6.26 3.59 3.50 3.47 3.46 3.43 2.87 2.84 2.75 2.71 1.25
Ph
O
N
Q
H
N
3az
0.96 0.99 1.00 1.01 1.03 2.01 1.04 2.02 2.96 2.09 0.99 0.97 2.90 0.81 1.03 1.00 1.00 3.03
12.0 11.5 11.0 10.5 10.0 9.5 9.0 8.5 8.0 7.5 7.0 6.5 6.0 5.5 5.0 4.5 4.0 3.5 3.0 2.5 2.0 1.5 1.0 0.5 0.0 -0.5
f1 (ppm)

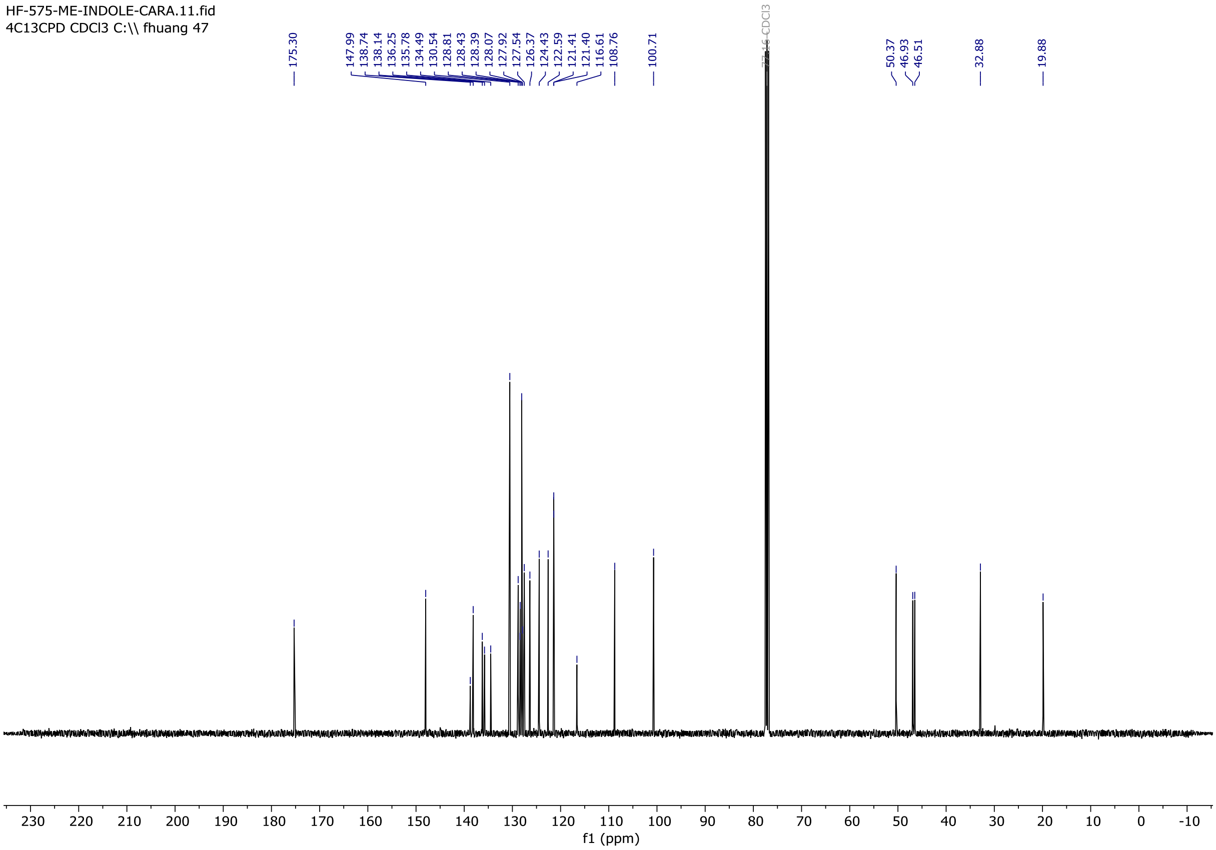
HF-575-ME-INDOLE-CARA.11.fid
4C13CPD CDCl3 C:\\ fhuang 47
175.30
147.99 138.74 138.14 136.25 135.78 134.49 130.54 128.81 128.43 128.39 128.07 127.92 127.54 126.37 124.43 122.59 121.41 121.40 116.61 108.76
100.71
77.16 CDCl3
50.37 46.93 46.51
32.88
19.88
230 220 210 200 190 180 170 160 150 140 130 120 110 100 90 80 70 60 50 40 30 20 10 0 -10
f1 (ppm)

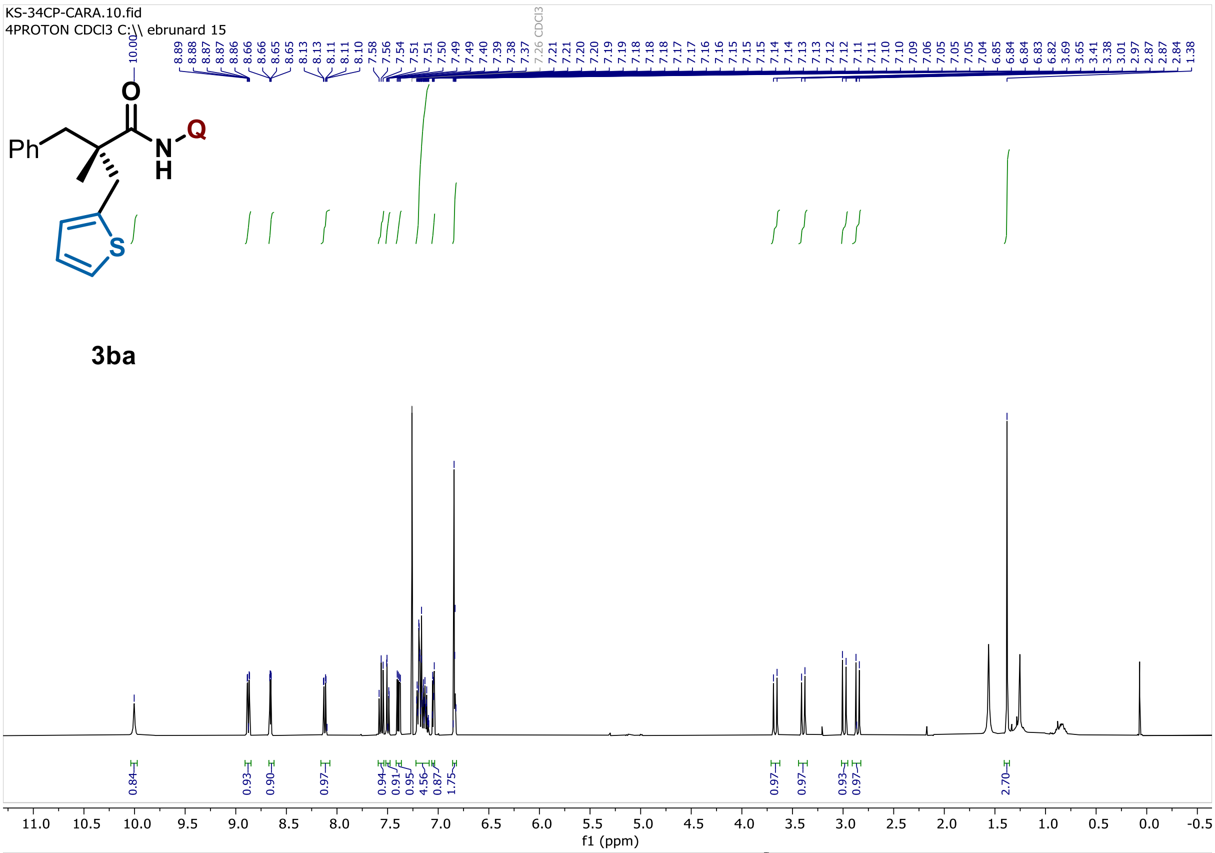

KS-34CP-CARA.10.fid
4PROTON CDCl3 C:\\ ebrunard 15
10.00
8.89 8.88 8.87 8.87 8.86 8.66 8.66 8.65 8.65 8.13 8.13 8.11 8.11 8.10 7.58 7.56 7.54 7.51 7.51 7.50 7.49 7.40 7.39 7.38 7.37 7.26 CDCl3 7.21 7.21 7.20 7.20 7.19 7.19 7.18 7.18 7.18 7.17 7.17 7.16 7.16 7.15 7.15 7.15 7.15 7.14 7.14 7.13 7.13 7.12 7.12 7.11 7.11 7.10 7.10 7.09 7.06 7.05 7.05 7.05 7.04 6.85 6.84 6.84 6.83 6.82 3.69 3.65 3.41 3.38 3.01 2.97 2.87 2.84 1.38
Ph
O
N
H
Q
S
3ba
0.84 0.93 0.90 0.97 0.94 0.91 0.95 4.56 0.87 1.75 0.97 0.97 0.93 0.97 2.70
11.0 10.5 10.0 9.5 9.0 8.5 8.0 7.5 7.0 6.5 6.0 5.5 5.0 4.5 4.0 3.5 3.0 2.5 2.0 1.5 1.0 0.5 0.0 -0.5
f1 (ppm)

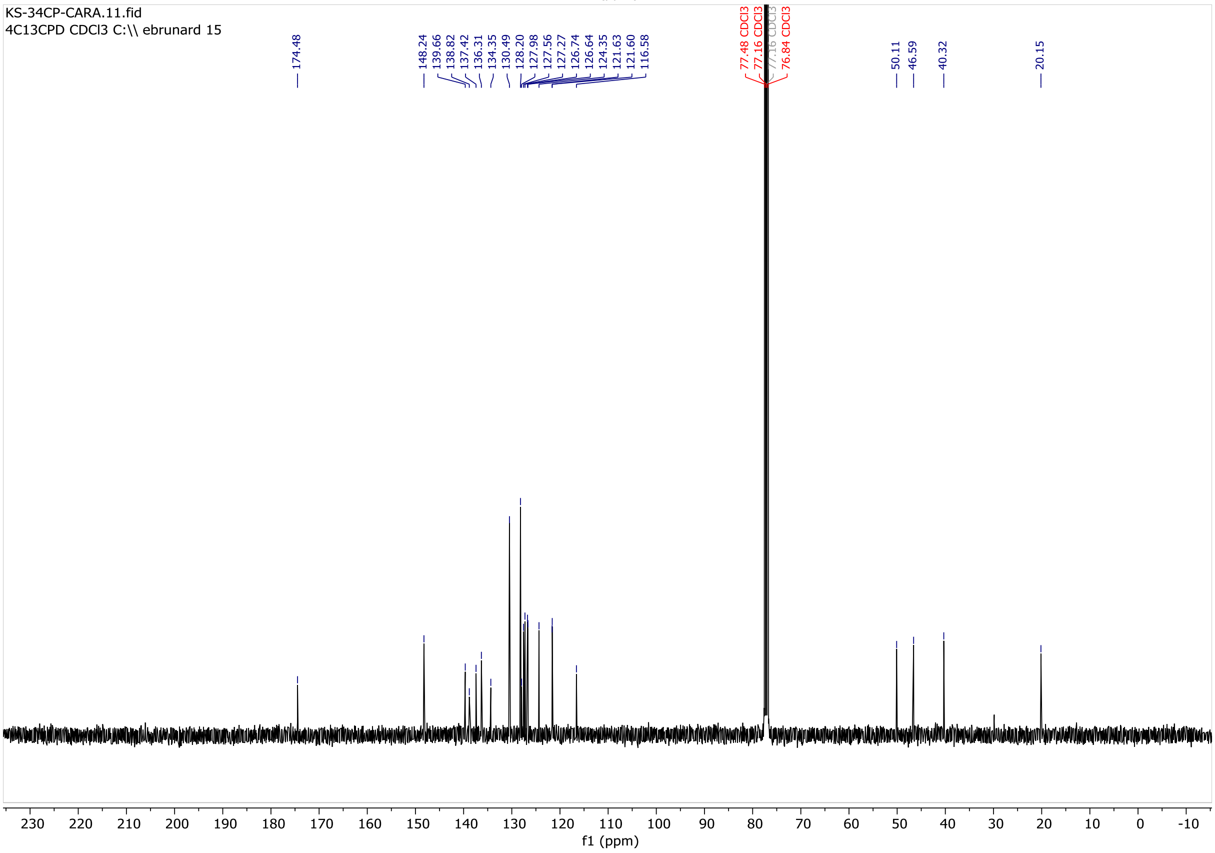

KS-34CP-CARA.11.fid
4C13CPD CDCl3 C:\\ ebrunard 15
174.48
148.24 139.66 138.82 137.42 136.31 134.35 130.49 128.20 127.98 127.56 127.27 126.74 126.64 124.35 121.63 121.60 116.58
77.48 CDCl3 77.16 CDCl3 77.16 CDCl3 76.84 CDCl3
50.11 46.59 40.32 20.15
230 220 210 200 190 180 170 160 150 140 130 120 110 100 90 80 70 60 50 40 30 20 10 0 -10
f1 (ppm)

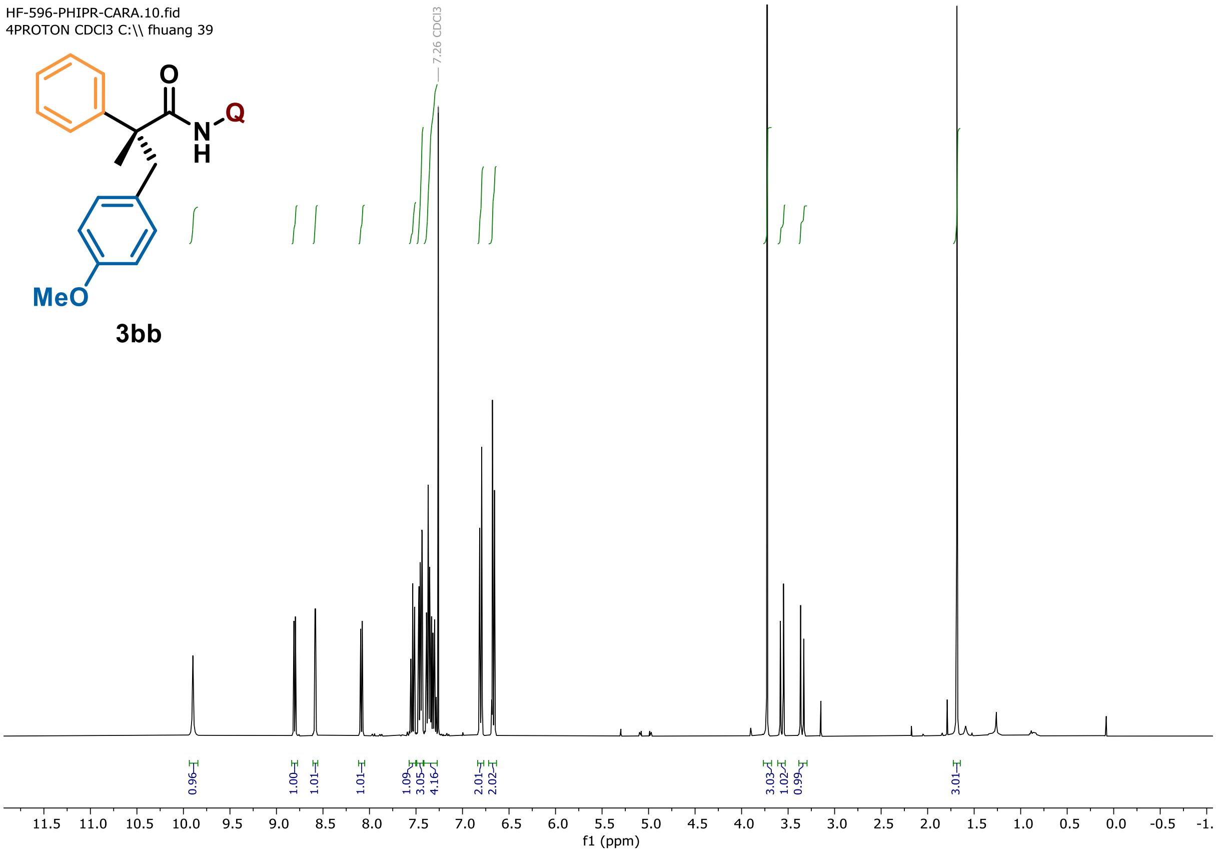
HF-596-PHIPR-CARA.10.fid
4PROTON CDCl3 C:\\ fhuang 39
O
N
H
Q
MeO
3bb
7.26 CDCl3
0.96
1.00
1.01
1.01
1.09
3.05
4.16
2.01
2.02
3.03
1.02
0.99
3.01
11.5 11.0 10.5 10.0 9.5 9.0 8.5 8.0 7.5 7.0 6.5 6.0 5.5 5.0 4.5 4.0 3.5 3.0 2.5 2.0 1.5 1.0 0.5 0.0 -0.5 -1.
f1 (ppm)

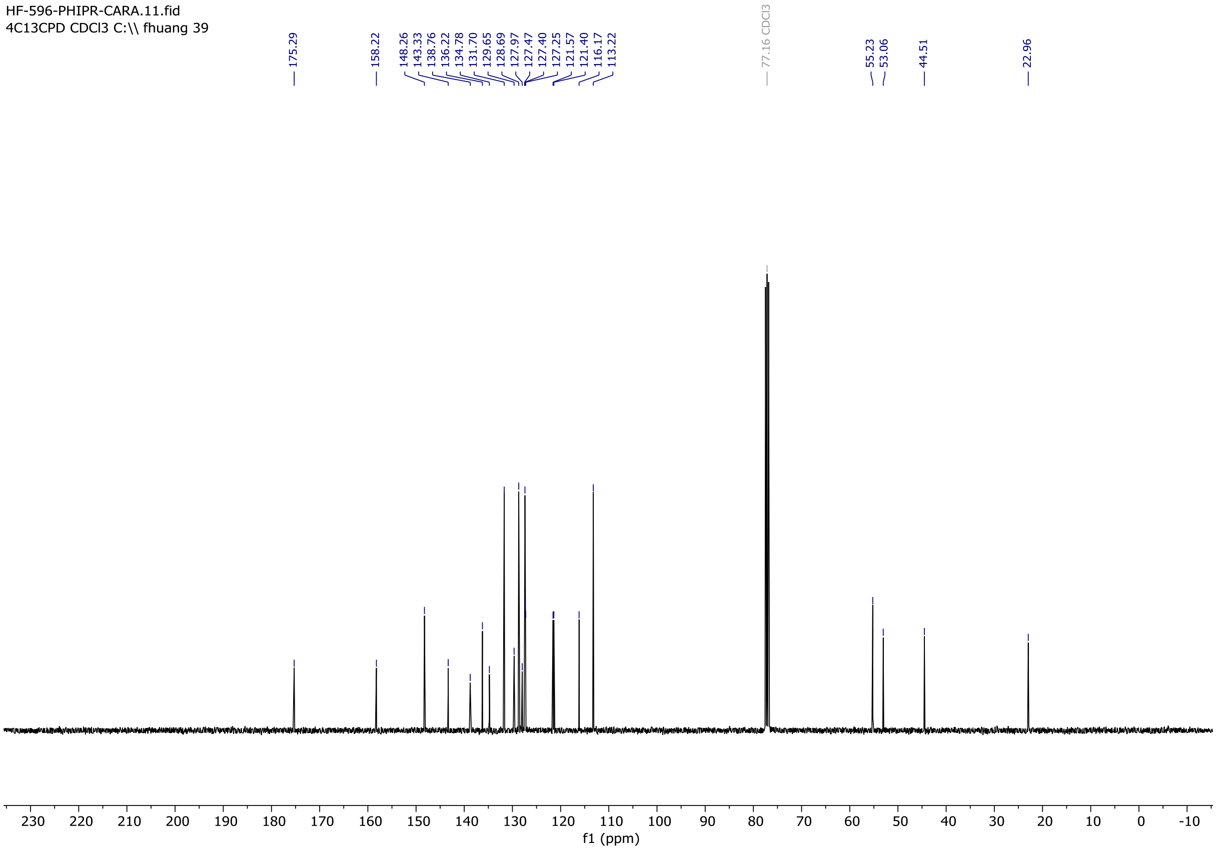
HF-596-PHIPR-CARA.11.fid
4C13CPD CDCl3 C:\\ fhuang 39
175.29
158.22
148.26
143.33
138.76
136.22
134.78
131.70
129.65
128.69
127.97
127.47
127.40
127.25
121.57
121.40
116.17
113.22
77.16 CDCl3
55.23
53.06
44.51
22.96
230 220 210 200 190 180 170 160 150 140 130 120 110 100 90 80 70 60 50 40 30 20 10 0 -10
f1 (ppm)

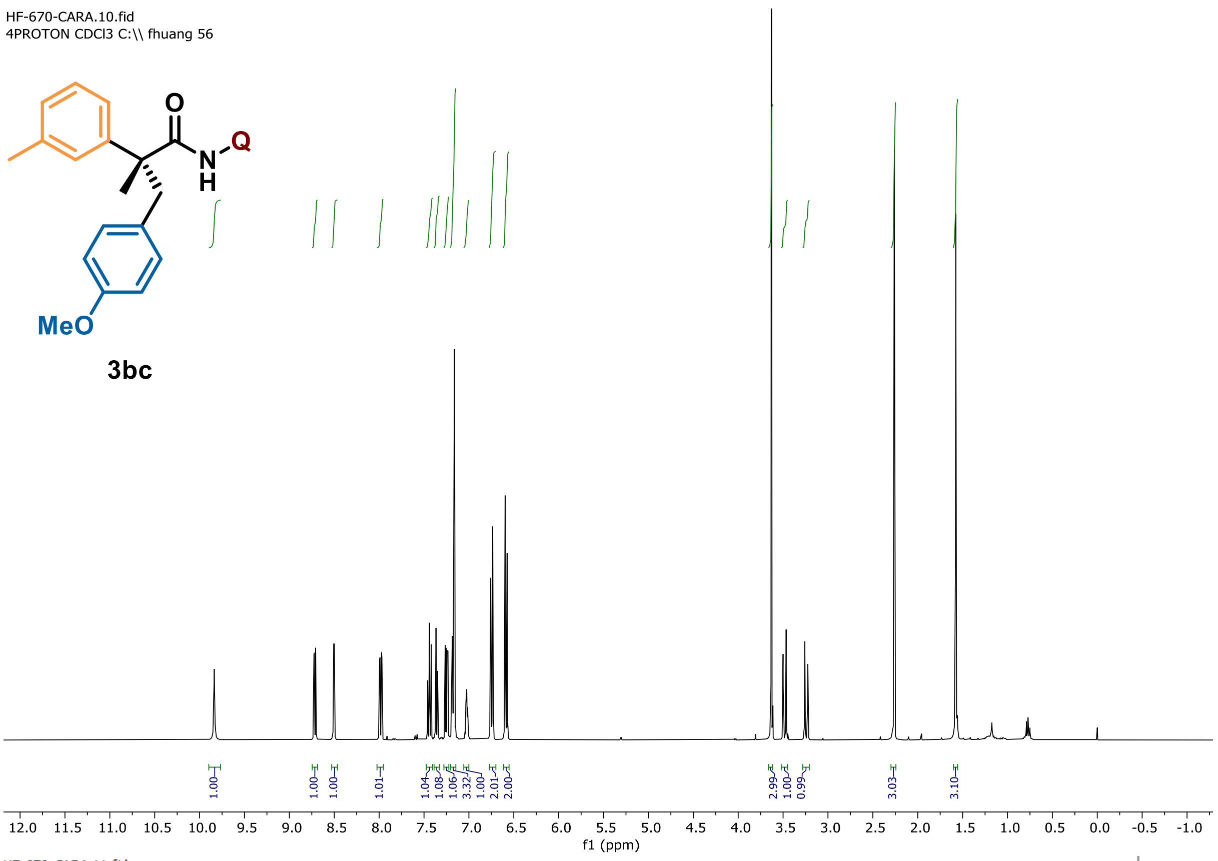
HF-670-CARA.10.fid
4PROTON CDCl3 C:\\ fhuang 56
O
N
H
Q
MeO
3bc
1.00
1.00
1.00
1.01
1.04
1.08
1.06
3.32
1.00
2.01
2.00
2.99
1.00
0.99
3.03
3.10
12.0 11.5 11.0 10.5 10.0 9.5 9.0 8.5 8.0 7.5 7.0 6.5 6.0 5.5 5.0 4.5 4.0 3.5 3.0 2.5 2.0 1.5 1.0 0.5 0.0 -0.5 -1.0
f1 (ppm)

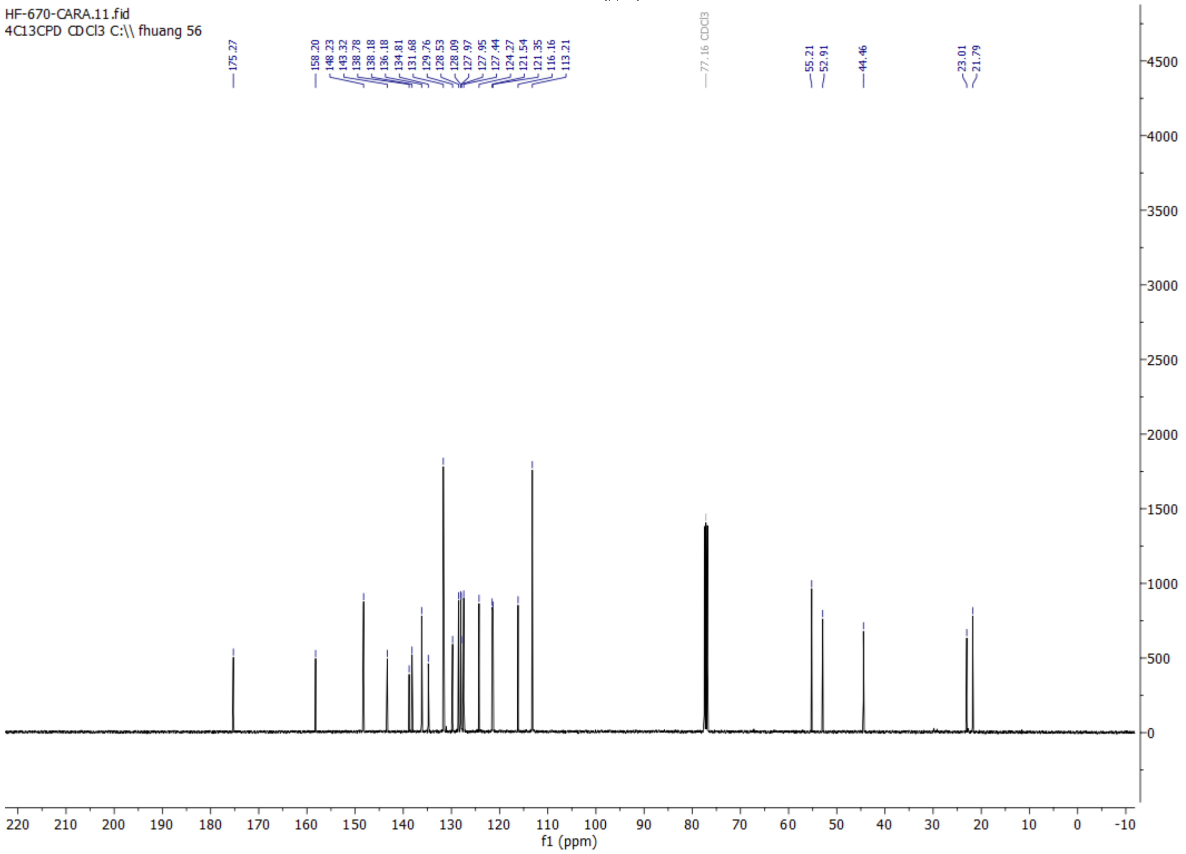
HF-670-CARA.11.fid
4C13CPD CDCl3 C:\\ fhuang 56
175.27
158.20
148.23
143.32
138.78
138.18
136.18
134.81
131.68
129.76
128.53
128.09
127.97
127.95
127.44
124.27
121.54
121.35
116.16
113.21
77.16 CDCl3
55.21
52.91
44.46
23.01
21.79
4500
4000
3500
3000
2500
2000
1500
1000
500
0
220 210 200 190 180 170 160 150 140 130 120 110 100 90 80 70 60 50 40 30 20 10 0 -10
f1 (ppm)

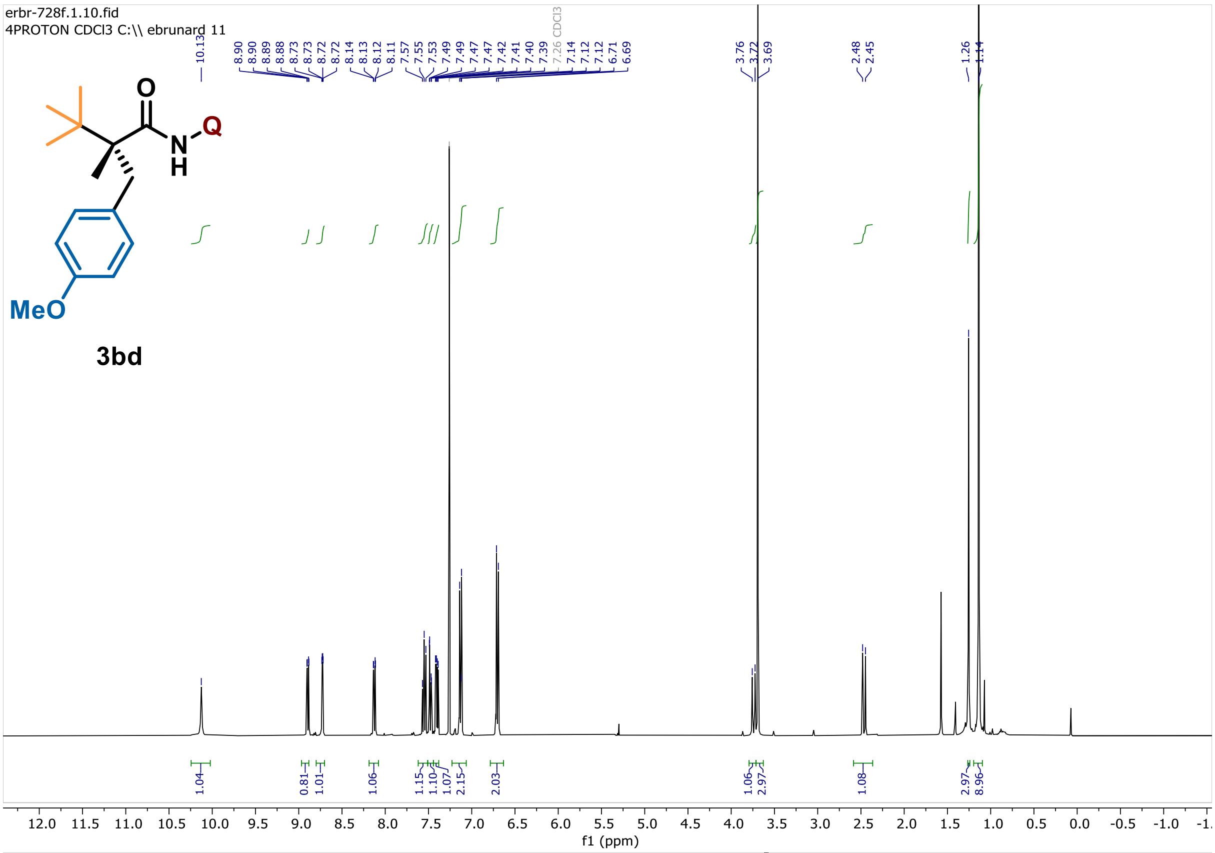
erbr-728f.1.10.fid
4PROTON CDCl3 C:\\ ebrunard 11
O
N
Q
H
MeO
3bd
10.13
8.90 8.90 8.89 8.88 8.73 8.73 8.72 8.72 8.14 8.13 8.12 8.11 7.57 7.55 7.53 7.49 7.49 7.47 7.47 7.42 7.41 7.40 7.39 7.26 CDCl3 7.14 7.12 7.12 6.71 6.69
3.76 3.72 3.69
2.48 2.45
1.26 1.14
1.04 0.81 1.01 1.06 1.15 1.10 1.07 2.15 2.03 1.06 2.97 1.08 2.97 8.96
f1 (ppm)

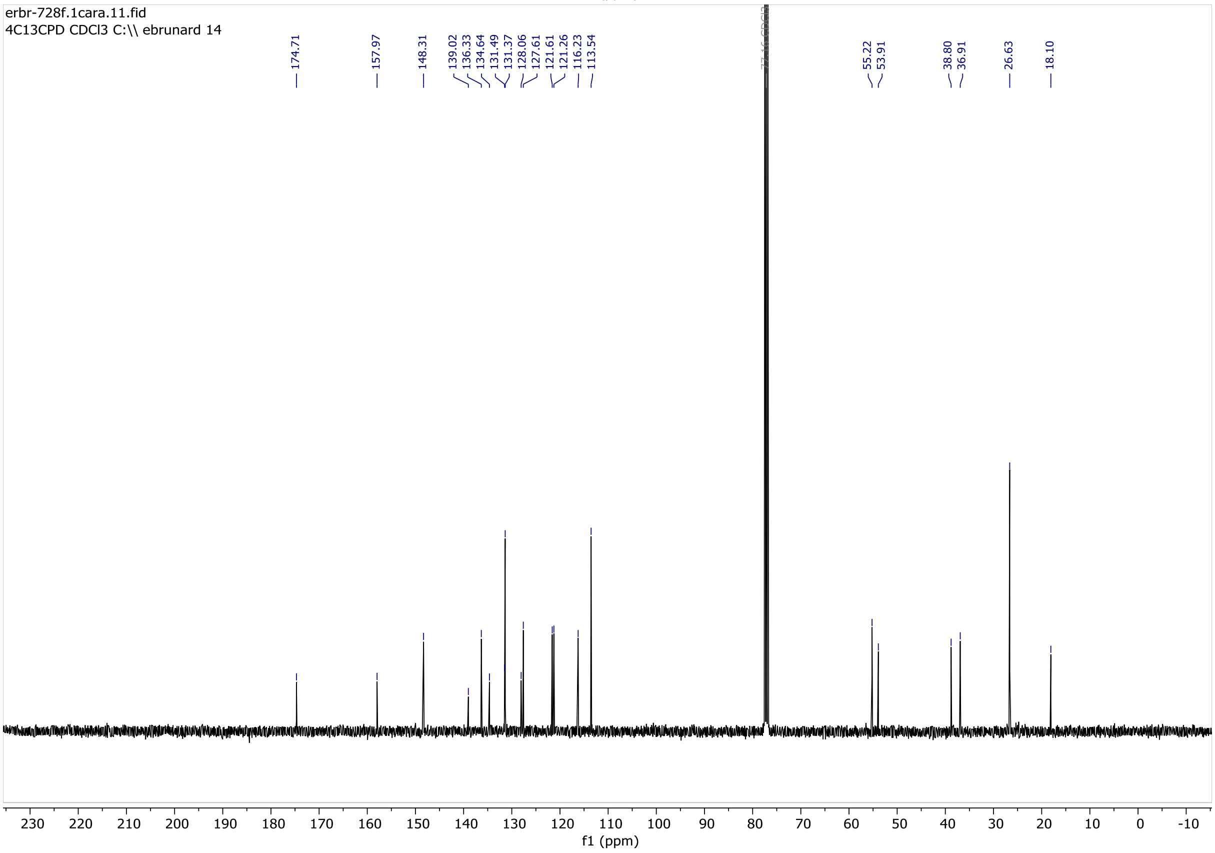
erbr-728f.1cara.11.fid
4C13CPD CDCl3 C:\\ ebrunard 14
174.71
157.97
148.31
139.02 136.33 134.64 131.49 131.37 128.06 127.61 121.61 121.26 116.23 113.54
55.22 53.91
38.80 36.91
26.63
18.10
f1 (ppm)

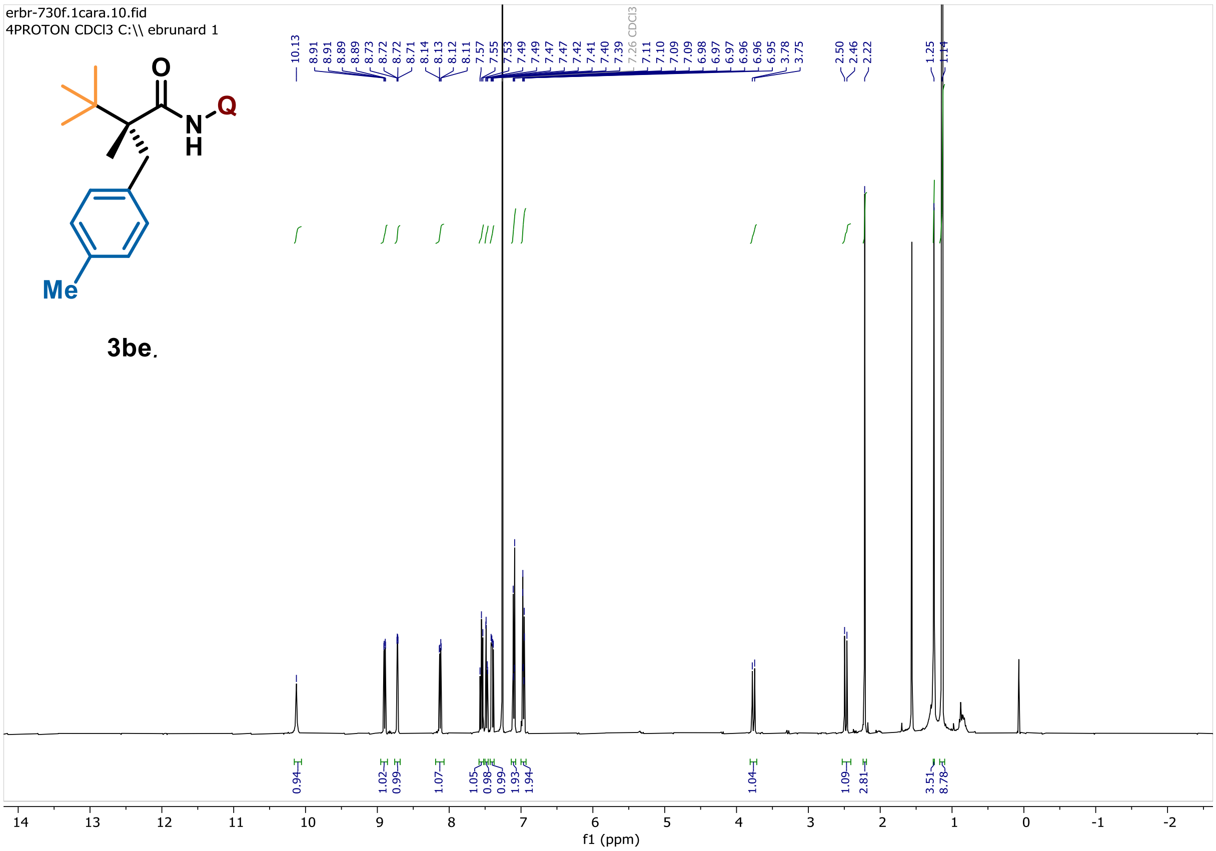
erbr-730f.1cara.10.fid
4PROTON CDCl3 C:\\ ebrunard 1
O
N
H
Q
Me
3be.
10.13
8.91
8.91
8.89
8.89
8.73
8.72
8.72
8.71
8.14
8.13
8.12
8.11
7.57
7.55
7.53
7.49
7.49
7.47
7.47
7.42
7.41
7.40
7.39
7.26 CDCl3
7.11
7.10
7.09
7.09
6.98
6.97
6.97
6.96
6.96
6.95
3.78
3.75
2.50
2.46
2.22
1.25
1.14
0.94
1.02
0.99
1.07
1.05
0.98
0.99
1.93
1.94
1.04
1.09
2.81
3.51
8.78
f1 (ppm)

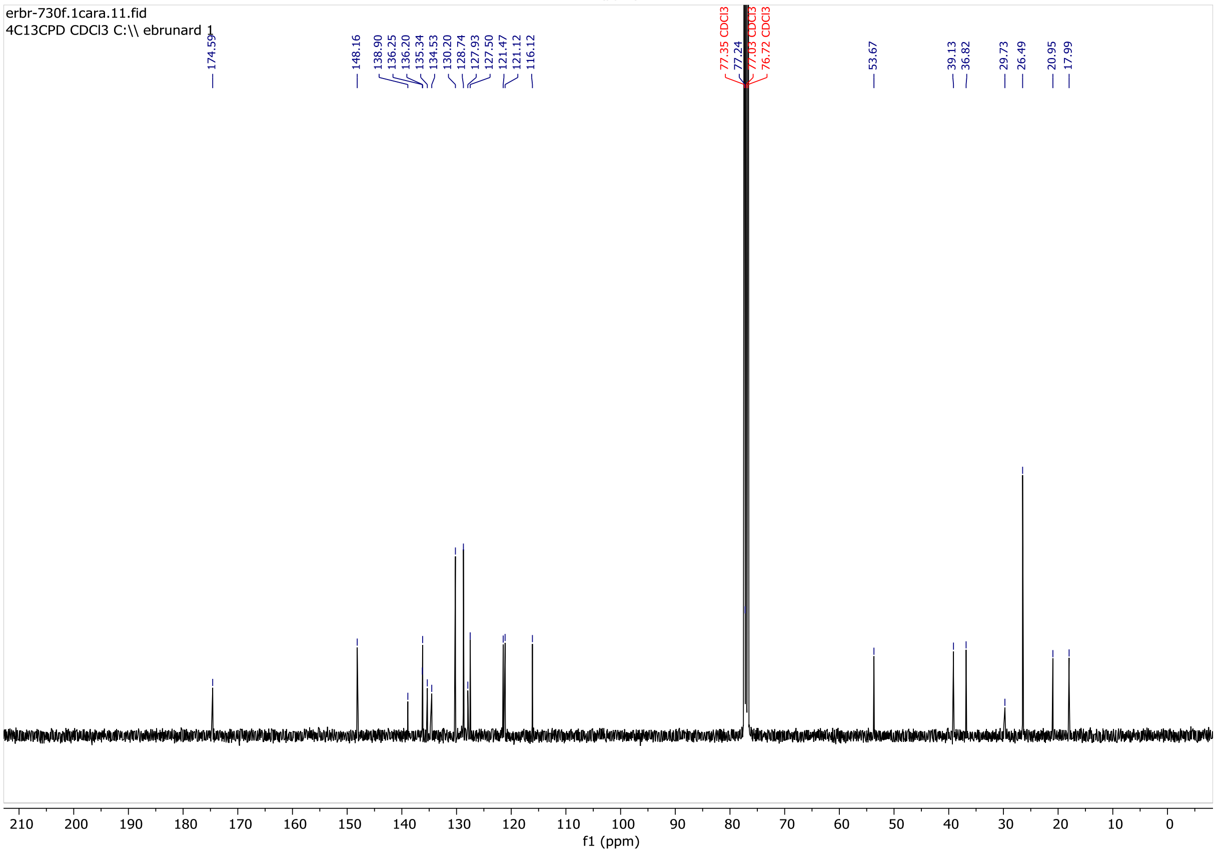
erbr-730f.1cara.11.fid
4C13CPD CDCl3 C:\\ ebrunard 1
174.59
148.16
138.90
136.25
136.20
135.34
134.53
130.20
128.74
127.93
127.50
121.47
121.12
116.12
77.35 CDCl3
77.24
77.03 CDCl3
76.72 CDCl3
53.67
39.13
36.82
29.73
26.49
20.95
17.99
f1 (ppm)

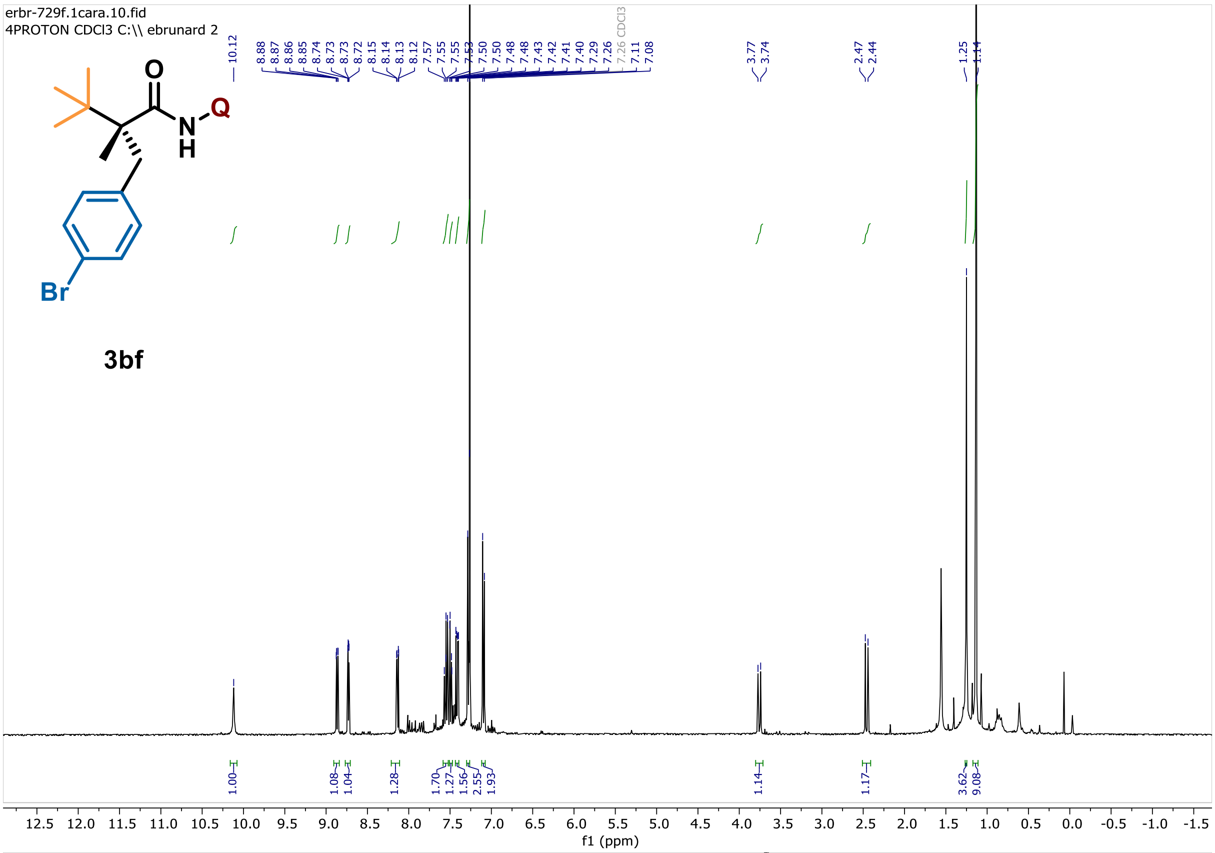
erbr-729f.1cara.10.fid
4PROTON CDCl3 C:\\ ebrunard 2
O
N
H
Q
Br
3bf
10.12
8.88
8.87
8.86
8.85
8.74
8.73
8.73
8.72
8.15
8.14
8.13
8.12
7.57
7.55
7.55
7.53
7.50
7.50
7.48
7.43
7.42
7.41
7.40
7.29
7.26
7.26 CDCl3
7.11
7.08
3.77
3.74
2.47
2.44
1.25
1.14
1.00
1.08
1.04
1.28
1.70
1.27
1.56
2.55
1.93
1.14
1.17
3.62
9.08
f1 (ppm)

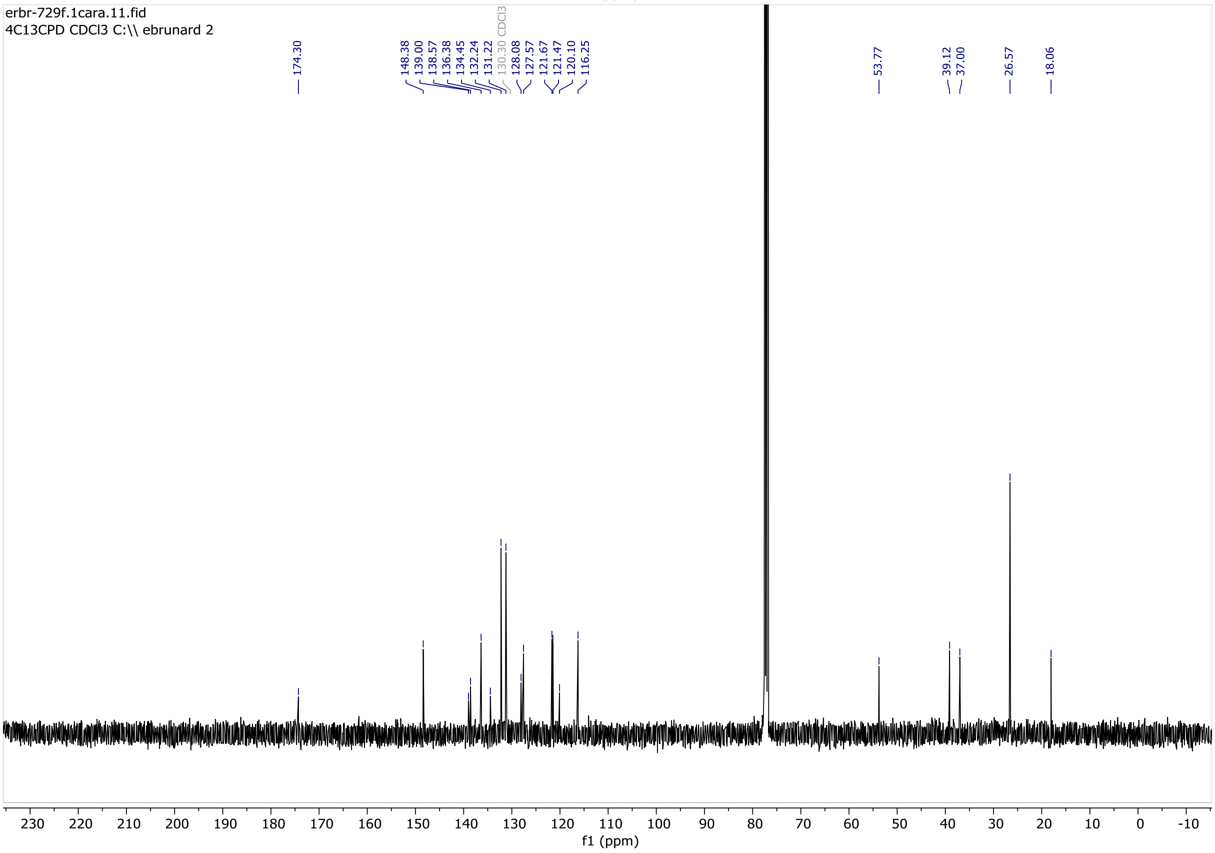
erbr-729f.1cara.11.fid
4C13CPD CDCl3 C:\\ ebrunard 2
174.30
148.38
139.00
138.57
136.38
134.45
132.24
131.22
130.30 CDCl3
128.08
127.57
121.67
121.47
120.10
116.25
53.77
39.12
37.00
26.57
18.06
f1 (ppm)

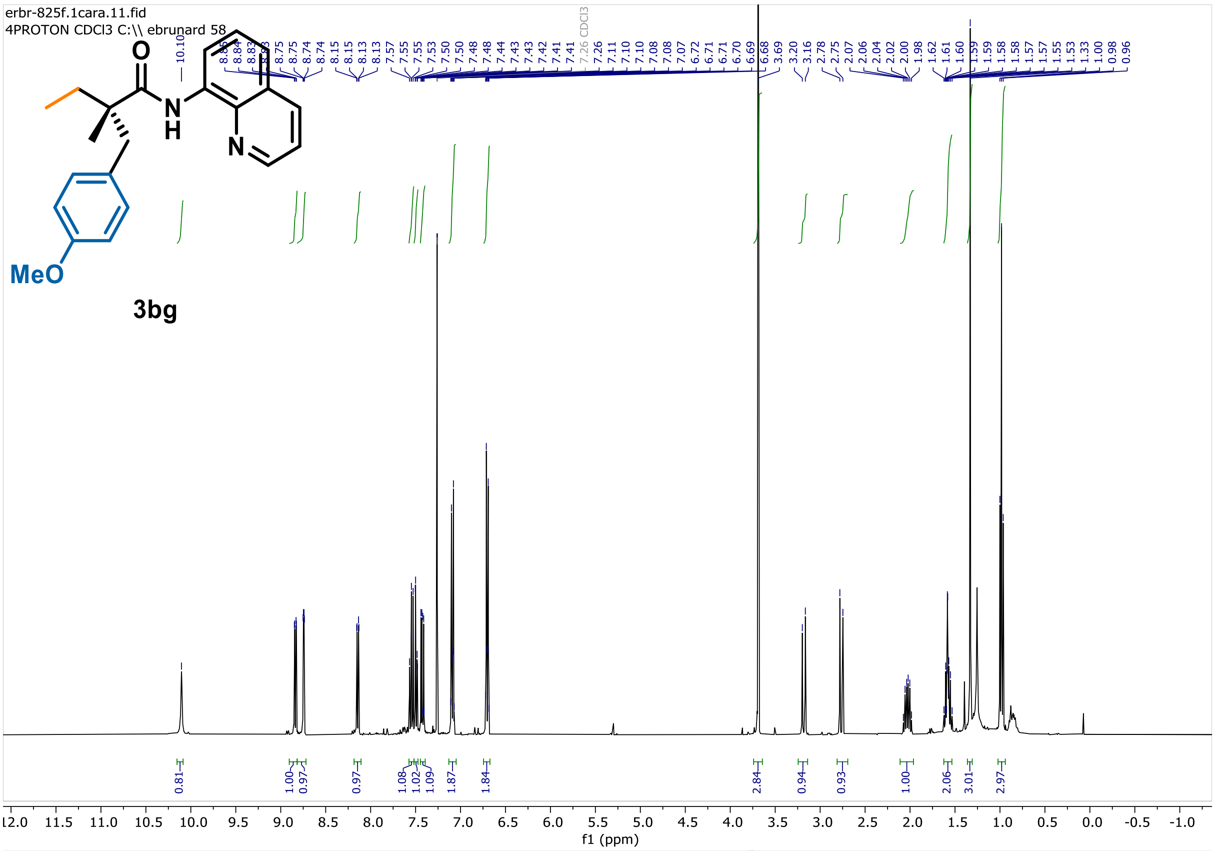
erbr-825f.1cara.11.fid
4PROTON CDCl3 C:\\ ebrunard 58
O
N
H
N
MeO
3bg
10.10
8.75
8.75
8.74
8.74
8.15
8.15
8.13
8.13
7.57
7.55
7.55
7.53
7.50
7.50
7.48
7.48
7.44
7.43
7.43
7.42
7.41
7.41
7.26 CDCl3
7.11
7.10
7.10
7.08
7.08
7.07
6.72
6.71
6.71
6.70
6.69
6.68
3.69
3.20
3.16
2.78
2.75
2.07
2.06
2.04
2.02
2.00
1.98
1.62
1.61
1.60
1.59
1.59
1.58
1.58
1.57
1.57
1.55
1.53
1.33
1.00
0.98
0.96
0.81
1.00
0.97
0.97
1.08
1.02
1.09
1.87
1.84
2.84
0.94
0.93
1.00
2.06
3.01
2.97
12.0 11.5 11.0 10.5 10.0 9.5 9.0 8.5 8.0 7.5 7.0 6.5 6.0 5.5 5.0 4.5 4.0 3.5 3.0 2.5 2.0 1.5 1.0 0.5 0.0 -0.5 -1.0
f1 (ppm)

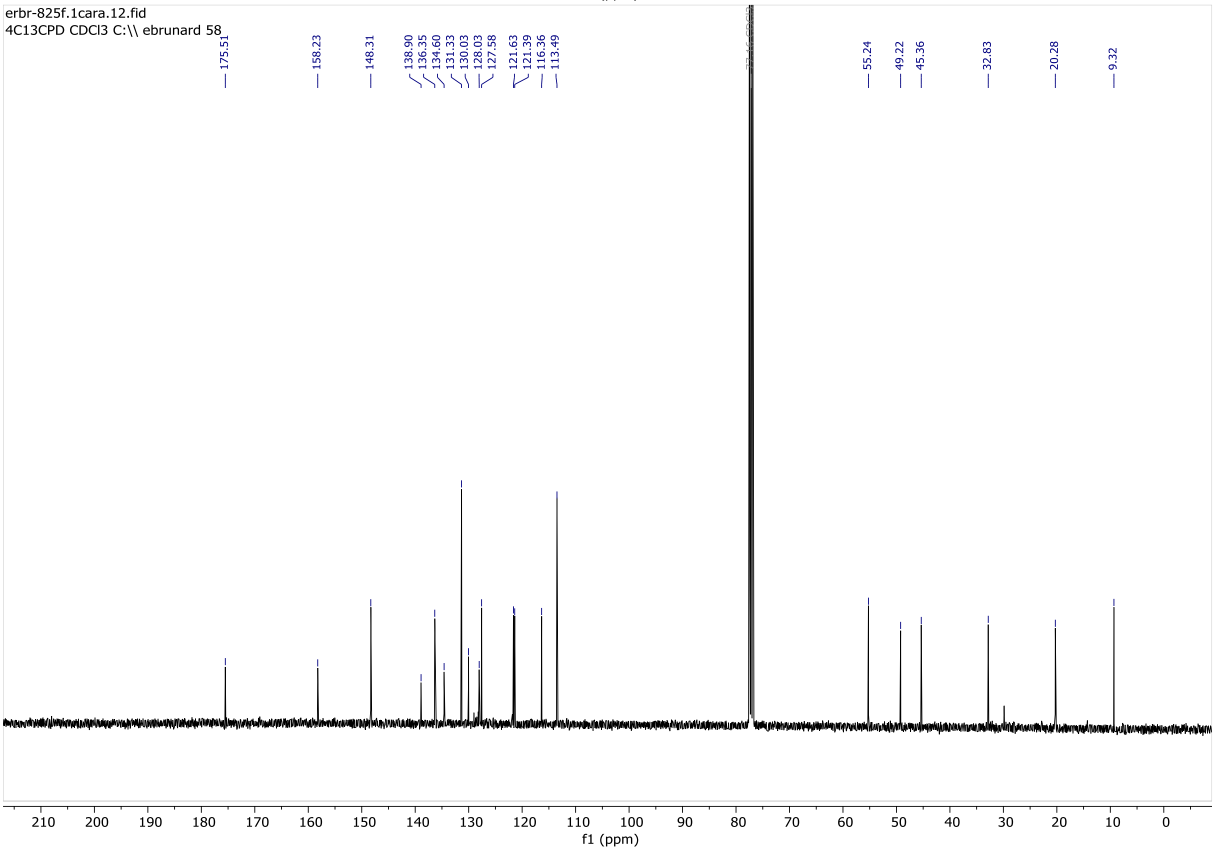
erbr-825f.1cara.12.fid
4C13CPD CDCl3 C:\\ ebrunard 58
175.51
158.23
148.31
138.90
136.35
134.60
131.33
130.03
128.03
127.58
121.63
121.39
116.36
113.49
55.24
49.22
45.36
32.83
20.28
9.32
210 200 190 180 170 160 150 140 130 120 110 100 90 80 70 60 50 40 30 20 10 0
f1 (ppm)

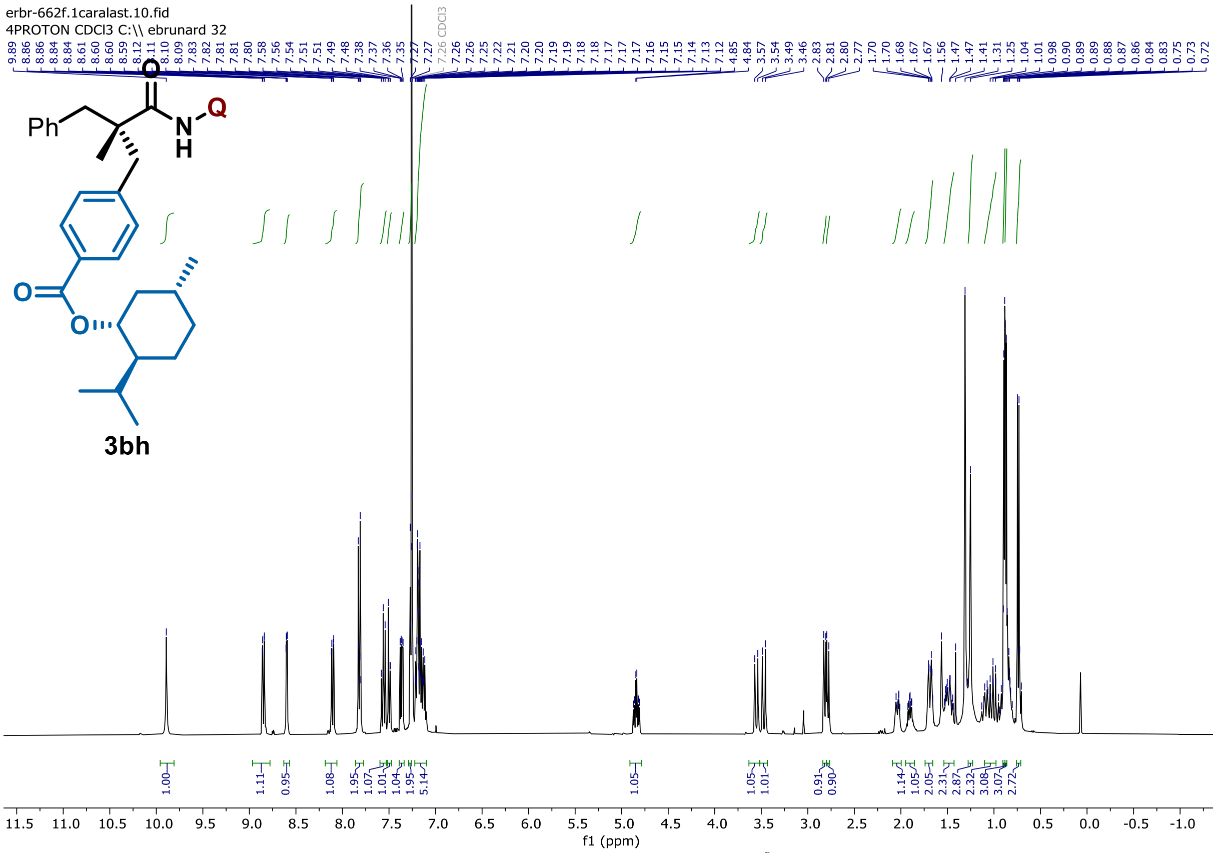
erbr-662f.1caralast.10.fid
4PROTON CDCl3 C:\\ ebrunard 32
9.89 8.86 8.86 8.84 8.84 8.61 8.60 8.60 8.59 8.12 8.11 8.10 8.09 7.83 7.82 7.81 7.81 7.80 7.58 7.56 7.54 7.51 7.51 7.49 7.48 7.38 7.37 7.36 7.35 7.27 7.26 CDCl3 7.26 7.25 7.22 7.21 7.20 7.20 7.19 7.19 7.18 7.18 7.17 7.17 7.16 7.15 7.15 7.14 7.13 7.12 4.85 4.84 3.57 3.54 3.49 3.46 2.83 2.81 2.80 2.77 1.70 1.68 1.67 1.67 1.56 1.47 1.47 1.41 1.31 1.25 1.04 1.01 0.98 0.90 0.89 0.89 0.88 0.87 0.86 0.84 0.83 0.75 0.73 0.72
Ph
O
N
Q
H
O
O
3bh
1.00 1.11 0.95 1.08 1.95 1.07 1.01 1.04 1.95 5.14 1.05 1.05 1.01 0.91 0.90 1.14 1.05 2.05 2.31 2.87 2.32 3.08 3.07 2.72
11.5 11.0 10.5 10.0 9.5 9.0 8.5 8.0 7.5 7.0 6.5 6.0 5.5 5.0 4.5 4.0 3.5 3.0 2.5 2.0 1.5 1.0 0.5 0.0 -0.5 -1.0
f1 (ppm)

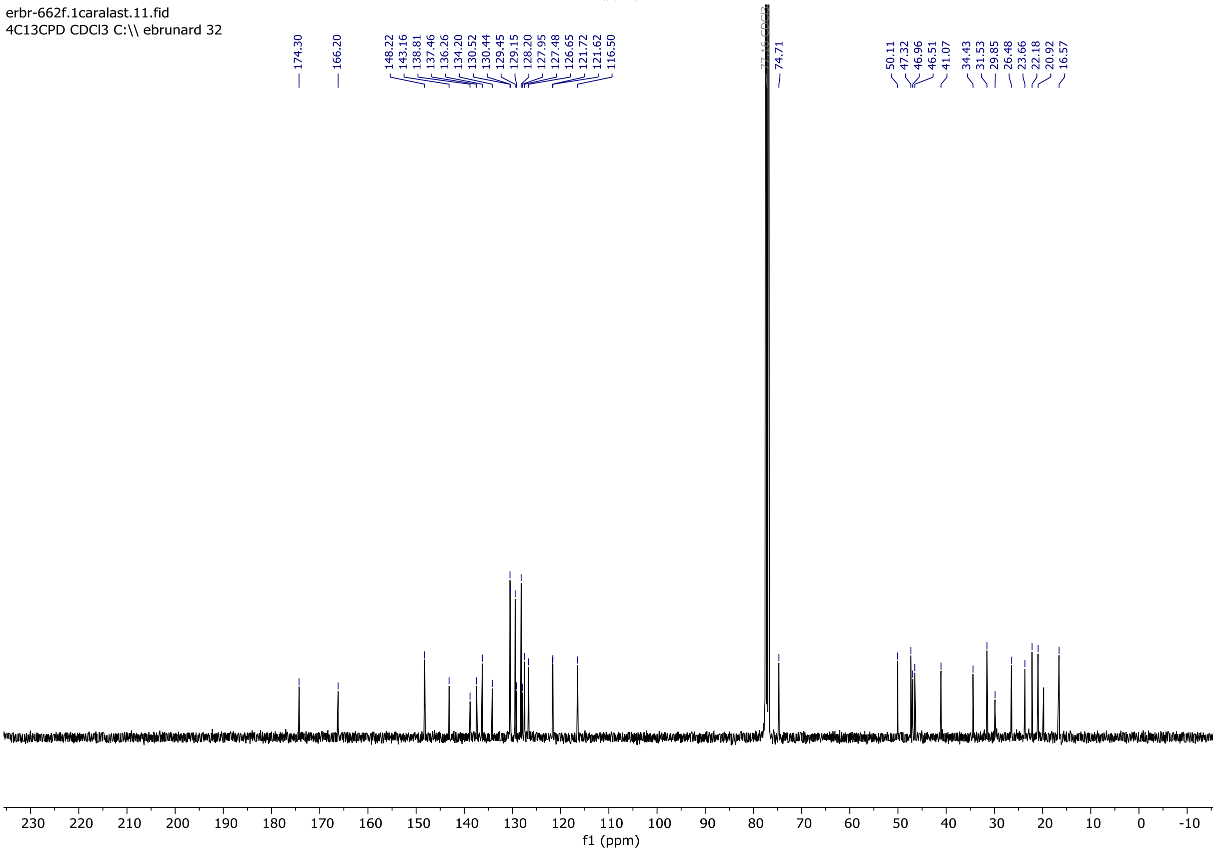
erbr-662f.1caralast.11.fid
4C13CPD CDCl3 C:\\ ebrunard 32
174.30 166.20 148.22 143.16 138.81 137.46 136.26 134.20 130.52 130.44 129.45 129.15 128.20 127.95 127.48 126.65 121.72 121.62 116.50 77.16 CDCl3 74.71 50.11 47.32 46.96 46.51 41.07 34.43 31.53 29.85 26.48 23.66 22.18 20.92 16.57
230 220 210 200 190 180 170 160 150 140 130 120 110 100 90 80 70 60 50 40 30 20 10 0 -10
f1 (ppm)

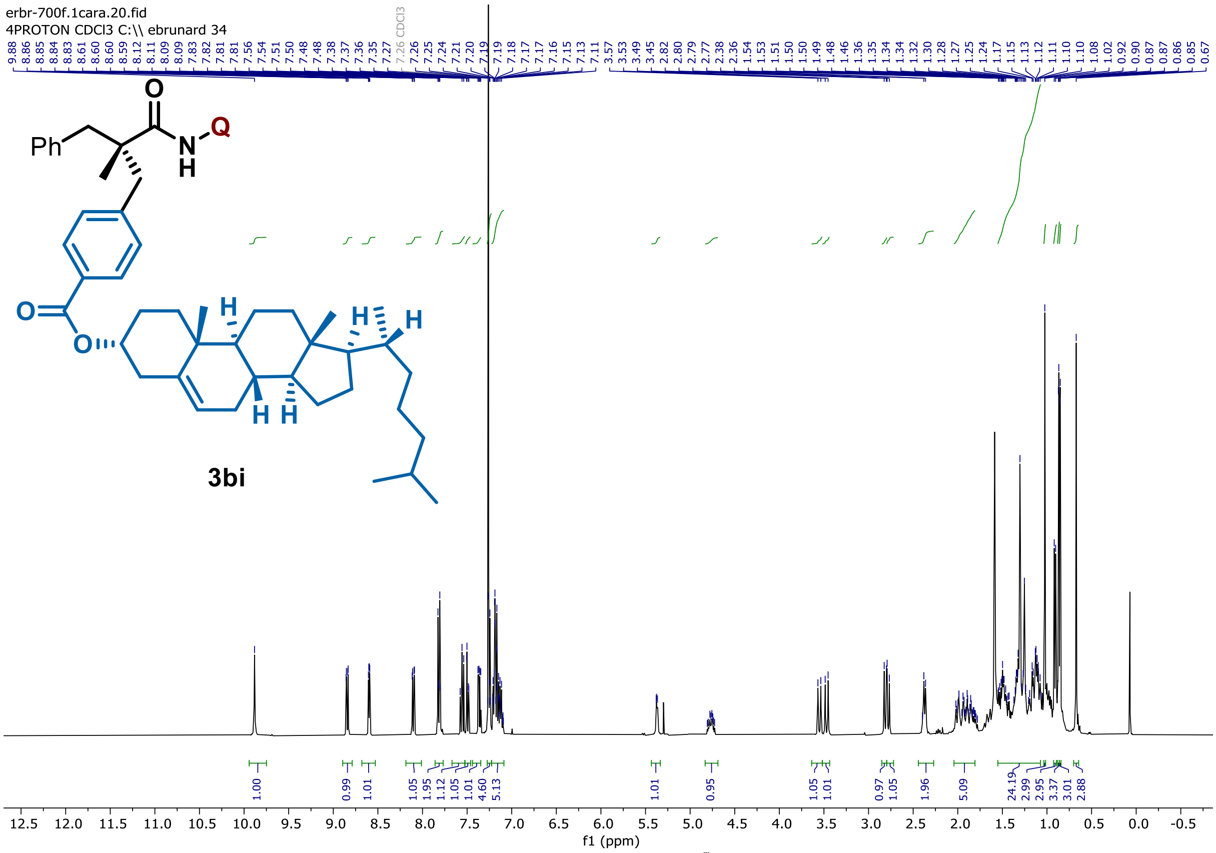
erbr-700f.1cara.20.fid
4PROTON CDCl3 C:\\ ebrunard 34
9.88 8.86 8.85 8.84 8.83 8.61 8.60 8.59 8.12 8.11 8.09 8.09 7.83 7.82 7.81 7.81 7.56 7.54 7.51 7.50 7.48 7.48 7.38 7.37 7.36 7.35 7.27 7.26 CDCl3 7.25 7.24 7.21 7.20 7.19 7.19 7.18 7.17 7.17 7.16 7.15 7.13 7.11 3.57 3.53 3.49 3.45 2.82 2.80 2.79 2.77 2.38 2.36 1.54 1.53 1.51 1.50 1.50 1.49 1.48 1.46 1.36 1.35 1.34 1.34 1.32 1.30 1.28 1.27 1.25 1.24 1.17 1.15 1.13 1.12 1.11 1.10 1.08 1.02 0.92 0.90 0.87 0.87 0.86 0.85 0.67
Ph
O
N
H
Q
O
O
H
H
H
H H
3bi
1.00 0.99 1.01 1.05 1.95 1.12 1.05 1.01 4.60 5.13 1.01 0.95 1.05 1.01 0.97 1.05 1.96 5.09 24.19 2.99 2.95 3.37 3.01 2.88
12.5 12.0 11.5 11.0 10.5 10.0 9.5 9.0 8.5 8.0 7.5 7.0 6.5 6.0 5.5 5.0 4.5 4.0 3.5 3.0 2.5 2.0 1.5 1.0 0.5 0.0 -0.5
f1 (ppm)

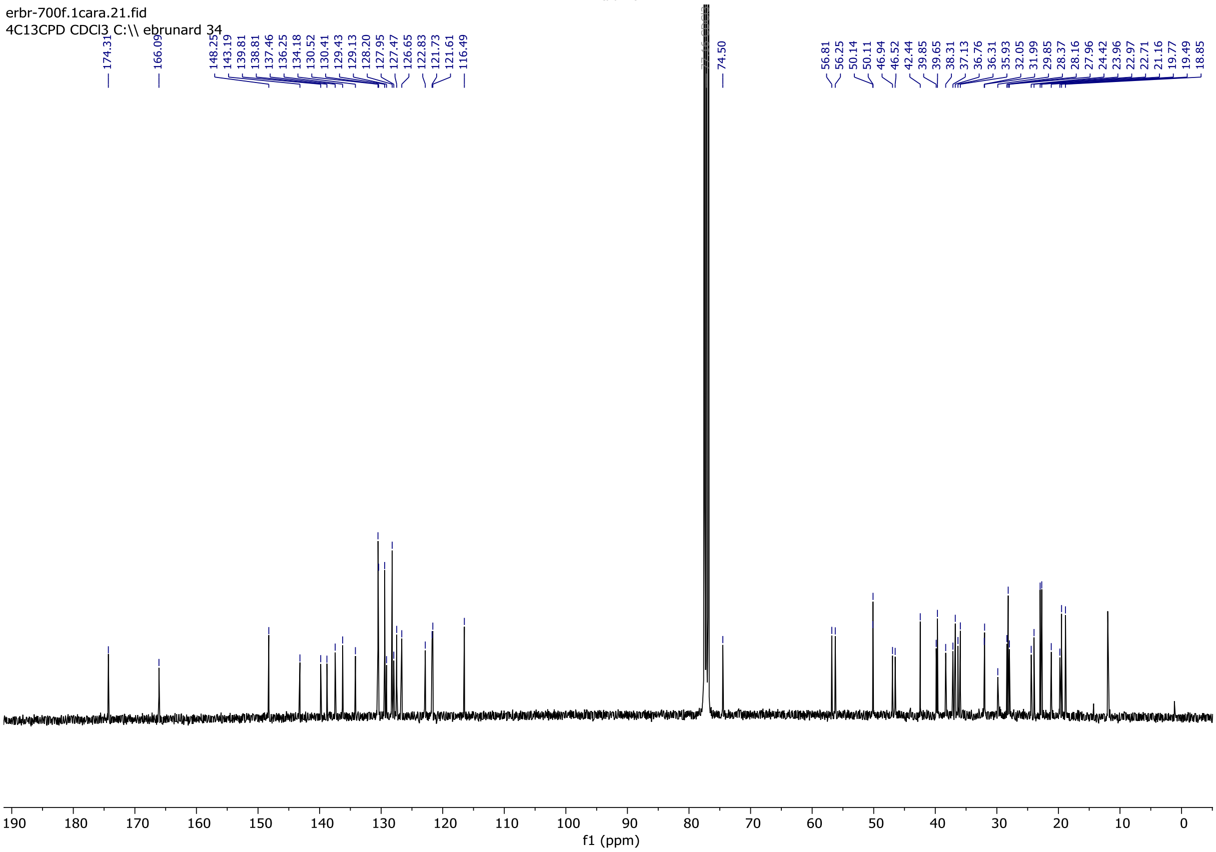
erbr-700f.1cara.21.fid
4C13CPD CDCl3 C:\\ ebrunard 34
174.31 166.09 148.25 143.19 139.81 138.81 137.46 136.25 134.18 130.52 130.41 129.43 129.13 128.20 127.95 127.47 126.65 122.83 121.73 121.61 116.49 74.50 56.81 56.25 50.14 50.11 46.94 46.52 42.44 39.85 39.65 38.31 37.13 36.76 36.31 35.93 32.05 31.99 29.85 28.37 28.16 27.96 24.42 23.96 22.97 22.71 21.16 19.77 19.49 18.85
190 180 170 160 150 140 130 120 110 100 90 80 70 60 50 40 30 20 10 0
f1 (ppm)

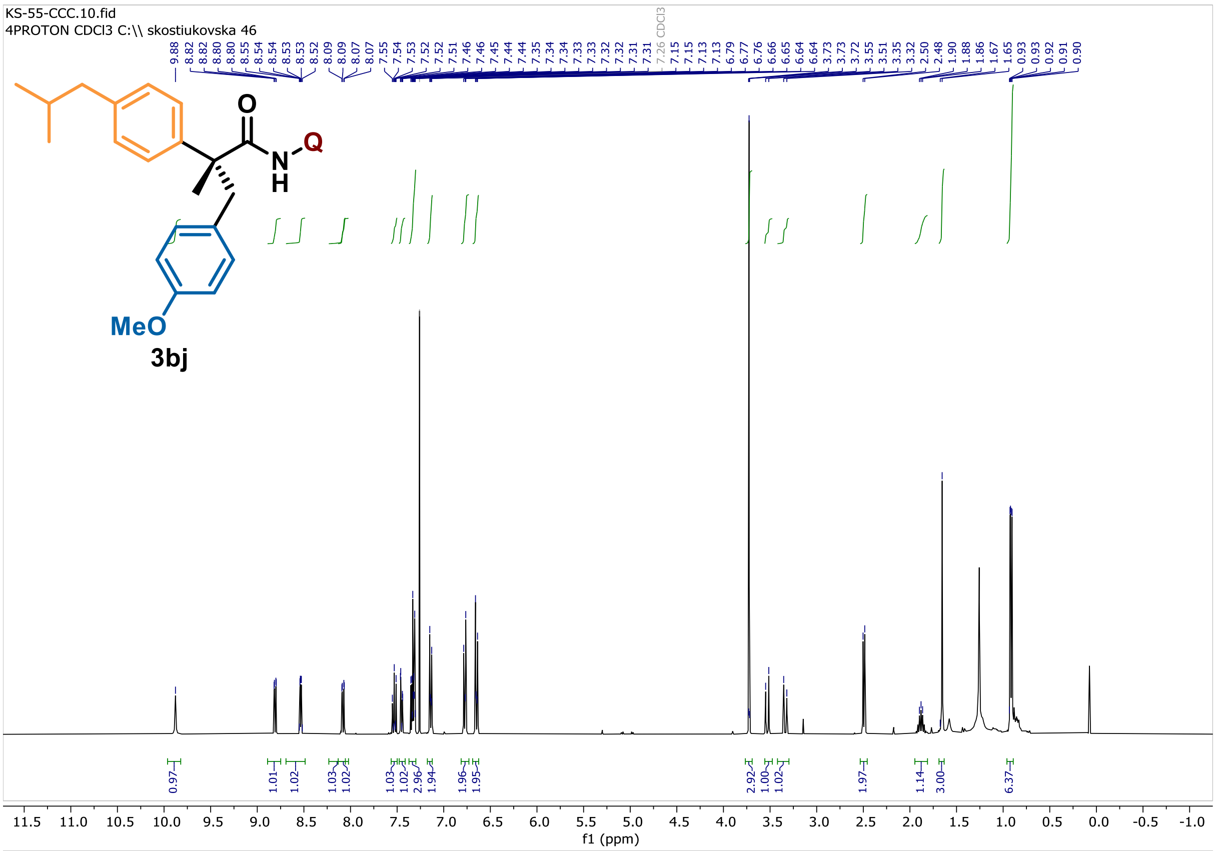
KS-55-CCC.10.fid
4PROTON CDCl3 C:\\ skostiukovska 46
9.88
8.82
8.82
8.80
8.80
8.55
8.54
8.54
8.53
8.53
8.52
8.09
8.09
8.07
8.07
7.55
7.54
7.53
7.52
7.52
7.51
7.46
7.46
7.45
7.44
7.35
7.34
7.34
7.33
7.33
7.32
7.32
7.31
7.31
7.26 CDCl3
7.15
7.15
7.13
7.13
6.79
6.77
6.76
6.66
6.65
6.64
6.64
3.73
3.73
3.72
3.55
3.51
3.35
3.32
2.50
2.48
1.90
1.88
1.86
1.67
1.65
0.93
0.93
0.92
0.91
0.90
O
N
Q
H
MeO
3bj
0.97
1.01
1.02
1.03
1.02
1.03
1.02
2.96
1.94
1.96
1.95
2.92
1.00
1.02
1.97
1.14
3.00
6.37
11.5
11.0
10.5
10.0
9.5
9.0
8.5
8.0
7.5
7.0
6.5
6.0
5.5
5.0
4.5
4.0
3.5
3.0
2.5
2.0
1.5
1.0
0.5
0.0
-0.5
-1.0
f1 (ppm)

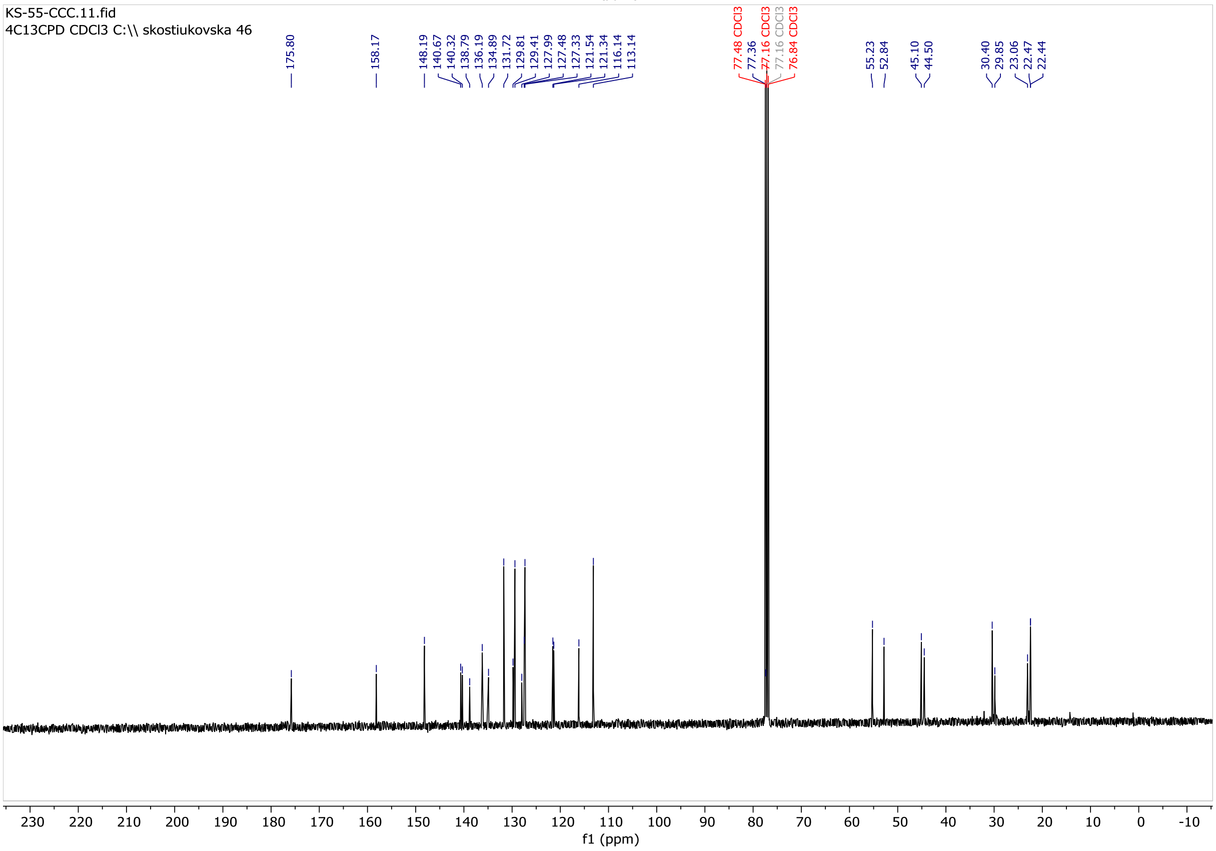
KS-55-CCC.11.fid
4C13CPD CDCl3 C:\\ skostiukovska 46
175.80
158.17
148.19
140.67
140.32
138.79
136.19
134.89
131.72
129.81
129.41
127.99
127.48
127.33
121.54
121.34
116.14
113.14
77.48 CDCl3
77.36
77.16 CDCl3
77.16 CDCl3
76.84 CDCl3
55.23
52.84
45.10
44.50
30.40
29.85
23.06
22.47
22.44
230
220
210
200
190
180
170
160
150
140
130
120
110
100
90
80
70
60
50
40
30
20
10
0
-10
f1 (ppm)

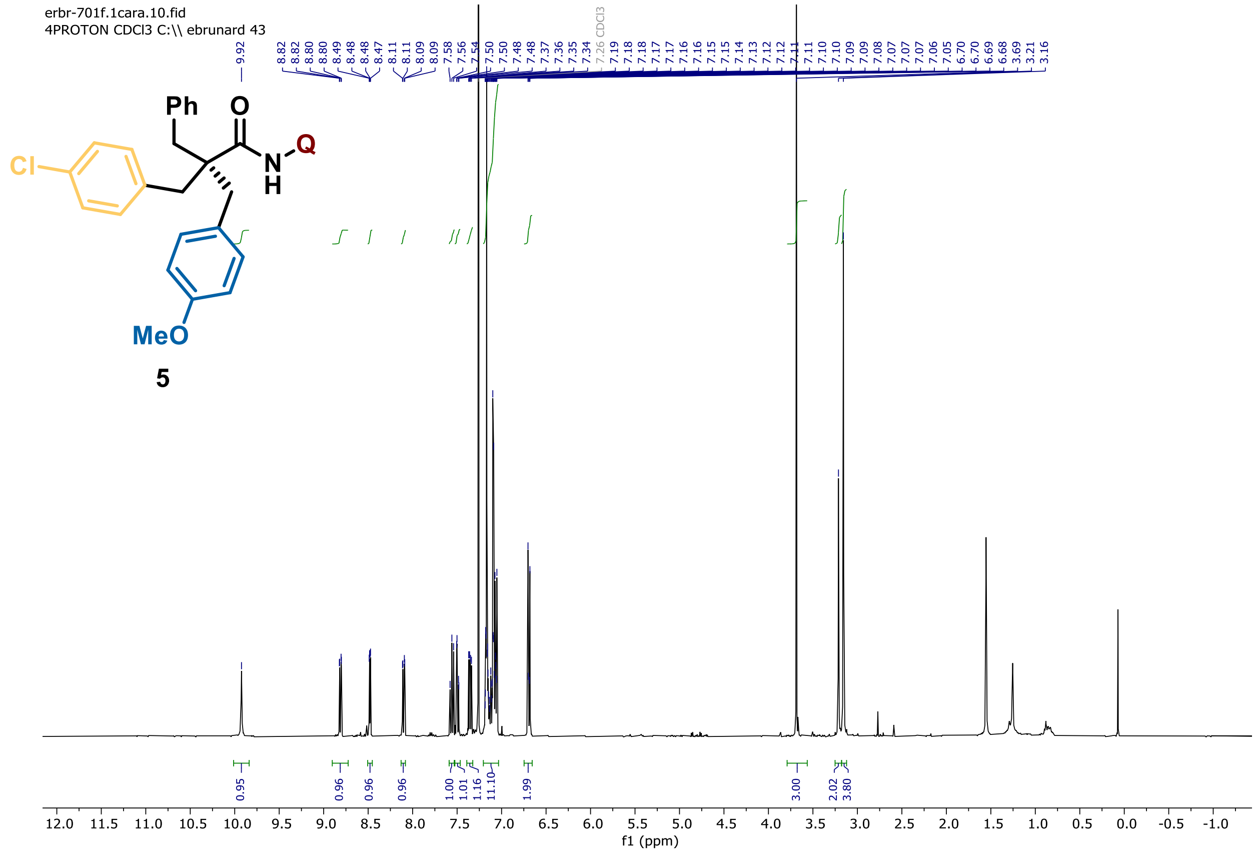
erbr-701f.1cara.10.fid
4PROTON CDCl3 C:\\ ebrunard 43
9.92
8.82 8.82 8.80 8.49 8.48 8.48 8.47 8.11 8.11 8.09 8.09 7.58 7.56 7.54 7.50 7.50 7.48 7.48 7.37 7.36 7.35 7.34
7.26 CDCl3
7.19 7.18 7.18 7.17 7.17 7.16 7.16 7.15 7.15 7.14 7.13 7.12 7.12 7.11 7.11 7.10 7.10 7.09 7.09 7.08 7.07 7.07 7.06 7.05 6.70 6.69 6.68 3.69 3.21 3.16
Ph
O
Cl
N
Q
H
MeO
5
0.95 0.96 0.96 0.96 1.00 1.01 1.16 11.10 1.99 3.00 2.02 3.80
12.0 11.5 11.0 10.5 10.0 9.5 9.0 8.5 8.0 7.5 7.0 6.5 6.0 5.5 5.0 4.5 4.0 3.5 3.0 2.5 2.0 1.5 1.0 0.5 0.0 -0.5 -1.0
f1 (ppm)

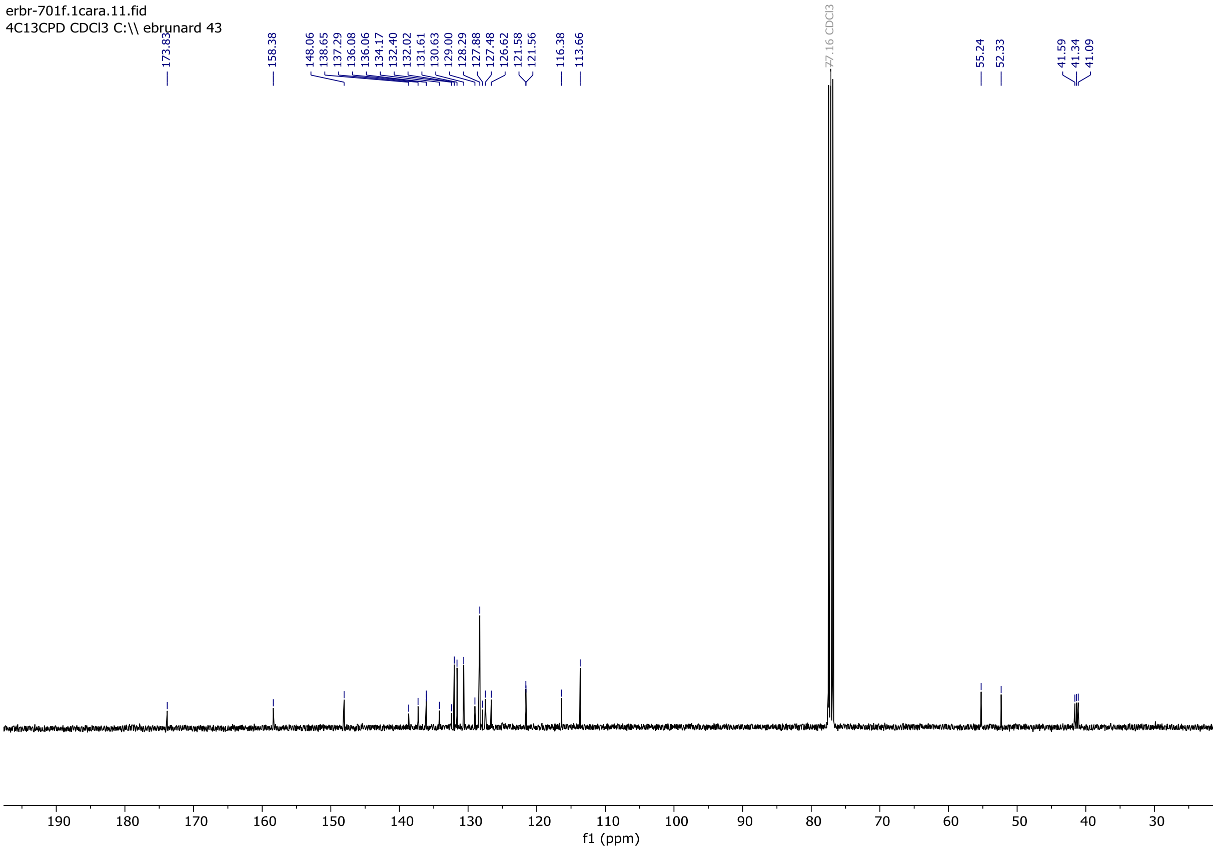
erbr-701f.1cara.11.fid
4C13CPD CDCl3 C:\\ ebrunard 43
173.83
158.38
148.06 138.65 137.29 136.08 136.06 134.17 132.40 132.02 131.61 130.63 129.00 128.29 127.88 127.48 126.62 121.58 121.56
116.38
113.66
77.16 CDCl3
55.24
52.33
41.59 41.34 41.09
190 180 170 160 150 140 130 120 110 100 90 80 70 60 50 40 30
f1 (ppm)

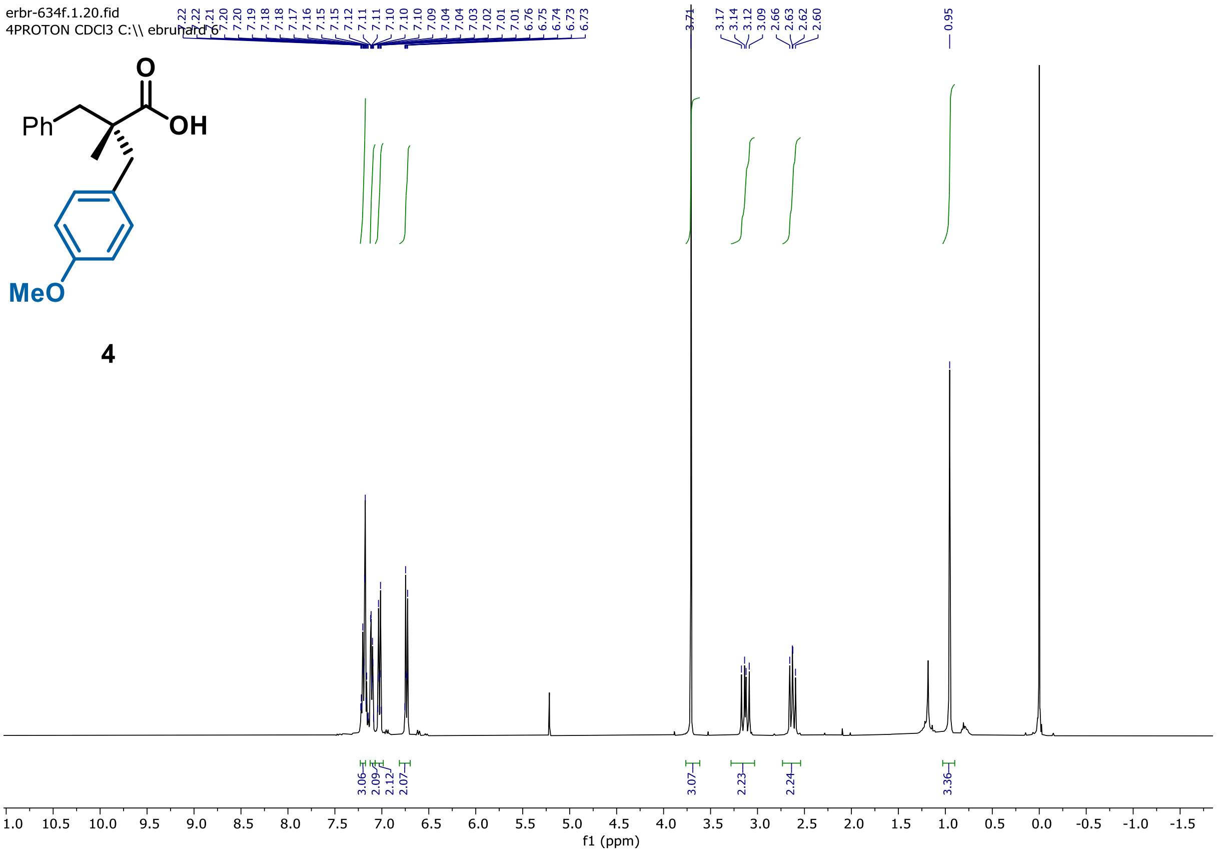
erbr-634f.1.20.fid
4PROTON CDCl3 C:\\ ebrunard 6
O
OH
Ph
MeO
4
3.71
0.95
3.06
2.09
2.12
2.07
3.07
2.23
2.24
3.36
f1 (ppm)

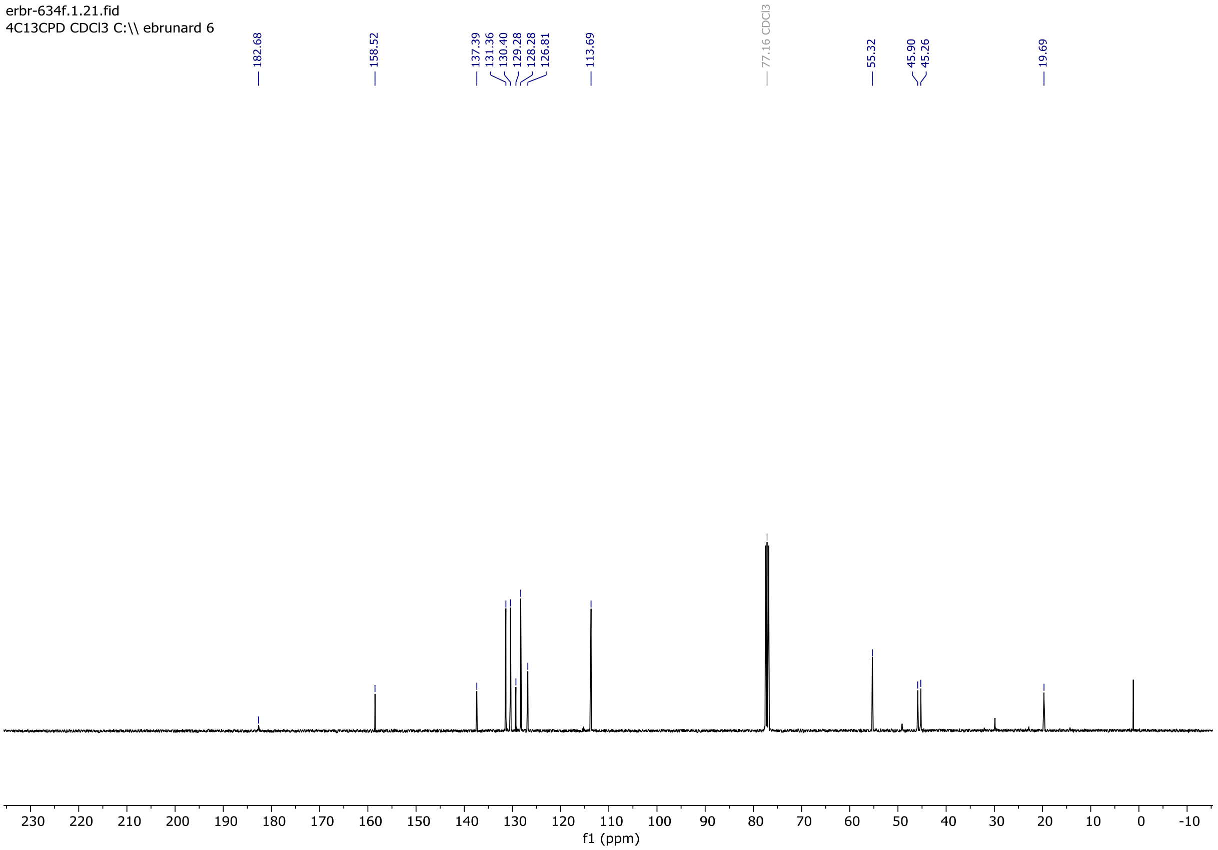
erbr-634f.1.21.fid
4C13CPD CDCl3 C:\\ ebrunard 6
182.68
158.52
137.39
131.36
130.40
129.28
128.28
126.81
113.69
77.16 CDCl3
55.32
45.90
45.26
19.69
f1 (ppm)

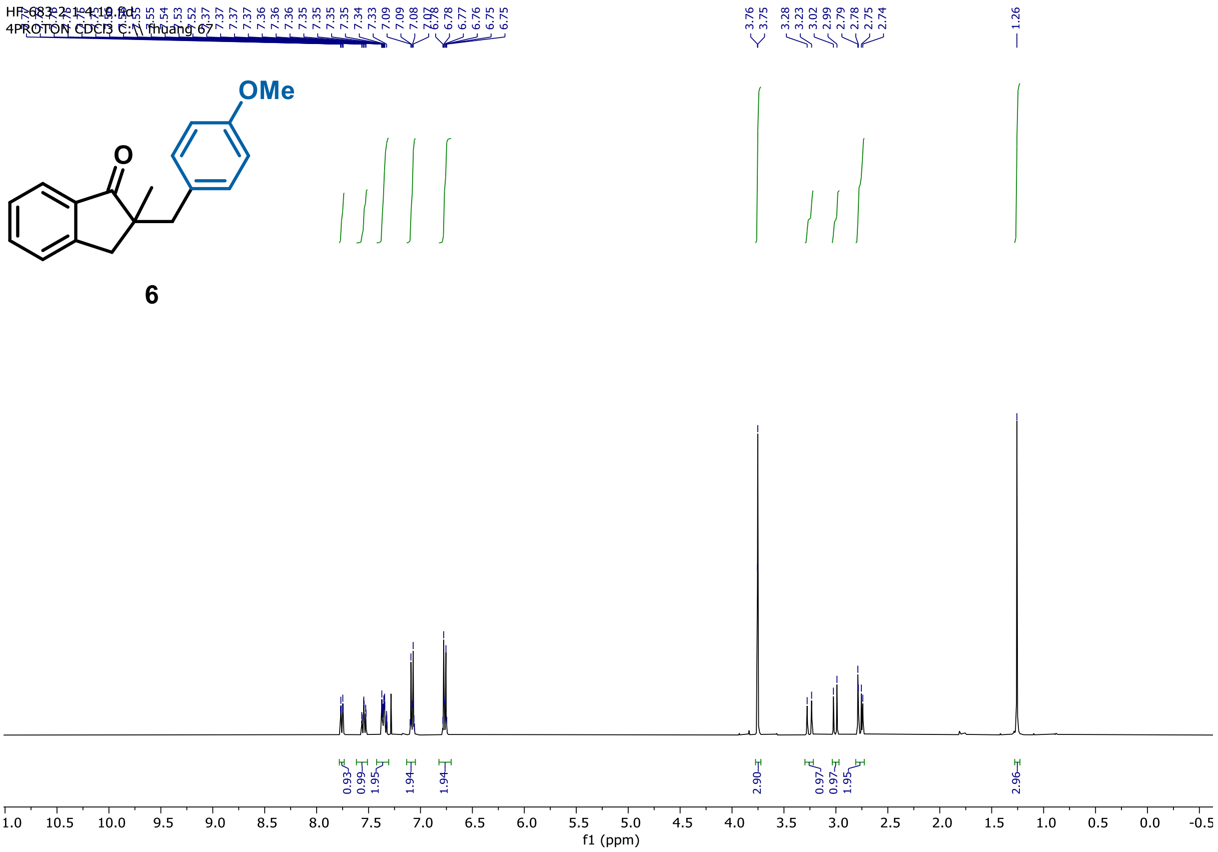
HF-683-2-1-4-19.fid
4PROTON CDCl3 C:\\ Thuang 67
OMe
O
3.76
3.75
3.28
3.23
3.02
2.99
2.79
2.78
2.75
2.74
1.26
0.93
0.99
1.95
1.94
1.94
2.90
0.97
0.97
1.95
2.96
f1 (ppm)

6

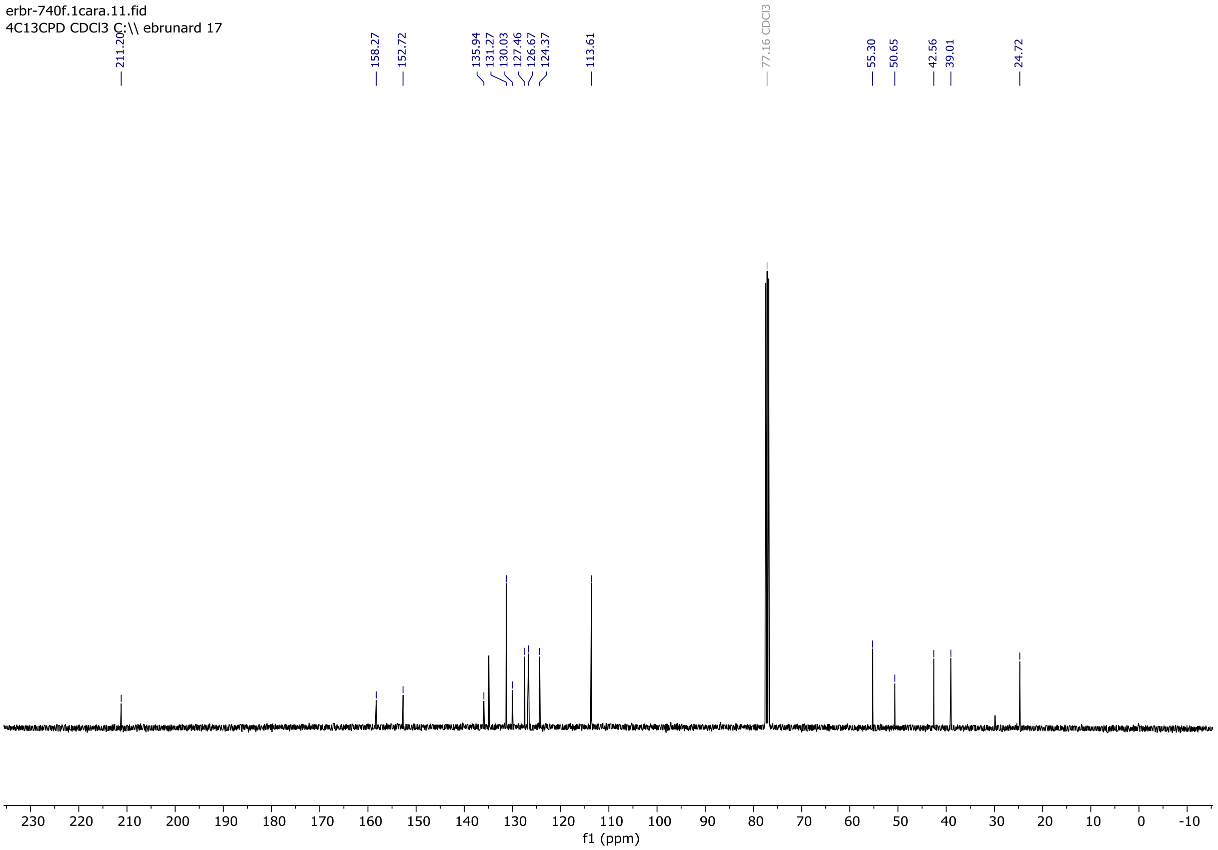
erbr-740f.1cara.11.fid
4C13CPD CDCl3 C:\\ ebrunard 17
211.20
158.27
152.72
135.94
131.27
130.03
127.46
126.67
124.37
113.61
77.16 CDCl3
55.30
50.65
42.56
39.01
24.72
f1 (ppm)

erbr-686f.1cara.10.fid
4PROTON CDCl3 C:\\ ebrunard 19

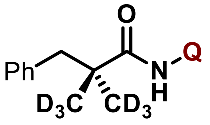

[D]1aa

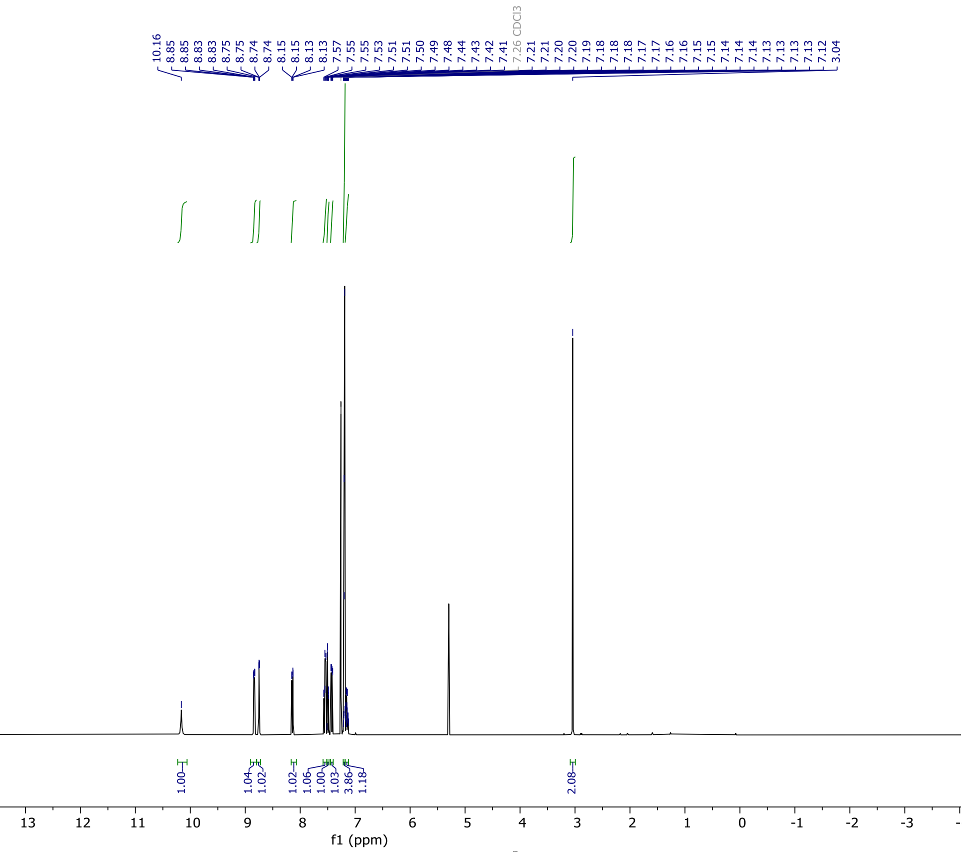

erbr-686f.1cara.11.fid
4C13CPD CDCl3 C:\\ ebrunard 19

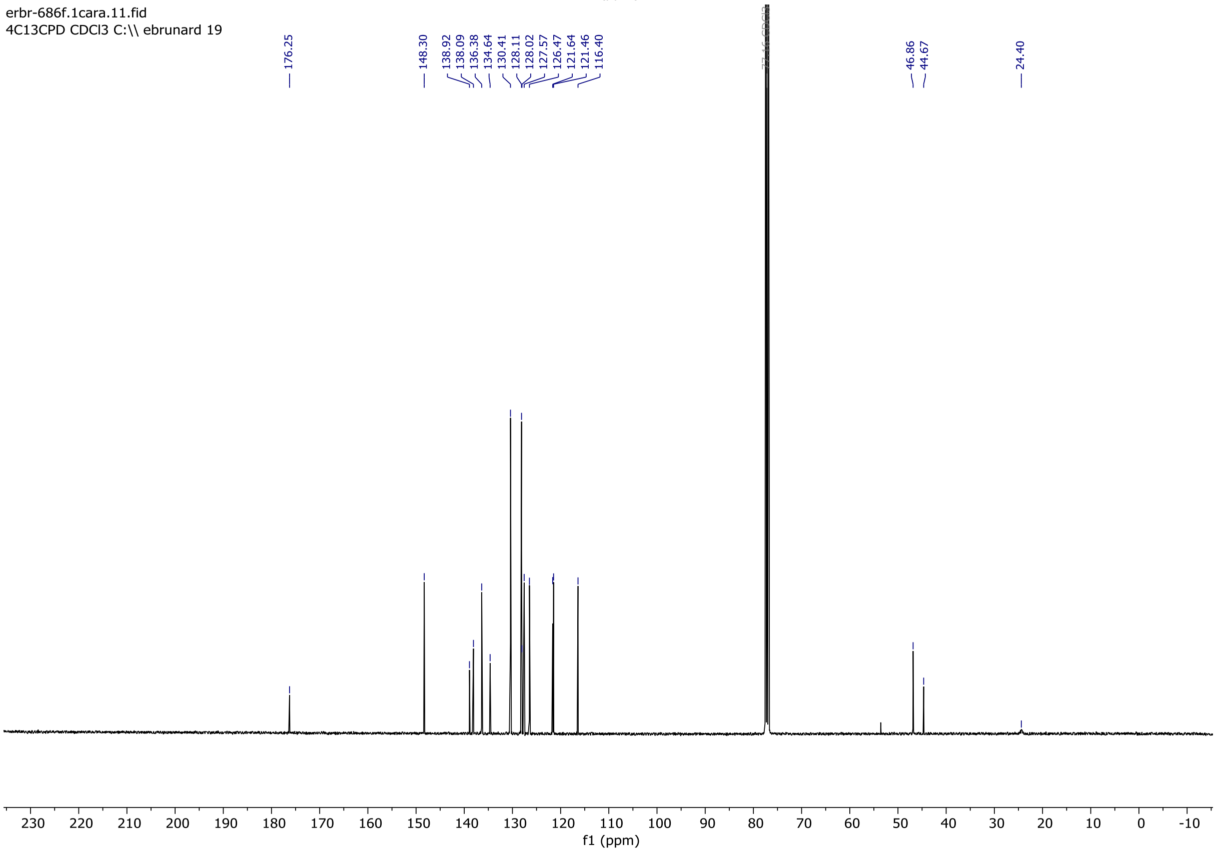

11. References

1. Motati, D. R.; Uredi, D.; Watkins, E. B. A General Method for the Metal-Free, Regioselective, Remote C–H Halogenation of 8-Substituted Quinolines. Chem. Sci. 2018, 9, 1782–1788. https://doi.org/10.1039/C7SC04107A.
2. Aihara, Y.; Chatani, N. Nickel-Catalyzed Reaction of C–H Bonds in Amides with I2: ortho-Iodination via the Cleavage of C(sp2)–H Bonds and Oxidative Cyclization to β-Lactams via the Cleavage of C(sp3)–H Bonds. ACS Catal. 2016, 6, 4323–4329. https://doi.org/10.1021/acscatal.6b00964.
3. Chen, H.; Li, P.-H.; Wang, M.; Wang, L. Transition-Metal-Free Regioselective C–H Bond Fluorination of 8-Amidoquinolines with Selectfluor. Eur. J. Org. Chem. 2018, 2018, 2091–2097. https://doi.org/10.1002/ejoc.201800389.
4. G Wang, J Han, K Wang, H Li, G Duan, C Xia, F Li (2018) Methoxylation on the C5 and C6 positions of quinolines with methanol- *Catal. Commun*, *114*, 37-41. https://doi.org/10.1016/j.catcom.2018.05.021
5. Li, Y.; Zhu, L. Z.; Cao, X.; Au, C.-K.; Qiu, R. H.; Yin, S.-F. Metal-Free C5–H Bromination of Quinolines for One-Pot C–X (X = C, O, S) Bond Formations. Adv. Synth. Catal. 2017, 359, 2864–2873. https://doi.org/10.1002/adsc.201700391.
6. Srinivas, D.; Satyanarayana, G. Palladium-Catalyzed Distal m-C–H Functionalization of Arylacetic Acid Derivatives. Org. Lett. 2021, 23, 7353–7358. https://doi.org/10.1021/acs.orglett.1c02460.
7. Romanov-Michailidis, F.; Guénée, L.; Alexakis, A. Enantioselective Organocatalytic Fluorination-Induced Wagner–Meerwein Rearrangement. Angew. Chem., Int. Ed. 2013, 52, 9266–9270. https://doi.org/10.1002/anie.201303527.
8. Nistanaki, S. K.; Williams, C. G.; Wigman, B.; Wong, J. J.; Haas, B. C.; Popov, S.; Werth, J.; Sigman, M. S.; Houk, K. N.; Nelson, H. M. Catalytic Asymmetric C–H Insertion Reactions of Vinyl Carbocations. Science 2022, 378, 1085–1091. https://doi.org/10.1126/science.ade5320.
9. Lindsay, V. N. G.; Charette, A. B. Design and Synthesis of Chiral Heteroleptic Rhodium(II) Carboxylate Catalysts: Experimental Investigation of Halogen Bond Rigidification Effects in Asymmetric Cyclopropanation. ACS Catal. 2012, 2, 1221–1225. https://doi.org/10.1021/cs300214v.
10. Yao, Q.-J.; Chen, J.-H.; Song, H.; Huang, F.-R.; Shi, B.-F. Cobalt/Salox-Catalyzed Enantioselective C–H Functionalization of Arylphosphinamides. Angew. Chem., Int. Ed. 2022, 61, e202202892. https://doi.org/10.1002/anie.202202892.
11. Chang, X.; Zhang, Q.; Guo, C. Switchable Smiles Rearrangement for Enantioselective O-Aryl Amination. Org. Lett. 2019, 21, 4915–4918. https://doi.org/10.1021/acs.orglett.9b01848.
12. González, J. M.; Vidal, X.; Ortuño, M. A.; Mascareñas, J. L.; Gulías, M. Chiral Ligands Based on Binaphthyl Scaffolds for Pd-Catalyzed Enantioselective C–H Activation/Cycloaddition Reactions. J. Am. Chem. Soc. 2022, 144, 21437–21442. https://doi.org/10.1021/jacs.2c09479.
13. Aihara, Y.; Chatani, N. Nickel-Catalyzed Direct Arylation of C(sp3)–H Bonds in Aliphatic Amides via Bidentate-Chelation Assistance. J. Am. Chem. Soc. 2014, 136, 898–901. https://doi.org/10.1021/ja411715v.
14. Li, M.-L.; Dong, J.-X.; Huang, X.-L.; Li, K.-Z.; Wu, Q.; Song, F.-J.; You, J.-S. Nickel-Catalyzed Chelation-Assisted Direct Arylation of Unactivated C(sp3)–H Bonds with Aryl Halides. Chem. Commun. 2014, 50, 3944–3946. https://doi.org/10.1039/C4CC00716F.
15. Wang, X.; Xie, P.; Qiu, R.; Zhu, L.; Liu, T.; Li, Y.; Iwasaki, T.; Au, C.-T.; Xu, X.; Xia, Y.; et al. Nickel-Catalysed Direct Alkylation of Thiophenes via Double C(sp3)–H/C(sp2)–H Bond Cleavage: The Importance of KH2PO4. Chem. Commun. 2017, 53, 8316–8319. https://doi.org/10.1039/C7CC04252C.
16. Frisch, M. J.; Trucks, G. W.; Schlegel, H. B.; Scuseria, G. E.; Robb, M. A.; Cheeseman, J. R.; Scalmani, G.; Barone, V.; Petersson, G. A.; Nakatsuji, H.; et al. Gaussian 16, Revision C.01; Gaussian, Inc.: Wallingford CT, 2016.
17. Becke, A. D. Density-Functional Thermochemistry. III. The Role of Exact Exchange. J. Chem. Phys. 1993, 98, 5648–5652. https://doi.org/10.1063/1.464913.

18. Grimme, S.; Antony, J.; Ehrlich, S.; Krieg, H. A Consistent and Accurate Ab Initio Parametrization of Density Functional Dispersion Correction (DFT-D) for the 94 Elements H–Pu. J. Chem. Phys. 2010, 132, 154104. https://doi.org/10.1063/1.3382344.
19. Grimme, S.; Ehrlich, S.; Goerigk, L. Effect of the Damping Function in Dispersion Corrected Density Functional Theory. J. Comput. Chem. 2011, 32, 1456–1465. https://doi.org/10.1002/jcc.21759.
20. Rassolov, V. A.; Pople, J. A.; Ratner, M. A.; Windus, T. L. 6-31G* Basis Set for Atoms K through Zn. J. Chem. Phys. 1998, 109, 1223–1229. https://doi.org/10.1063/1.476673.
21. Hay, P. J.; Wadt, W. R. Ab Initio Effective Core Potentials for Molecular Calculations. Potentials for K to Au Including the Outermost Core Orbitals. J. Chem. Phys. 1985, 82, 299–310. https://doi.org/10.1063/1.448975.
22. Marenich, A. V.; Cramer, C. J.; Truhlar, D. G. Universal Solvation Model Based on Solute Electron Density and a Continuum Model of the Solvent Defined by the Bulk Dielectric Constant and Atomic Surface Tensions. J. Phys. Chem. B 2009, 113, 6378–6396. https://doi.org/10.1021/jp810292n.
23. Legault, C. Y. CYLview 1.0; Université de Sherbrooke: Sherbrooke, QC, Canada, 2020. http://www.cylview.org.
24. Johnson, E. R.; Keinan, S.; Mori-Sánchez, P.; Contreras-García, J.; Cohen, A. J.; Yang, W. Revealing Noncovalent Interactions. J. Am. Chem. Soc. 2010, 132, 6498–6506. https://doi.org/10.1021/ja100936w.
25. Lu, T.; Chen, F. Multiwfn: A Multifunctional Wavefunction Analyzer. J. Comput. Chem. 2012, 33, 580–592. https://doi.org/10.1002/jcc.22885.
